# The Acidocerinae (Coleoptera, Hydrophilidae): taxonomy, classification, and catalog of species

**DOI:** 10.3897/zookeys.1045.63810

**Published:** 2021-06-18

**Authors:** Jennifer C. Girón, Andrew Edward Z. Short

**Affiliations:** 1 Department of Entomology, Purdue University, West Lafayette, Indiana, USA Purdue University West Lafayette United States of America; 2 Natural Science Research Laboratory, Museum of Texas Tech University, Lubbock, Texas, USA Natural Science Research Laboratory, Museum of Texas Tech University Lubbock United States of America; 3 Department of Ecology and Evolutionary Biology, and Division of Entomology, Biodiversity Institute, University of Kansas, Lawrence, KS 66045, USA University of Kansas Lawrence United States of America

**Keywords:** aquatic beetles, distribution, new taxa, nomenclature, references, water scavenger beetles

## Abstract

The cosmopolitan subfamily Acidocerinae (Coleoptera: Hydrophilidae) is one of the largest and most taxonomically challenging lineages of water scavenger beetles. Recent phylogenetic studies have substantially advanced our understanding of acidocerine relationships but also illuminated the twin challenges of poorly delineated generic concepts and a classification broadly incompatible with the phylogeny. Here, these two challenges are addressed by providing a comprehensive synthesis and taxonomic tools for the Acidocerinae, including (1) a brief history and the current state of acidocerine classification, (2) a review of acidocerine ecology and collection methods, (3) the current knowledge of larval and fossil acidocerines, (4) a morphological primer on characters of taxonomic and systematic importance within the lineage, (5) a key to the world genera of Acidocerinae, (6) diagnoses, habitus, and aedeagal images, distribution maps, and summary of knowledge for each of the 23 extant genera in the subfamily, and (7) a complete annotated taxonomic catalog including the published distributions, synonyms, and references for all described 541 acidocerine species recognized as of 1 April 2021. The following nomenclatural acts are proposed to bring the phylogeny and classification into alignment: *Colossochares***gen. nov.** is established to accommodate two African species previously described as *Helochares* (s. str.); *Novochares***gen. nov.** is newly established to accommodate 15 Neotropical species previously included in *Helochares* (s. str.); the remaining *Helochares* subgenera *Helocharimorphus* Kuwert **syn. nov.** and *Hydrobaticus* MacLeay **syn. nov.** are synonymized with *Helochares* Mulsant. *Peltochares* Régimbart **sensu nov.** is redefined to include eight Old World species previously included in *Helochares* (s. str.). A lectotype is designated for *Peltochares conspicuus* Régimbart, the type species of the genus. The taxonomic and morphological circumscription of *Helochares***sensu nov.** is narrowed and redefined.

## Abstract (sp)

La subfamilia Acidocerinae (Coleoptera: Hydrophilidae) es cosmopolita y representa uno de los linajes más diversos y taxonómicamente más desafiantes de escarabajos acuáticos detritívoros. Estudios filogenéticos recientes han incrementado considerablemente nuestro entendimiento sobre las relaciones entre acidocerinos, así como iluminaron los conceptos genéricos pobremente definidos y una clasificación ampliamente incompatible con la filogenia. Aquí se abordan estos dos desafíos proporcionando una síntesis integral y herramientas taxonómicas para Acidocerinae, incluyendo (1) un resumen de la historia y estado actual de la clasificación de Acidocerinae, (2) una revisión de la ecología y los métodos de recolección para acidocerinos, (3) el conocimiento actual de acidocerinos larvales y fósiles, (4) un manual morfológico básico sobre caracteres de importancia taxonómica y sistemática dentro del linaje, (5) una clave para los géneros de Acidocerinae del mundo, (6) diagnosis, imágenes del hábito y del edeago, mapas de distribución y resumen del conocimiento actual para cada uno de los 23 géneros existentes en la subfamilia, y (7) un catálogo taxonómico anotado y completo que incluye las distribuciones publicadas, sinónimos y referencias para todas las 541 especies de Acidocerinae descritas y reconocidas al 1 de abril de 2021. Se proponen los siguientes actos taxonómicos para alinear la clasificación con la filogenia: *Colossochares***gen. nov.** se establece para incluir dos especies africanas descritas previamente como *Helochares* (s. str.); *Novochares***gen. nov.** se establece como nuevo para acomodar 15 especies neotropicales previamente incluidas en *Helochares* (s. str.); los subgéneros restantes de *Helochares* Mulsant, *Helocharimorphus* Kuwert **syn. nov.** e *Hydrobaticus* MacLeay **syn. nov.** se sinonimizan con *Helochares*. *Peltochares* Régimbart **sensu nov.** es redefinido para incluir ocho especies del viejo mundo previamente incluidas en *Helochares* (s. str.). Se designa un lectotipo para *Peltochares conspicuus* Régimbart, la especie tipo del género. La circunscripción taxonómica y morfológica de *Helochares***sensu nov.** se reduce y redefine.

## Introduction

The water scavenger beetle family Hydrophilidae Latreille, with more than 3,000 described species, is the most diverse family of polyphagan aquatic beetles, and the second largest for all aquatic Coleoptera (Short 2018). This diversity is reflected in their species richness and their ecological habits: members of the family are associated not only with aquatic ecologies, but also various hygropetric and a broad range of terrestrial habitats (Bloom et al. 2014). A comprehensive molecular phylogeny for the family by Short and Fikáček (2013) organized the lineage into six subfamilies: Hydrophilinae Latreille, Chaetarthriinae Bedel, Enochrinae Thomson, Acidocerinae Zaitzev, Cylominae Zaitzev (changed from Rygmodinae d’Orchymont; Seidel et al. 2016), and Sphaeridiinae Latreille. With more than 500 species, the Acidocerinae is the third largest hydrophilid subfamily (after Hydrophilinae and Sphaeridiinae). The Acidocerinae occupies a key position in the evolutionary history and in the broader ecological evolution of water scavenger beetles, as it diverges after the primarily aquatic Hydrophilinae, Chaetarthriinae and Enochrinae, while serving as the sister group to the largely terrestrial Cylominae+Sphaeridiinae (Short and Fikáček 2013).

**Figure 1.**
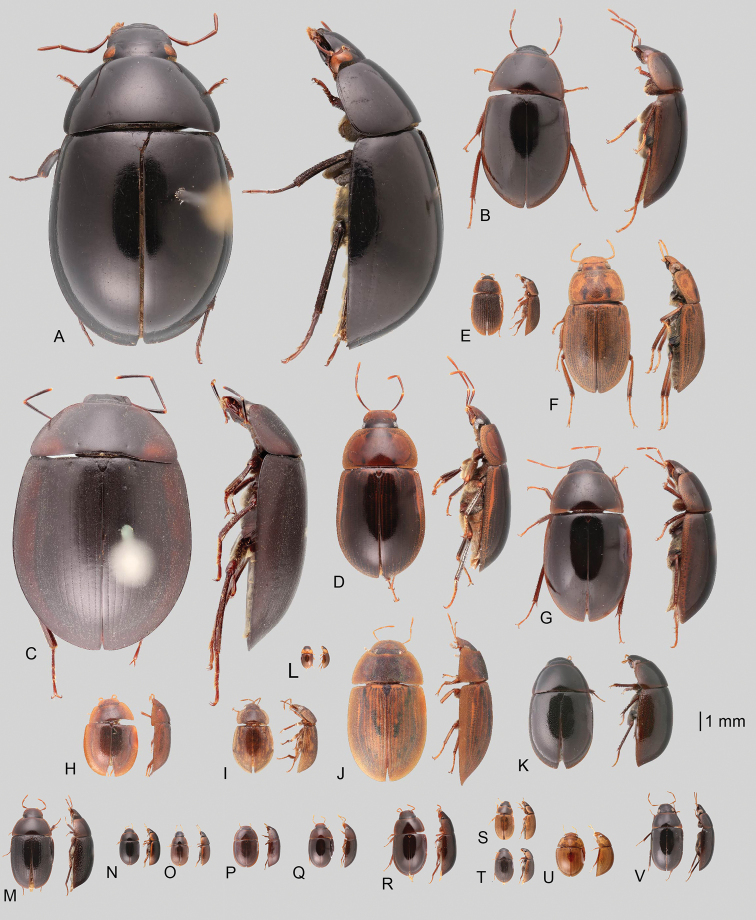
Variation across Acidocerinae, dorsal and lateral views **A***Colossochares ellipticus***B***Peltochares* sp. **C***Peltochares conspicuus***D***Aulonochares tubulus***E***Helochares* sp. **F***Helochares tristis***G***Novochares* sp. **H***Helopeltarium ferrugineum***I***Batochares* sp. **J***Helobata larvalis***K***Radicitus* sp. **L***Nanosaphes tricolor***M**Agraphydrus cf. attenuatus**N***Tobochares luteomargo***O***Tobochares sulcatus***P***Quadriops similaris***Q***Crucisternum ouboteri***R***Primocerus neutrum***S***Agraphydrus coomani***T***Agraphydrus* sp. **U***Globulosis flavus***V***Crephelochares nitescens*.

In morphological terms, Acidocerinae is a heterogeneous assemblage of beetles, as a variety of sizes, colorations and body shapes can be found in the group (Fig. 1). Species range in size from 1.1 mm (*Nanosaphes* Girón & Short; Figs 1L, 41) to 14 mm (*Colossochares* gen. nov.; Figs 1A, 26) and range in color from pale yellowish and orange brown to nearly black (Fig. 1). Body forms vary from compact and convex (e.g., *Globulosis* García; Figs 1U, 32) to broadly explanate and dorsoventrally compressed (e.g., *Helobata* Bergroth, Figs 1J, 33; *Helopeltarium* d’Orchymont, Figs 1H, 38). Although most genera are relatively easy to tell apart, within a genus, the external morphology ranges from extremely homogeneous (e.g., *Aulonochares* Girón & Short; Figs 1D, 21) to highly variable (e.g., *Primocerus* Girón & Short, Figs 1R, 46; *Agraphydrus* Régimbart, Figs 1S, T, 18, 19). This morphological diversity, which may be a consequence of adapting to the broad range of habitats where acidocerines occur, and compounded by the widespread distribution of some taxa, has resulted in taxonomic confusion. Acidocerine species can be found across a wide variety of environments, spanning almost the full range of habitats that occur in the Hydrophilidae as a whole, including fully aquatic settings like ponds, streams, and river margins, hygropetric habitats like rock seepages, and terrestrial niches such as rotting fruits.

Although the circumscription of the subfamily is well supported by several molecular studies (Short and Fikáček 2013; Short et al. 2021) the morphological diversity of acidocerines has befuddled efforts to define the lineage as a whole, as well as many of its historical genera. There is presently no known synapomorphy for the lineage that does not have at least one exception. Additionally, rampant homoplasy in certain characters that have historically been used to circumscribe genera and subgenera (such as the presence of elytral striae and the length of the maxillary palps) have significantly complicated acidocerine taxonomy. A recent comprehensive molecular phylogeny of the subfamily (Short et al. 2021) combined with an explosion of new genera and species from all parts of the world created both the opportunity and the need for a comprehensive taxonomic review of the Acidocerinae. In this work, we provide an integrated synthesis and taxonomic tools for the Acidocerinae, including (1) a brief history and the current state of acidocerine classification, (2) a review of acidocerine ecology and collection methods, (3) the current knowledge of larval and fossil acidocerines, (4) a morphological primer on characters of taxonomic and systematic importance within the lineage, (5) a key to the world genera of Acidocerinae, (6) descriptions, differential diagnoses, habitus and aedeagal images, distribution maps, and summary of knowledge for each of the 23 extant genera in the subfamily, and (7) a complete annotated taxonomic catalog including the published distributions, synonyms, and references for all described acidocerine species.

## Taxonomic history and composition of the Acidocerinae

Horn (1873) established the monogeneric tribe Helopeltini for the newly established genus *Helopeltis* (now *Helobata*; Figs 1J, 33). Horn (1873) viewed the genus as quite distinct and warranting its own tribe based on the broadly explanate body form, concealed labrum, and long maxillary palps (he retained *Helochares*, the only other Acidocerinae [in the current sense] in North America at the time, within the Hydrobiini with most other hydrophilids). However, Helopeltini was unavailable due to its type genus *Helopeltis* being a preoccupied name (Hansen 1999b). Later, Zaitzev (1908) placed the genus *Acidocerus* Klug (Fig. 17) into its own “subfamily” under the new name Acidocerini without comment. It is unclear why he considered the taxon so unique as to give it such a prominent rank in his classification, which placed it equal to the rank he considered for Epimetopidae, Spercheidae, and other currently recognized hydrophiloid families. A decade later, d’Orchymont (1919c), either unaware or unconcerned with the Acidocerini of Zaitzev, proposed the subtribe Helocharae for *Helochares*, *Enochrus*, and their apparent relatives (including *Acidocerus*). Unlike Helopeltini and Acidocerini, the erection of Helocharae was not done to bestow recognition on a single bizarre taxon, but to unite a morphologically similar collection of genera. The name and concept of the Helocharae (either as a subtribe of Hydrobiini or as the tribe Helocharini (of Hydrobiinae) remained in use for the next 70 years.

Hansen (1991) was the first to both recognize Zaitzev’s Acidocerini as having priority over Helocharae and to affirm the circumscription of the lineage in a phylogenetic context (as the subtribe Acidocerina of Hydrophilini). Twenty years later, Short and Fikáček (2011), elevated the Acidocerini to tribal level, citing accumulating evidence that the Hydrophilini sensu Hansen was not monophyletic. In a subsequent comprehensive molecular phylogeny and reclassification of the Hydrophilidae, Short and Fikáček (2013) elevated the lineage further to its current subfamily rank, while transferring *Enochrus* Thomson, *Cymbiodyta* Bedel, and *Helocombus* Horn from the Acidocerinae into the newly defined subfamily Enochrinae. This circumscription has remained unchanged to date.

In terms of diversity, Acidocerinae included nearly 300 species grouped in 14 genera when it was first recognized as a subfamily (*Acidocerus*, *Agraphydrus*, *Chasmogenus* Sharp, *Dieroxenus* Spangler, *Globulosis*, *Helochares*, *Helobata*, *Helopeltarium*, *Horelophopsis* Hansen, *Megagraphydrus* Hansen, *Peltochares*, *Quadriops* Hansen, *Tobochares* Short & García, and *Troglochares* Spangler; Short and Fikáček 2013). Since then, six genera have been described (*Crucisternum* Girón & Short, *Katasophistes* Girón & Short, and *Nanosaphes*, Girón & Short, 2018; *Aulonochares*, *Primocerus*, and *Ephydrolithus* Girón & Short, 2019), and two genera have been synonymized (*Dieroxenus* synonym of *Chasmogenus*; Girón and Short 2018; *Horelophopsis* synonym of *Agraphydrus*; Short et al. 2021).

The most comprehensive molecular phylogenetic analysis of the subfamily Acidocerinae was recently conducted by Short et al. (2021). The dataset included DNA sequence data for the mitochondrial gene COI and the nuclear genes 18S, 28S, H3, and CAD, for 206 acidocerine and eleven outgroup terminals (Short et al. 2021). These analyses confirmed the monophyly of the subfamily, as well as of most genera, with the unsurprising exception of a polyphyletic *Helochares* (Short et al. 2021: figs 1, 2).

**Figure 2.**
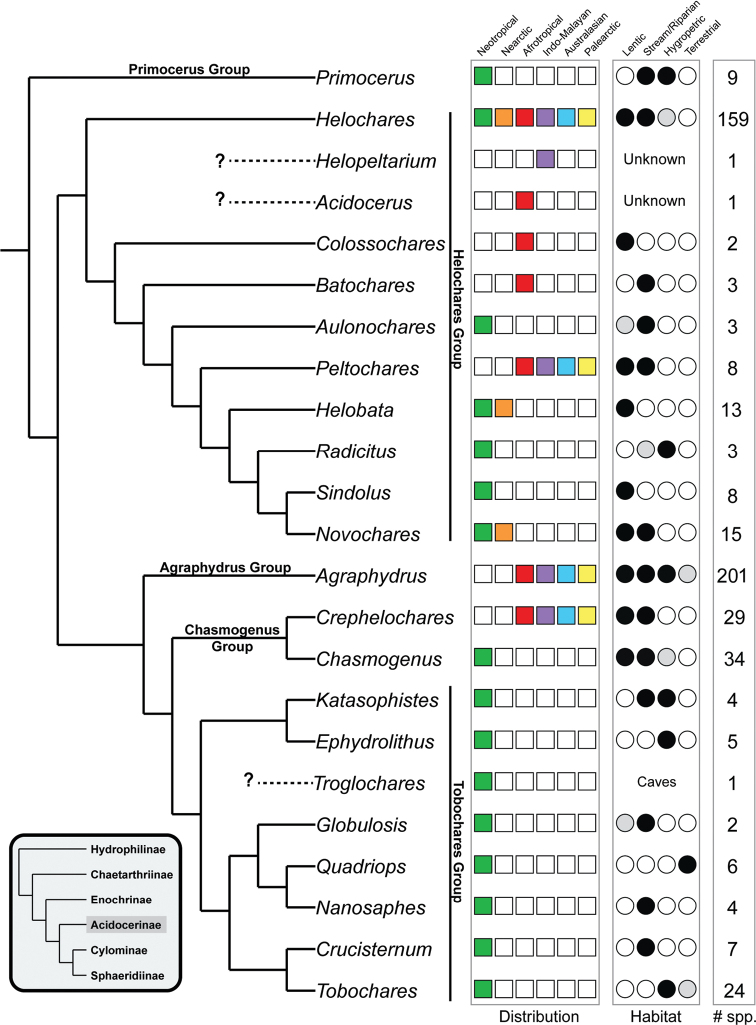
Phylogeny of the Acidocerinae simplified from Short et al. (2021), indicating the distribution, preferred habitat, and currently described number of species for each genus. For habitat, filled black circles indicate that at least some species of the genus are commonly found in this habitat; light grey circles indicate the genus has been found in this habitat, but is rare or not typical for the group; white circles indicate no species have been recorded for the genus in this habitat.

### The *Helochares* problem

At the time Acidocerinae was elevated to subfamily, *Helochares* was its largest and most widespread genus, grouping nearly 2/3 of the species in the lineage. *Helochares* was traditionally divided into five subgenera: *Batochares* Hansen (e.g., Figs 1I, 23), *Helochares* (e.g., Fig. 1B), *Helocharimorphus* Kuwert (e.g., Fig. 35D–F), *Hydrobaticus* MacLeay (e.g., Figs 35A–C, 36A–C) and *Sindolus* Sharp (e.g., Fig. 51), some of which were recognized mostly by the absence [*Helochares* (s. str.)] or presence [Helochares (Hydrobaticus)] of rows of serial punctures along the elytra.

The phylogeny presented by Short et al. (2021; figs 1, 2 therein) provided evidence for elevating *Batochares* and *Sindolus* to full generic status, as well as for synonymizing *Helocharimorphus* and *Hydrobaticus* with *Helochares*. Nevertheless, there are several taxonomic issues within *Helochares* left unresolved, which we aim to sort out here. In addition, it is now clear that the presence of rows of serial punctures along the elytra is not necessarily a reliable character to recognize genera (or subgenera) within Acidocerinae, whereas the configuration of the male genitalia, which is much more conserved within clades, is very useful for recognizing allied species.

### Updating the classification of the Acidocerinae

Based on their phylogeny, Short et al. (2021) defined five monophyletic genus groups within the Acidocerinae (Fig. 2): the *Primocerus* group (including only *Primocerus*; *Helochares* group (including *Helochares*, *Colossochares* gen. nov., *Batochares*, *Aulonochares*, *Peltochares*, *Helobata*, *Radicitus*, *Sindolus*, and *Novochares* gen. nov.), *Agraphydrus* group (including only *Agraphydrus*), *Chasmogenus* group (*Chasmogenus* and *Crephelochares*), and *Tobochares* group (*Katasophistes*, *Ephydrolithus*, *Globulosis*, *Quadriops*, *Nanosaphes*, *Crucisternum*, and *Tobochares*).

*Colossochares* gen. nov. is established to accommodate two African species previously described as *Helochares* (s. str.) (Fig. 2; *Helochares* Clade B in Short et al. 2021: fig. 2). *Peltochares* sensu nov. is hereby redefined to include eight Old World species previously described as *Helochares* (s. str.) (Fig. 2; *Helochares* Clade C in Short et al. 2021: fig. 2); a lectotype is designated for its type species *P. conspicuus* Régimbart. *Novochares* gen. nov. is newly established to accommodate 15 Neotropical species previously described as *Helochares* (s. str.) (Fig. 2; *Helochares* Clade D in Short et al. 2021: fig. 2). *Helochares* sensu nov. is redefined, including 159 species world-wide distributed (Fig. 1; *Helochares* Clade A in Short et al. 2021: fig. 1). After the publication of a series of revisions of the genus *Agraphydrus* (Komarek and Hebauer 2018; Komarek 2018, 2019, 2020; Komarek and Freitag 2020), *Helochares* is now the second largest genus in number of species.

### Genus groups within the Acidocerinae

Although the Acidocerinae is the third largest subfamily of Hydrophilidae and is experiencing a rapid growth in diversity, it is not partitioned into tribes as the largest two subfamilies are (Sphaeridiinae and Hydrophilinae). Although there do seem to be reciprocally monophyletic lineages that could serve as tribes, some do not have clear or unambiguous morphological synapomorphies and are therefore very difficult to diagnose. Instead, Short et al. (2021) established five genus groups in place of formal tribes.

#### *Primocerus* group

This group contains a single Neotropical genus, *Primocerus* with nine described species. The group is defined by the lack of a distinct sclerotized gonopore and the presence of a sclerotized projection at the apex of the median lobe. However, it is more readily recognized by the presence of a sharp sutural stria, which is otherwise only found in members of the *Chasmogenus* group. As such, care must be taken to separate *Primocerus* and *Chasmogenus*, as the genera overlap in the Guiana Shield region of South America; the condition of the posterior elevation of the mesoventrite is a useful character to distinguish them.

#### *Helochares* group

The *Helochares* group is the largest lineage of Acidocerinae, which contains 11 genera with a combined 213 species. It is extremely heterogeneous in body form, containing species from very small (e.g., 2 mm in some *Helochares*) to the largest acidocerine, *Colossochares ellipticus* (d’Orchymont). The group is distributed worldwide. There is no clear unique morphological synapomorphy for the lineage, but it exhibits a putative behavioral synapomorphy: the females of most (if not all) species in the group carry around their egg case attached to the ventral surface of the abdomen.

#### *Agraphydrus* group

The *Agraphydrus* group contains a single genus (*Agraphydrus*) that is distributed primarily in the Old World tropics, particularly southeast Asia. The group has exploded in diversity over the last few years, as more than 100 species have been described in a multi-part revision starting in 2018 (Komarek and Hebauer 2018). Potential synapomorphies for the *Agraphydrus* group include the V-shaped abdominal sternite 9 (Minoshima 2016). Although all placed within a single genus, the morphological variation is rather broad (though perhaps not as broad as *Helochares*) and includes a variety of forms that have been at times placed in other genera, most notably two species that were not long ago placed in their own subfamily (Horelophopsinae).

#### *Chasmogenus* group

The *Chasmogenus* group contains two genera, the Neotropical-endemic *Chasmogenus* and the Old-World *Crephelochares*. The group is most easily distinguished from all others, except the *Primocerus* group, by the sharply impressed sutural striae. Indeed, in the Old World, it is the only group of Acidocerinae with sutural striae.

#### *Tobochares* group

The *Tobochares* group is comprised of seven Neotropical genera, all of which were described in the last 20 years. Although the group is well-supported as monophyletic by molecular data (Short et al. 2021), there is no clear synapomorphy that identifies membership in the lineage. All species are relatively small (most less than 3 mm), and includes the smallest known acidocerines (e.g., *Nanosaphes*, at just 1.1 mm in length).

## Materials and methods

### Morphological methods

Specimen preparation and examination methods are identical to those given in Girón and Short (2017). For each genus, a list of diagnostic character states is provided, followed by notes comparing with similar genera. Morphological terminology largely follows Hansen (1991) except for the use of meso- and metaventrite instead of meso- and metasternum, and the terminology for veins and areas of the hind wings, which follows those of Lawrence and Ślipiński 2013. Diagnoses of genera and species lists are organized in alphabetical order. Figures illustrating each genus are arranged in alphabetical sequence, but within each plate, images are organized to display variation.

### Distributional data

**Figure 3.**
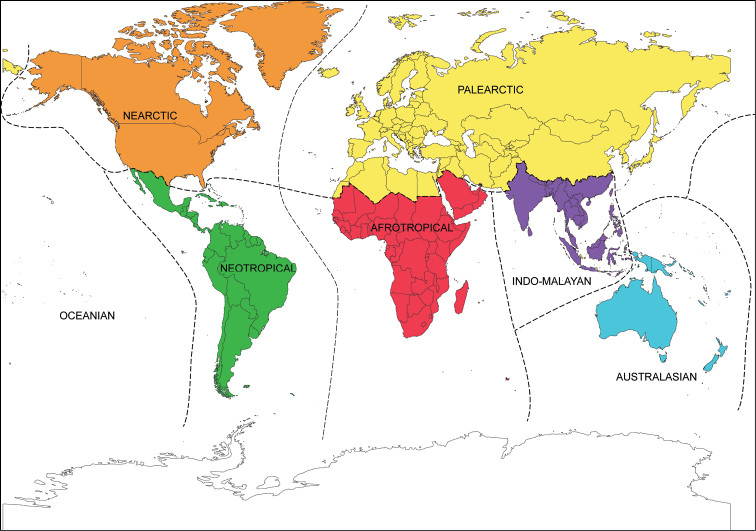
World map showing the boundaries of the biogeographic regions as used in this work, modified from Hansen (1999b).

For consistency, we followed the biogeographic regions as delimited by Hansen (1999b) with the following exceptions for convenience: Saudi Arabia is here treated entirely as Afrotropical (rather than split between Afrotropical and Palearctic regions), and India is considered entirely Indo-Malayan (rather than being split between the Indo-Malayan and Palearctic regions) (Fig. 3). To increase precision for several larger countries, records are given for the States/Provinces of Brazil, China, India, and the United States. Specimen data regarding the material examined in this study can be searched by species through the Collection Resources for Aquatic Coleoptera (CReAC) portal at http://creac.kubiodiversityinstitute.org/collections/.

Current numbers of species per genus have been consolidated and are presented for each of the regions where acidocerines occur. Known distributional information obtained from the literature has been summarized for each species and included in the catalog.

### Catalog

Each current genus or species name is followed by its original name including its full reference. A list of subsequent names and references, in chronological order, is also included where appropriate, indicating in square brackets the kind of reference involved, for example, [checklist], [redescription], [taxonomic treatment], etc. Page numbers where the taxon name appears in the text are given for each reference using colon “:” after the publication year. For the most part, the list of names is based on Hansen’s (1999b) catalog; additional references are also listed. Species described between 15 December 1999 and 1 April 2021 are added to this catalog. The full checklist of valid names is available online via GBIF (https://doi.org/10.15468/ypcrsp; Girón and Short 2021b).

## Results

### Distribution and regional diversity of Acidocerinae

Acidocerines can be found in all biogeographic regions except the Antarctic. A summary of the distributional information of each acidocerine genus is presented in Table 1. Regions correspond to those in Fig. 3. The total number of species are given per genus, per region; in parenthesis the number of species that are shared with other regions. An en-dash is used to indicate that there are no species recorded for a given genus in a given region.

**Table 1.** Distributional information for Acidocerinae. Numbers in parentheses correspond to the number of species from the region that are shared with other regions. En-dash (–) indicates that no species of the genus are recorded from that particular region.

	Afrotropical	Australasian	Indo-Malayan	Nearctic	Neotropical	Palearctic	Total
***Acidocerus*** Klug, 1855	1	–	–	–	–	–	1
***Agraphydrus*** Régimbart, 1903	30 (1)	5 (1)	162 (13)	–	–	21 (15)	201
***Aulonochares*** Girón & Short, 2019	–	–	–	–	3	–	3
***Batochares*** Hansen, 1991	3	–	–	–	–	–	3
***Chasmogenus*** Sharp, 1882	–	–	–	–	33	–	33
***Colossochares*** Girón & Short, gen. nov.	2	–	–	–	–	–	2
***Crephelochares*** Kuwert, 1890	18	3	7 (2)	–	–	3 (2)	29
***Crucisternum*** Girón & Short, 2018	–	–	–	–	7	–	7
***Ephydrolithus*** Girón & Short, 2019	–	–	–	–	5	–	5
***Globulosis*** García, 2001	–	–	–	–	2	–	2
***Helobata*** Bergroth, 1888	–	–	–	1 (1)	13 (1)	–	13
***Helochares*** Mulsant, 1844	92 (2)	16 (3*)	35 (6)	2 (2)	8 (2)	15 (5)	159
***Helopeltarium*** d’Orchymont, 1943	–	–	1	–	–	–	1
***Katasophistes*** Girón & Short, 2018	–	–	–	–	4	–	4
***Nanosaphes*** Girón & Short, 2018	–	–	–	–	4	–	4
***Novochares*** Girón & Short, gen. nov.	–	–	–	(1)	15	–	15
***Peltochares*** Régimbart, 1907	2 (1)	3 (1)	4 (1)	–	–	(1)	8
***Primocerus*** Girón & Short, 2019	–	–	–	–	9	–	9
***Quadriops*** Hansen, 1999	–	–	–	–	6	–	6
***Radicitus*** Short & García, 2014	–	–	–	–	3	–	3
***Sindolus*** Sharp, 1882	–	–	–	–	8	–	8
***Tobochares*** Short & García, 2007	–	–	–	–	24	–	24
***Troglochares*** Spangler, 1981	–	–	–	–	1	–	1
**TOTAL by region**	148	27	209	4	146	40	541

* Only one species has been recorded from the Oceanian region (Samoa, Tonga).

### Natural history and habitat preferences of Acidocerinae

Acidocerines, as a whole, occupy one of the widest habitat breadths of any aquatic beetle group, although most individual species are fairly narrow and predictable in their ecological preferences. Consequently, collecting in a variety of habitats using multiple methods is often required to adequately survey a locality.

**Collecting methods.** Members of the subfamily are generally poor swimmers, even those most commonly found in ponds and streams. They primarily move around their habitat by clinging and crawling on substrates of submerged detritus and vegetation. When dislodged, they will float to the surface of the water until they can grab onto something to pull themselves below again. Because of this, the most effective method for collecting acidocerines is typically to agitate the habitat they are living in (e.g., detritus, emergent vegetation, etc.) and collect them either by hand or with a small strainer or sieve when they float to the surface. For example, vigorously treading along the margin of a marsh or pond (Fig. 7C) will cause many non-swimming hydrophilids to rise to the surface for easy collection. In habitats where this is difficult, the vegetation or detritus can be submerged and agitated in a pan or bucket of water to create the same effect (Fig. 8D). Likewise, the pan flotation method is also effective for seepage taxa, where the moss, detritus, or other seepage debris can be put in a pan of water and the specimens floated out of it.

Some species readily come to lights, occasionally in large numbers, especially those that live in open marsh and other similar lentic type habitats. Flight intercept traps (FITs) have been effective for collecting select taxa in dense tropical forests. While FITs do not generally produce high volumes of acidocerine specimens, they have been effective at trapping species that are rare or otherwise may miss detection. This is especially true for species that are not found in traditional aquatic habitats. For instance, early collections of the genus *Quadriops* were almost exclusively known from FIT samples, prior to our knowledge that it was a terrestrial genus. Malaise traps are generally ineffective at surveying acidocerines, and water beetles in general.

**Open marsh and pond habitats.** Open, exposed lentic habitats such as shallow marshes (Fig. 7C), pond margins (Fig. 7B), and vegetated ditches (Fig. 7A) are perhaps thought of as being the most “classical” habitat for acidocerines. This includes the largely slack-water margins and floating macrophytes of large rivers. Most acidocerines are found in shallow and/or marginal areas, or in areas with abundant emergent vegetation or detritus. Because they are clingers/crawlers, they will not be found in deep water or in areas that are devoid of ample detritus or vegetation in which to hide or cling to. This is a common habitat for many *Helochares* and *Novochares* species, and the near-exclusive habitat of *Sindolus* and *Helobata*. Other genera such as *Chasmogenus*, *Crephelochares*, and *Agraphdyrus* that are mostly found in other habitats have at least one open lentic species.

**Figure 4.**
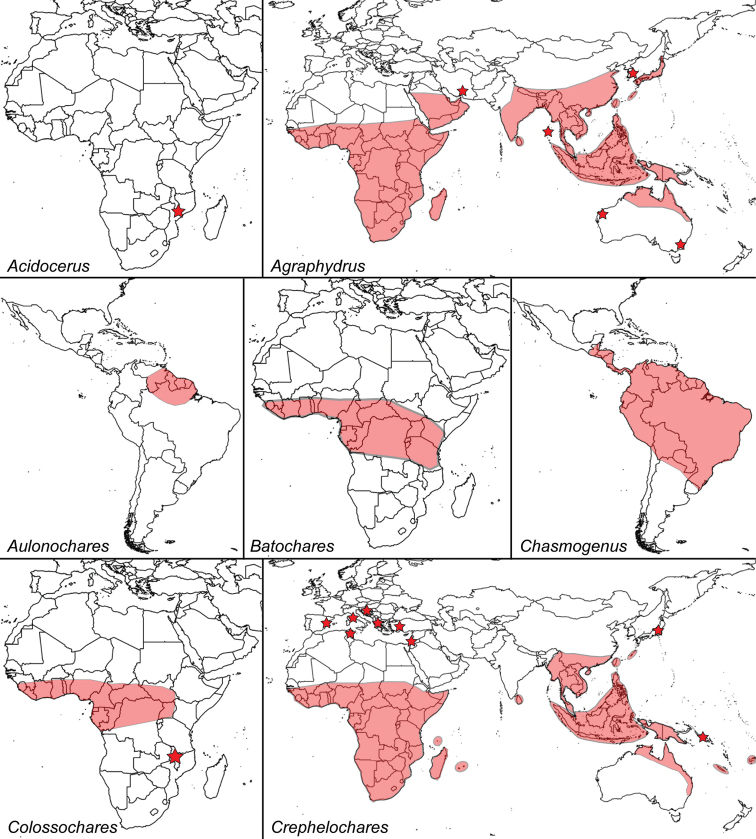
Known distribution of genera of Acidocerinae: *Acidocerus*, *Agraphydrus*, *Aulonochares*, *Batochares*, *Chasmogenus*, *Colossochares*, and *Crephelochares*.

**Figure 5.**
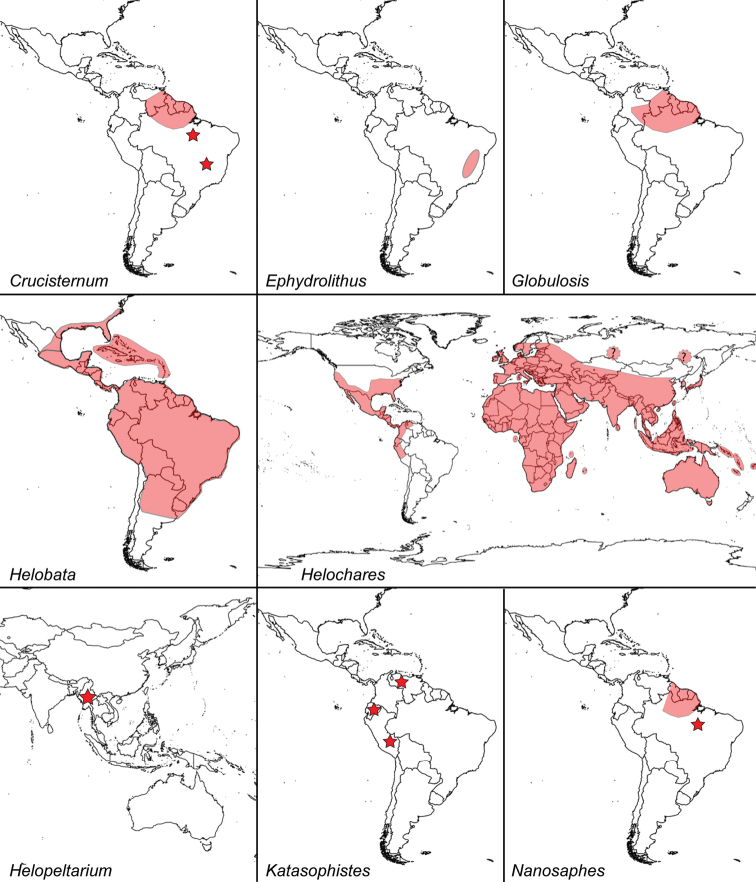
Known distribution of genera of Acidocerinae: *Crucisternum*, *Ephydrolithus*, *Globulosis*, *Helobata*, *Helochares*, *Helopeltarium*, *Katasophistes*, and *Nanosaphes*.

**Forested lentic habitats.** Standing water habitats such as forested pools (Fig. 7E, F) and shallow swamps (Fig. 7D) can be extremely productive for collecting acidocerines, especially when there is abundant detritus. Shallow detrital pools, especially in the early to mid-dry season when they are contracting, can contain abundant acidocerines. In the Neotropics, this is the most common habitat for species of *Novochares* and *Chasmogenus*. We presume that similar habitats in Africa and Asia would be productive for *Helochares*, *Crephelochares*, and *Peltochares*.

**Figure 6.**
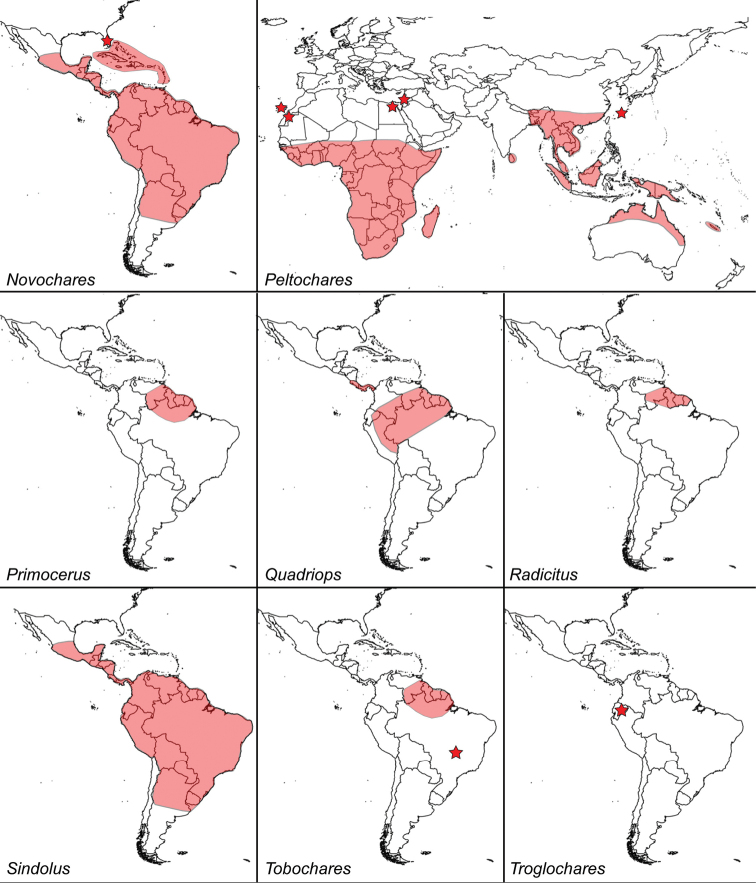
Known distribution of genera of Acidocerinae: *Novochares*, *Peltochares*, *Primocerus*, *Quadriops*, *Radicitus*, *Sindolus*, *Tobochares*, and *Troglochares*.

**Figure 7.**
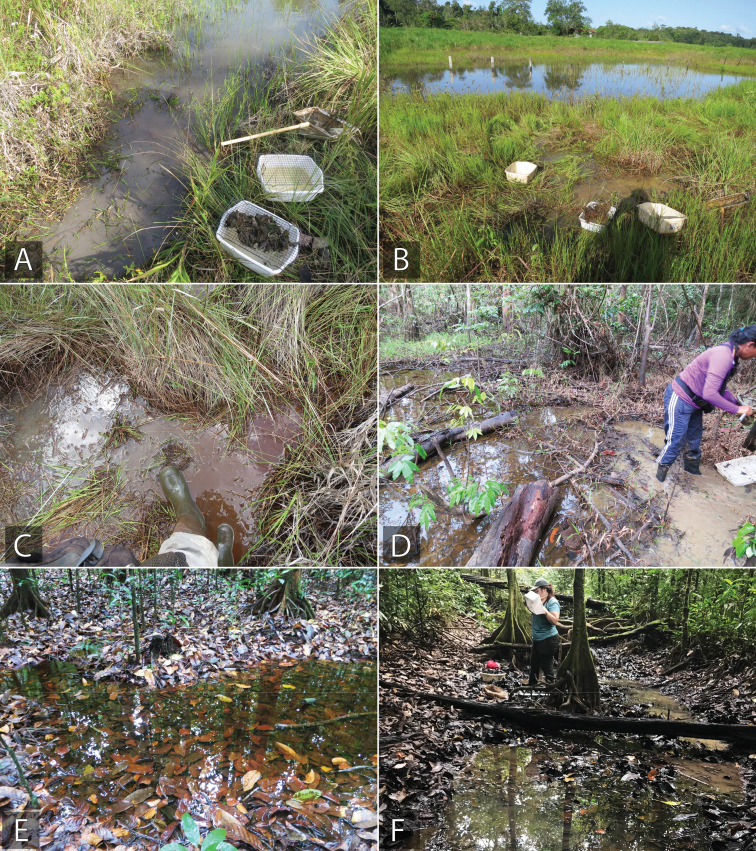
Examples of open and forested lentic habitat for Acidocerinae**A** vegetated ditch **B** pond margin (Brazil: BR18-0720-04A) **C** stomping vegetation and substrate in a shallow marsh or ditch (Brazil: BR18-07-01A) **D** margin of forested swamp (Brazil: BR18-0724-04A) **E** forested detrital pool (Suriname: SR13-0817-01A) **F** forested detrital pool (French Guiana: FG20-0307-01D).

**Figure 8.**
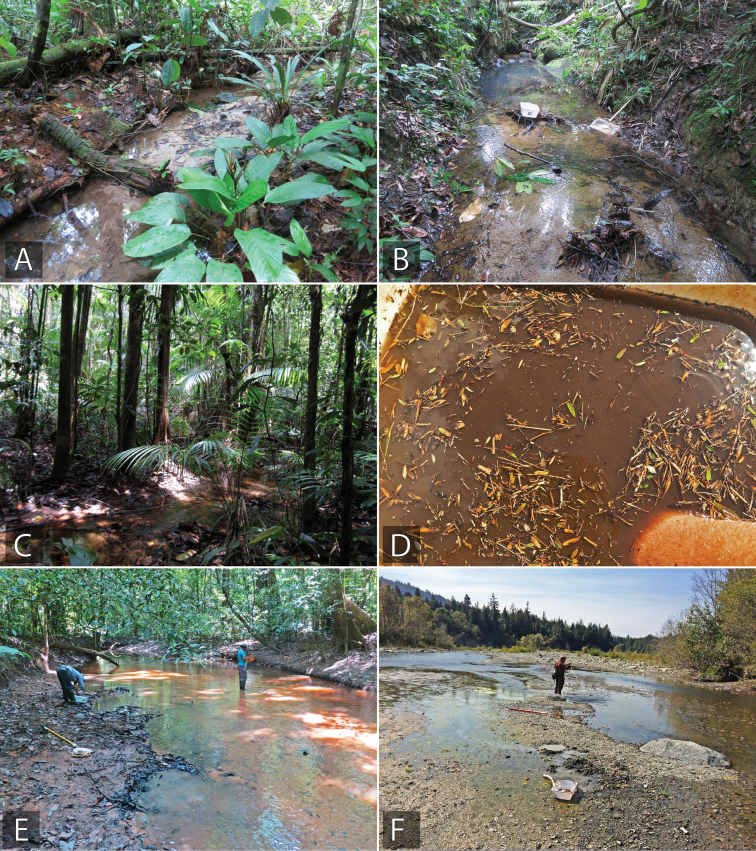
Examples of lotic and riparian habitat for Acidocerinae**A** forested stream (Suriname: SR12-0320-02A) **B** forested stream (Suriname: SR17-0331-01B) **C** forested stream (Suriname: SR10-0820-01A) **D** technique of flotation of detritus from stream margin in a white pan, a few small acidocerines can be seen floating on the surface **E** forested stream (Guyana: GY14-0925-01B) **F** open gravel stream (USA: California: US16-0908-04A).

**Stream and riparian habitats.** Lotic habitats harbor a broad range of acidocerine taxa, although these can typically be broken into two categories: (1) stream margins that are vegetated or otherwise formed by “banks” with roots (Fig. 8A–C), and (2) stream margins that are composed of sand or gravel, also including sandbars and floodplains (Fig. 8E, F). The vegetated margins of small to medium sized streams, especially those in tropical forests, are the preferred habitat for a number of genera, including *Globulosis*, *Crucisternum*, *Nanosaphes*, and *Aulonochares*. Other genera such as *Helochares*, *Novochares*, *Katasophistes*, and *Agraphydrus* have taxa that occur here as well. Sand and gravel margins of streams are also common habitats for certain acidocerinae species, but there is little overlap between the species that prefer gravel margins and those that occur in vegetated/root mat margins. In North and Central America, these sandy margins are frequently home to *Helochares normatus* (LeConte). In South America, some species of *Chasmogenus* are common in these habitats, especially in the foothills of the Andes.

**Figure 9.**
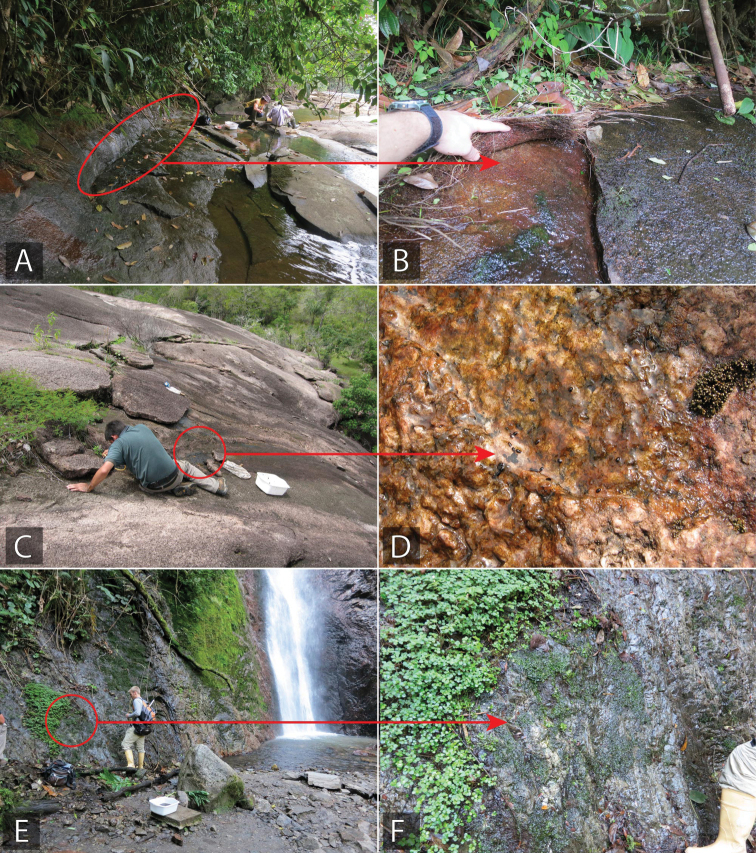
Examples of seepage habitat for Acidocerinae**A, B** marginal seepage along river (Guyana: GY14-0312-01B) **C, D** isolated seep on granite inselberg (Venezuela: VZ10-0710-01A) **E, F** hygropetric zone next to waterfall (Venezuela: VZ12-0122-03A).

**Hygropetric and seep habitats.** Hygropetric habitats encompass a surprisingly diverse array of microhabitats that are generally characterized by thin water films flowing or seeping over rocky substrate. These habitats most frequently occur in association with (and connected to) rivers and streams, such as in misting or trickle zones adjacent to waterfalls (Fig. 9E, F), or where streams flow over or near expanses of rock (Fig. 9A, B). Others may be isolated or self-contained, such as the seasonal seeps that form on inselbergs and are not necessarily connected to a larger lotic network (Fig. 9C, D). The genera *Tobochares*, *Ephydrolithus*, *Radicitus*, and *Primocerus* almost exclusively occur in seepage habitats. Many other genera have at least one hygropetric specialist, including *Agraphydrus* (numerous), *Katasophistes* (*K. merida* Girón & Short), and *Chasmogenus* (*C. cremnobates* (Spangler)).

**Figure 10.**
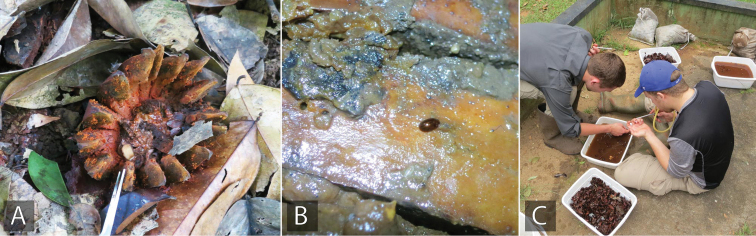
Examples of terrestrial habitat for Acidocerinae**A, B** Rotting *Clusia* fruit, showing *Quadriops clusia* crawling on the surface (Suriname: SR17-0322-03A) **C** collecting specimens by submerging rotting fruits in pans of water and waiting for the beetles to float to the surface.

**Terrestrial habitats.** Although rare within Acidocerinae, several genera contain at least one species that has been collected in terrestrial situations. All species of *Quadriops* are known or suspected of being entirely terrestrial (Girón and Short 2017). One species, *Q. clusia* Girón & Short, is reliably found in the rotting fruits of *Clusia* fruits (Fig. 10), while *Q. reticulatus* Hansen has been collected from sap flows in freshly cut trees. Other species are known from passive collecting methods such as FITs but were not found in nearby aquatic habitats. Some species of *Agraphydrus* also appear to be terrestrial, as we have seen series of at least one species from Madagascar from several samples of sifted rainforest litter (Short, pers. obs.). Some other *Agraphydrus* species have ambiguous or incidental collecting information suggesting they may occur in terrestrial habitats, but more data is needed (e.g., *A. vadoni* Komarek). Additionally, *Tobochares fusus* Girón & Short has been collected from both seepage habitats as well as from the rotting fruits of *Clusia*, suggesting it might have a broad ecological niche (Girón and Short 2021a).

**Other unusual habitats.** The blind genus *Troglochares* is only known from a single cave in Ecuador, where it was found clinging to a stalactite. A few species of *Agraphydrus* [e.g., *A. hanseni* (Satô & Yoshitomi)] are associated with the gravel margins of estuarine rivers (Satô and Yoshitomi 2004), however it is not known to what extent they may have any tolerance for salinity.

### Karyotypes of Acidocerinae

A paper summarizing the available information on the karyotypes of water scavenger beetles was recently published by Angus et al. (2020). According to Angus et al. (2020), in Acidocerinae “the diploid number of chromosomes is 2n = 18”. Table 2 presents the list of known acidocerine karyotypes.

**Table 2.** List of acidocerine species with known karyotypes. Origin refers to the country where the adults were collected according to Angus et al. (2020).

**Species**	**Origin**
*Agraphydrus decipiens* Minoshima, Komarek & Ôhara	Taiwan
*Agraphydrus variabilis* Komarek & Hebauer	Taiwan
*Helochares lividus* (Forster)	United Kingdom
*Helochares obscurus* (Müller)	Sweden
*Helochares punctatus* Sharp	United Kingdom
*Helochares sauteri* d’Orchymont	Taiwan

### Larvae of Acidocerinae

From the 541 acidocerine species, immature stages are only known for 18 species in seven different genera to date. Information is summarized in Table 3.

**Table 3.** Summary of information on immature stages of Acidocerinae. Origin refers to the country where the adults, eggs, or larvae were collected according to the provided references.

**Species**	**Origin**	**Described stages**	**References**
*Agraphydrus hanseni* (Satô & Yoshitomi) [as *Horelophopsis hanseni*]	Japan	Third instar larva	Minoshima et al. 2013
*Agraphydrus narusei* (Satô)	Japan	First and third instar larva	Minoshima and Hayashi 2011
*Crephelochares nitescens* (Fauvel) [as *Helochares nitescens* or *Chasmogenus nitescens*]	Australia	Eggs, egg case, first and third instar larvae, pupa	Anderson 1976; Archangelsky 1997
*Helobata larvalis* (Horn)	Guatemala	Egg case, first instar larva	Spangler and Cross 1972; Archangelsky 1997
*Helochares anchoralis* Sharp	Japan	First instar larva	Minoshima and Hayashi 2011
*Helochares clypeatus* (Blackburn)	Australia	Third instar larva	Watts 2002
*Helochares lividus* (Forster) [also *Helochares griseus* (Fabricius)]*	Unknown (Palearctic) – Italy	Unknown stage larva in d’Orchymont 1913b; first, second and third instar larvae in Panzera 1932	d’Orchymont 1913b; Panzera 1932
*Helochares luridus* (MacLeay)	Australia	Third instar larva	Watts 2002
*Helochares maculicollis* Mulsant	USA	Eggs, first and third instar larvae, pupa	Richmond 1920; Archangelsky 1997
*Helochares nipponicus* Hebauer	Japan	First, second and third instar larvae	Minoshima and Hayashi 2011
*Helochares pallens* (MacLeay)	Japan	First, second and third instar larvae	Minoshima and Hayashi 2011
*Helochares tenuistriatus* Régimbart	Australia	Third instar larva	Watts 2002
*Helochares tristis* (MacLeay)	Australia	Eggs, first, second and third instar larvae, pupa	Anderson 1976; Watts 2002
*Novochares pallipes* (Brullé) [as Helochares (s. str.) pallipes]	Argentina	Egg sac, first, second and third instar larvae, pupa	Fernández 1983
*Peltochares conspicuus* Régimbart**	Madagascar	Unknown stage larva	Bertrand 1962
*Peltochares foveicollis* (Montrouzier) [as *Helochares foveicollis*]	Australia	Third instar larva	Watts 2002
*Sindolus femoratus* (Fernández) [as Helochares (Sindolus) femoratus]	Argentina	Egg case, first, second and third instar larvae, pupae	Fernández 2004
*Sindolus talarum* (Fernández) [as Helochares (Sindolus) talarum]	Argentina	Egg case, first, second and third instar larvae, pupae	Fernández 1983

* Panzera (1932) described the larvae of “*Helochares griseus*” (p. 54) and *Helochares lividus* (p. 60); “*Helochares griseus*” is a synonym of *Helochares lividus* (Forster), with some varieties of “*Helochares griseus*” synonymized with *Helochares obscurus* (Müller). This description might correspond to *Helochares lividus* (Forster) or *Helochares obscurus* (Müller). **Peltochares conspicuus has never been reported from Madagascar. The species identification is likely incorrect.

Females lay between 18 (*Crephelochares nitescens* (Fauvel); Anderson 1976) and 103 eggs (*Novochares pallipes* (Brullé) comb. nov.; Fernández 1983) per egg case or nest. In observations from rearing experiments, it has been described that the larvae emerging from egg sacs carried by the females, the larvae seem to emerge towards the mother’s air bubble to capture their own first air bubble (Anderson 1976). For *Crephelochares nitescens*, it was described that the females deposit their eggs in cavities built by the adults in damp soil (Anderson 1976). Larvae of *Sindolus talarum* have been described to perforate and enter the aerenchyma of *Spirodella intermedia* (Araceae) and staying in the plant tissue for some time, apparently breathing the air stored in the plant tissues (Fernández 1983).

### The fossil record of Acidocerinae

Five fossil species have been assigned to Acidocerinae (one of them ambiguously; Table 4). Four of these are compression fossils, one from Australia and three from China. The fifth fossil is a Baltic amber inclusion from Poland, which has been assigned to an extant genus (*Helochares fog* Arriaga-Varela, Brunke, Girón & Fikáček). Despite the diagnostic features presented by Fikáček et al. (2014) on their subfamily designations, the authors highlight that these compression fossils exhibit a generalized morphology in which only specific combinations of character states (as opposed to the presence of synapomorphic features) support those designations. Unlike compression fossils, where there is no realistic way to recover additional information from what is preserved and visible on the rock, amber inclusions have the possibility of offering more details when studied with techniques such as visualization using X-ray micro-computed tomography (μCT, Arriaga-Varela et al. 2019). *Helochares fog* has been used as a calibration point to date the phylogeny of Hydrophilidae (Bloom et al. 2014; Toussaint and Short 2018). One additional fossil, *Cretocrenis burmanicus* Fikáček, Minoshima, Komarek, Short, Huang, & Cai from Burmese amber (ca. 99 ma) has been formally placed in the Anacaenini, although it does have some superficial similarities with Acidocerinae (Fikáček et al. 2017).

**Table 4.** Summary of information on fossil species of Acidocerinae.

**Species**	**Type locality**	**Geological epoch**
*Alegorius yixianus* Fikáček, Prokin, Yan, Yue, Wang, Ren & Beattie, 2014*; Fikáček et al. 2014	China, Liaoning Province, Shangyuan County, Chaomidian Village, Huangbanjigou.	Yixian Formation: Early Cretaceous, Lower Cretaceous, Aptian, 124.6 Mya; Jurassic–Cretaceous boundary, Late Tithonian–Berriasian, ca. 145–140 Mya
*Helochares fog* Arriaga-Varela, Brunke, Girón & Fikáček, 2019; Arriaga-Varela et al. 2019	Poland.	Baltic amber: Lower Eocene to Lower Oligocene, ca. 44 Mya
*Hydroyixia elongata* Fikáček, Prokin, Yan, Yue, Wang, Ren & Beattie, 2014; Fikáček et al. 2014	China, Liaoning Province, Shangyuan County, Chaomidian Village, Huangbanjigou.	Yixian Formation, Early Cretaceous, Lower Cretaceous, Aptian, 124.6 Mya; Jurassic–Cretaceous boundary, Late Tithonian–Berriasian, ca. 145–140 Mya
*Hydroyixia latissima* Fikáček, Prokin, Yan, Yue, Wang, Ren & Beattie, 2014; Fikáček et al. 2014	China, Liaoning Province, Shangyuan County, Chaomidian Village, Huangbanjigou.	Yixian Formation, Early Cretaceous, Lower Cretaceous, Aptian, 124.6 Mya; Jurassic–Cretaceous boundary, Late Tithonian–Berriasian, ca. 145–140 Mya
*Protochares brevipalpis* Fikáček, Prokin, Yan, Yue, Wang, Ren & Beattie, 2014; Fikáček et al. 2014	Australia, New South Wales, Talbragar Fossil Fish Bed, ca. 14 km NNW of Ulan, 25 km NE of Gulgong, 32°9.9'S, 149°41.0'E.	Late Jurassic Oxfordian–Tithonian, 161–145 Mya; Kimmeridgian, 155–150 Mya.

* The genus *Alegorius* has been assigned in doubt to either Acidocerinae or Enochrinae.

### Morphological variation in Acidocerinae and its taxonomic importance

The Acidocerinae have been described as “relatively uniform and difficult to characterize” (Short and Fikáček 2013), mostly because for each proposed synapomorphy, there are taxa that exhibit exceptional character states. The phylogeny presented by Short et al. (2021) revealed a high recurrence of morphological convergence across the phylogeny of the Acidocerinae that seem to track ecologies rather than phylogenetic relationships. Here we present an account of morphological features, how they vary in the subfamily, and their usefulness for recognizing taxonomic units. A summary of the main diagnostic features of each genus is presented in Table 5 at the end of ths section.

**Table 5.** Summary of main diagnostic features of acidocerine genera.

Genus	Size	Antennomeres	Sutural Stria	Serial punctures or striae	5^th^ Ventrite	Metafemora
*** Acidocerus ***	2.8 mm	9	Absent	Present	Emarginated	Mostly pubescent
*** Agraphydrus ***	1.4-4.8 mm	8 or 9	Absent	Variable	Variable	Variable
*** Aulonochares ***	5.8-7.5 mm	9	Absent	Absent	Emarginated	Mostly pubescent
*** Batochares ***	3-4 mm	9	Absent	Present	Truncate	Mostly pubescent
*** Chasmogenus ***	2.5-5.0 mm	8	Present	Absent	Emarginated (weak)	Mostly Pubescent
*** Colossochares ***	8.5-14.0 mm	9	Absent	Absent	Emarginated	Mostly pubescent
*** Crephelochares ***	2.5-4.8 mm	9	Present	Absent	Emarginated (weak)	Mostly pubescent
*** Crucisternum ***	2.0-2.5 mm	9	Absent	Absent	Rounded	Mostly pubescent
*** Ephydrolithus ***	1.8-3.3 mm	9	Absent	Variable	Truncate	Mostly glabrous
*** Globulosis ***	1.9-2.3 mm	8	Absent	Absent	Emarginated	Mostly pubescent
*** Helobata ***	4-7 mm	8	Absent	Variable	Emarginated	Mostly pubescent
*** Helochares ***	2-7 mm	9	Absent	Variable	Emarginated	Mostly pubescent
*** Helopeltarium ***	3.5 mm	9	Absent	Absent	Emarginated	Mostly pubescent
*** Katasophistes ***	2.7-4.5 mm	9	Absent	Absent	Emarginated (weak)	Mostly pubescent
*** Nanosaphes ***	1.1-1.5 mm	8	Absent	Absent	Emarginated	Mostly pubescent
*** Novochares ***	4.5-9.0 mm	9	Absent	Variable	Emarginated	Mostly pubescent
*** Peltochares ***	6-14 mm	9	Absent	Variable	Emarginated	Mostly pubescent
*** Primocerus ***	2.4-4.9 mm	8	Present	Variable	Variable	Variable
*** Quadriops ***	1.6-2.6 mm	9	Absent*	Variable	Rounded	Mostly glabrous
*** Radicitus ***	4.5-6.2 mm	9	Absent	Variable	Rounded	Pubescent on anterior third
*** Sindolus ***	2.5-5.0 mm	9	Absent	Absent	Emarginated	Mostly pubescent
*** Tobochares ***	1.5-2.6 mm	8	Absent*	Variable	Rounded	Mostly glabrous
*** Troglochares ***	1.9 mm	9	Absent	Absent	Rounded	Pubescent (~half)*

* When impressed, the stria I on each elytron can be comparatively more strongly impressed, specially along the posterior half of the elytron, which might resemble a well-developed sutural stria.

**Size and shape of body.** This subfamily includes members among the largest (14.0 mm) and smallest (1.1 mm) hydrophilids (Fig. 1). In general terms, acidocerines can very roughly be grouped by their size: most genera in the *Helochares* group (sensu Short et al. 2021) are larger than 4 mm (Fig. 1), whereas *Agraphydrus*, *Chasmogenus*, *Crephelochares*, *Primocerus*, and members of the *Tobochares* group are smaller than 4.5 mm (Fig. 1). The body is usually oval and parallel-sided, occasionally slightly broader anteriorly or posteriorly; it can also be rather dorsoventrally flattened [e.g., *Helobata* (Fig. 1J), *Peltochares* (Fig. 1C), *Helopeltarium* (Fig. 1H)], or strongly convex [e.g., *Globulosis* (Fig. 1U), *Colossochares* (Fig. 1A), *Radicitus* (Fig. 1K)], but it is generally moderately convex. The outline of the body in dorsal view is continuous (not interrupted between pronotum and elytra) when the specimens are in natural resting position; when a specimen is card-mounted the outline of the body may appear interrupted.

**Coloration.** Body color ranges from very pale (yellowish) to very dark brown (appearing almost black), and it is usually uniform along the dorsal surfaces of the body, although sometimes the margins of the pronotum and elytra may be slightly paler than the disc (Fig. 1). The ventral surface of the body and the appendages (or parts of appendages) tend to be paler than the dorsum. In *Batochares* (e.g., Fig. 1I) and *Helobata* (e.g., Fig. 1J), there are alternating areas of darker/paler colorations along the elytra, giving specimens a flecked or speckled appearance. In some species of *Nanosaphes*, different regions of the body (head, pronotum, elytra) have different colorations (e.g., Fig. 1L); in some species of *Tobochares*, the lateral margins of the clypeus are paler (e.g., Fig. 1N); in both cases, coloration can be used for species group recognition. The coloration of the maxillary palps can also be helpful in diagnosing species (e.g., in *Tobochares* and *Helochares*), as the apex, or rarely the entire palp can be darkened. In some genera, internal structural reticulations are visible throughout the surface (mostly on the elytra), giving the beetles a “checkered” appearance of darker spots over a paler background, e.g., *Aulonochares* (Fig. 1D), New World *Helochares* (Fig. 36A, B; Short and Girón 2018).

**Punctation.** Three kinds of punctures can be recognized along the dorsal surface of the body in Acidocerinae that may be shallowly to moderately or sharply (strongly) marked. Ground punctures are usually fine and uniformly distributed along the entire body. Systematic punctures (sensu Hansen 1991), those bearing a seta inserted in a doughnut-shaped socket (thrichobothria sensu Short and Fikáček 2013; Fig. 13A–C, red arrows), are usually well developed and can also be found along the entire body, being more densely distributed in particular areas of the head, pronotum and elytra. The seta on a systematic puncture is usually fine and can be short or long; sometimes these setae may be lost by abrasion but are usually visible along the lateral and posterior areas of the elytra. Systematic punctures usually form well defined rows along the elytra; quite a few species in some genera exhibit four or five rows of systematic punctures clearly enlarged in comparison with the remainder elytral punctation, e.g., *Agraphydrus* (Fig. 1M, S, T), *Ephydrolithus* (Fig. 31), *Katasophistes* (Fig. 39). Serial punctures are only present along the elytra and can only be recognized when well-developed (larger and usually more impressed than ground punctures), as they form usually ten well-defined rows, at least along the posterior third of each elytron (e.g., *Radicitus*, Fig. 50A, B); some *Agraphydrus* species have strongly enlarged and irregular elytral series of punctures (e.g., Fig. 18D–F). Serial punctures were traditionally used for the recognition of subgenera within *Helochares* sensu Hansen (1999b), but it has been shown that the presence or absence of this kind of punctures has taxonomic value only at the species or species group level in certain genera (e.g., *Primocerus*, Fig. 46; *Tobochares*, Fig. 52–54). The presence, size, density, degree of impression and development/differentiation of punctures on the dorsal surface of the body are useful for recognition of certain genera and species, but there are no general character states that cover the entire subfamily.

**Figure 11.**
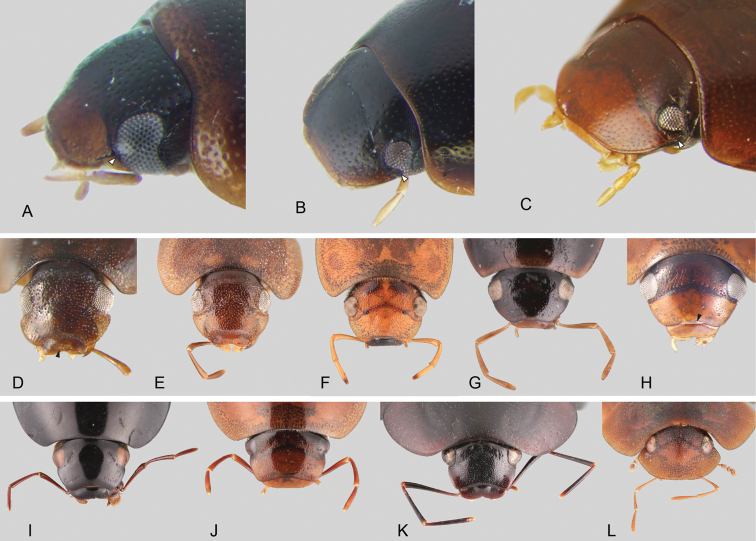
Head of miscellaneous Acidocerinae**A–D** anterolateral view: **A***Tobochares luteomargo* with white arrow pointing to straight anterior margin of eye **B***Tobochares emarginatus* with white arrow pointing to canthus emarginating anterior margin of eye **C***Quadriops politus* with white arrow pointing to canthus fully dividing the eye in dorsal and ventral faces **D***Batochares* sp. black arrow pointing to transverse carina on labrum **E–L** dorsal view of head: **E***Batochares* sp. **F***Helochares tristis***G***Crephelochares nitescens*, **H***Chasmogenus australis* with black arrow pointing to preclypeal membrane **I***Colossochares ellipticus***J***Aulonochares tubulus***K***Peltochares conspicuus***L***Helobata larvalis*.

**Eyes.** The only known species of hydrophilid lacking eyes (*Troglochares ashmolei* Spangler, Fig. 56) is a member of the Acidocerinae. Eyes range in shape from subquadrate to oval and are usually of moderate size (Fig. 11E–L), although in some species the eyes are relatively small (e.g., *Primocerus ocellatus* Girón & Short, *Tobochares microps* Girón & Short). In some genera, the anterior corners of the frons extend posteriorly forming a canthus that emarginates the anterior margin of the eyes (Fig. 11B), which is more evident in lateral view (e.g., *Tobochares*, Fig. 11B; *Helobata*, Fig. 11L). There is only one known acidocerine genus in which the canthus reaches the posterior margin of the eye, thus completely dividing the eye in dorsal and ventral faces (*Quadriops*; Fig. 11C). In some genera the eyes are protruding, interrupting the outline of the head (e.g., *Aulonochares*; Fig. 11J). In most cases the proportion between the width of an eye and the distance between eyes remains constant across congeneric species. The shape, size, and degree of protrusion of the eyes are useful for generic recognition.

**Clypeus.** It is usually roughly trapezoid (clearly wider at base; Fig. 11F–I) and relatively flat or antero-medially convex. In some genera, it fully conceals the labrum (e.g., *Helobata*, Fig. 11L; *Helopeltarium*, Fig. 38A). The shape of the anterior margin of the clypeus, and the development of a membranous preclypeal area (Fig. 11H) are useful for diagnosing species within some genera (e.g., *Chasmogenus*). In some *Helochares* the surfaces along the lateral margins of the clypeus are slightly bent upwards.

**Figure 12.**
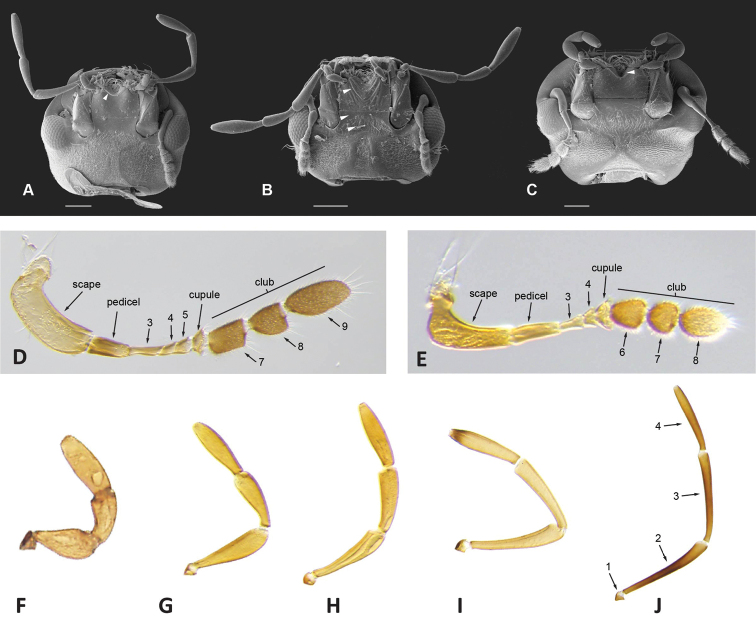
Head structures **A–C** scanning electron micrographs of ventral view of head: **A***Tobochares pallidus* with smooth mentum and white arrow pointing to transverse carina limiting posterior margin of antero-medial depression **B***Nanosaphes tricolor* with top white arrow pointing to oblique crenulations of mentum, mid white arrow pointing to flat and smooth anterior surface of submentum, and bottom white arrow pointing to concave posterior surface of submentum **C***Quadriops reticulatus* with white arrow pointing to antero-medial depression of mentum **D, E** light micrographs of antenna: **D***Aulonochares tubulus* (9 antennomeres) **E***Chasmogenus cremnobates* (8 antennomeres) **F–J** light micrographs of maxillary palps: **F***Quadriops reticulatus***G***Agraphydrus insidiator***H***Helochares* sp. **I***Helochares lividus***J***Aulonochares tubulus*. Scale bars: 100 μm (**A–C**)

**Maxillary palps.** In general, the maxillary palps in Acidocerinae have been described as ‘curved inward’ (e.g., Hansen 1991), which means that the outer margin of the maxillary palpomere 2 is apically or medially curved towards the midline of the body, and the apex of palpomere 2 is oblique, so that the palpomere 3 articulates pointing towards the midline of the body. The inner margin of maxillary palpomere 2 ranges from straight (Figs 12F, G) to slightly and uniformly curved (concave; Figs 12H–J). All palpomeres tend to be of somewhat similar proportions among them, and are usually similar in length as well, although it is common that the maxillary palpomere 2 is slightly longer. The comparative length of maxillary palpomeres 3 and 4 may be useful as a supporting diagnostic feature. According to the diagnosis of the Acidocerinae offered by Hansen (1991) and by Short and Fikáček (2013), the maxillary palps are at least as long or usually longer than the width of the head (except for some *Agraphydrus* and *Quadriops*). The number of exceptions to this rule keeps growing, the more seepage taxa are found (e.g., *Ephydrolithus*, *Radicitus*, some *Tobochares*). The length of the maxillary palpomeres in Acidocerinae ranges from very short and stout (nearly half width of the head; e.g., *Quadriops*, Figs 11C, 12G), to very long and slender (nearly 2 × width of the head; e.g., *Peltochares conspicuus*, Fig. 11K).

**Mentum.** The anterior margin of the mentum is usually laterally emarginated by the base of the palpigers, mesally emarginated, and deeply depressed in ventral view (projected upwards) (Fig. 12A–C); this antero-medial depression varies in width and depth and may be demarcated by a transverse crest or carina (Fig. 12A). The surface of the mentum may be flat, medially depressed or bear oblique elevations (Fig. 12B); the surface may further range from smooth (Fig. 12A) to punctate, to anteriorly striate, with little or no variation within genera. Characteristics of the mentum and submentum may be useful as supporting diagnostic features.

**Antennae.** The number of antennomeres is either nine (the ancestral state in Hydrophilidae; Hansen 1991; Fig. 12D) or reduced to eight (Fig. 12E). The cupule (the antennomere right before the club) can be symmetric, or slightly to strongly asymmetric. The three-part pubescent antennal club is always loosely articulated in Acidocerinae; the proportions of the club antennomeres have been used in the past to recognize some groups.

**Figure 13.**
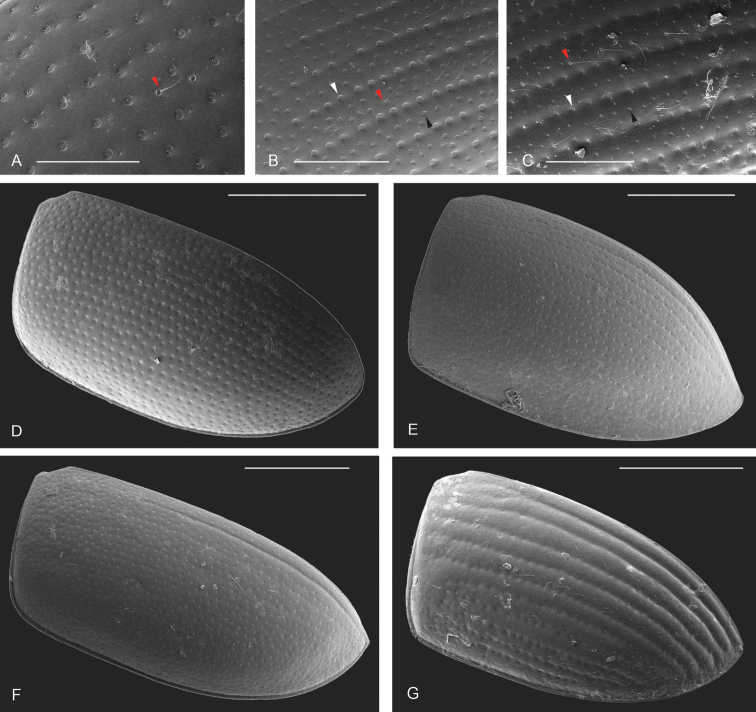
Elytral punctation **A***Tobochares communis* with red arrow pointing to systematic puncture **B***Tobochares sipaliwini* with red arrow pointing to systematic puncture, white arrow pointing to serial puncture, and black arrow pointing to ground/interserial puncture **C***Tobochares striatus* with red arrow pointing to systematic puncture, white arrow pointing to serial puncture, and black arrow pointing to ground/interserial puncture **D***Tobochares communis* elytron with all kinds of punctures similar in size and degree of impression, seemingly evenly distributed (to longitudinally aligned) **E***Quadriops similaris* with serial punctures longitudinally aligned **F***Primocerus maipure* with sutural stria **G***Tobochares striatus* with impressed serial striae. Scale bars: 100 μm (**A**); 200 μm (**B, C**); 500 μm (**D–G**).

**Figure 14.**
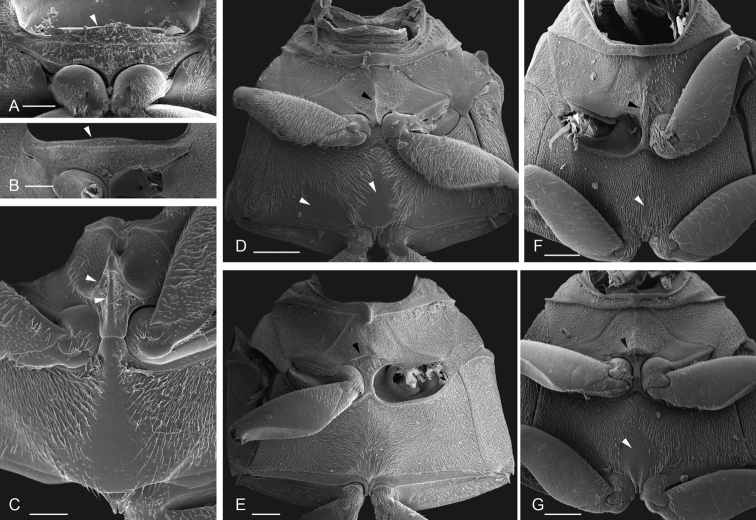
Scanning electron micrographs of thorax in ventral view **A, B** prosternum: **A***Tobochares striatus* with white arrow pointing to anterior projection **B***Quadriops reticulatus* with white arrow pointing to anterior projection **C–G** mesoventrite and metaventrite: **C***Crucisternum ouboteri* with white arrows pointing to anteriorly pointed transverse ridge and longitudinal carina of mesoventrite, metaventrite with median glabrous patch **D***Nanosaphes tricolor* with black arrow pointing to longitudinal carina along mesoventrite and white arrows pointing to median and postero-lateral glabrous patches of metaventrite **E***Quadriops reticulatus* with black arrow pointing to transverse carina across mesoventrite and metaventrite uniformly pubescent **F***Tobochares communis* with black arrow pointing to longitudinal carina along mesoventrite and white arrow pointing to narrow postero-medial glabrous patch on metaventrite **G***Tobochares kasikasima* with black arrow pointing to transverse elevation across mesoventrite and white arrow pointing to broad postero-medial glabrous patch on metaventrite. Scale bars: 100 μm.

**Thoracic venter.** The prosternum in Acidocerinae is usually rather flat (Fig. 14A, B), at most medially tectiform or broadly bulging, except in *Acidocerus* and *Crucisternum* which bear a medial longitudinal carina. The surface of the posterior elevation of the mesoventrite is taxonomically important; it may be projected in various forms: as a longitudinal carina (Fig. 14D, F), cruciform projection (Fig. 14C), transverse ridge (Fig. 14E, G) or acute spine. The shape of the projection on the posterior elevation of the mesoventrite can sometimes be used for recognition of genera, but it may also vary among congeneric species (e.g., *Ephydrolithus*, *Nanosaphes*). The shape of the anapleural sutures ranges from angulate (forming an obtuse angle; e.g., *Primocerus*, *Troglochares* (Spangler 1981a: fig. 8) to only slightly curved (e.g., *Katasophistes*, *Nanosaphes* (Girón and Short 2018: figs 11A, 17A, respectively); the orientation along their anterior section may be nearly parallel (e.g., *Helobata*; Clarkson et al. 2016: fig. 8) or anteriorly converging; they may be widely separated anteriorly (anterior margin of mesoventrite nearly as wide as anterior margin of mesepisternum; e.g., *Globulosis*, *Nanosaphes* (Girón and Short 2018: fig. 17A), or very closely converging (anterior margin of mesoventrite 0.2 × the width of the anterior margin of mesepisternum; e.g., *Ephydrolithus* (Girón and Short 2019: fig. 7A), *Katasophistes* (Girón and Short 2018: fig. 11A). The metaventrite is usually densely and uniformly covered by hydrofuge pubescence; a posteromedian glabrous patch and/or posterolateral glabrous patches may also be present (Fig. 14C–G). The size and shape of the posteromedian glabrous patch is useful for recognition of some genera and subgenera (e.g., *Tobochares*).

**Elytra.** The shape and punctation of the elytra are highly variable in the Acidocerinae. The elytra may be evenly convex (e.g., *Radicitus*, Fig. 1K) or with nearly flat dorsal outline (e.g., *Helopeltarium*, Fig. 1H), with outer margins slightly flared or broadly explanate (e.g., *Helobata*, Fig. 1J); the surface is usually smooth, but can also be granulate (e.g., *Acidocerus*, Fig. 17; *Helobata*, Fig. 33). Sutural striae are only present in *Chasmogenus* (Fig. 24), *Crephelochares* (Fig. 28), and *Primocerus* (Figs 13F, 46). The elytral punctation has been traditionally considered as a diagnostic feature at the subgenus level, in *Helochares* for example, but it is clear now that this character system can be variable among congeneric species (e.g., *Ephydrolithus*, Fig. 31; *Katasophistes*, Fig. 39; and *Primocerus*, Fig. 46). In some cases, all kinds of punctures (ground punctures, systematic punctures, and serial punctures) are well-developed and therefore easily recognized (e.g., Fig. 13B, C), but in other instances they can be virtually indistinguishable from each other (e.g., Fig. 13A, D, F). In some species, or even groups of species within a genus, the serial punctures are impressed forming longitudinal grooves that can extend from the anterior to the posterior margins of the elytra (e.g., Fig. 13G; *Tobochares sulcatus* Short & García, Fig. 52A, B), or at least along the posterior third of each elytron (e.g., *Tobochares akoerio*, Fig. 54C, D). When serial punctures are well developed, the ground punctures between series have been called “interserial punctures” (Fig. 13B [black arrows], C [black arrows], G; Girón and Short 2021a), and their distribution may be informative at the species level.

**Figure 15.**
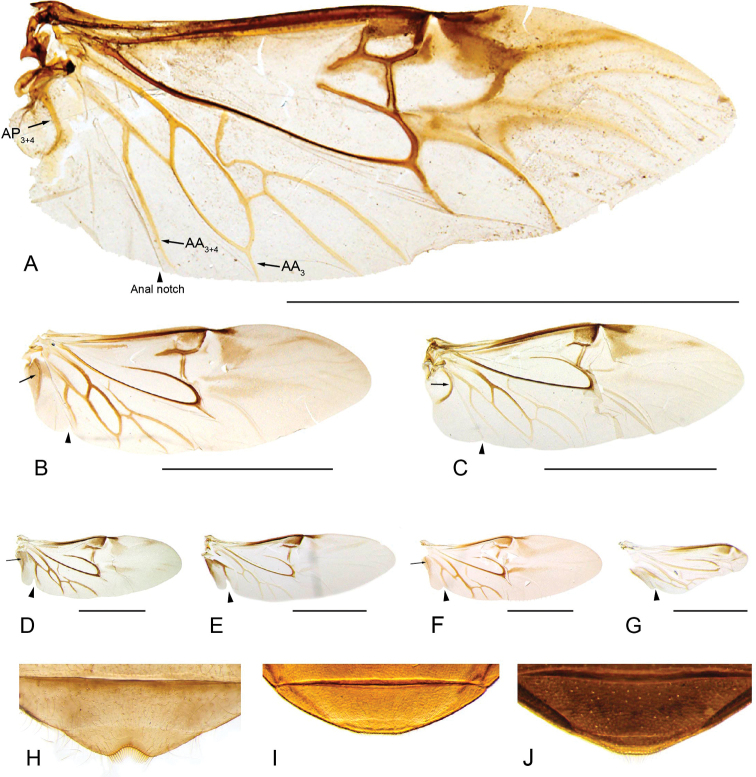
Hind wing and abdominal ventrite 5 **A–G** hind wings: **A***Colossochares ellipticus***B***Primocerus gigas***C***Helobata larvalis***D***Crucisternum ouboteri***E***Tobochares sipaliwini***F***Quadriops similaris***G***Tobochares microps***H–J** abdominal ventrite 5: **H***Aulonochares tubulus***I***Primocerus neutrum***J***Ephydrolithus hamadae*. Scale bars: 1 cm (**A**); 3 mm (**B, C**); 1 mm (**D–F**); 0.5 mm (**G**).

**Hind wings.** The hind wings of the Acidocerinae are usually well developed, with most of the general venation clearly visible. The posterior margin of the wing usually has a well-defined anal notch, demarcating a noticeable “jugal lobe” (Hansen 1991: fig. 285) that is either broad (Fig. 15B, C) or narrow (Fig. 15D–G). AP3+4 can be either thick and curved (Fig. 15A, C), or evanescent and angulate (Fig. 15B, D–G). *Tobochares microps* Girón & Short was found to be polymorphic for hind wing development: the reduced hind wing morph (Fig. 15G) has most veins still well developed, but the entire apical region of the wing is reduced (Girón and Short 2021a).

**Protibiae.** Two main features of the protibia are taxonomically relevant: the shape and size of the apical spurs and the characteristics of the spines composing the median longitudinal anterior row. The apical spurs are usually large and slender (longer than protarsomere 1) but can be relatively short and stout (as long as or shorter than protarsomere 1; e.g., *Aulonochares*). The spines composing the median longitudinal anterior row can be very short, stout, and appressed to the surface of the tibia in most members of the *Helochares* group (sensu Short et al. 2021), or be long, relatively thick, seta-like, and semi-erect (e.g., *Tobochares* group).

**Metafemora.** In Acidocerinae the metafemora are moderate to strongly antero-posteriorly compressed. The anterior surface of the metafemur may be covered to a variable degree with hydrofuge pubescence. Usually, species found in typical fully aquatic habitats (streams, ponds, marshes) have the anterior surface of the metafemora mostly covered by pubescence (e.g., Figs 21C, 26C, 32C, 36C), whereas species found in hygropetric habitats (seepages) exhibit a reduced coverage (about half the surface or less, e.g., Figs 39C, 46I, 50F) and fully terrestrial species (on rotten fruits) lack any pubescence (i.e., *Quadriops*, Fig. 48C, F). The degree of coverage may be useful for generic identifications in many cases, and it is also known to vary among species of *Agraphydrus* and *Primocerus*. The degree of development of the tibial grooves (ventral surface that is either flat or concave) of the metafemora can also be used as a supporting character for identifications; they may be well developed, when at least the posterior edge is sharply marked, or reduced, or absent when the ventral surface of the metafemur is convex or only relatively flattened, without any sharp edges.

**Tarsi.** The tarsal formula of acidocerine beetles is always 5-5-5, with tarsomeres 1–4 usually similar in shape and length and tarsomere 5 longer and slender; tarsomere 2 is the most variable in length, ranging from similar to tarsomere 1 to as long as tarsomere 5. The coverage of the ventral surface of the tarsomeres is variable. Usually, the protarsomeres will have a dense and uniform coverage of thick setae; the coverage of meso- and metatarsomeres 1 may be asymmetric, with thick setae only along its outer margin. Tarsomeres 2, 3 and 4 may be densely covered ventrally, but more frequently bear a pair of lateral rows of denticles, spines or spiniform setae. Tarsomeres 5 are usually glabrous ventrally, rarely bear a ventral medial row of tiny denticles or fine setae. Very fine and relatively long natatorial setae (swimming hairs sensu Hansen 1991) may be present on the dorsal face of meso- and metatarsomeres but are scarce and do not form a fringe. The length of metatarsomeres 5 relative to the length of all or some of the remaining tarsomeres may be useful as a supporting character to recognize genera.

**Apical margin of fifth abdominal ventrite**. The apical margin of the fifth abdominal ventrite usually bears a mesal emargination that varies in depth and is usually fringed by flat and stout setae (Fig. 15H). There is a trend for taxa from seepages or terrestrial habitats to have a rounded or truncate posterior margin of the fifth abdominal ventrite (Fig. 15I, J); in these cases, the flat and stout setae are reduced or absent.

**Figure 16.**
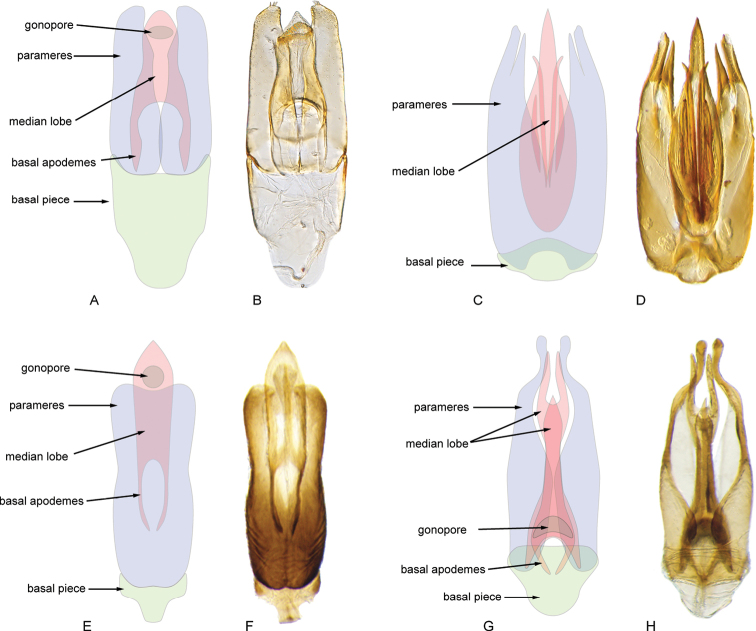
Aedeagi **A–E** trilobed: **A** schematic **B***Chasmogenus schmits***C, D** spiked: **C** schematic **D***Peltochares foveicollis***E, F** tubular: **E** schematic **F***Helochares politus***G, H** divided: **G** schematic **H***Novochares pallipes*.

**Aedeagus.** The general configuration of the aedeagus in acidocerines is highly variable across the subfamily (Fig. 16), yet (usually) strongly conserved within genera and even groups of genera. An attempt to group African species of Helochares (Hydrobaticus) by aedeagal categories was made by Hebauer (1996).

For merely practical purposes, here we propose four main aedeagal forms in Acidocerinae. These categories are very general and by no means exhaustive or detailed but encompass some of the broad variations we have found. We do not use these categories to convey any phylogenetic meaning, although certainly there is likely very strong phylogenetic signal within the aedeagal morphology of the subfamily.

1. trilobed aedeagus (Fig. 16A, B): parameres separated from each other for most of their lengths; parameres and median lobe simple (without subdivisions); basal piece of variable length; gonopore usually well differentiated, variable in positioning along median lobe. With the exception of the Helochares group, this is the dominant type of aedeagus within the subfamily. All species of the Primocerus (Fig. 47), Tobochares (Figs 30, 40, 49A–D, 55) and Agraphydrus groups (Fig. 20) share this aedeagal form. Batochares (Fig. 22D) and part of Chasmogenus (Fig. 25A–C) do as well.
2. spiked aedeagus (Fig. 16C, D): main component of median lobe strongly sclerotized, distally elongated and apically acute, usually accompanied by additional shorter slender sclerotizations (these may or may not be symmetrical); apical region of parameres usually partly heavily sclerotized and partly membranous, often bifurcated; basal piece strongly reduced; gonopore usually not clearly visible; e.g., Peltochares (Fig. 45).
3. tubular aedeagus (Fig. 16E, F): parameres fused to each other for most of their lengths, forming a tubular structure with apex either simple or bifurcate/bilobate; median lobe with long to very long basal apodemes (as long or longer than main component of median lobe); median lobe either simple (without subdivisions), or with different kinds of sclerotizations of inner membranes; basal piece usually much shorter than parameres; gonopore of variable development; e.g., Aulonochares (Fig. 22A–C), Helochares (Fig. 37).
4. divided aedeagus (Fig. 16G, H): parameres usually separated from each other for most of their lengths; median lobe divided in dorsal and ventral plates; dorsal and ventral plates may be further bilaterally subdivided, or otherwise shaped; basal piece shorter than parameres, always noticeable; gonopore usually clearly visible, variable in positioning along median lobe. This form is apparent in Helobata (Fig. 34), Novochares (Fig. 43), and Sindolus (Fig. 49E, F).

Some of these aedeagal categories are further modified in an incredible array of shapes (e.g., Figs 37, 43), and clearly deserve detailed morphological and functional studies. The particular configuration and relative proportions of parts is, for the most part, genus specific. Even though the median lobe is divided in *Crephelochares* (Fig. 27B–D), the aedeagal form does not quite conform to any of the described above. Though most genera include species with only one of the forms given above, a few are known to include diverse forms: for example, the genus *Radicitus* includes forms that are relatively simple and trilobed (Fig. 49I–L) as well as those that are greatly modified, with divided and hooked parameres (Fig. 49G, H). Likewise, the vast majority of *Chasmogenus* species share a simple trilobed form (e.g., Fig. 25A–C), but a few recently described species exhibit a bizarre and unique aedeagal configuration in which both the parameres and median lobe are enlarged and asymmetrical (Fig. 25D, E).

### Key to genera of Acidocerinae of the World

**Table:** 

1	Distributed in the Old World	**2**
–	Distributed in the New World	**9**
2	Labrum concealed by clypeus. Only known from the Indo-Malayan region	***Helopeltarium* (Figs 1H, 37I, 38)**
–	Labrum not concealed by clypeus	**3**
3	Elytra with distinctly impressed sutural striae (Fig. 1V)	***Crephelochares* (Figs 1V, 11G, 27B–D, 28)**
–	Elytra without sutural striae	**4**
4	Labrum with apical region anteriorly flattened, thus bearing a fine transverse carina across anterior margin (Fig. 11D, E); pronotum antero-laterally explanate and bent upwards (marginal areas concave; Fig. 23A, B); elytra with margins explanate, especially along anterior third (Fig. 23A); body smaller than 5 mm; basal piece of aedeagus nearly 1.5 × longer than parameres (Fig. 22D). Only known from the Afrotropical region	***Batochares* (Figs 1I, 22D, 23)**
–	Labrum with apical region not anteriorly flattened, with even surface (without transverse carina, e.g., Fig. 11H, K); pronotum evenly convex, not laterally explanate (e.g., Fig. 1A, G); elytra with margins not explanate, at most flared (e.g., Fig. 1A, G); if elytra with margins explanate, body approximately 10 mm (e.g., Fig. 1C); basal piece of aedeagus variable in length, usually less than 0.5 × length of parameres (e.g., Fig. 16C–F). Afrotropical or elsewhere in the Old World	**5**
5	Head and pronotum with granulate surface (Fig. 17); body size small (ca. 3 mm); prosternum with median carina; elytra narrowly explanate laterally, with ten well defined rows of coarse serial punctures impressed into striae (Fig. 17A). Only known from the Afrotropical region	***Acidocerus* (Fig. 17)**
–	Head and pronotum shallowly to moderately punctate, without granulations (e.g., Fig. 1A, E, F); body size variable (2–14 mm); prosternum flat to medially broadly bulging, without median carina; elytra at most flared, with or without impressed serial punctures (e.g., Fig. 1A, E, F). Afrotropical or elsewhere in the Old World	**6**
6	Body length 8.5–14.0 mm; body shape broadly oval in dorsal view, strongly and uniformly convex in lateral view (Fig. 1A); ground punctation extremely fine and shallow; coloration uniformly dark brown (nearly black). Only known from the Afrotropical region	***Colossochares* gen. nov. (Figs 26, 27A)**
–	Body length 1.4–14.0 mm; body shape broadly oval in dorsal view, weakly to moderately convex in lateral view (Fig. 1B, C, E, F); ground punctation from fine and shallow to moderately marked; coloration variable, ranging from yellow to dark brown. Widespread in the Old World	**7**
7	Body length 1.4–4.8 mm; inner margin of maxillary palpomere 2 straight to nearly straight (Fig. 12G); metaventrite with posteromedian glabrous patch (e.g., Figs 18C, F, I); posterolateral glabrous patches absent; antennae with eight or nine antennomeres	***Agraphydrus* (Figs 1M, S, T, 18–20)**
–	Body length 2–10 mm; inner margin of maxillary palpomere 2 weakly and evenly curved (e.g., Fig. 12H, I), seldom nearly straight; metaventrite without posteromedian glabrous patch (e.g., Figs 35C, F, 36C, F); posterolateral glabrous patches may be present; antennae with nine antennomeres (Fig. 12D)	**8**
8	Body length 2–7 mm; dorsal coloration yellow to medium brown (Figs 35, 36); posterior elevation of mesoventrite flat to simply bulging; tibial grooves absent to weakly developed; aedeagus tubular (Figs 16E, F, 37A–H)	***Helochares* (in part; Figs 1E, F, 35–37)**
–	Body length 6–14 mm; dorsal coloration dark brown to black (Fig. 44); posterior elevation of mesoventrite longitudinally elevated; tibial grooves sharply marked; aedeagus spiked (Fig. 16C, D)	***Peltochares* (Figs 1B, C, 44, 45)**
9	Eyes absent. Known only from a cave in Ecuador	***Troglochares* (Fig. 56)**
–	Eyes present	**10**
10	Eyes completely divided into dorsal and ventral sections by a lateral projection of frons (Fig. 11C). Size small (<3 mm). Ranging from Costa Rica to northern South America	***Quadriops* (Figs 1P, 48, 49A–D)**
–	Eyes not divided into dorsal and ventral sections by frons (e.g., Fig. 11A, B). Size variable. Anywhere in the New World	**11**
11	Labrum concealed by clypeus (Fig. 11L), elytral margins broadly explanate (Fig. 33A, D–F). Body extremely dorsoventrally compressed (Fig. 33B)	***Helobata* (Figs 1J, 11L, 33, 34)**
–	Labrum not concealed by clypeus (e.g., Fig. 11H, J), elytral margins not or at most weakly explanate (e.g., Fig. 1N–R). Body form variable but rarely dorsoventrally compressed (e.g., Fig. 1N–R)	**12**
12	Elytra with distinctly impressed sutural striae (e.g., Fig. 1R). Only Neotropical region	**13**
–	Elytra without sutural striae (e.g., Figs 1N–Q, U). Both Neotropical and Nearctic	**14**
13	Posterior elevation of the mesoventrite either flat, broadly elevated or with a longitudinal elevation. Gonopore present and distinct (Fig. 24)	***Chasmogenus* (Figs 24, 25)**
–	Posterior elevation of the mesoventrite with a transverse curved ridge, either sharp or reduced, or with a sharp, pyramidal (triangular) spine-like projection. Gonopore absent (Fig. 47)	***Primocerus* (Figs 1R, 46, 47)**
14	Prosternum with strongly elevated median carina (Fig. 29C)	***Crucisternum* (Figs 29, 30A–E)**
–	Prosternum not or only very slightly carinate or at most tectiform medially (e.g., Fig. 14 A, B)	**15**
15	Posterior elevation of mesoventrite with a large, sharp and strongly elevated laminar longitudinal carina (Fig. 51C); body in lateral view evenly and moderately convex (Fig. 51B)	***Sindolus* (Figs 49E–F, 51)**
–	Posterior elevation of mesoventrite variable, but never with a large, sharp and strongly elevated laminar longitudinal carina; body in lateral view variable (Fig. 1L, N, O)	**16**
16	Elytral systematic punctures very distinct, distinctly larger than surrounding ground punctation, forming five longitudinal rows along each elytron (Figs 31, 39). Antennae with nine antennomeres (Fig. 12D)	**17**
–	Elytral systematic punctures indistinct, usually blending with surrounding ground punctation (e.g., Figs 32, 41, 52). Antennae with eight or nine antennomeres (Fig. 12E)	**18**
17	Metafemora mostly glabrous, with only few scattered setae on anterior surface (Fig. 31C, F). Found in the highlands of eastern Brazil	***Ephydrolithus* (Figs 30F–I, 31)**
–	Metafemora at most glabrous along apical third (Fig. 39C, F). Recorded from the Andean region	***Katasophistes* (Figs 39, 40A–D)**
18	Antennae with eight antennomeres (Fig. 12E). Size small (< 3 mm)	**19**
–	Antennae with nine antennomeres (Fig. 12D). Size variable but usually > 4 mm	**21**
19	Anterior surfaces of metafemora mostly glabrous, with scattered setae (e.g., Fig. 52C, F)	***Tobochares* (Figs 1N, O, 52–55)**
–	Anterior surfaces of metafemora densely covered by hydrofuge pubescence along basal 3/4 (e.g., Figs 32C, 41C, F)	**20**
20	Body form circular, rounded (Fig. 32A). Size very small (1.9–2.3 mm)	***Globulosis* (Figs 30J, 32)**
–	Body form ovoid, parallel sided (Fig. 41A, D). Size exceedingly small (1.1–1.5 mm)	***Nanosaphes* (Figs 1L, 40E–H, 41)**
21	Fifth ventrite entire, without apical emargination or truncation. Maxillary palps shorter than the width of the head	***Radicitus* (Figs 1K, 49G–L, 50)**
–	Fifth ventrite with apical emargination. Maxillary palps as long or longer than the width of the head	**22**
22	Head subquadrate (Fig. 11J); eyes relatively small, separated by a distance nearly 6.5 × the maximum width of an eye; mentum and submentum roughly punctate; pubescence covering abdominal ventrites composed of long golden setae; ventral surface of metatarsomeres 1–4 densely setose. Northern Amazon region	***Aulonochares* (Figs 1D, 21, 22A–C)**
–	Head trapezoid; eyes moderate in size, separated by a distance nearly 4 × the maximum width of an eye; mentum obliquely striate, submentum smooth to shallowly punctate; pubescence covering abdominal ventrites composed of short setae; ventral surface of metatarsomeres 1–4 only with paired rows of denticles	**23**
23	Body size 4.2–7.0 mm; maxillary palps nearly as long as maximum width of the head; internal structural reticulations usually visible along entire dorsal surface of elytra (Fig. 36A, B); metaventrite uniformly covered by hydrofuge pubescence (Fig. 36C); tibial grooves absent to weakly developed; aedeagus tubular (e.g., Fig. 37G). Ranging from USA to Venezuela and Peru (Andean region)	***Helochares* (in part; Figs 36A–C, 37G)**
–	Body size 4.5–9.0 mm; maxillary palps 1.1–1.5 × the maximum width of the head; internal structural reticulations of elytra absent (Fig. 42); metaventrite with median glabrous patch, sometimes very narrow and extending along entire length of metaventrite (Fig. 42C, F); tibial grooves well-developed, with sharp margins; aedeagus divided (e.g., Fig. 16G, H)	***Novochares* gen. nov. (Figs 1G, 42, 43)**

### Taxonomy

#### Subfamily Acidocerinae

Taxon classificationAnimaliaColeopteraHydrophilidae

Zaitzev, 1908

Acidocerini Zaitzev, 1908: 353, as subfamily.
Acidocerina as subtribe Acidocerina [of tribe Hydrophilini, subfamily Hydrophilinae] in Hansen (1991: 282; 1999b: 155).
Acidocerina as tribe [of subfamily Hydrophilinae] in Short and Fikáček (2011: 85).
Acidocerina as subfamily in Short and Fikáček (2013: 741).
Helopeltini Horn, 1873: 118; synonymized by Hansen (1991: 282); unavailable: generic name is preoccupied (ICZN 1999, Code Art. 39).
Type genus: Helopeltis Horn, 1873: 137 [synonym of Helobata Bergroth, 1888: 221].
Helocharae d’Orchymont, 1919c: 147; described as subtribe, synonymized by Hansen (1991: 282).
Type genus: Helochares Mulsant, 1844a: 197.
Horelophopsinae Hansen, 1997: 108.
Type genus: Horelophopsis Hansen, 1997: 109; synonymized by Short and Fikáček (2013: 15, in table, discussed along the text).
Globulina García, 2001: 153; emended to Globulosina by Short and Hebauer (2006: 338); synonymized with tribe Acidocerini by Short and Fikáček 2011: 84.
Type genus: Globulosis García, 2001: 153.

##### Type genus.

*Acidocerus* Klug, 1855: 649.

##### Diagnosis.

**Figure 17.**
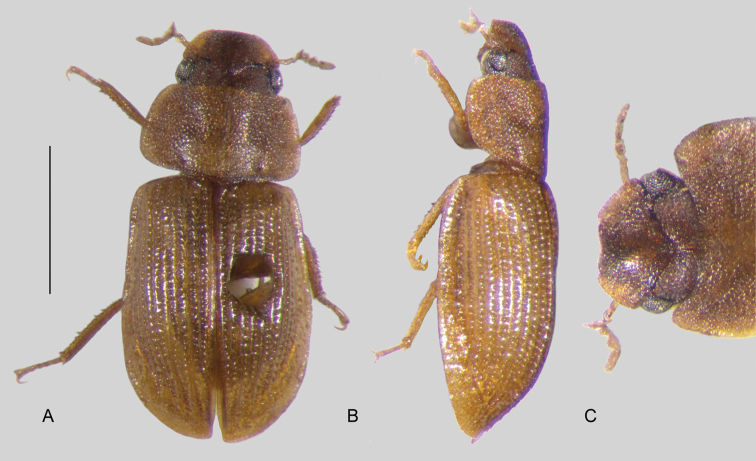
Habitus of *Acidocerus aphodioides***A** dorsal habitus **B** lateral habitus **C** head. Scale bar: 1 mm.

Body length 1.2–14.0 mm. Body shape oval in dorsal view, dorsoventrally flattened, or weakly to strongly convex in lateral view (Fig. 1); surface even (without elevations or depressions), granulate (e.g., Figs 17, 33) or smooth on head and pronotum. From yellowish to dark brown in coloration (Fig. 1), usually uniform, sometimes different regions of body colored differently. Shape of head variable (trapezoid, subquadrate, round; Fig. 11E–L). Anterior corners of frons sometimes extended posteriorly forming canthus and emarginating anterior margin of eyes (e.g., *Tobochares*, *Helobata*; e.g., Fig. 11B, C). Eyes varying in size, shape, degree of emargination, and degree of projection from outline of head (Fig. 11E–L); absent only in cavernicolous genus *Troglochares* Spangler, 1981a. Clypeus variable in shape (rectangular to trapezoid; Fig. 11E–L), with anterior margin from straight to mesally emarginate. Labrum usually exposed; concealed by clypeus in *Helobata* (Fig. 11L) and *Helopeltarium* (Fig. 1H). Mentum usually wider than long, with strong median anterior depression, may be limited by low transverse carina (Fig. 12A–C); surface of mentum with variable sculpture, ranging from smooth (Fig. 12A) to roughly punctate or obliquely striate (Fig. 12B). Antennae with eight or nine antennomeres (Fig. 12D, E), with cupule varying in symmetry and shape. Maxillary palps curved inward, ranging from very short (nearly half width of the head; e.g., *Quadriops reticulatus*, Fig. 12C) and stout, to very long and slender (nearly twice the width of the head; e.g., *Peltochares*, Fig. 11K). Pronotum evenly convex, usually with systematic punctures forming paired anterolateral semicircles and paired short posterolateral transverse bands. Elytra with or without sutural striae, with outer margins simple, slightly flared, or laterally explanate; elytral punctation variable (Fig. 13). Hind wings usually well developed (Fig. 15A–F), seldom reduced along apical region (Fig. 15G). Surface of prosternum flat (e.g., Fig. 14A, B), convex or rarely medially carinate (e.g., *Crucisternum*; Fig. 29C), with anterior margin straight or anteriorly projected. Posterior elevation of mesoventrite either only weakly bulging or with transverse (e.g., Fig. 14E, G) or longitudinal ridge (e.g., Fig. 14D, F); with strongly produced, anteriorly pointed and longitudinally carinate transverse ridge in *Crucisternum* (Fig. 14C). Anapleural sutures variable in shape and orientation. Metaventrite rather uniformly covered by hydrofuge pubescence (e.g., Fig. 14E), sometimes with posteromesal glabrous patch (e.g., Fig. 14D, F, G), sometimes also with posterolateral glabrous patches (e.g., Fig. 14D). Protibiae with anterior row of spines varying in shape and development; apical spurs of protibiae varying in development. Metafemora with tibial grooves of varying development; hydrofuge pubescence on anterior surface of metafemora absent, reduced to only basal or dorsal patch, or increasingly covering most of surface. Tarsomeres 5-5-5; tarsomeres variable in size, proportions, and dorsal and ventral coverage. Abdomen with five pubescent ventrites, density of setae ranging from sparse to very dense. Fifth abdominal ventrite with apex either rounded (Fig. 15I), truncate (Fig. 15J), or emarginate (Fig. 15H); apex with or without fringe of flat and stout setae. Aedeagus usually symmetrical (Fig. 16), with basal piece varying in size from longer than parameres (e.g., *Primocerus*, Fig. 47; *Batochares*, Fig. 22D), to reduced and virtually absent (e.g., *Peltochares*, Fig. 45); parameres highly variable in shape, either slender and only connected to each other at base of ventral surface (e.g., Fig. 16A–D, G, H), or fused together forming tube-like structure (e.g., Fig. 16E, F); apex of parameres either simple, or bifurcated and modified with hooks and spines (e.g., Fig. 16C, D); median lobe either simple or with dorsal and ventral lobes, with well-developed lateral basal apodemes; further modifications (longitudinal divisions, presence of internal hooks and spines, development of gonopore) widespread.

##### Differential diagnosis.

Acidocerines can be generally recognized by their oval and moderately convex body shapes with slender maxillary palps and uniformly slender tibiae (usually strongly convex and sometimes rounded in Cylominae and Sphaeridiinae, with short and stout maxillary palps and stout to apically broadened tibiae). The maxillary palps are always curved inwards in Acidocerinae (maxillary palpomere 2 with inner margin straight to concave; Fig. 12F–J), with palpomeres 2–4 similar in length and proportions (curved outwards, zig-zag oriented, or with shorter palpomere 3 in most Enochrinae and Chaetarthriinae). In addition, Acidocerines always bear five tarsomeres on the meso- and metatarsi (four in some enochrines).

##### Selected references.

Hansen 1991: diagnosis of the group (at the time as a subtribe, and including some genera now placed in the subfamily Enochrinae), list of genera and subgenera with synonyms, key to genera, and description of each genus (8 out of the 23 recognized in this paper). Hansen 1999b: catalog with full list of species at the time (nearly 300), synonyms and references. Short and Fikáček 2013: Acidocerinae as a subfamily excluding enochrine genera, with Horelophopsinae as synonym, list of genera, general diagnosis. Short et al. (2021): molecular phylogeny and biogeography of the subfamily, groups of genera.

##### Remarks.

The subfamily Acidocerinae is a group with many contrasts. It includes some of the largest as well as smallest hydrophilids; some genera are either strikingly different from, or extremely similar to others; the external morphology of some genera is extremely uniform and species can only be recognized by characters of the male genitalia, or so variable that is difficult to diagnose the group as a unit; at the species level, the distributions can be very narrow and restricted to one or a few fairly close localities, or very broadly widespread across several continents. There is a trend for species living in the same kind of habitats to have certain shared morphological features. For example, species that live in aquatic habitats tend to have slender and relatively long maxillary palps and metafemora mostly covered by hydrofuge pubescence, whereas species living in hygropetric habitats tend to have shorter and stouter maxillary palps and reduced or absent coverage of hydrofuge pubescence on the metafemora.

#### Genus Acidocerus

Taxon classificationAnimaliaColeopteraHydrophilidae

Klug, 1855

Figs 2 , 4 , 17

Acidocerus Klug, 1855: 649.

##### Gender.

Masculine.

##### Type species.

*Acidocerus aphodioides* Klug, 1855: 649; by monotypy.

##### Diagnosis.

Small beetles, body length nearly 2.8 mm. Body shape elongate oval in dorsal view, moderately convex in lateral view, with dorsal outline nearly straight along anterior 2/3 of elytra (Fig. 17). Surface of head and pronotum granulate (Fig. 17C). Body pale/yellowish brown, with head slightly darker. Eyes with anterior margin straight in lateral view (not emarginate), in dorsal view slightly projecting from outline of head (Fig. 17C). Labrum not concealed by clypeus (Fig. 17C). Antennae with nine antennomeres, with strongly asymmetric cupule, with longer side acute. Maxillary palps elongate, with palpomere 4 nearly as long as palpomere 3 (d’Orchymont 1943f: 7, in key). Elytra without sutural striae, narrowly explanate laterally, serial punctures strongly marked, arranged in rows (Fig. 17A). Prosternum flat, rather sharply carinate medially, with angulate anteromedian projection. Posterior elevation of mesoventrite only weakly bulging. Metaventrite with hydrofuge pubescence. Metafemora without distinct tibial grooves, mostly pubescent, only glabrous at apex. Metatarsomeres 1–4 similar in length; metatarsomere 5 similar in length to metatarsomeres 1–4 combined. Fifth abdominal ventrite apically emarginate, with stout setae.

##### Differential diagnosis.

The long fifth metatarsomere (longer than metatarsomeres 1–4 combined) is unusual but not unique in the subfamily (Hansen 1991). The granulate surface of the head and body resembles that of *Helobata*, but besides their geographic origin, the exposed labrum of *Acidocerus* (as opposed to concealed in *Helobata*) allows its recognition. The small size and coarse punctation of the elytra of *Acidocerus* resemble some of the Old World *Helochares* (e.g., Fig. 36D–F) and some *Agraphydrus* (e.g., *Agraphydrus hanseni*, Fig. 19A), from which it can be differentiated by the medially sharply carinate prosternum (Hansen 1991).

##### Distribution.

**Afrotropical**: Mozambique; Fig. 4.

##### Natural history.

There is no natural history information available for the genus.

##### Larvae.

Immature stages are not known for the genus.

##### Taxonomic history.

The taxon was originally described as related to *Spercheus* Kugelann, with maxillary palps similar to those of *Hydraena* Kugelann (Klug 1855), and even later afforded its own subfamily (see taxonomic history of the Acidocerinae section, above). d’Orchymont (1943f: 7) provided a list of diagnostic characters in a key, including the relative length of its tarsal segments, specifically that the fifth tarsomere is as long as tarsomeres 1–4 combined. Hansen (1991) redescribed the taxon based on syntypes. Hansen (1991: 149) further commented that he had seen other “typical” species of *Helochares* that also shared this feature and stated that “although *Acidocerus* may be somewhat reminiscent of a small *Helochares*… I prefer to maintain it as a distinct genus at the present stage”. The genus was not included in the molecular phylogeny in Short et al. (2021), and its assignment to the *Helochares* group is based primarily on its overall dorsal sculpturing, lack of a sutural stria, and Afrotropical distribution.

##### Remarks.

Only one described species. Hansen (1991) studied Klug’s syntypes housed at the Museum für Naturkunde der Humboldt-Universität in Berlin, Germany (ZMHB), which are the only known specimens for the genus. The diagnostic features listed above include information from d’Orchymont (1943f), Hansen (1991), and our own observations of photographs of the syntypes. Given that the specimens were mounted on cards when photographed, features of the ventral surface were not viewed by us. Characters of the ventral features (as well as the maxillary palps) as described above are based on d’Orchymont (1943f) and Hansen (1991), as the maxillary palps appeared to be missing by the time Hansen examined the syntypes. Until additional specimens are found, it is unlikely there will be a satisfactory resolution on deciding if *Acidocerus* is in fact a distinct genus or rather another variant of *Helochares*.

##### Species examined.

*Acidocerus aphodioides* (photographs of syntypes).

##### Selected references.

Klug 1855: 649: original description; d’Orchymont 1943f: 7: offers diagnostic features in a key; Hansen 1991: 149: redescription; Short and Fikáček 2013: 741: *Acidocerus* listed in subfamily Acidocerinae; Short et al. 2021: phylogenetic position and affinities discussed.

#### Genus Agraphydrus

Taxon classificationAnimaliaColeopteraHydrophilidae

Régimbart, 1903

**Figure 18.**
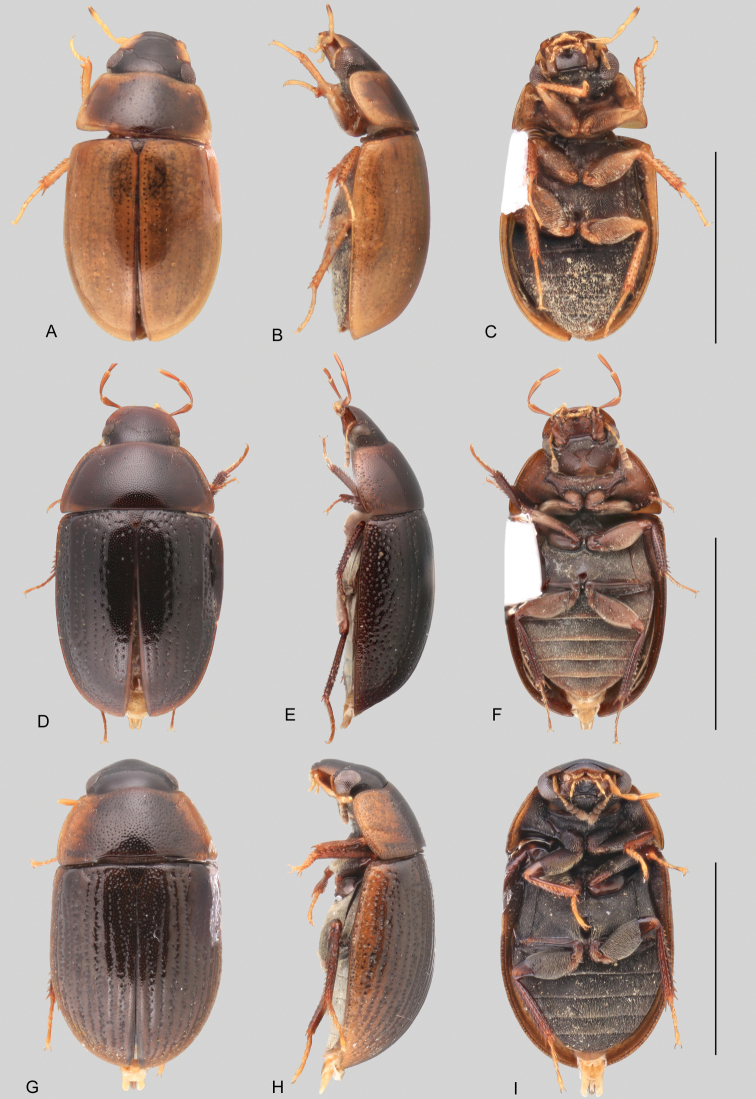
Habitus of *Agraphydrus* spp. **A–C***A. coomani*: **A** dorsal habitus **B** lateral habitus **C** ventral habitus **D–F***A. cf. attenuatus*: **D** dorsal habitus **E** lateral habitus **F** ventral habitus **G–I***A.* sp. ex Madagascar: **G** dorsal habitus **H** lateral habitus **I** ventral habitus. Scale bars: 1 mm.

**Figure 19.**
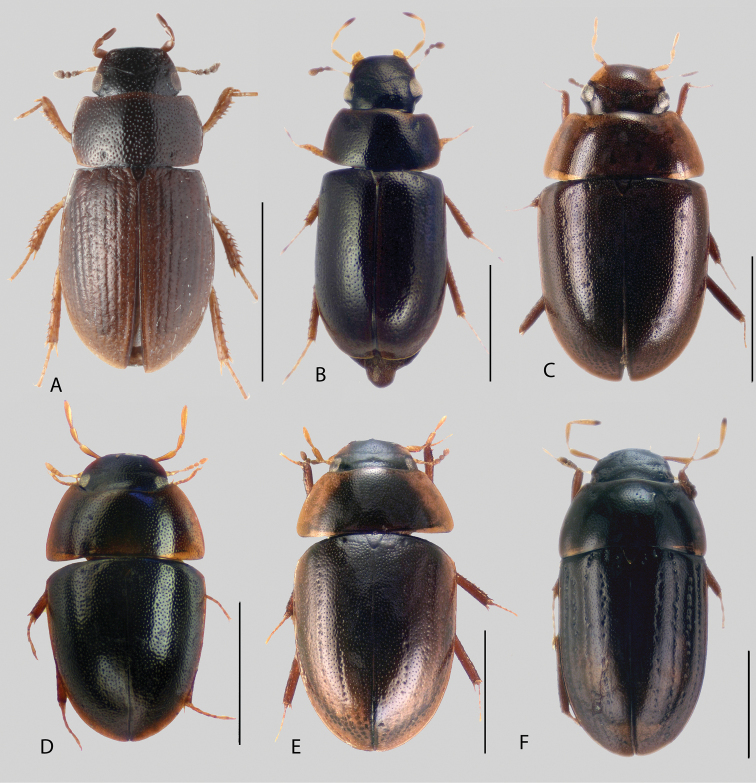
Habitus of *Agraphydrus* spp. **A***A. hanseni***B***A. jilanzhui***C***A. longipalpus***D***A. contractus***E***A. anhuianus***F***A. puzhelongi*. Images **B–F** from Komarek and Hebauer (2018). Scale bars: 1 mm.

**Figure 20.**
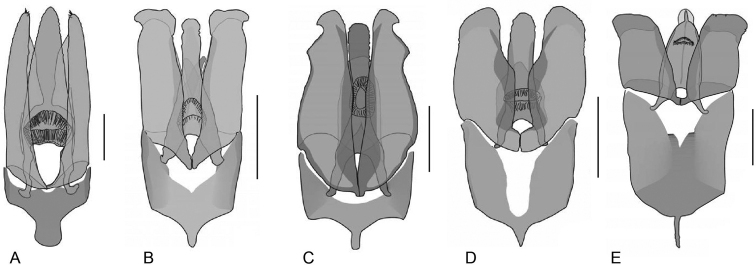
Aedeagi of *Agraphydrus* spp. **A***A. attenuatus***B***A. gracilipalpis***C***A. masatakai***D***A. chinensis***E***A. puzhelongi*. Scale bars: 0.1 mm. Line drawings taken from Komarek (2018).

Figs 1M, S, T , 2 , 4 , 18 , 19 , 20

Agraphydrus Régimbart, 1903a: 33.
Type species: Agraphydrus punctatellus Régimbart, 1903a: 34; by monotypy.
Pseudohelochares Satô, 1960: 77; Satô (1965: 128) [synonymy].
Type species: Pseudohelochares narusei Satô, 1960: 77; by original designation and monotypy.
Pseudopelthydrus Jia, 1998: 225.
Type species: Pseudopelthydrus longipalpus Jia, 1998: 229; by original designation. Komarek (2003: 384) [synonymy].
Megagraphydrus Hansen, 1999a: 137.
Type species: Megagraphydrus siamensis Hansen, 1999a: 140; by original designation. Minoshima et al. (2015: 7) [synonymy].
Gymnhelochares d’Orchymont, 1932: 692; as subgenus of Helochares.
Type species: Helochares (Gymnhelochares) geminus d’Orchymont, 1932: 694; by original designation. Komarek and Hebauer (2018: 17) [synonymy].
Horelophopsis Hansen, 1997: 109.
Type species: Horelophopsis avita Hansen, 1997: 109, by original designation; Short et al. (2021) [synonymy].

##### Gender.

Masculine.

##### Type species.

*Agraphydrus punctatellus* Régimbart, 1903: 34; by monotypy.

##### Diagnosis.

Small beetles, body length 1.4–4.8 mm. Body shape elongate to broadly oval in dorsal view, weakly to moderately convex in lateral view, rarely strongly convex (Figs 18, 19). Surface of head and pronotum smooth, usually with shallow ground punctation. Body ranging from pale/yellowish to dark brown (Figs 18, 19), either uniform across body regions or with different regions colored differently (e.g., darker head, paler elytra and margins of pronotum; Fig. 18A, B). Eyes with anterior margin straight in lateral view (not emarginate), in dorsal view slightly projecting from outline of head. Clypeus moderately convex, with distinct systematic punctures, with anterior margin slightly to clearly emarginate. Labrum not concealed by clypeus. Mentum nearly 1.5 × wider than long, with variable surface, with wide and moderate median anterior depression limited by low transverse carina. Antennae with eight or nine antennomeres, with slightly asymmetric cupule, round in outline. Maxillary palps elongate, 0.7–1.5 × width of head, with inner margin of palpomere 2 usually straight and palpomere 4 nearly as long to slightly longer than palpomere 3 (Fig. 12G). Pronotum with ground punctation usually moderate. Elytra without sutural striae, not laterally explanate, with serial punctures usually absent; systematic punctures usually rather sparse and aligned in four rows along elytra. Prosternum slightly convex, not carinate medially. Posterior elevation of mesoventrite variable, from simply bulged, to bearing variously shaped elevations; anapleural sutures variable in shape and orientation. Metaventrite with posteromedian glabrous patch. Metafemora without distinct tibial grooves, either mostly pubescent (only glabrous at apex), or with pubescence reduced to small basal area (“*Gymnhelochares*”). Metatarsomere 1 shorter than 2; metatarsomere 2 slightly shorter than 5; metatarsomere 5 similar in length to metatarsomeres 3 and 4 combined. Fifth abdominal ventrite apically emarginate, sometimes very slightly, or rounded, with or without fringe of stout setae. Aedeagus trilobed in form (Fig. 20); basal piece shorter to longer than parameres; outline of apical region of parameres variable; median lobe triangular, with well-developed lateral basal apodemes, usually rounded at apex; gonopore well developed.

##### Differential diagnosis.

*Agraphydrus* can be considered highly variable both morphologically and ecologically. Given their usually small to very small size, in the regions where *Agraphydrus* is distributed, they may be confused with smaller species of *Helochares*, from which *Agraphydrus* can be distinguished by the presence of a posteromesal glabrous patch on the metaventrite (metaventrite uniformly and densely covered by hydrofuge pubescence in *Helochares*); their size allows to differentiate them from the much larger *Colossochares* and *Peltochares*. The lack of sutural stria in *Agraphydrus* allows to recognize the larger *Agraphydrus* from similarly sized *Crephelochares*. The maxillary palps tend to be shorter in *Agraphydrus*. Most *Agraphydrus* have moderately punctate head and pronotum and generally lack elytral serial punctures; although they may have very coarse systematic punctures somewhat aligned in rows, these rows are not quite uniform as in many Old World *Helochares* or *Acidocerus*. The outer margins of the elytra of *Agraphydrus* are only slightly flared, as opposed to laterally expanded which differentiates them from *Batochares*. The most similar genus to *Agraphydrus* would be the Neotropical genus *Tobochares*, but they do not co-occur; the body shape in *Agraphydrus*, in general, tends to be more elongated (1.1–1.4 × longer than wide), whereas in *Tobochares* it tends to be only slightly longer than wide (1.07–1.15 × longer than wide); in addition, the metafemora in *Tobochares* are always mostly glabrous, with scattered setae, and their serial punctures are well aligned longitudinally.

##### Distribution.

**Afrotropical**: Angola, Botswana, Cameroon, Democratic Republic of the Congo, Djibouti, Eritrea, Eswatini, Ethiopia (in doubt), Gabon, Ghana, Guinea, Ivory Coast, Kenya, Madagascar, Malawi, Mozambique, Namibia, Nigeria, Oman, Republic of South Africa, Saudi Arabia, Sudan, Tanzania, United Arab Emirates, Yemen, Zimbabwe. **Australasian**: Australia (New South Wales, Northern Territory, Queensland, Western Australia), Indonesia (Java, Papua), Papua New Guinea. **Indo-Malayan**: Bhutan, Brunei, China (Fujian, Guangdong, Guangxi, Guizhou, Hainan, Himachal, Hong Kong, Hunan, Jiangxi, Taiwan, Yunnan, Zhejiang), India (Arunachal Pradesh, Assam, Goa, Himachal Pradesh, Kerala, Karnataka, Madhya Pradesh, Maharashtra, Meghalaya, North Andaman Island, Sikkim, Tamil Nadu, Uttar Pradesh, Uttarakhand), Laos, Malaysia, Myanmar, Nepal, Philippines, Sri Lanka, Thailand, Vietnam. **Palearctic**: China (Anhui, Gansu, Hubei, Shaanxi, Shandong, Sichuan, Tibet), Iran, Japan, Korea, Pakistan, South Korea; Fig. 4.

##### Natural history.

*Agraphydrus* can be found in an extremely broad range of habitats, from rivers, streams and forest pools, to hygropetric environments around waterfalls or seepages over rocks; a few species have been collected in terrestrial habitats by sifting moss and leaves from near water bodies, or in the gravel along the bank of a river; in many cases specimens have been found associated with floating vegetation, mosses and algae (Komarek 2018, 2019, 2020, Komarek and Freitag 2020, Komarek and Hebauer 2018).

##### Larvae.

Only the larvae of two species of *Agraphydrus* are currently known: *A. narusei* (Satô) (first and third instars; Minoshima and Hayashi 2011), and *A. hanseni* (Satô and Yoshitomi) (third instar; Minoshima et al. 2013). Minoshima (2016) offers a diagnosis for *Agraphydrus* larvae.

##### Taxonomic history.

Originally described as a genus by Régimbart in 1903; downgraded to a subgenus of *Enochrus* by d’Orchymont (1919c: 155); transferred as a subgenus to *Helochares* by d’Orchymont (1927a: 250); generic status re-established by Satô (1965: 128). Hansen (1991: 148) placed *Gymnhelochares* as a subgenus of *Agraphydrus*; Komarek and Hebauer (2018: 17) placed *Gymnhelochares* as a synonym of *Agraphydrus* given that they could not identify any unique morphological traits that allowed the two genera to be differentiated. Minoshima et al. (2015: 7) synonymized *Megagraphydrus* with *Agraphydrus* also based on the lack of morphological traits in support of their separation. Short and Fikáček (2013) recovered *Horelophopsis* and *Agraphydrus* as sister taxa within the Acidocerinae (*Horelophopsis* had been described as, and was prior to Short and Fikáček (2013), its own subfamily of Hydrophilidae). These affinities between *Agraphydrus* and *Horelophopsis* were also recognized by Minoshima et al. (2013) based on larval characters. Finally, Short et al. (2021), based on their molecular phylogenetic analyses, synonymized *Horelophopsis* with *Agraphydrus*, as *Horelophopsis* was recovered as a lineage nested within *Agraphydrus*. The genus was redescribed by Komarek (2020). For more details on the taxonomic history of the genus and its synonyms see Minoshima et al. (2015).

##### Remarks.

With 201 described species, *Agraphydrus* is currently the largest genus of Acidocerinae, due to a series of recent revisions and monographs (Minoshima et al. 2015; Komarek 2018, 2019, 2020; Komarek and Hebauer 2018; Komarek and Freitag 2020), making it the fifth largest genus of Hydrophilidae (behind *Berosus* Leach, *Laccobius* Erichson, *Cercyon* Leach, and *Enochrus* Thomson). The condition of the maxillary palpomere 2 being straight (with inner margin straight) is not unique to *Agraphydrus* but shared with *Tobochares* and some *Helochares*. Minoshima et al. (2015) proposed the V-shaped male abdominal sternite 9 as a possible synapomorphy of the genus, but the condition is shared with some members of the *Tobochares* group.

The genus appears well supported as monophyletic as currently defined, despite its substantial morphological and ecological variation (Short et al. 2021). Although previous decisions to synonymize derived genera (e.g., *Megagraphydrus*, *Pseudopelthydrus*, *Horelophopsis*) were necessary to preserve the monophyly of the broader concept of *Agraphydrus*, it has rendered the genus unmanageably large and with no internal formal or informal classification system. The lineage would be well-served by further phylogenetic studies to define species groups or to partition into subgenera.

Hebauer (2002a) listed several species of *Agraphydrus* as “in press”, and some specimens in collections bear associated red and orange holotype or paratype labels bearing these names; however, those were never formally published. Many of these taxa appeared in Komarek and Hebauer (2018) or subsequent revisions by Komarek (2019, 2020), with names different from those proposed by Hebauer (2002a).

##### Species examined.

*Agraphydrus anatinus* Komarek, *A. attenuatus* (Hansen), *A. coomani* (d’Orchymont), *A. decipiens* Minoshima, Komarek & Ôhara*, *A. hanseni* (Satô & Yoshitomi), *A. insidiator* Minoshima, Komarek & Ôhara*, *A. ishiharai* (Matsui), *A. kempi* (d’Orchymont), *A. luteilateralis* (Minoshima & Fujiwara)*, *A. malayanus* (Hebauer)*, *A. masatakai* Minoshima, Komarek & Ôhara*, *A. minutissimus* (Kuwert), *A. narusei* (Satô), *A. pauculus* (Knisch), *A. politus* (Hansen), *A. pygmaeus* (Knisch), *A. siamensis* (Hansen), *A. stagnalis* (d’Orchymont), *A. thaiensis* Minoshima, Komarek & Ôhara, and numerous unidentified specimens. For species marked with an asterisk, paratype specimens were studied.

##### Selected references.

Minoshima et al. 2015: character discussion, taxonomic history, synonymization of *Megagraphydrus*, description of seven new species; Komarek and Hebauer 2018: 17: synonymized the subgenus Gymnhelochares with *Agraphydrus*, taxonomic revision for China and Taiwan describing 33 new species; Komarek 2018: taxonomic revision for India describing 36 new species; Komarek 2019: taxonomic revision for South East Asia (except Philippines) and Australasian Region, describing 60 new species; Komarek and Freitag 2020: revision of the species from the Philippines describing nine new species and providing barcodes for the species treated therein; Komarek 2020: revision of the African and Western Asian species, describing 25 new species and redescribing the genus; Short et al. 2021: synonymization of *Horelophopsis* with *Agraphydrus*, phylogenetic placement of *Agraphydrus*.

#### Genus Aulonochares

Taxon classificationAnimaliaColeopteraHydrophilidae

Girón & Short, 2019

**Figure 21.**
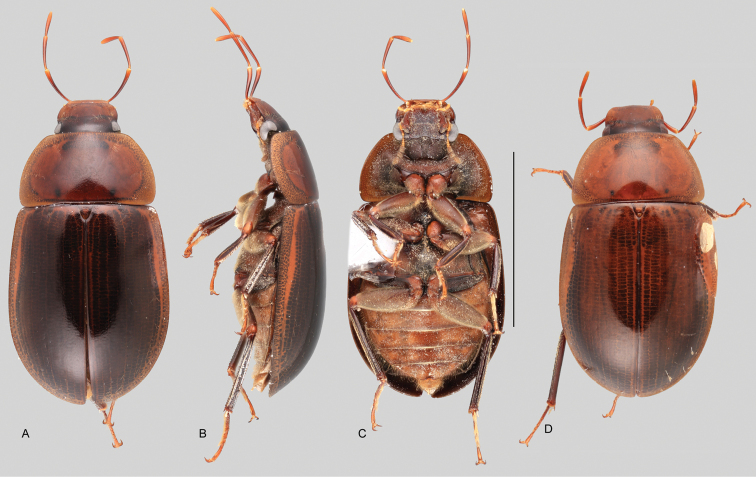
Habitus of *Aulonochares* spp. **A–C***A. tubulus*: **A** dorsal habitus **B** lateral habitus **C** ventral habitus **D***A. lingulatus*, dorsal habitus. Scale bar: 5 mm.

**Figure 22.**
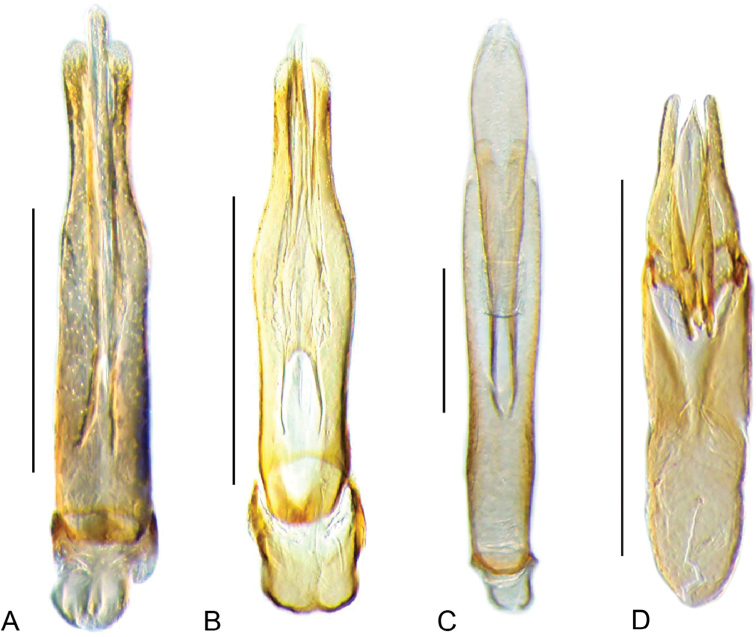
Aedeagi of *Aulonochares* and *Batochares* spp. **A***A. tubulus***B***A. novoairensis***C***A. lingulatus***D***B.* sp. Scale bars: 0.5 mm.

Figs 1D , 2 , 4 , 11J , 21 , 22A–C

Aulonochares Girón & Short, 2019: 112.

##### Gender.

Masculine.

##### Type species.

*Aulonochares tubulus* Girón & Short, 2019: 120; by original designation.

##### Diagnosis.

Medium sized beetles, total body length 5.8–7.5 mm. Body shape elongated oval in dorsal view; weakly convex in lateral view (Fig. 21). Color orange brown to dark brown; ventral surface covered with rather long golden setae, especially on abdominal ventrites, and more densely so (with shorter setae) on surface of femora. Head subquadrate in dorsal view, seemingly constricted at anterior margin of eyes (Fig. 11J). Eyes relatively small, separated by distance nearly 6.5 × the maximum width of an eye (Fig. 11J). Clypeus with lateral margins nearly parallel, slightly convex, with anterior margin only slightly narrower than posterior margin (Fig. 11J). Labrum fully exposed. Mentum and submentum roughly punctate (Fig. 21C). Antennae with nine antennomeres, with cupule slightly asymmetrical and round in outline. Maxillary palps long, nearly 1.5 × longer than maximum width of head, with inner and outer margins of maxillary palpomere 2 evenly curved (Fig. 21A). Pronotum with ground punctation shallow and uniformly sparse. Elytra without sutural striae, with outer margins slightly flared; serial punctures, ground punctures and systematic punctures similar in size, shallowly impressed. Surface of prosternum flat (slightly carinate only along midline of antero-mesal projection of anterior margin). Posterior elevation of mesoventrite simple, without carinae or ridges; anapleural sutures concave, anteriorly converging, anteriorly separated by distance nearly 0.3 × as wide as anterior margin of mesepisternum. Metaventrite densely and uniformly pubescent. Protibiae with spines of anterior row very small and appressed (Fig. 21C); apical spurs of protibiae very short (not exceeding the length of the first tarsomere) and stout. Hydrofuge pubescence covering most surface of metafemora (Fig. 21C). Ventral face of tarsomeres 1–4 densely covered by stiff setae. Apex of fifth abdominal ventrite strongly emarginate; emargination fringed by stout setae. Aedeagus tubular (Fig. 22A–C), somewhat cylindrical, with parameres forming a 5–7 × longer than wide tube; basal piece very short and strongly concave; gonopore reduced, located at apex of median lobe.

##### Differential diagnosis.

*Aulonochares* can be easily mistaken with *Novochares* in the New World, and the two genera can be collected together. The subquadrate shape of the head (Fig. 11J; as opposed to trapezoid as in Fig. 11G), the roughly punctate mentum, the long setae composing the ventral pubescence of the abdominal ventrites, ventrally densely setose tarsomeres, along with the tubular shape of the aedeagus (Fig. 22A–C) are very distinctive and uniquely combined in *Aulonochares* among Neotropical acidocerines.

##### Distribution.

**Neotropical**: Brazil (Amazonas, Roraima), French Guiana, Guyana, Suriname, Venezuela; Fig. 4.

##### Natural history.

Specimens of *Aulonochares* have been collected in densely forested sandy streams and detrital pools in forests along creeks. They seem to prefer habitats with abundant detritus or decaying organic matter. Females of *A. tubulus* and *A. ligulatus* have been observed carrying their egg cases underneath their abdomen (Girón and Short 2019; pers. obs.).

##### Larvae.

Immature stages are not known for the genus.

##### Taxonomic history.

Recently described by Girón and Short (2019).

##### Remarks.

Only three species are known for the genus (Girón and Short 2019).

##### Species examined.

*Aulonochares lingulatus* Girón & Short, *A. novoairensis* Girón & Short, *A. tubulus* Girón & Short. Holotypes and paratypes of all three species were available for this study. We have not seen any specimens of the genus from outside the Guiana Shield region of South America.

##### Selected references.

Girón and Short 2019: original description of the genus and all its currently known species; Short et al. 2021: phylogenetic placement.

#### Genus Batochares

Taxon classificationAnimaliaColeopteraHydrophilidae

Hansen, 1991

**Figure 23.**
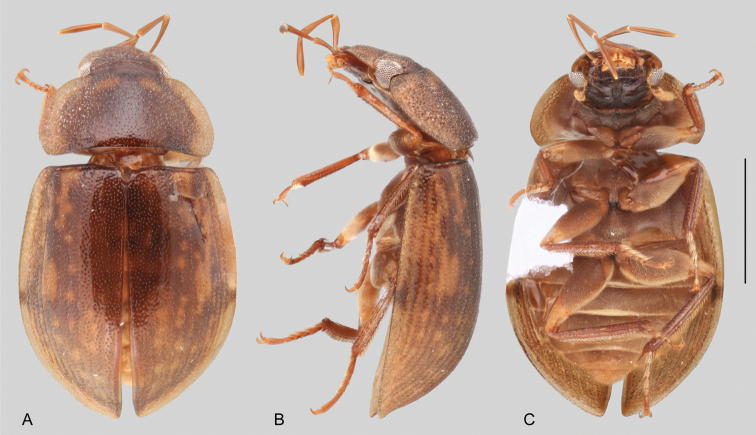
Habitus of *Batochares* sp. **A** dorsal habitus **B** lateral habitus **C** ventral habitus. Scale bar: 1 mm.

Figs 1I , 2 , 4 , 9D, E , 22D , 23

Batochares d’Orchymont, 1939b: 293 [Described as subgenus; unavailable, ICZN (1999) Art. 13.3: no type species designated].
Fixed as subgenus of Helochares by Hansen (1991: 292) [available, granting authorship to Hansen under ICZN (1999) Art. 50.1.].
Elevated to genus by Short et al. (2021).

##### Gender.

Masculine.

##### Type species.

Helochares (Batochares) burgeoni d’Orchymont, 1939b: 294; by original designation (Hansen 1991: 292).

##### Diagnosis.

Body length between 3–4 mm. Body shape oval in dorsal view, moderately convex in lateral view, with dorsal outline nearly straight along basal 2/3 (Fig. 23). Dorsal surfaces smooth, uniformly covered by short setae, brown to pale brown in coloration, either uniform or with yellowish patches along margins of pronotum and elytra, or scattered throughout surface giving spotted appearance (Fig. 23A, B); ground punctation fine and shallow; ventral surfaces rather densely covered by rather long and fine golden setae. Head rather oval in dorsal view, clearly constricted at anterior margin of eyes (Fig. 11E). Eyes not emarginate, moderate in size, separated by nearly 3.8 × width of eye, strongly projected from outline of head (Fig. 11E). Clypeus with anterior margin broadly emarginate, with medial region of emargination nearly straight; anterior corners round. Labrum fully exposed, with apical region anteriorly flattened, thus forming fine transverse carina across anterior region (Fig. 11D). Mentum rather flat, surface laterally punctate, mesally and anteriorly striate, with anteromedial region depressed. Submentum finely and shallowly punctate. Antennae with nine antennomeres, with strongly asymmetric and round cupule. Maxillary palps nearly 1.5 × longer than maximum width of head, with palpomere 4 0.8 × as long as palpomere 3 (Fig. 23C); inner margin of maxillary palpomere 2 nearly straight, outer margin apically slightly curved. Pronotum medially evenly convex, explanate and somewhat bending upwards along antero-lateral areas; posterior margin of pronotum clearly narrower than anterior margin of elytra combined. Elytra without sutural striae, with outer margins explanate, especially along anterior third; serial punctures well developed, forming longitudinal rows, at least well defined along outer areas, or visible along entire length of elytra; seta bearing systematic punctures irregularly distributed. Surface of prosternum slightly elevated along midline, with anterior margin acutely triangular and slightly projected anteriorly. Posterior elevation of mesoventrite rather flat; intercoxal process of mesoventrite broad (nearly as wide as antennal club), apically truncate; anapleural sutures sinuate, separated at anterior margin by distance slightly shorter than anterior margin of mesepisternum. Metaventrite with medial surface elevated as platform, densely covered with hydrofuge pubescence, except for posterolateral patches (Fig. 23C). Protibiae with spines of anterior row very fine and erect; apical spurs of protibiae small (larger spur similar in size and shape to tarsal claws). Metafemora without tibial grooves; metafemora with hydrofuge pubescence covering at least basal 2/3 of anterior surface (Fig. 23C). Metatarsomere 5 1.5 × longer than metatarsomere 2, metatarsomere 2 nearly as long as metatarsomeres 3 and 4 combined; tarsomeres 1 to 4 with sparse long setae on dorsal surface, and spiniform dense setae on ventral surface; tarsomere 5 with few setae along apical margin. Abdomen with five pubescent ventrites. Fifth abdominal ventrite with apex broadly truncate, without stout setae. Aedeagus trilobed, with basal piece nearly as long as parameres (Fig. 22D); parameres somewhat triangular, slender and apically narrowing; median lobe tapering to round apex; gonopore well-developed.

##### Differential diagnosis.

*Batochares* differs from all other known acidocerines by its unique labrum (with apical region anteriorly flattened, forming a transverse carina across anterior region; Fig. 11D), combined with oval head which is constricted at the anterior margins of the eyes, anterolaterally explanate pronotum, explanate elytra, rows of serial punctures visible at least along outer margins, broadly truncate posterior margin of fifth abdominal ventrite, and unusually large basal piece of the aedeagus (longer than parameres). These features, especially the configuration of the labrum, pronotum and elytra, along with the yellow spots along the surface of the elytra distinguish *Batochares* from all other known acidocerines.

##### Distribution.

**Afrotropical**: Burundi/Rwanda, Central African Republic, Democratic Republic of the Congo, Gabon, Guinea, Kenya, Republic of the Congo, Uganda; Fig. 4.

##### Natural history.

Little natural history information is available for the genus. Recent collecting data for a few series suggests it may be associated with the margins of streams and small rivers.

##### Larvae.

Immature stages for *Batochares* remain unknown.

##### Taxonomic history.

*Batochares* was described as a subgenus of *Helochares* by d’Orchymont (1939b) who did not explicitly designate a type species; therefore, the subgenus name was unavailable according to article 13.3 of the ICZN (1999). In 1991, Hansen validated *Batochares* as a subgenus of *Helochares* by fixing the type species for it; therefore, under article 50.1 of the Code (ICZN 1999), Hansen is granted authorship of the subgenus name. d’Orchymont considered *Batochares* as a subgenus of *Helochares* based for the most part in the number of antennomeres, relatively long maxillary palps, characters of the mentum and pubescent femora, even though the author recognized the distinctiveness of the shape of the head and the explanate elytra. *Batochares* was elevated to full generic status based on the phylogenetic analysis in Short et al. (2021), in which it was resolved as an early-diverging, isolated lineage within the *Helochares* group.

##### Remarks.

There are three species of *Batochares* described to date. In his description of *Batochares corrugatus*Balfour-Browne (1958a: 183), the author pointed out that his record of *B. burgeoni* from Mutsora, Parc National Albert (currently Virunga National Park, Democratic Republic of the Congo; Balfour-Browne 1950b) was not actually *B. burgeoni*, but a larger and likely different species. The author also indicated the existence of a different species from Angola.

##### Species examined.

*Batochares burgeoni* (d’Orchymont) and *B. byrrhus* (d’Orchymont).

##### Selected references.

d’Orchymont 1939b: 293: original description; Balfour-Browne 1958a: 183: description of one additional species; Hansen 1991: 292: type species designated, subgenus validated; Short et al. 2021: generic status, phylogenetic position and affinities discussed.

#### Genus Chasmogenus

Taxon classificationAnimaliaColeopteraHydrophilidae

Sharp, 1882

**Figure 24.**
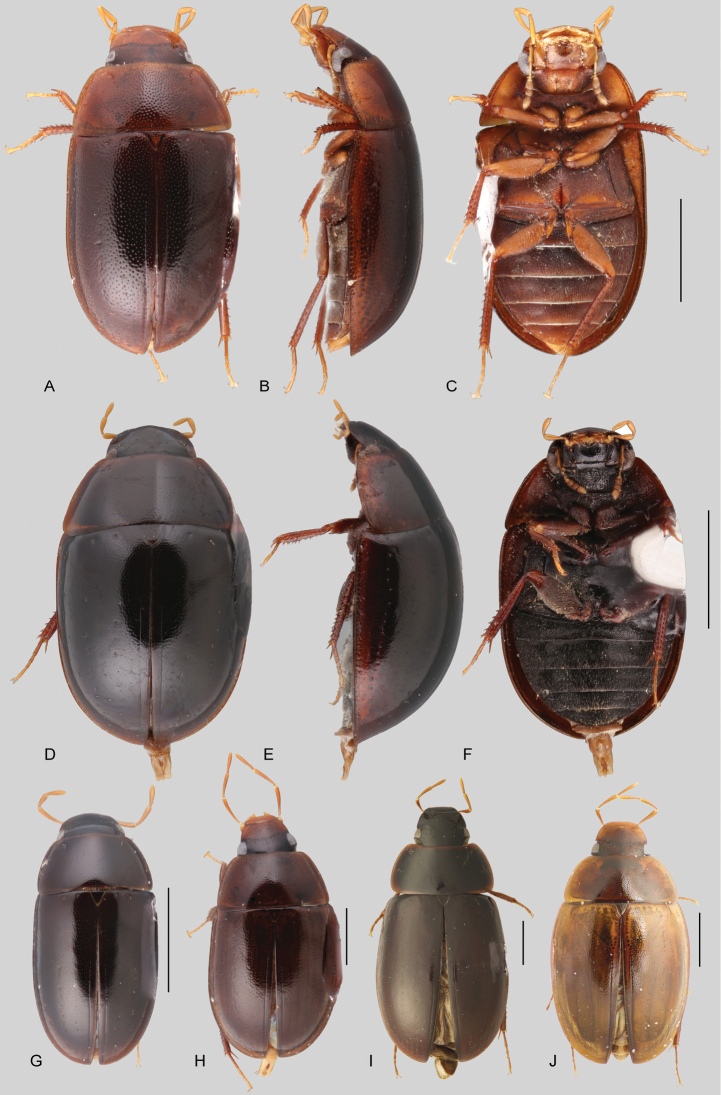
Habitus of *Chasmogenus* spp. **A–C***C. ruidus*: **A** dorsal habitus **B** lateral habitus **C** ventral habitus **D–F***C. cremnobates*: **D** dorsal habitus **E** lateral habitus **F** ventral habitus **G***C. lineatus***H***C. amplius***I***C. itatiaia***J***C. fluminensis*. **G, H** from Smith and Short 2020; **I, J** from Clarkson and Ferreira Jr 2014. Scale bars: 1 mm.

**Figure 25.**
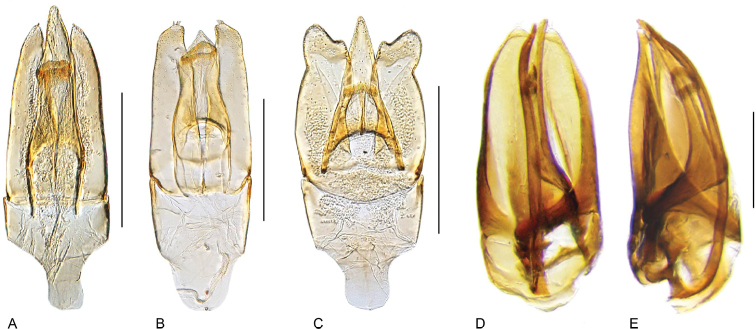
Aedeagi of *Chasmogenus* spp. **A***C. acuminatus***B***C. schmits***C***C. lineatus***D, E***C. tafelbergensis*: **D** dorsal view **E** lateral view. Images from Smith and Short 2020. Scale bars: 0.5 mm.

Figs 2 , 4 , 11H , 24 , 25

Chasmogenus Sharp, 1882: 73; Fernández 1986: 189 [generic status reinstated].
Type species: Chasmogenus fragilis Sharp, 1882: 73; by monotypy.
Helochares (Chasmogenus) Sharp; d’Orchymont 1919c: 149 [as subgenus of Helochares]; Knisch 1924: 195 [catalog].
Dieroxenus Spangler, 1979: 753; Girón and Short 2018: 154 [synonymy].
Type species: Dieroxenus cremnobates Spangler, 1979: 754; by original designation and monotypy.

##### Gender.

Masculine.

##### Type species.

*Chasmogenus fragilis* Sharp, 1882: 73; by monotypy.

##### Diagnosis.

Body length ranging from 2.5–5.0 mm. Body shape oval in dorsal view, parallel-sided to broader around midlength, dorsoventrally flattened, weakly to moderately convex in lateral view (Fig. 24), either evenly convex or flattened along anterior half. Surface of head, pronotum and elytra smooth, with usually shallow ground punctation. Coloration ranging from yellowish orange to dark brown, usually uniform along body, sometimes darker on head or only frons. Shape of head trapezoid (Fig. 11H). Eyes varying in size, usually subquadrate in dorsal view, only very weakly emarginated anteriorly, and usually projected from outline of head. Clypeus trapezoid, with anterior margin mesally weakly to strongly emarginated; membranous preclypeal area visible when clypeus strongly emarginated (Fig. 11H). Labrum fully exposed, semioval, anteriorly mesally emarginated. Mentum usually rather smooth, with anterior depression often reaching midlength of mentum, sometimes limited by low transverse carina. Antennae with eight antennomeres, with cupule slightly asymmetric and rounded. Maxillary palps usually slender and slightly longer than width of head, with inner margin slightly and evenly curved, and outer margin curved along apical half. Pronotum evenly convex. Elytra with sutural striae, with outer margins slightly flared; ground punctures usually only shallowly marked, serial punctures absent and at least one median row of systematic punctures clearly visible on each elytron (Fig. 24). Surface of prosternum usually flat, only rarely with low medial carina along intercoxal process. Posterior elevation of mesoventrite with an either blunt or sharp longitudinal elevation; anapleural sutures sinuate, separated at anterior margin by distance similar or slightly shorter than anterior margin of mesepisternum. Metaventrite with posteromesal and posterolateral glabrous patches (Fig. 24C). Protibiae with spines of anterior row semi erect, relatively long, thick and sparse; apical spurs of protibiae moderately long and thick, reaching apex of protarsomere 2. Metafemora with tibial grooves moderately developed, with sharp posterior margin; hydrofuge pubescence covering at least basal 3/4 of anterior surface of metafemora (Fig. 24C, F). Metatarsomeres 2–4 with two rows of spiniform setae on ventral surface; metatarsomere 5 nearly as long as 3 and 4 combined; metatarsomere 2 shorter to nearly as long as 5. Apex of fifth abdominal ventrite emarginate, with fringe of flat and stout setae. Aedeagus trilobed (Fig. 25); basal piece shorter to nearly as long as parameres; outline of apical region of parameres variable; sometimes parameres asymmetrical; median lobe triangular, either simple or bearing additional sclerite, with well-developed lateral basal apodemes and gonopore.

##### Differential diagnosis.

*Chasmogenus* most closely resembles *Crephelochares*, although they do not co-occur in the same biogeographic regions (*Chasmogenus* occurs exclusively in the Neotropical region, whereas *Crephelochares* occurs throughout the Old World). They can be differentiated by the number of antennomeres (eight in *Chasmogenus*, nine in *Crephelochares*) and by the form of the aedeagus (trilobed in most *Chasmogenus*, Fig. 25), divided and further modified in *Crephelochares*, Fig. 27B–D). Among New World taxa, *Chasmogenus* can easily be distinguished by the presence of sutural striae, a character shared only with *Primocerus*, from which it can be distinguished by the shape of the posterior elevation of the mesoventrite: longitudinally elevated in *Chasmogenus*, transversally elevated in *Primocerus*. Although *Primocerus* is quite rare and has a more restricted range in the Neotropics compared with *Chasmogenus*, the two genera can co-occur in forested steams in the Guiana Shield region.

##### Distribution.

**Neotropical**: Argentina, Brazil (Amapá, Amazonas, Minas Gerais, Pará, Piauí, Rio de Janeiro, Roraima, São Paulo), Costa Rica, Ecuador, French Guiana, Guatemala, Guyana, Panama, Paraguay, Suriname, Venezuela; Fig. 4.

##### Natural history.

The vast majority of *Chasmogenus* are known from forested habitats, including the margins of streams and forest pools. A few species are known from open marsh habitats (e.g., *Chasmogenus australis* García and *Chasmogenus sapucay* Fernández). They can be found among the vegetation and submerged leaf litter. They are also attracted to lights, though usually not in large numbers. Only one species [*Chasmogenus cremobates* (Spangler)] has been collected in seepages. See Smith and Short (2020) for more detail on habitat information.

##### Larvae.

The larvae of *Chasmogenus* remain unknown. The only descriptions of immature stages were made for *Chasmogenus nitescens* Fauvel (from Australia), which is now assigned to *Crephelochares*.

##### Taxonomic history.

*Chasmogenus* was originally described by Sharp (1882) as a genus to accommodate one Neotropical species from Guatemala and Panama. d’Orchymont (1919c: 149) synonymized *Chasmogenus* with *Crephelochares* (from the Old World) and placed it as a subgenus of *Helochares*. The generic rank of *Chasmogenus* was re-established by Fernández (1986: 189), with *Crephelochares* maintained as a junior synonym. Some authors continued to treat *Crephelochares* as a valid subgenus (e.g., Hebauer 1992, 1995) while others did not recognize any distinction between the two names (Hansen 1991, 1999). The monotypic genus *Dieroxenus* was synonymized with *Chasmogenus* by Girón and Short (2018). The recent phylogeny by Short et al. (2021) offered support considering *Chasmogenus* and *Crephelochares* as separate genera and affirmed *Dieroxenus* as a derived lineage within *Chasmogenus*.

##### Remarks.

There are 33 described species of *Chasmogenus* to date, and we are aware of many yet undescribed species in South America. *Chasmogenus* is a fairly commonly found group of beetles with very little variation in external morphology. Recent collecting efforts and taxonomic study in the genus have revealed a hidden diversity and interesting biogeographic patterns in South America (Smith and Short 2020).

##### Species examined.

*Chasmogenus australis* García*, *C. amplius* Smith & Short*, *C. bariorum* García*, *C. barrae* Short*, *C. cremnobates* (Spangler), *C. lineatus* Smith & Short*, C. *lorenzo* Short*, *C. ruidus* Short*, *C. schmits* Smith & Short*. Paratypes of the species marked with an asterisk were available for this study.

##### Selected references.

Sharp 1882: 73: genus description; Spangler 1979: 753: description of *Dieroxenus*; Fernández 1986: notes on the genus and one new species; Hebauer 1992: notes, recognition of two subgenera, emphasis on *Crephelochares*; García 2000: four new species from Venezuela; Short 2005: new species from Costa Rica; Short and Fikáček 2013: inclusion of *Chasmogenus* species in molecular phylogeny; Clarkson and Ferreira-Jr 2014b: four new species from Brazil; Girón and Short 2018: synonymization of *Dieroxenus*; Alves et al. 2020: description of a new species from Brazil; Smith and Short 2020: description of 18 new species from northeastern South America; Short et al. 2021: phylogenetic placement.

#### Genus Colossochares

Taxon classificationAnimaliaColeopteraHydrophilidae

Girón & Short gen. nov.

http://zoobank.org/4B774C0E-8A05-4DA7-8392-B809D29DDEE2

**Figure 26.**
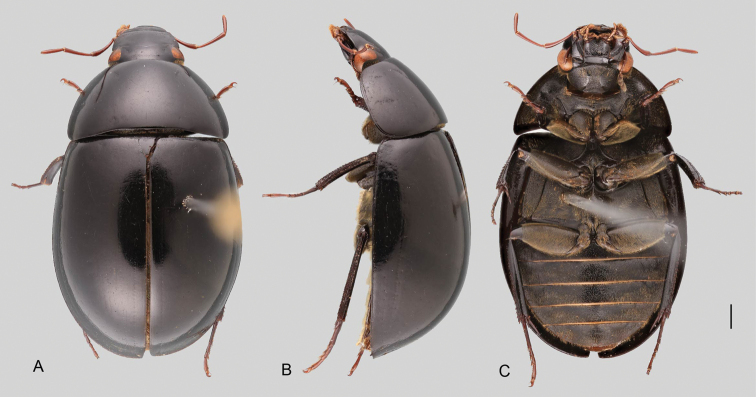
Habitus of *Colossochares ellipticus***A** dorsal habitus **B** lateral habitus **C** ventral habitus. Scale bar: 1 mm.

**Figure 27.**
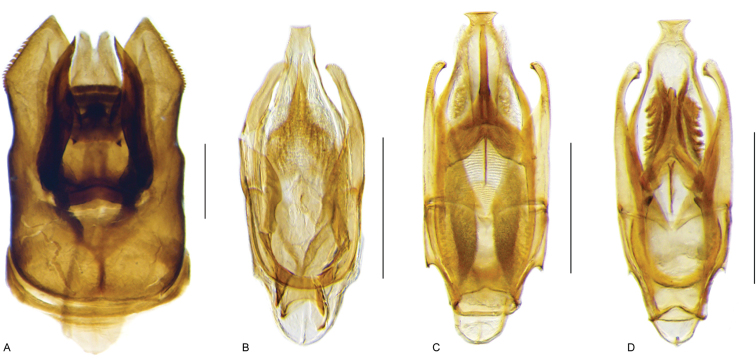
Aedeagi of *Colossochares* and *Crephelochares* spp. **A***Colossochares ellipticus***B***Crephelochares szeli***C***Crephelochares* sp. (Australia) **D***Crephelochares abnormalis* (Thailand). Scale bars: 0.5 mm.

Figs 1A , 2 , 4 , 11I , 26 , 27A

Helochares “Clade B”, Short et al. (2021).

##### Gender.

Masculine.

##### Type species.

*Helochares ellipticus* d’Orchymont, 1933: 306; by present designation.

##### Etymology.

From the Latin word *colossus*, meaning extremely large, in reference to the comparatively large and robust bodies of the members of the genus, combined with the ending *chares*, expressing affinity with *Helochares*. Masculine.

##### Diagnosis.

Body length 8.5–14.0 mm. Body shape broadly oval in dorsal view, strongly and uniformly convex in lateral view (Fig. 26). Dorsal surfaces even and smooth, uniformly dark brown (nearly black) in coloration with reddish antennae, palps and tarsi; ground punctation extremely fine and shallow (Fig. 26A); ventral surfaces rather densely covered by rather long and fine golden setae (Fig. 26C). Eyes not emarginate, moderate in size, subquadrate in dorsal view, separated by nearly 4 × width of eye, projected from outline of head (Fig. 11I). Frons with large (and somewhat fused together) systematic punctures along inner margin of eye. Clypeus with anterior margin broadly roundly emarginate. Labrum fully exposed, medially convex (Fig. 11I). Antennae with nine antennomeres, with strongly asymmetric and round cupule. Maxillary palps slender, slightly longer than maximum width of head, with palpomere 4 0.7 × as long as palpomere 3 (Fig. 11I). Mentum medially broadly depressed, laterally punctate, mesally and anteriorly striate; sculpture of mentum ranging from shallow to strong. Pronotum evenly convex, and very smooth, with ground punctation very fine and shallow; systematic punctures of pronotum reduced to paired depressions near anterior margin and at midlength of lateral margins. Elytra without sutural striae, with margins slightly flared; serial punctures either absent or only visible along outer lateral area and posterior third of elytra; systematic punctures enlarged, broadly separated longitudinally, forming five rows mostly visible along outer lateral area and posterior third of elytra (Fig. 26A, B). Surface of prosternum flat to broadly convex, with anterior margin slightly projected anteriorly (Fig. 26C). Posterior elevation of mesoventrite with broad longitudinal elevation; anapleural sutures concave, anteriorly converging and separated by distance nearly 1/3 of anterior margin of mesepisternum. Metaventrite uniformly densely covered by with hydrofuge pubescence, medial surface elevated as platform. Protibiae with anterior row of spines extremely reduced to tiny and scanty, appressed denticles; apical spurs of protibiae large, outer nearly as thick and reaching apex of protarsomere 2. Metafemora with tibial grooves well-developed; metafemora with hydrofuge pubescence covering basal 4/5 of anterior surface (Fig. 26C). Metatarsomeres laterally compressed, metatarsomere 2 longer than 5, metatarsomere 5 nearly as long as 3 and 4 combined; all tarsomeres with rows spiniform setae covering ventral surface. Fifth abdominal ventrite with apex emarginate, with fringe of flat and stout setae. Aedeagus symmetrical, either trilobed (*C. satoi*; Hebauer 2003a: fig. 1) or highly modified (Fig. 27A), with basal piece shorter than parameres; median lobe variable.

##### Differential diagnosis.

*Colossochares* groups some of the largest acidocerines. *Colossochares* species are strongly and uniformly convex and highly polished, with enlarged systematic punctures on the head and elytra; systematic punctures on the pronotum are reduced to a pair of anterior and a pair of lateral depressions, not forming the usual antero-lateral semicircles that are common in acidocerines. Some members of *Peltochares* may exhibit similar coloration and general highly polished appearance to *Colossochares* (e.g., compare Fig. 1A vs. 1B); those *Peltochares* are always dorsoventrally flattened, generally slender, and the pronotum has systematic punctures forming antero-lateral semicircles. Other than general appearance, both genera are very similar to each other in details of the external morphology, except by the sculpture of the submentum, which is smooth in *Colossochares* and punctate or otherwise sculptured in *Peltochares*. In addition, the aedeagal form in *Peltochares* (spiked, Fig. 16C, D) is quite different from the forms present in *Colossochares* (trilobed or as in Fig. 27A).

##### Distribution.

**Afrotropical**: Benin, Burkina Faso, Cameroon, Democratic Republic of the Congo, Ethiopia, Gabon, Ghana, Guinea, Ivory Coast, Liberia, Malawi, Nigeria, Republic of the Congo, Uganda; Fig. 4.

##### Natural history.

Little is known about the biology of *Colossochares*, and no museum specimens we examined contained any habitat or collecting information. We have seen some light trap samples from Congo in which *C. ellipticus* is relatively common.

##### Larvae.

The larvae of species of *Colossochares* remain unknown.

##### Taxonomic history.

Given how large and distinctive *Colossochares* species are, it is remarkable that it has not been previously recognized as a separate genus, especially given how many other genera and subgenera have been described based on less striking features. The reason may have been due in part to an original identification error: Régimbart (1907: 47) first gave a description for what is now *Helochares ellipticus*, but mistakenly thought they were conspecific with another already-described central African taxon, *Hydrophilus ellipticus* Fabricius. Régimbart (1907), based on this incorrect interpretation of his specimens, further recognized that they were not allied with *Hydrophilus* and instead shared similarities with *Helochares*, so he transferred Fabricius’ species to *Helochares*, creating the combination *Helochares ellipticus* (Fabricius). Later, d’Orchymont (1933) recognized Régimbart’s error and clarified the situation, confirming *Helochares ellipticus* as a valid species of *Helochares*, and also different from the original *Hydrophilus ellipticus* Fabricius.

Hebauer (2003) described *Helochares satoi* Hebauer and discussed its affinities with *Helochares ellipticus*. A specimen of *Helochares ellipticus* was included in the molecular phylogeny by Short et al. (2021), where it was resolved as an early-diverging and isolated member of the *Helochares* group of genera. Given that it is not nested within *Helochares*, and it is morphologically distinct, the genus *Colossochares* is here established to house the two species: *Colossochares ellipticus* (d’Orchymont) comb. nov. and *Colossochares satoi* (Hebauer) comb. nov.

##### Remarks.

Despite the external similarity between the two known species of *Colossochares*, the male genitalia are quite different from each other. This particularity is quite unusual in the subfamily given that, in general, each genus has a particular aedeagal type shared by all its species (though there are some known exceptions, e.g., *Chasmogenus*). The genitalia of *C. satoi* can be categorized as trilobed, whereas that of *C. ellipticus* is quite uniquely configured (Fig. 27B). More work is needed to confirm the close relationship of these two taxa.

##### Species examined.

Specimens of *Colossochares ellipticus* (d’Orchymont) and female paratypes of *C. satoi* (Hebauer) were available for study.

##### Selected references.

Régimbart 1907: 47: description of *Helochares ellipticus* attributed to Fabricius; d’Orchymont 1933: 306: clarification and reaffirmation of species name; Hebauer 2003: new species and discussion of affinities; Short et al. 2021: phylogenetic placement.

#### Genus Crephelochares

Taxon classificationAnimaliaColeopteraHydrophilidae

Kuwert, 1890

**Figure 28.**
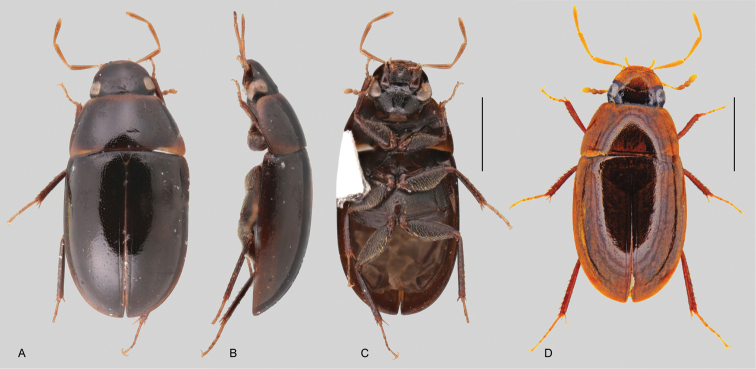
Habitus *Crephelochares* spp. **A–C***Crephelochares nitescens*: **A** dorsal habitus **B** lateral habitus **C** ventral habitus **D***Crephelochares cf. patrizii* (image from Bird et al. 2017). Scale bars: 1 mm.

Figs 1V , 2 , 4 , 11G , 27B–D , 28

Helochares (Crephelochares) Kuwert, 1890a: 38.
Helochares (Crepidelochares) Ganglbauer, 1904: 248 [unjustified emendation of Crephelochares Kuwert, 1890].
Helochares (Chasmogenus) Kuwert; d’Orchymont 1919c: 148 [taxonomic treatment]; Knisch 1924a: 195 [catalog].
Crephelochares Kuwert; Fernández 1986: 148 [junior synonym of Chasmogenus as genus]; Hansen 1991: 293 [catalog]; Short et al. 2021 [elevated to generic rank].
Chasmogenus (Crephelochares) Kuwert; Hebauer 1992: 62 [as subgenus of Chasmogenus].

##### Gender.

Masculine.

##### Type species.

*Helochares livornicus* Kuwert, 1890: 38; subsequent designation by d’Orchymont (1939a: 154).

##### Diagnosis.

Body length ranging from 2.5–4.8 mm. Body shape oval in dorsal view, dorsoventrally slightly flattened, moderately convex in lateral view, with dorsal outline nearly evenly convex (Fig. 28); surface even and smooth, with usually shallow ground punctation (Fig. 28). Coloration usually dark brown seldom yellowish, uniform across body regions. Head trapezoid (Fig. 11G). Eyes relatively large, at most only slightly emarginated anteriorly, and not or only slightly projected from outline of head. Clypeus trapezoid, with anterior margin mesally emarginate; membranous preclypeal area visible when clypeus strongly emarginated. Labrum fully exposed. Mentum punctate or punctate laterally and medially obliquely striate; medial surface flat to depressed (Fig. 28C); anteromedial depression sometimes limited by low transverse carina. Antennae with nine antennomeres, with cupule slightly asymmetric and rounded. Maxillary palps slender, 1.2–1.5 × longer than width of head; maxillary palpomere 4 nearly 0.7 × length of maxillary palpomere 3; inner margin of maxillary palpomere 2 nearly straight, and outer margin curved along apical half. Pronotum evenly convex. Elytra with sutural striae, with outer margins slightly flared; ground punctures usually only shallowly marked, serial punctures absent and at least one median row of systematic punctures visible on each elytron (Fig. 28). Surface of prosternum usually flat, sometimes tectiform. Posterior elevation of mesoventrite with longitudinal carina; anapleural sutures sinuate, separated at anterior margin by distance similar to slightly shorter than anterior margin of mesepisternum. Metaventrite with posteromesal and posterolateral glabrous patches (Fig. 28C). Protibiae with spines of anterior row semi erect, relatively long, thick and sparse; apical spurs of protibiae relatively short and stout, not reaching apex of protarsomere 2. Metafemora with tibial grooves moderately developed; hydrofuge pubescence covering basal 4/5 of anterior surface of metafemora (Fig. 28C). Metatarsomeres 2–4 gradually decreasing in size, with two rows of spines on ventral surface; metatarsomere 2 slightly longer than 5, 5 shorter than 3 and 4 combined. Fifth abdominal ventrite emarginate at apex, with fringe of flat and stout setae. Aedeagus (Fig. 27B–D) with parameres at most only fused at base on dorsal surface; median lobe divided in dorsal and ventral plates; dorsal plate sclerotized along margins, medially membranous, membranes with papillae or denticles along apico-medial region; ventral plate as inverted Y, sometimes accompanied by basal median laminar sclerite; basal piece nearly as long as or longer than ventral length of parameres, always noticeable; gonopore not clearly visible.

##### Differential diagnosis.

Among Old World acidocerines, *Crephelochares* is unique in the presence of sutural stria. The Neotropical *Chasmogenus* is the most similar genus, as they both share this character (along with the more distantly related Neotropical genus *Primocerus*). They can be differentiated by the number of antennomeres (eight in *Chasmogenus*, nine in *Crephelochares*) and by the form of the aedeagus (trilobed in *Chasmogenus*, Fig. 25; divided and further modified in *Crephelochares*, Fig. 27B–D). The configuration of the aedeagus in *Crephelochares* is quite unique in Acidocerinae, especially because of the configuration of the median lobe and its inner membranes.

##### Distribution.

**Afrotropical**: Angola, Benin, Botswana, Burundi, Cameroon, Democratic Republic of the Congo, Gabon, Gambia, Ghana, Guinea, Kenya, Liberia, Madagascar, Mauritius, Mozambique, Namibia, Niger, Nigeria, Rwanda, Senegal, Seychelles (Aldabra), Sierra Leone, Somalia, Republic of South Africa, Sudan, Tanzania, Uganda, Zambia, Zimbabwe. **Australasian**: Australia (New South Wales, Northern Territory, Queensland), Fiji (Vanua Levu, Viti Levu), New Caledonia, Papua New Guinea. **Indo-Malayan**: Cambodia, China (Guangdong, Hong Kong, Taiwan, Yunnan), Indonesia (Borneo, Java, Papua, Sulawesi, Sumatra), Laos, Malaysia, Sri Lanka, Thailand, Vietnam. **Palearctic**: Bosnia, Croatia, Greece, Israel, Italy, Japan, Serbia and Montenegro, Spain, Tunisia, Turkey; Fig. 4.

##### Natural history.

Archangelsky (1997: 55) reproduced the larval descriptions by Anderson (1976), who reared larvae from adults of *Crephelochares nitescens* (as *Helochares nitescens*) in laboratory conditions. According to Anderson (1976: 223), females lay between 18 and 25 eggs, “located below the surface of damp soil, in a mossy hollow constructed by the adult; the hollow was always of the same size and shape and lined inside with loose silk. Eggs were deposited at right angles to base of nest, each covered by strands of fine silk attached to floor, walls and adjacent eggs”. The larvae hatch in 5–7 days and are predaceous (Archangelsky 1997: 55). “The larvae would not pupate in damp tissue paper, but only in moss. […]. The larvae pupated naked in the upper moss or in curled decaying leaves” (Anderson 1976: 223). Complete development lasted 24–33 days. Fikáček (2003) provided a diagnosis, pointed out the incompleteness of the descriptions and drawings offered by Anderson (1976), and commented on the unusualness of the habit of laying eggs on the ground by hydrophilid standards.

As for the adults, ecological information is very scarce. According to Hebauer (1992), *C. livornicus* (Kuwert) was collected in stagnant water with decaying plants and *C. orbus* (Watanabe) was collected in a rice field. The recently described *C. parorbus* (Jia and Tang) was also recorded from stagnant waters (Jia and Tang 2018).

##### Larvae.

The only species for which immature stages are known is *Crephelochares nitescens* [from Australia; immature stages were originally described as *Helochares nitescens* by Anderson (1976)]. Anderson (1976) described the breeding method he used, the eggs and egg case, first and third instar larvae and pupa, as well as the entire life cycle. Archangelsky (1997: 55) reproduced Anderson’s (1976) findings.

##### Taxonomic history.

*Crephelochares* was originally described as a subgenus of *Helochares* by Kuwert (1890: 38). In 1904, Ganglbauer established *Crepidelochares* without justification or explanation. Later, d’Orchymont (1919c: 148) synonymized *Crephelochares* with *Chasmogenus* keeping *Chasmogenus* as a subgenus of *Helochares*. In 1986, Fernández reinstated *Chasmogenus* as a genus, with *Crephelochares* as a junior synonym. Subsequent authors alternately either treated *Crephelochares* as a subgenus or junior synonym. Hebauer (1992) removed *Crephelochares* from synonymy with *Chasmogenus*, and established it as a subgenus of *Chasmogenus*, discussing morphological features in support of this view, which he maintained in subsequent works (Hebauer 1995). However, Hansen (1991, 1999b) viewed the differences in antennomeres and the aedeagal complexity as “rather subtle” and maintained the two names as synonymous without subgeneric division. The phylogenetic analysis by Short et al. (2021), together with the morphological evidence offered by Hebauer, resulted in the recognition of the generic status of *Crephelochares*.

##### Remarks.

There are 29 species of *Chephelochares* described to date; some of the older species have long lists of synonyms. The most comprehensive treatment for the genus was by Hebauer (1992); the genus was then considered as a subgenus of *Chasmogenus*.

##### Species examined.

*Crephelochares abnormalis* (Sharp), *C. africanus* (d’Orchymont), *C. balkei* (Short)*, *C. irianus* (Hebauer)*, *C. livornicus* (Kuwert), *C. mauritiensis* (Balfour-Browne), *C. molinai* (Hebauer)*, *C. nitescens* (Fauvel), *C. orbus* (Watanabe), *C. paramollis* (Hebauer)*, *C. patrizii* (Balfour-Browne), *C. punctulatus* (Short)*, *C. ruandanus* (Balfour-Browne), *C. rubellus* (Hebauer)*, *C. rusticus* (d’Orchymont), *C. rutiloides* (d’Orchymont), *C. rutilus* (d’Orchymont), *C. szeli* (Hebauer)*. For species marked with an asterisk, paratypes were available.

##### Selected references.

Hebauer 1992: diagnosis, key to species, diagnoses, descriptions for 22 species, and genitalia drawings for 19 of them; Hebauer 1995: one new species from Namibia; Watts 1995: revision of the Australian species of the genus; Short 2010: revision of the species from the Southwest Pacific islands, describing two new species from Fiji and newly recording *C. nitescens* (Fauvel) for New Caledonia; Devi et al. 2016: redescription and lectotype designation for *C. abnormalis* (Sharp) with a discussion on its distribution and morphological variation; Short et al. 2021: generic status and phylogenetic placement.

#### Genus Crucisternum

Taxon classificationAnimaliaColeopteraHydrophilidae

Girón & Short, 2018

**Figure 29.**
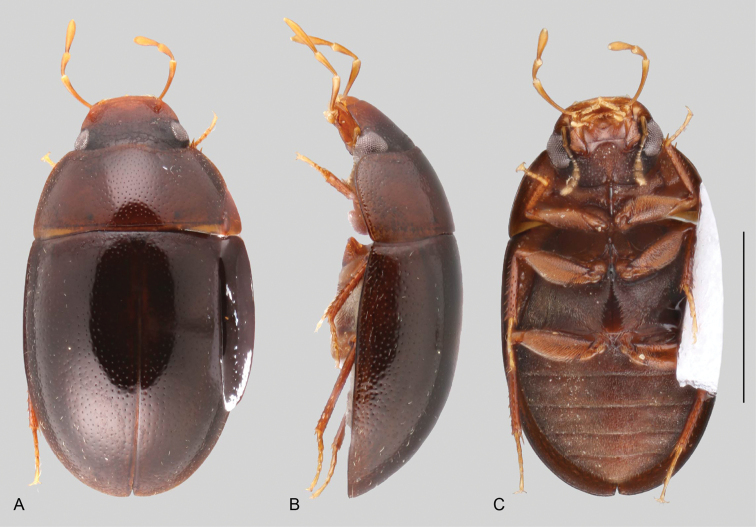
Habitus of *Crucisternum ouboteri***A** dorsal habitus **B** lateral habitus **C** ventral habitus. Scale bar: 1 mm.

**Figure 30.**
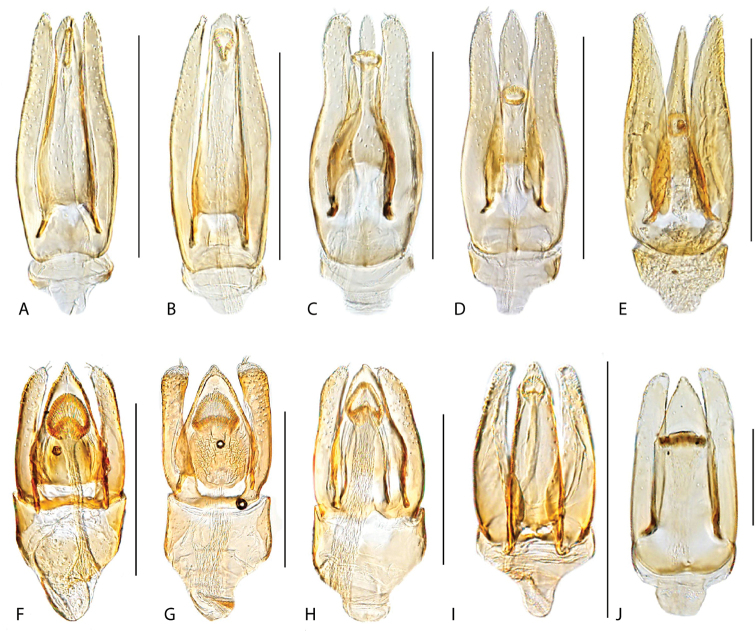
Aedeagi of *Crucisternum*, *Ephydrolithus*, and *Globulosis* spp. **A***C. ouboteri***B***C. toboganensis***C***C. sinuatus***D***C. vanessae***E***C. queneyi***F***E. teli***G***E. spiculatus***H***E. ogmos***I***E. minor***J***G. flavus*. Scale bars: 0.25 mm.

Figs 1Q , 2 , 5 , 14C , 29 , 30A–E

Crucisternum Girón & Short, 2018: 116.

##### Gender.

Masculine.

##### Type species.

*Crucisternum ouboteri* Girón & Short, 2018: 121; by original designation.

##### Diagnosis.

Small beetles, body length 2.0–2.5 mm. Body shape elongated oval in dorsal view; moderately convex in lateral view (Fig. 29). Color orange brown to dark brown. Head trapezoid. Eyes moderate to small, projected from outline of head. Clypeus trapezoid, with anterior margin broadly and roundly emarginate. Labrum fully exposed. Mentum with lateral oblique ridges; anterior median depression marked by transverse carina (Fig. 29C). Antennae with nine antennomeres, with cupule only slightly asymmetrical and rounded. Maxillary palps moderately long, slightly longer than width of head (Fig. 29A). Elytra without sutural striae, with outer margins of elytra slightly flared; serial punctures, ground punctures and systematic punctures similar in size and degree of impression, either shallow or rather sharply marked; all punctures seemingly arranged in rows (Fig. 29A). Prosternum with well-developed median, longitudinal, laminar carina (Fig. 29C). Posterior elevation of mesoventrite with a strongly produced, anteriorly pointed transverse ridge, longitudinally carinate (Fig. 14C); anapleural sutures sinuate, separated by distance nearly 0.6 × width of anterior margin of mesepisternum. Metaventrite densely pubescent, except for median and postero-lateral glabrous patches (Fig. 29C). Protibiae with spines of anterior row long and thick; apical spurs of protibiae short and stout, almost reaching apex of protarsomere 2. Metafemora covered by hydrofuge pubescence along basal 4/5 (Fig. 29C). Metatarsomeres 2–4 gradually slightly decreasing in size; metatarsomere 5 slightly longer than 2; ventral coverage of tarsomeres composed of fine and spiniform setae. Fifth abdominal ventrite apically rounded, truncate, or slightly emarginate, without stout setae. Aedeagus trilobate (Fig. 30A–E); basal piece 0.2–0.25 × the length of parameres; median lobe with well-developed lateral basal apodemes, and acute to narrowly rounded apex; parameres nearly as long as median lobe, with outer margins usually sinuate; gonopore situated distad of midlength of median lobe.

##### Differential diagnosis.

Although *Crucisternum* is generally unremarkable dorsally from other small-bodied Neotropical acidocerines, several sternal features are strikingly unique and easily separate the genus from all others. The strongly developed prosternal carina found in the genus, combined with the cruciform shape of the posterior elevation of the mesoventrite (formed by the fusion of both transverse and longitudinal ridges), is unique for this genus in the subfamily. *Crucisternum* is most likely to be confused in samples as a very small *Chasmogenus* but can also easily be distinguished from that genus by the lack of sutural striae.

##### Distribution.

**Neotropical**: Brazil (Minas Gerais, Pará), French Guiana, Guyana, Suriname, Venezuela; Fig. 5.

##### Natural history.

All species of the genus are associated with forested streams, usually along margins that contain ample detritus. A single specimen of *C. ouboteri* was collected at a black light trap.

##### Larvae.

Immature stages are not known for the genus.

##### Taxonomic history.

The genus was only recently described.

##### Remarks.

There are seven species currently known.

##### Species examined.

Holotypes and paratypes of all the known species were examined for this study.

##### Selected references.

Girón and Short 2018: original description of the genus and all its known species; Short et al. 2021: phylogenetic placement.

#### Genus Ephydrolithus

Taxon classificationAnimaliaColeopteraHydrophilidae

Girón & Short, 2019

**Figure 31.**
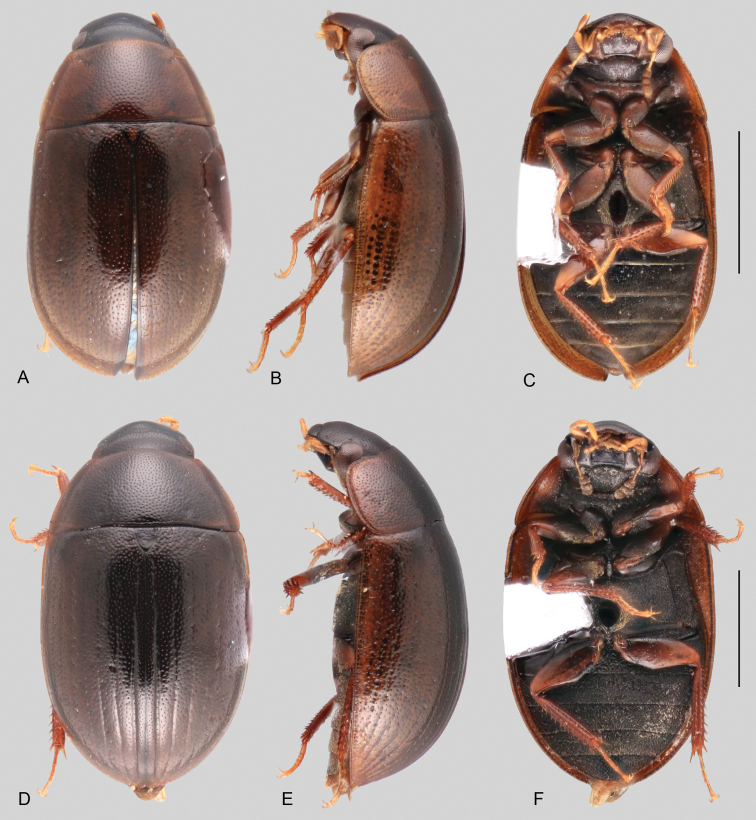
Habitus of *Ephydrolithus* spp. **A–C***E. hamadae*: **A** dorsal habitus **B** lateral habitus **C** ventral habitus **D–F***E. ogmos*: **D** dorsal habitus **E** lateral habitus **F** ventral habitus. Scale bars: 1 mm.

Figs 2 , 5 , 30F–I , 31

Ephydrolithus Girón & Short, 2019: 122.

##### Gender.

Masculine.

##### Type species.

*Ephydrolithus hamadae* Girón & Short, 2019: 130; by original designation.

##### Diagnosis.

Small beetles, body length 1.8–3.3 mm. Body shape oval in dorsal view, moderate to strongly convex in lateral view (Fig. 31); with ground punctation usually moderately marked. Color yellowish brown to dark brown, usually uniform across body regions (Fig. 31). Shape of head trapezoid. Eyes relatively small, at most only slightly emarginated anteriorly, usually moderately projected from outline of head. Clypeus trapezoid, with anterior margin from broadly to only slightly emarginate. Labrum fully exposed. Mentum with strong median anterior depression sometimes limited by low transverse carina; surface of mentum mostly smooth and undulated. Antennae with nine antennomeres; cupule slightly asymmetric, with rounded outline. Maxillary palps short, nearly 2/3 width of head, and stout (Fig. 31C); inner margin of maxillary palpomere 2 nearly straight, outer margin strongly curved along apical half. Elytra without sutural striae, and only rarely with impressed striae; ground punctures moderate to sharply marked, uniformly and rather densely distributed; systematic punctures slightly larger and deeper than remainder punctures; serial punctures usually not clearly differentiated; outer margins of elytra only slightly flared (Fig. 31A, D). Prosternum flat, sometimes only slightly elevated along longitudinal midline (Fig. 31C). Posterior elevation of mesoventrite either with transverse ridge, or with well-developed tooth that extends anteriorly as longitudinal carina; anapleural sutures concave, separated at anterior margin by distance nearly 0.3 × anterior margin of mesepisternum. Metaventrite densely pubescent, except for large median teardrop-shaped glabrous patch (Fig. 31C, F); anteromedian area of metaventrite with a deep and narrow transverse depression before anterior intercoxal process. Protibiae with spines of anterior row hair-like, semi erect, relatively long and thick (Fig. 31C). All tarsomeres bearing long apical hair-like setae on dorsal face, and two lateral rows of hair-like spines on ventral face of tarsomeres 2–4. Posterior femora mostly glabrous, with few scattered setae along basal half to basal 2/3, with hydrofuge pubescence along anterodorsal margin (Fig. 31C, F); tibial grooves well-developed, sometimes covered by hydrofuge pubescence. Fifth abdominal ventrite apically truncate, with stout setae. Aedeagus trilobed (Fig. 30F–I), with outer margins convex, straight or sinuate, with basal piece 0.45–0.9 × length of parameres; median lobe somewhat triangular in shape, with well-developed lateral basal apodemes; apex of median lobe widely to narrowly acute, sometimes “pinched”; parameres nearly as long as median lobe; well-developed gonopore, preapically situated.

##### Differential diagnosis.

*Ephydrolithus* can be distinguished from most Neotropical acidocerines by their mostly glabrous metafemora. From other genera exhibiting the same condition, such as *Quadriops* (Girón and Short 2017), *Ephydrolithus* can be distinguished by the entire (as opposed to divided; Fig. 11C) eyes; from *Tobochares* (Kohlenberg and Short 2017), *Ephydrolithus* can be differentiated by the number of antennomeres (nine in *Ephydrolithus*, eight in *Tobochares*).

##### Distribution.

**Neotropical**: Brazil (Bahía, Minas Gerais); Fig. 5.

##### Natural history.

All known species are exclusively associated with rock seepages (e.g., Fig. 9; Girón and Short 2019).

##### Larvae.

Immature stages are not known for the genus.

##### Taxonomic history.

*Ephydrolithus* was only recently described.

##### Remarks.

In the etymology section of the original publication, Girón and Short (2019) indicate that the genus name is neuter, which is erroneous. The name is masculine, which is the gender for the Greek word *lithos*, the last component of the genus name. Four species of *Ephydrolithus* have been described until now, all of them from southeastern Brazil.

##### Species examined.

Holotypes and paratypes of all known species were examined for this study. We have also seen specimens of additional undescribed species.

##### Selected references.

Girón and Short 2018: original description of the genus and all its known species; Short et al. 2021: phylogenetic placement.

#### Genus Globulosis

Taxon classificationAnimaliaColeopteraHydrophilidae

García, 2001

**Figure 32.**
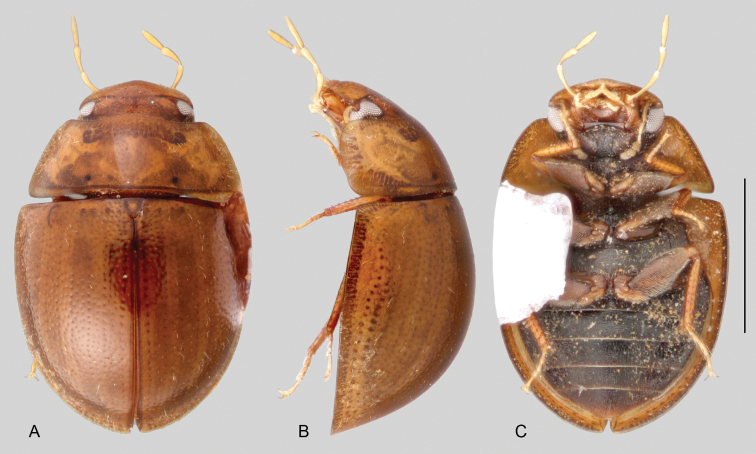
Habitus of *Globulosis flavus***A** dorsal habitus **B** lateral habitus **C** ventral habitus. Scale bar: 1 mm.

Figs 1U , 2 , 5 , 30J , 32

Globulosis García, 2001: 153.

##### Gender.

Masculine.

##### Type species.

*Globulosis hemisphericus* García, 2001: 153; by original designation.

##### Diagnosis.

Small beetles, body length 1.9–2.3 mm. Body shape rounded in dorsal view, strongly convex in lateral view (Fig. 32). Surface of head, pronotum and elytra smooth, with moderate to shallow ground punctation. Coloration yellow to dark brown, uniform along body, with paler mouthparts and tarsi (Fig. 32). Shape of head relatively oval. Eyes relatively small, anteriorly emarginated (Fig. 32B), not projected from outline of head. Clypeus trapezoid, with anterior margin mesally broadly emarginate. Labrum fully exposed. Mentum with anterior depression limited by low transverse carina; surface of mentum only slightly striate. Antennae with eight antennomeres, with cupule only slightly asymmetric and rounded in outline. Maxillary palps slender, slightly shorter than width of head (Fig. 32C). Pronotum evenly convex. Elytra without sutural or other distinct striae, with outer margins slightly flared; elytral ground punctation shallow to moderate, uniformly distributed (Fig. 32). Surface of prosternum flat. Mesoventrite with transverse ridge, usually elevated medially into acute tooth (Fig. 32C); anapleural sutures concave, separated at anterior margin by distance nearly as width of anterior margin of mesepisternum. Metaventrite uniformly covered by hydrofuge pubescence, with small, longitudinal posteromesal glabrous patch, and reduced posterolateral glabrous patches (Fig. 32C). Protibiae with spines of anterior row long, thick, semi erect and sparse; apical spurs of protibiae short and of moderate thickness. Metafemora with moderate tibial grooves; hydrofuge pubescence covering basal 4/5 of anterior surface (Fig. 32C). Tarsomeres 1–4 ventrally with rows of long and thick setae. Metatarsomeres 2–4 gradually decreasing in size, 5 nearly as long as 2–4 combined. Fifth abdominal ventrite with small truncation at apex, with fringe of flat and stout setae. Aedeagus trilobed (Fig. 30J); with short basal piece, less than 1/3 length of parameres; median lobe wider than width of parameres; gonopore well differentiated.

##### Differential diagnosis.

*Globulosis* is among the smallest acidocerines. Its small size along with very round and convex body shape, sets it apart from all other acidocerines known to date.

##### Distribution.

**Neotropical**: Brazil (Amazonas, Pará), Colombia, Guyana, Suriname, Venezuela; Fig. 5.

##### Natural history.

The genus is most commonly found along the margins of small, sandy forested streams, especially with vegetated margins. However, a few specimens have been taken in shallow swamps.

##### Larvae.

The immature stages of *Globulosis* remain unknown.

##### Taxonomic history.

García (2001) described the genus with one species, and placed it in its own tribe (Globulosina, now synonymized with Acidocerinae). The genus was revised in 2017 by Short et al., who described one new species and examined new material that greatly expanded the range of the previously known species.

##### Remarks.

There are two described species of *Globulosis*. One female specimen from Colombia has been left unidentified as it could not be reliably assigned to any species. Because of the extremely uniform external morphology in the genus, the male genitalia is the most reliable feature for species recognition. Based on additional material we have examined the genus appears to be more broadly distributed in the Amazon region than as currently published.

##### Species examined.

The holotype, along with several additional specimens of *Globulosis hemisphericus* García, and the holotype and paratypes of *G. flavus* Short, García & Girón were examined in this study.

##### Selected references.

García 2001: genus description, monotypic; Short et al. 2017: description of one new species from Venezuela, range expansion for type species; Short et al. 2021: phylogenetic placement.

#### Genus Helobata

Taxon classificationAnimaliaColeopteraHydrophilidae

Bergroth, 1888

**FIgure 33.**
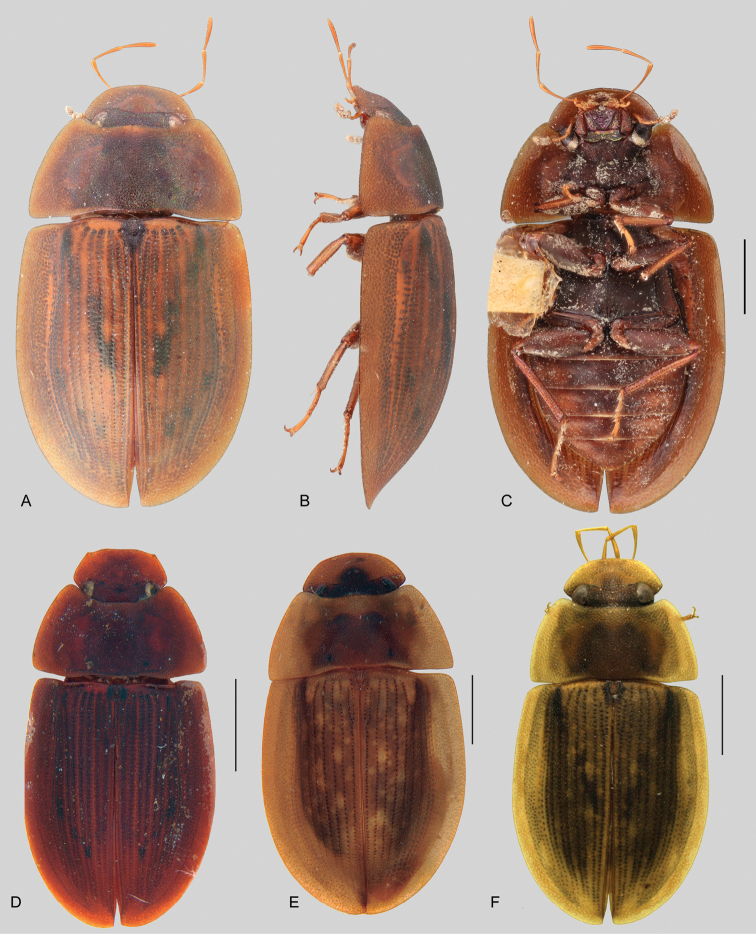
Habitus of *Helobata* spp. **A–C***H. larvalis*: **A** dorsal habitus **B** lateral habitus **C** ventral habitus **D***H. quatipuru* (from Clarkson and Almeida 2018) **E***H. amazonensis* (from Clarkson and Almeida 2018) **F***H. pantaneira* (from Clarkson et al. 2016). Scale bars: 1 mm.

**Figure 34.**
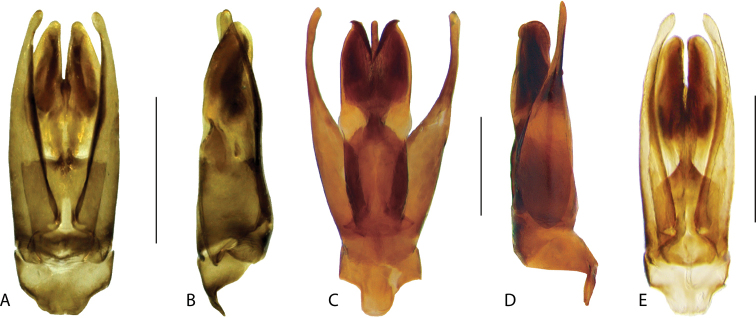
Aedeagi of *Helobata* spp. **A, B***H. pantaneira* (from Clarkson et al. 2016): **A** dorsal view **B** lateral view **C, D***H. quatipuru* (Clarkson and Almeida 2018) **A** dorsal view **D** lateral view **E***H.* sp. (Ecuador), dorsal view. Scale bars: 0.5 mm.

Figs 1J , 2 , 5 , 11L , 33 , 34

Helopeltis Horn, 1873: 137.
Type species: Helopeltis larvalis Horn, 1873: 137; by monotypy.
Helobata Bergroth, 1888: 221 – Replacement name for Helopeltis Horn, 1873.
Helopeltina Cockerell, 1906: 240 – Replacement name for Helopeltis Horn, 1873.
Type species: Helopeltis larvalis Horn, 1873: 137.

##### Gender.

Feminine.

##### Type species.

*Helopeltis larvalis* Horn, 1873: 137; by monotypy.

##### Diagnosis.

Medium sized beetles, body length 4–7 mm. Body shape oval in dorsal view, dorsoventrally flattened, with dorsal outline nearly straight along medial third in lateral view (Fig. 33); surface even and granulate. From yellowish, orange brown to dark brown in coloration, usually with patterns along elytra, with different areas of head and pronotum darkened. Shape of head somewhat trapezoid (Fig. 11L). Anterior corners of frons extended laterally and posteriorly, emarginating anterior margin of eyes. Eyes of moderate size, somewhat oval, anteriorly deeply emarginated, not projected from outline of head. Clypeus somewhat pentagonal, laterally explanate, with anterior margin usually straight (Fig. 11L). Labrum concealed by clypeus (Fig. 11L). Mentum with surface variably sculptured, usually with oblique and transverse striae (Fig. 33C). Antennae with eight antennomeres, with cupule strongly asymmetric and oval in outline. Maxillary palps slender, slightly longer than greatest width of head; inner margin of maxillary palpomere 2 weakly and evenly curved, and outer margin weakly curved along apical third (Fig. 33C). Pronotum with surface of lateral areas flat. Elytra without sutural striae, with outer margins laterally explanate; serial punctures clearly aligned in longitudinal rows (Fig. 33A). Scutellar shield U-shaped. Surface of prosternum flat, to medially bulging, smooth to irregularly sculptured. Posterior elevation of mesoventrite only weakly bulging, with pair of lateral, longitudinal, low ridges; anapleural sutures nearly parallel along anterior section, separated anteriorly by distance slightly shorter than anterior margin of mesepisternum. Metaventrite uniformly covered by hydrofuge pubescence, with medial, narrow, and slightly carinate glabrous patch; posterolateral glabrous patches reduced. Protibiae with spines of anterior row short and semi erect; apical spurs of protibiae reduced, much shorter than protarsomere 1. Metafemora with tibial grooves moderately developed; hydrofuge pubescence covering 5/6 of anterior surface (Fig. 33C). Tarsomeres 1–4 ventrally densely covered by setae; metatarsomere 2 longer than 3 and 4 combined, 1 nearly as long as 3, and 5 nearly as long as 2–4 combined. Fifth abdominal ventrite apically emarginate, with fringe of flat and stout setae. Aedeagus divided (Fig. 34), parameres separated from each other for most of their lengths; median lobe divided in dorsal and ventral plates; dorsal plate usually strongly sclerotized; ventral plate bilaterally bifurcated, forming thick lateral lobes along apical region; basal piece nearly 0.2 × the length of parameres, always noticeable; gonopore not clearly visible.

##### Differential diagnosis.

*Helobata* is one of the most conspicuous genera of acidocerines, especially in the New World. The flattened and broadly explanate body shape and concealed labrum, accompanied by granulose surface, long and slender maxillary palps and well-defined elytral serial punctures, are quite unique in the subfamily. The only genus that shares some of these features is *Helopeltarium*, except that the latter has short maxillary palps, smooth surface and lacks serial punctures along the elytra. The configuration of the aedeagus (Fig. 34), in particular the thickness of the lateral lobes of the ventral plate of the median lobe, is also unique among acidocerines.

##### Distribution.

**Nearctic**: United States (California, Florida, Louisiana, Mississippi, North Carolina, South Carolina, Texas, Virginia). **Neotropical**: Argentina, Bolivia, Brazil (Amazonas, Ceará, Mato Grosso, Mato Grosso do Sul, Pará, Rio de Janeiro, Roraima), Cuba, Guatemala, Mexico, Paraguay, Suriname, Venezuela; Fig. 5.

##### Natural history.

Species of *Helobata* occur primarily in open habitats with abundant vegetation. According to Clarkson et al. (2016), specimens of *Helobata* are uncommonly encountered and occur in marshes, swamps, and ponds, most often in small numbers, although they are rarely found in modest amounts (dozens of individuals; Short, pers. obs.). According to Archangelsky (1997), they can be found in slow moving creeks or rivers, living among the littoral vegetation or on floating plants. They are attracted to lights. Females have been observed carrying their egg cases attached to the ventral side of their abdominal ventrites (Archangelsky 1997).

##### Larvae.

The larva (first instar) and egg case are only known for *Helobata larvalis*; these immature stages were described by Spangler and Cross (1972). A differential diagnosis of the first instar larva was provided by Fikáček (2003).

##### Taxonomic history.

This genus was described by Horn (1873) under the name *Helopeltis*, which was preoccupied by *Helopeltis* Signoret, 1858 (Hemiptera). Bergroth (1888) proposed the name *Helobata* as a replacement name for *Helopeltis* Horn, whereas Cockerell (1906a) proposed the name *Helopeltina*. *Helobata* has priority, so it is the currently valid name for the genus, which was revised by Fernández and Bachmann (1987).

##### Remarks.

There are 13 species of *Helobata* described to date. The type species, *Helobata larvalis* (Horn), has generally been known under the name *Helobata striata* (originally published as *Hydrophilus striatus* Brullé, 1841: 58, which is a primary homonym of *Hydrophilus striatus* Say, 1825 [now *Berosus striatus* (Say)]; therefore unavailable. The name *Helobata larvalis* (Horn) was then reinstated by Hansen (1991: 293). Photos of a syntype of *Helopeltis larvalis* (Horn) are available at https://mczbase.mcz.harvard.edu/guid/MCZ:Ent:101 (accessed 9 January 2021). The external morphology of members of *Helobata* is very homogeneous. Some variation can be observed in the shape of the clypeus (e.g., Fernández 1987; Clarkson et al. 2016). *Helobata* is the only Neotropical genus truly widespread in the New World, as it ranges from southeastern North America, all the way to Argentina and Southern Brazil.

##### Species examined.

*Helobata cuivaum* García (paratype), *H. larvalis* (Horn), and *H. lilianae* García (paratype).

##### Selected references.

Horn 1873: original description of the genus and the type species; Spangler and Cross 1972: description of egg case and first instar larva; Fernández and Bachmann 1987: review of the genus, description of four new species from Argentina, Brazil and Paraguay; García 2000: three new species from Venezuela; Makhan 2007: two new species from Suriname; Clarkson et al. 2016: two new species from Brazil, review and new country records of Brazilian species; Clarkson and Almeida 2018: new records from Brazil; Short et al. 2021: phylogenetic placement.

#### Genus Helochares

Taxon classificationAnimaliaColeopteraHydrophilidae

Mulsant, 1844

**Figure 35.**
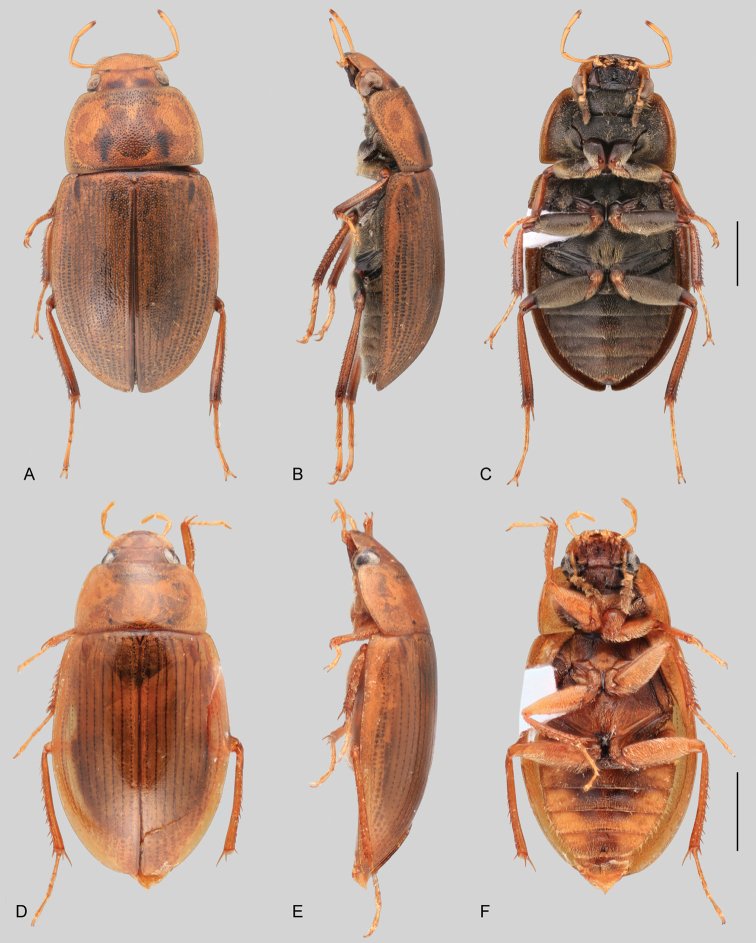
Habitus of *Helochares* spp. **A–C***Helochares tristis*: **A** dorsal habitus **B** lateral habitus **C** ventral habitus **D–F***H. sharpi*: **D** dorsal habitus **E** lateral habitus **F** ventral habitus. Scale bar: 1 mm.

**Figure 36.**
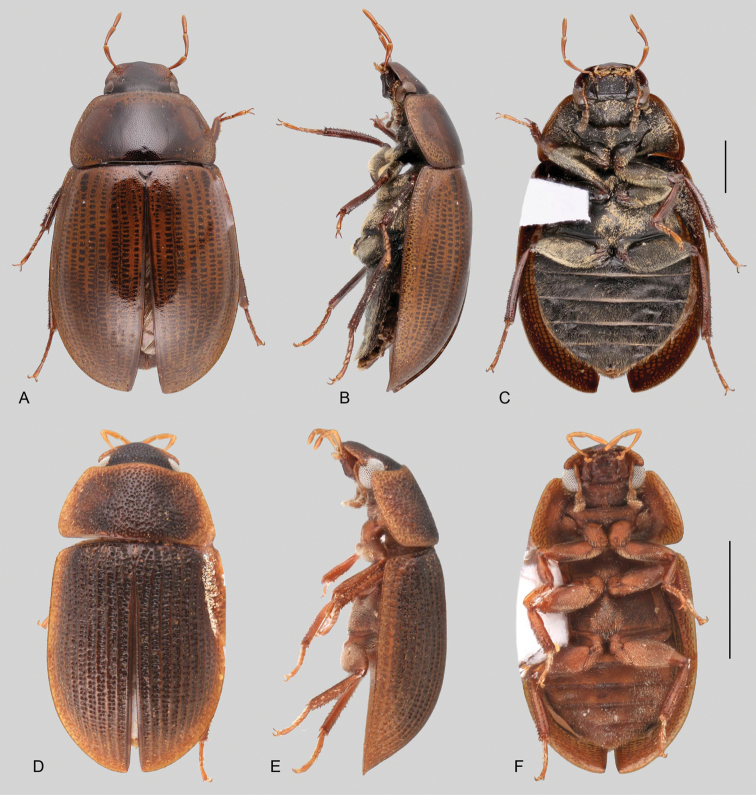
Habitus of *Helochares* spp. **A–C***H. laevis*: **A** dorsal habitus **B** lateral habitus **C** ventral habitus **D–F***H.* sp. (India, Goa): **D** dorsal habitus **E** lateral habitus **F** ventral habitus. Scale bar: 1 mm.

**Figure 37.**
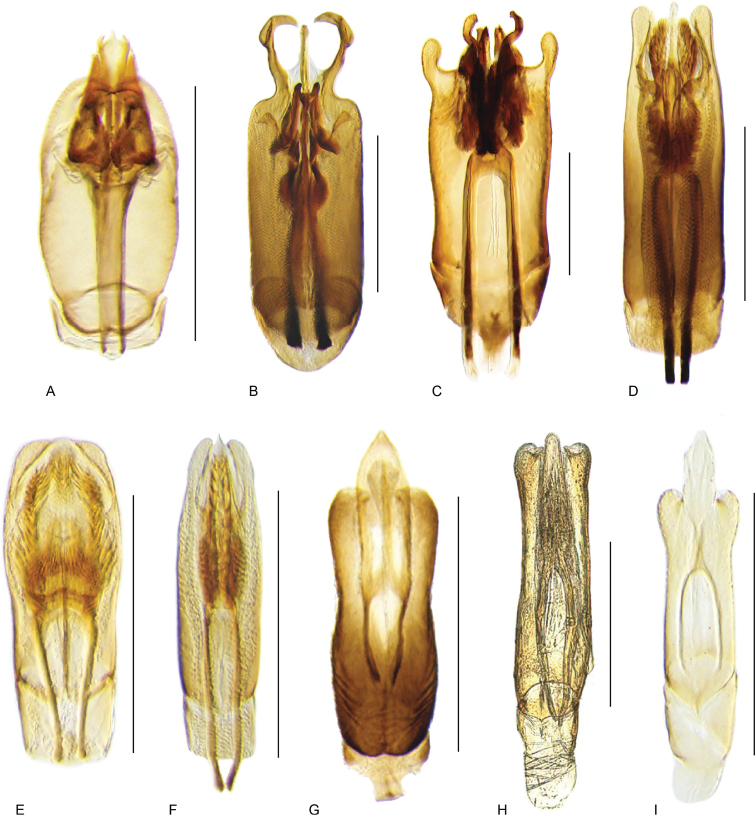
Aedeagi **A–H***Helochares* spp.: **A***H.* sp. (Guinea) **B***H. tristis***C**H. nr. cresphontes**D**H. nr. tatei**E***H.* sp. (India, Goa) **F***H.* sp. (Vietnam) **G***H. politus***H***H. songi* (from Jia and Tang 2018, fig. 48) **I***Helopeltarium ferrugineum*. Scale bars: 0.5 mm.

Figs 1E, F , 2 , 5 , 11F , 35 , 36 , 37A–H

Helophilus Mulsant, 1844a: 132 [rejected name no. 1707 (ICZN 1964, Opinion 710)].
Helochares Mulsant, 1844a: 197; replacement name for Helophilus Mulsant, 1844a: 132; official name no. 1601 (ICZN 1964, Opinion 710).
Enhydrus Dahl 1823: 34 [nomen nudum; rejected name no. 1705 (ICZN 1964, Opinion 710)].
Enhydrus MacLeay, 1825: 35 [rejected name no. 1704 (ICZN 1964, Opinion 710)].
Pylophilus Motschulsky, 1845: 32.
Type species: Hydrophilus griseus Fabricius, 1787: 189; fixed by monotypy = Dytiscus lividus Forster, 1771.
Peloxenus Motschulsky, 1845: 549; replacement name for Pylophilus Motschulsky, 1845.
Helophygas Motschulsky, 1853: 11 [rejected name no. 1708 (ICZN 1964, Opinion 710)].
Helocharis Thomson, 1859: 18 [incorrect subsequent spelling].
Hydrobaticus MacLeay, 1871: 131, syn. nov.
Type species: Hydrobaticus tristis MacLeay, 1871: 131; by subsequent designation by d’Orchymont (1943a: 2); originally described as genus; downgraded to subgenus of Helochares by d’Orchymont (1919c: 148).
Helocharimorphus Kuwert, 1890: 306, syn. nov.
Type species: Helocharimorphus sharpi Kuwert, 1890: 307; by monotypy; originally described as genus; downgraded to subgenus of Helochares by d’Orchymont (1919c: 148).
Graphelochares Kuwert, 1890: 38.
Type species: Helophilus melanophthalmus Mulsant, 1844a: 137; by monotypy.
Grapidelochares Ganglbauer, 1904: 248; [unjustified emendation of Graphelochares Kuwert, 1890].

##### Gender.

Masculine.

##### Type species.

*Dytiscus lividus* Forster, 1771: 52; by subsequent designation (Thomson 1859: 18).

##### Diagnosis.

Small to medium sized beetles, body length 2–7 mm. Body shape oval in dorsal view; slightly to moderately convex in lateral view, with dorsal outline nearly flat along anterior half of elytra, or somewhat evenly curved (Figs 35, 36). Coloration usually yellowish brown, sometimes orange brown, pale brown to medium brown; ground punctation shallow (e.g., Fig. 35D) to strongly marked (e.g., Fig. 36D). Shape of head trapezoid to oval (e.g., Fig. 11F). Eyes medium sized to large, not or moderately emarginated anteriorly, usually projected from outline of head. Clypeus trapezoid, with anterior margin broadly and roundly emarginate; sometimes lateral margins of clypeus slightly bent upwards. Labrum fully exposed. Mentum rather flat, sparsely punctate, coarsely to shallowly, rarely striate (e.g., Figs 35C, 36C); median anterior depression of mentum relatively shallow; submentum shallowly punctate to smooth. Antennae with nine antennomeres; cupule strongly asymmetric, with rounded outline; antennomere 9 slightly, to 3 × longer than antennomere 7. Maxillary palps slender, moderately long, 0.6–1.2 × the width of head (e.g., Figs 35C, 36C); inner margin of maxillary palpomere 2 weakly and evenly curved to nearly straight, outer margin evenly curved to curved along apical 2/3; maxillary palpomere 3 slightly longer than 4. Prosternum flat to medially bulging to tectiform. Elytra without sutural striae, with ground punctures usually moderately marked; often with serial punctures forming ten longitudinal rows along elytra (e.g., Fig. 35A). Posterior elevation of mesoventrite, flat to simply bulging (e.g., Fig. 35C); bulge usually with long fine setae; anapleural sutures strongly concave, nearly parallel along anterior section, separated anteriorly by distance 0.6–1.0 × anterior margin of mesepisternum. Metaventrite densely covered by hydrofuge pubescence, without glabrous patches (e.g., Figs 35C, 36C). Protibiae with spines of anterior row either nearly absent (e.g., Fig. 35C) or as long thick semi-erect setae. Metafemora with tibial grooves weakly developed to absent; hydrofuge pubescence covering basal 6/7 of anterior surface. Tarsomeres 1–4 with pair of lateral rows of long fine spines on ventral face, sometimes ventral face densely covered by hair-like spines; tarsomere 5 with medial row of long fine spines; metatarsomeres variable in proportions (2–4 gradually decreasing in size with 5 nearly as long as 3 and 4 combined; 2 and 5 similar in length, each slightly longer than 3 and 4 combined). Fifth abdominal ventrite apically emarginate, with fringe of stout setae. Aedeagus tubular (Fig. 37A–H); parameres fused to each other for most of their lengths, with apex either simple or bifurcate/bilobate; median lobe with very long basal apodemes (as long or longer than main piece of median lobe), often extending beyond base of parameres in repose; median lobe either simple (without subdivisions), or with multiple and different kinds of sclerotizations of inner membranes; basal piece usually much shorter than parameres; gonopore of variable development, usually visible when median lobe is simple.

##### Differential diagnosis.

In the present definition, most species of *Helochares* are yellowish to brown in coloration, ranging in size from 2–7 mm (e.g., Figs 35, 36), usually moderately punctate throughout the dorsal surface, and most diverse in the Old World. Smaller members of the genus may be confused with *Agraphydrus*, from which *Helochares* can be distinguished by its uniformly pubescent metaventrite (e.g., 36C, F; *Agraphydrus* bears a distinct posteromedian glabrous patch on the metaventrite, e.g., Fig. 18F, I). From *Peltochares*, and *Novochares*, members of *Helochares* can be distinguished by their shorter and relatively stout maxillary palps [0.6–1.2 × the width of the head in *Helochares* (e.g., Fig. 35C), as opposed to slender, 1.3–1.8 × in *Peltochares* (e.g., Fig. 44C, F), 1.1–1.5 × in *Novochares* (e.g., Fig. 42C, F)]; and by the development of the tibial grooves (weakly developed to absent in *Helochares*, well developed in both *Novochares* and *Peltochares*). The most problematic species would be those that are dark brown, relatively flattened, highly polished, and 4–5 mm long. In those cases, the most reliable feature for identification would be the male genitalia: *Helochares* has tubular aedeagi (e.g., Figs 16E, F, 37A–H), *Peltochares* has spiked aedeagi (e.g., Figs 16C, D, 45), and *Novochares* has divided aedeagi (e.g., Figs 16G, H, 43); see explanation of aedeagal types under the aedeagus section of Morphological variation in Acidocerinae and its taxonomic importance).

##### Distribution.

**Afrotropical**: Angola, Benin, Botswana, Burkina Faso, Burundi, Cameroon, Chad, Democratic Republic of the Congo, Eritrea, Ethiopia, Gabon, Gambia, Ghana, Guinea, Guinea Bissau, Ivory Coast, Kenya, Liberia, Madagascar, Malawi, Mali, Mauritania, Mauritius (incl. Mascarene Is., Rodrigues), Morocco [in doubt], Mozambique, Namibia, Niger, Nigeria, Oman, Republic of the Congo, Réunion, Rwanda, São Tomé and Príncipe, Saudi Arabia, Senegal, Seychelles (incl. Aldabra), Sierra Leone, Republic of South Africa, South Sudan, Sudan, Tanzania, Togo, Uganda, United Arab Emirates, Yemen (incl. Socotra), Zambia, Zimbabwe. **Australasian**: Australia (Australian Capital Territory, New South Wales, Northern Territory, Queensland, South Australia, Tasmania, Victoria, Western Australia), Fiji, Papua New Guinea (incl. Duke of York), Vanuatu. **Indo-Malayan**: Bangladesh, Burma, Cambodia, China (Fujian, Guangdong, Guangxi, Guizhou, Hainan, Hong Kong, Hunan, Jiangxi, Macao, Taiwan, Yunnan, Zhejiang), India (Andaman Is., Assam, Bihar, Karnataka, Madhya Pradesh, Nicobar Is., Uttarakhand, Uttar Pradesh, Tamil Nadu, West Bengal), Indonesia (Bali, Borneo, Java, Lombok, Papua, Sumatra), Laos, Malaysia (Peninsula, Sabah), Nepal, Philippines (Manila), Singapore, Sri Lanka, Thailand, Vietnam. **Nearctic**: U.S.A. (Alabama, Arkansas, Arizona, California, Delaware, District of Columbia, Florida, Georgia, Illinois, Indiana, Iowa, Kansas, Kentucky, Louisiana, Maryland, Mississippi, Missouri, Nevada, North Carolina, North Carolina, Ohio, Oklahoma, Oregon, Pennsylvania, South Carolina, Tennessee, Texas, Virginia). **Neotropical**: Costa Rica, Ecuador, El Salvador, Guatemala, Honduras, Mexico, Nicaragua, Panama, Peru, Venezuela. **Oceanian**: Samoa, Tonga. **Palearctic**: Algeria, Austria, Azerbaijan, Belarus, Bosnia Herzegovina, Bulgaria, Canary Islands, China (Chongqing, Jilin, Hubei, Jiangsu, Shanghai, Shaanxi, Sichuan, Xinjiang, Xizang [Tibet]), Croatia, Czech Republic, Denmark, Egypt, Estonia, Finland, France, Germany, Georgia, Great Britain, Greece, Hungary, Iran, Iraq, Ireland, Israel, Italy, Japan, Latvia, Lebanon, Lithuania, Luxembourg, Macedonia, Morocco, Netherlands, Norway, Pakistan, Poland, Portugal, Russia, Serbia and Montenegro, Slovakia, Slovenia, South Korea, Spain, Sweden, Switzerland, Syria, Tunisia, Turkey, Ukraine; Fig. 5.

##### Natural history.

Most of the older descriptions have no associated ecological information. Species of *Helochares* are aquatic (Hansen 1991) with a preference for quiet bodies of water (Archangelsky 1997) or slow flowing streams, rivers or pools, with pebbles, and mossy stones (Dong and Bian 2021); some species have been collected in rivers, streams, ponds, stagnant water, along sides of rivers, forest pool margins, usually associated with live or decomposing floating vegetation. They can be occasionally collected at light, sometimes in large numbers (Jia and Tang 2018). Females have been observed carrying their egg cases attached to the ventral side of their abdomen.

##### Larvae.

Anderson (1976) described the immature stages of *Helochares tristis* (MacLeay) along with the breeding method he used; the author described the eggs, egg case (25–50 eggs per case), first, second, and third instar larvae and pupa, as well as the entire life cycle. Anderson (1976) recorded observations of the emergence of larvae and adults. As the females carry their eggs attached to the ventral side of their bodies, Anderson (1976: 222) noted: “When hatching from an attached bag, larvae appeared to emerge into the ventral bubble of air. Larvae then rose to the surface of the water and swam away with an alternate head-to-tail movement. They were observed to have bubbles of air in the abdomen. No doubt this was taken from the ventral air bubble and enabled the larvae to become buoyant.” According to Archangelsky (1997) the larvae are predatory and also cannibalistic.

A diagnosis for larvae of *Helochares* as well as a list of the described immatures are provided in Fikáček (2003), at the time considering *Helochares* sensu Hansen (1991), including species of *Novochares* and *Peltochares*; the known larvae of the redefined *Helochares* are *H. lividus* (Forster) (unknown stage larva in d’Orchymont 1913b; first, second and third instar larvae in Panzera 1932), *H. maculicollis* Mulsant (eggs, first and third instar larvae and pupa in Richmond 1920), *H. obscurus* (Müller) (first, second and third instar larvae in Panzera 1932, as *H. griseus*], *H. tristis* (MacLeay) (eggs, first, second and third instar larvae, and pupa in Anderson 1976), *H. clypeatus* (Blackburn) (third instar larva in Watts 2002), *H. luridus* (MacLeay) (third instar larva in Watts 2002), *H. tenuistriatus* Régimbart (third instar larva in Watts 2002). Minoshima and Hayashi (2011) described *H. anchoralis* Sharp (first instar larva), *H. nipponicus* Hebauer (first, second and third instar larvae), and *H. pallens* (MacLeay) (first, second and third instar larvae); Table 3.

##### Taxonomic history.

The genus was originally described under the name of *Helophilus*, which was preoccupied by *Helophilus* Leach, 1817 (Diptera), therefore *Helochares* was proposed by Mulsant (1844) as a replacement name. Thomson, in 1859, designated the type species for the genus. Through time *Helochares*, as well as some of its species, have accumulated multiple synonyms. In 1919, d’Orchymont recognized five subgenera within *Helochares*: *Helochares*, *Chasmogenus*, *Helocharimorphus*, *Hydrobaticus*, and *Sindolus*. *Chasmogenus* was recognized as a separate genus by Fernández (1986). Hansen (1991) added *Batochares* as a subgenus of *Helochares* and commented on the possibility that the recognized subgenera of *Helochares* at the time, represented actually distinct genera. Short et al. (2021) elevated *Batochares* and *Sindolus* to generic status based on their molecular phylogeny, as they were found to indeed represent separate clades. Additionally, Short et al. (2021) found that the type species of *Helochares* (*Helochares lividus* (Forster), which is from the Palearctic region) and the type species of *Hydrobaticus* (*Helochares tristis* (MacLeay) from Australia) are actually relatively closely related and belong in the same subclade (Clade A3 in Short et al. 2021). Furthermore, both species share morphological details of the male genitalia, therefore, we synonymize *Hydrobaticus* syn. nov. with *Helochares*. Conversely, the morphological variation under the new concept of *Helochares* encompasses the features that were used for recognizing *Helocharimorphus*: lack of elytral striae, short maxillary palps, mesoventrite only slightly elevated in front of the mesocoxae, and metatibiae slightly curved (d’Orchymont 1919c: 149, in key). In contrast, more distinct and divergent morphotypes [e.g., small size (nearly 3 mm); strongly punctate surface; emarginated eyes; clypeus laterally bent upwards; Fig. 36D–F] are nested within the main *Helochares* clade. Therefore, despite not knowing the configuration of the aedeagus, we synonymize *Helocharimorphus* syn. nov. with *Helochares*.

While the newly defined concept of *Helochares* is strongly supported as monophyletic (Short et al. 2021), it is a relatively ancient lineage (more than 100 mya) that has accumulated significant morphological variation and deep phylogenetic structure. Short et al. (2021) recovered three strongly supported clades (named A1, A2, and A3), though the relationships among the clades were indecisive among analyses. These clades could potentially serve as a basis for future subgenera. Clade A1 comprises at least two currently described species (*H. fuliginosus* and *H. songi*) from southeast Asia that have a tubular form of the aedeagus (Fig. 37H for *H. songi*), although there appear to be additional undescribed species in the region. Clade A2, which is relatively similar in morphology to Clade A1, comprises all New World species that remain assigned to *Helochares*, also with a similar tubular aedeagal form (Fig. 37G for *H. politus*); this lineage was recently revised by Short and Girón (2017). All remaining species fall in Clade A3, which even in this reduced form contains tremendous morphological diversity (Fig. 37A–F). More study is needed for the genus as a whole, and in particular Clade A3, to further refine its classification and reintroduce species groups and subgenera. It is likely that features of the male genitalia will continue to prove useful in any refined classification of the genus.

##### Remarks.

*Helochares* has been generally considered the most diverse, most widespread, and most taxonomically challenging genus of acidocerines. Even after the removal of unrelated lineages by Short et al. (2021), there remain 159 described species of *Helochares*, although *Agraphydrus* has now eclipsed *Helochares* as the largest genus, with 201 described species. Efforts have been made to try to make sense of such diversity, by studying local faunas (Hansen 1982; Watts 1995; Hebauer 1996; Short and Girón 2017; Jia and Tang 2018), but traditional character systems used for classification have been inadequate for distinguishing monophyletic groups. Only now, after the phylogenetic study by Short et al. (2021), there is some clarity regarding morphological trends in the genus. Most of the representative specimens available for this study are card-mounted, therefore characters of the ventral surfaces in the diagnosis offered here, are based on observations made on a sample of pin-mounted specimens.

##### Species examined.

*Helochares aethiopicus* d’Orchymont,

*H. anchoralis* Sharp***,

*H. alberti* d’Orchymont,

*H. andreinii* d’Orchymont,

*H. anthonyae* Watts,

*H. balfourbrownei* Hansen,

*H. bohemani* d’Orchymont***,

*H. camerunensis* d’Orchymont,

*H. cancellatus* Hebauer*,

*H. championi* Sharp***,

*H. clypeatus* Blackburn,

*H. conformis* Hebauer*,

*H. congruens* d’Orchymont,

*H. crenatostriatus* Régimbart,

*H. crenatuloides* d’Orchymont***,

*H. crepitus* Balfour-Browne,

*H. crispus* d’Orchymont,

*H. densepunctus* Régimbart,

*H. densus* Sharp,

*H. depactus* d’Orchymont,

*H. didymus* d’Orchymont,

*H. difficilis* d’Orchymont,

*H. dilutus* Erichson***,

*H. dimorphus* d’Orchymont,

*H. dollmani* Balfour-Browne,

*H. dolus* d’Orchymont,

*H. egregius* Balfour-Browne,

*H. endroedyi* Hebauer*,

*H. fratris* Hebauer*,

*H. fuliginosus* d’Orchymont,

*H. insolitus* d’Orchymont,

*H. itylus* Balfour-Browne,

*H. ivani* Hebauer*,

*H. laevis* Short & Girón**,

*H. lentus* Sharp,

*H. lepidus* d’Orchymont,

*H. leptinus* d’Orchymont,

*H. lividoides* Hansen & Hebauer,

*H. lividus* (Forster),

*H. loticus* Hebauer*,

*H. luridus* (MacLeay),

*H. maculicollis* Mulsant,

*H. mecarus* d’Orchymont,

*H. mediastinus* d’Orchymont,

*H. melanophthalmus* (Mulsant),

*H. mentinotus* Kuwert,

*H. mersus* d’Orchymont,

*H. minax* d’Orchymont,

*H. minor* d’Orchymont,

*H. minusculus* d’Orchymont,

*H. nebridius* d’Orchymont,

*H. negatus* Hebauer*,

*H. neglectus* (Hope)***,

*H. nexus* Short & Girón**,

*H. nigrifrons* Brancsik,

*H. nigripalpis* Hebauer & Hendrich*,

*H. nigroseriatus* Hebauer*,

*H. nipponicus* Hebauer***,

*H. normatus* (LeConte),

*H. obscurus* (Müller)***,

*H. pallens* (MacLeay)***,

*H. percyi* Watts,

*H. perminutus* Hebauer,

*H. politus* Short & Girón**,

*H. punctatus* Sharp,

*H. salvazai* d’Orchymont,

*H. schwendingeri* Hebauer,

*H. scitulus* Balfour-Browne,

*H. sharpi* (Kuwert)***,

*H. skalei* Hebauer,

*H. steffani* Hebauer*,

*H. stenius* d’Orchymont,

*H. striatus* Boheman,

*H. strictus* d’Orchymont,

*H. strigellus* Hebauer*,

*H. structus* d’Orchymont,

*H. subtilis* d’Orchymont,

*H. tatei* (Blackburn)***,

*H. tenuistriatus* Régimbart,

*H. tristis* (MacLeay)***,

*H. trujillo* Short & Girón**,

*H. wagneri* Hebauer*,

*H. wattsi* Hebauer & Hendrich*,

*H. yangae* Hebauer, Hendrich & Balke*,

*H. zamora* Short & Girón**.

For species marked with one asterisk (*) at least one paratype was available. For species marked with two asterisks (**) the holotype, and in some cases paratypes were examined in this study; all these specimens were card-mounted. For species marked with three asterisks (***) some specimens were pin-mounted, allowing to view ventral structures. For *H. championi* Sharp one of the available specimens was previously compared with the holotype by A. Short.

##### Selected references.

d’Orchymont 1939b, 1943a, c, e: miscellaneous taxonomic works focused on *Helochares*, for the most part describing new species, some of which include aedeagal illustrations; Hansen 1982: notes on European species with morphological clarifications; Hansen 1991: generic diagnosis, synonyms, list of subgenera; Watts 1995: faunistic study for Australia; Hebauer 1996: faunistic study for Africa; Short and Girón 2017: faunistic study for the New World; Jia and Tang 2018: faunistic study for China; Short et al. 2021: phylogenetic placement and main clades within genus.

#### Genus Helopeltarium

Taxon classificationAnimaliaColeopteraHydrophilidae

d’Orchymont, 1943

**Figure 38.**
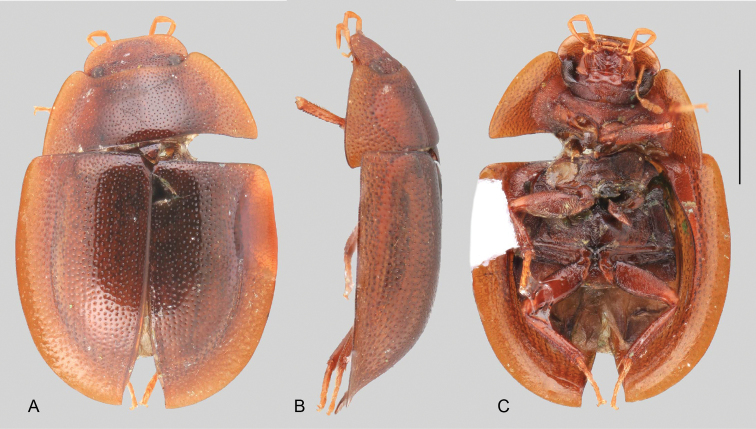
Habitus of *Helopeltarium ferrugineum***A** dorsal habitus **B** lateral habitus **C** ventral habitus. Scale bar: 1 mm.

Figs 1H , 2 , 5 , 37I , 38

Helopeltarium d’Orchymont, 1943f: 9.

##### Gender.

Masculine.

##### Type species.

*Helopeltarium ferrugineum* d’Orchymont, 1943f: 10; by original designation and monotypy.

##### Diagnosis.

Small beetles, body length nearly 3.5 mm. Body broadly oval and explanate in dorsal view, rather flat in lateral view, with dorsal outline nearly straight along median region (Fig. 38). Surface smooth (without granulations or reticulations), with ground punctation strongly marked. Body orange brown, slightly paler along margins (Fig. 38). Shape of head somewhat trapezoid. Anterior corners of frons extended laterally and posteriorly, emarginating anterior margin of eyes. Eyes relatively small, with anterior margin markedly emarginate in lateral view, in dorsal view not projecting from outline of head. Clypeus laterally expanded in front of eyes; anterior margin of clypeus slightly emarginate. Labrum concealed under clypeus. Mentum with surface obliquely striate (Fig. 38C). Antennae with nine antennomeres, cupule strongly asymmetric, with rounded outline. Maxillary palps short and moderately stout, hardly 3/4 as long as width of head; maxillary palpomere 4 nearly as long as palpomere 3; inner margin of maxillary palpomere 2 nearly straight, outer margin curved along apical half (Fig. 38C). Elytra without sutural striae, broadly explanate laterally, serial punctures absent, ground punctures sharply marked, densely and uniformly distributed (Fig. 38A). Prosternum slightly convex, not carinate medially (Fig. 38C). Posterior elevation of mesoventrite only bulging (Fig. 38C); anapleural sutures only slightly concave, separated at anterior margin by distance similar to anterior margin of mesepisternum. Metaventrite uniformly covered by hydrofuge pubescence (Fig. 38C). Protibiae with spines of anterior row long, thick, and semi-erect; apical spurs of protibiae stout, extending to apex of protarsomere 2. Metafemora without distinct tibial grooves; hydrofuge pubescence covering basal 3/4 of anterior surface of metafemora (Fig. 38C). Tarsomeres 2–4 ventrally densely covered by setae; metatarsomere 1 much shorter than 2; metatarsomere 5 nearly as long as metatarsomere 2 or 3 and 4 combined. Fifth abdominal ventrite apically emarginate, with fringe of flat and stout setae. Aedeagus tubular (Fig. 37I); distal region of each paramere diverging; apex of parameres rounded; basal piece nearly half as long as parameres; median lobe broad, apically tapering to rounded tip; gonopore not clearly visible.

##### Differential diagnosis.

*Helopeltarium* has a very unique appearance within acidocerines. The flattened and broadly explanate body shape and concealed labrum, accompanied by smooth surface, short and stout maxillary palps, lacking elytral serial punctures is unique in the subfamily. It may appear like a very small *Helobata*, but besides geographic origin, the lack of serial punctures, smooth surface and short maxillary palps sets *Helopeltarium* apart very easily. The configuration of the aedeagus in *Helopeltarium*, is very similar to that of some *Helochares*, but the external morphology alone allows for its immediate recognition.

##### Distribution.

**Indo-Malayan**: Myanmar (formerly Burma); Fig. 5.

##### Natural history.

There is no natural history information available for the genus.

##### Larvae.

Immature stages are not known for *Helopeltarium*.

##### Taxonomic history.

Originally described by d’Orchymont (1943f: 9). Redescribed by Hansen (1991: 149).

##### Remarks.

In the original description, d’Orchymont (1943f) compared *Helopeltarium* with *Helobata*. As far as we know, the genus is only known from two syntype specimens of the only known species. This genus was not included in the molecular phylogeny by Short et al. (2021). Its assignment to the *Helochares* group is primarily based on the form of the aedeagus, as well as its distribution in the Old World. Indeed, the genitalia is very similar to those found in some clades of *Helochares*, and it would not be surprising to us if *Helopelatarium* is eventually found to be sister to or nested within *Helochares*.

##### Species examined.

Syntypes of *Helopeltarium ferrugineum* d’Orchymont.

##### Selected references.

d’Orchymont 1943f: 9: original description; Hansen 1991: 149: redescription; Short et al. 2021: phylogenetic position and affinities discussed.

#### Genus Katasophistes

Taxon classificationAnimaliaColeopteraHydrophilidae

Girón & Short, 2018

**Figure 39.**
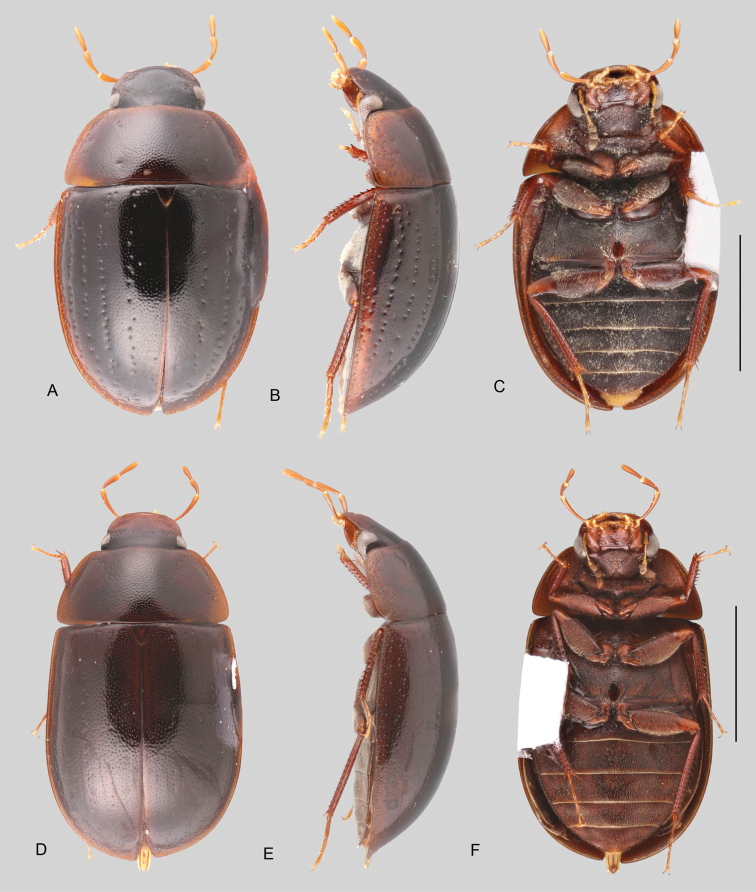
Habitus of *Katasophistes* spp. **A–C***K. merida*: **A** dorsal habitus **B** lateral habitus **C** ventral habitus **D–F***K. superficialis*: **D** dorsal habitus **E** lateral habitus **F** ventral habitus. Scale bars: 1 mm.

**Figure 40.**
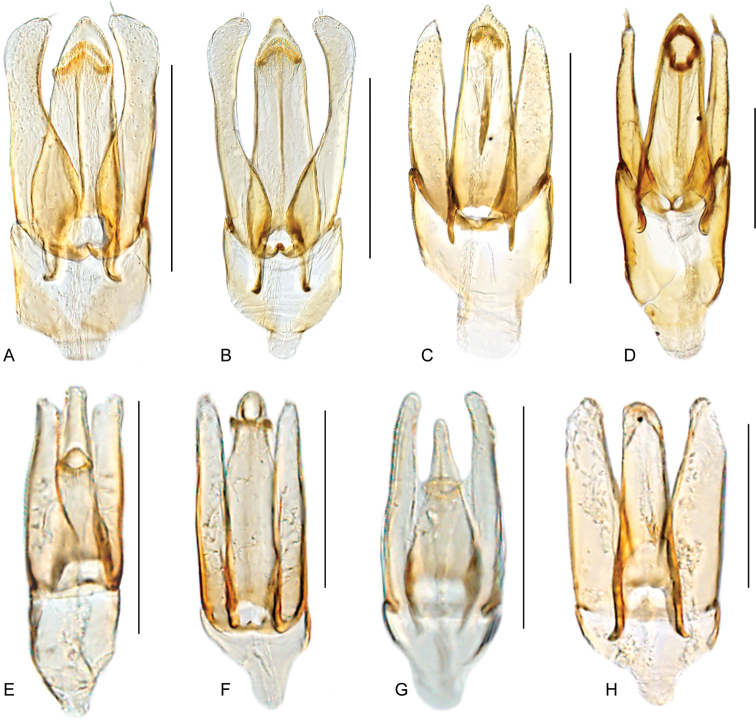
Aedeagi of *Katasophistes* and *Nanosaphes* spp. **A***K. charynae***B***K. cuzco***C***K. merida***D***K. superficialis***E***N. tricolor***F***N. hesperus***G***N. castaneus***H***N. punctatus*. Scale bars: 0.3 mm (**A–C**); 0.1 mm (**E–H**).

Figs 2 , 5 , 39 , 40A–D

Katasophistes Girón & Short, 2018: 132.

##### Gender.

Masculine.

##### Type species.

*Katasophistes merida* Girón & Short, 2018: 136; by original designation.

##### Diagnosis.

Medium to small beetles, body length 2.7–4.5 mm. Body shape oval to elongated in dorsal view; moderately and evenly convex in lateral view (Fig. 39). Color orange brown to dark brown, rather uniform along body regions (Fig. 39). Shape of head trapezoid. Eyes relatively small, subquadrate, at most only slightly emarginated anteriorly, moderately projected from outline of head. Clypeus trapezoid, with anterior margin broadly emarginate. Labrum fully exposed. Mentum with strong median anterior depression sometimes limited by low transverse carina; surface of mentum with lateral oblique ridges (Fig. 39C, F). Antennae with nine antennomeres; cupule slightly asymmetric, with rounded outline. Maxillary palps moderately long, 0.7 × to nearly as long as width of head; inner margin of maxillary palpomere 2 slightly curved near apex, outer margin curved, sometimes strongly, along apical half (Fig. 39C, F). Each elytron with five rows of deep/large systematic punctures; elytra without sutural striae, with outer margins slightly flared; serial punctures absent (Fig. 39A, D). Prosternum slightly convex to tectiform. Posterior elevation of mesoventrite, with a well-defined, curved transverse ridge; anapleural sutures forming an obtuse angle, separated at anterior margin by distance 0.2–0.3 × the width of anterior margin of mesepisternum. Metaventrite densely pubescent, except for large median rhomboid glabrous patch (Fig. 39C, F). Protibiae with spines of anterior row hair-like, semi erect, relatively long and thick. All tarsomeres bearing long apical hair-like setae on dorsal face, and hair-like spines on ventral face of tarsomeres 2–4. Posterior femora glabrous at most along apical third (Fig. 39C, F). Fifth abdominal ventrite apically truncate to slightly emarginate, with fringe of stout setae. Aedeagus trilobed (Fig. 40A–D), nearly parallel sided, with basal piece between 0.5 and 1.1 × length of parameres; median lobe wider than each paramere, gradually narrowing apically, with conspicuous median longitudinal sclerotization, and well-developed lateral basal apodemes; apex of median lobe acute; parameres nearly as long as median lobe, with apical setae; gonopore preapically situated.

##### Differential diagnosis.

At first glance *Katasophistes* may appear similar to some species of *Chasmogenus*, however the lack of sutural striae easily separates the two. The enlargement of the rows of elytral systematic punctures is also rare within the Acidocerinae (found in some *Chasmogenus* and *Agraphydrus*) and will separate it from New World *Helochares*, with which it may also be confused.

##### Distribution.

**Neotropical**: Ecuador, Peru, Venezuela; Fig. 5.

##### Natural history.

One species (*K. merida*) is known from seepages in the Venezuelan Andes. The other described species are known from forested stream pools with abundant detritus in Ecuador and Peru.

##### Larvae.

Immature stages are not known for the genus.

##### Taxonomic history.

*Katasophistes* was only recently described.

##### Remarks.

There are four known species of *Katasophistes*, all of them from Andean or Andean-adjacent localities.

##### Species examined.

Holotypes and paratypes of all known species were available for this study.

##### Selected references.

Girón and Short 2018: original description of the genus and all its known species; Short et al. 2021: phylogenetic placement.

#### Genus Nanosaphes

Taxon classificationAnimaliaColeopteraHydrophilidae

Girón & Short, 2018

**Figure 41.**
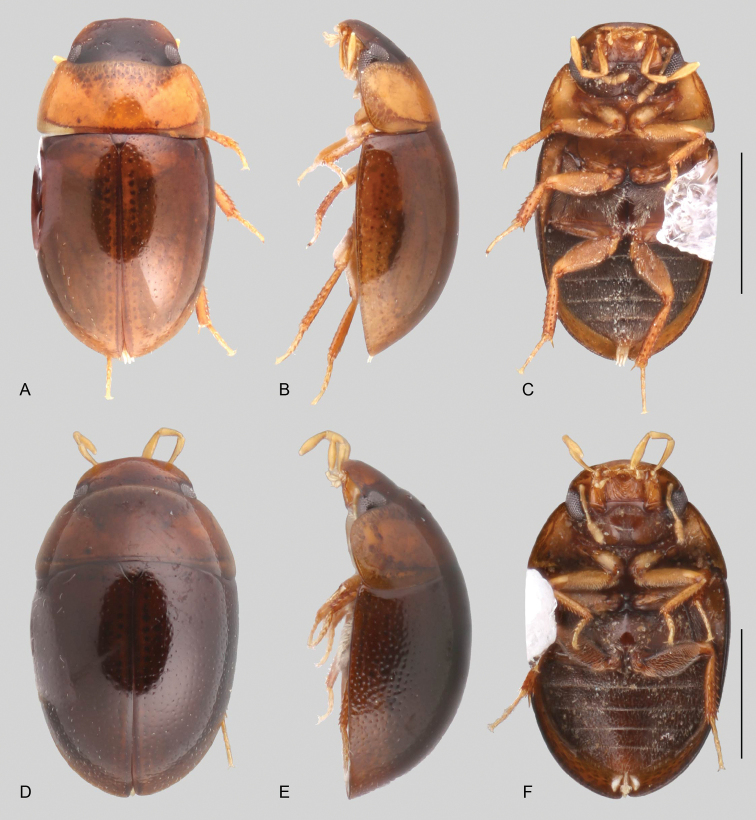
Habitus of *Nanosaphes* spp. **A–C***N. tricolor*: **A** dorsal habitus **B** lateral habitus **C** ventral habitus **D–F***N. punctatus*: **D** dorsal habitus **E** lateral habitus **F** ventral habitus. Scale bars: 0.5 mm.

Figs 1L , 2 , 40E–H , 41

Nanosaphes Girón & Short, 2018: 143.

##### Gender.

Masculine.

##### Type species.

*Nanosaphes tricolor* Girón & Short, 2018: 151; by original designation.

##### Diagnosis.

Very small beetles, body length 1.15–1.45 mm. Body shape oval in dorsal view; slightly to moderately, and evenly convex in lateral view (Fig. 41). Coloration uniformly brown, to variable along the body; ground punctation shallow to moderately marked (Fig. 41). Shape of head trapezoid and relatively wide. Eyes moderate in size, slightly emarginated anteriorly, not projected from outline of head. Clypeus trapezoid, with anterior margin broadly emarginate. Labrum fully exposed. Mentum with lateral oblique ridges. Antennae with eight antennomeres; cupule slightly asymmetric, with rounded outline. Maxillary palps slender, moderately long nearly 0.7 × the width of head; inner margin of maxillary palpomere 2 nearly straight, outer margin curved along apical half (e.g., Fig. 41C, F). Each elytron with ground punctures usually only shallowly marked, seemingly forming longitudinal rows, with irregularly distributed systematic punctures bearing rather long setae, denser along lateral and posterior regions; elytra without sutural striae. Prosternum flat, at most only weakly convex. Posterior elevation of mesoventrite, usually projected as low and short longitudinal carina between mesocoxae; anapleural sutures only weakly curved, separated at anterior margin by distance nearly 0.9 × width of anterior margin of mesepisternum. Metaventrite with posterolateral and mesal glabrous patches (e.g., Fig. 41C, F). Protibiae with spines of anterior row hair-like, semi erect, relatively long, thick and sparse. Metafemora mostly densely covered by hydrofuge pubescence (e.g., Fig. 41C, F). All tarsomeres with long and thick spines on ventral faces of tarsomeres 2–4; metatarsomeres 2–4 gradually decreasing in size, metatarsomere 5 as long as 3 and 4 combined, 2 slightly shorter. Fifth abdominal ventrite apically emarginate, with fringe of stout setae. Aedeagus trilobed (Fig. 40E–H), nearly parallel sided, with basal piece 0.3–0.6 × length of parameres; median lobe with well-developed lateral basal apodemes, wider at base than base of each paramere, usually narrower at apex than preapical width of parameres; apex of median lobe rounded; parameres from slightly shorter to longer than median lobe, and only narrowing at apex; gonopore situated beyond midpoint of median lobe.

##### Differential diagnosis.

The minute size of *Nanosaphes* make them smaller than any other Acidocerinae in the New World, and about equal in size to the smallest species of *Agraphydrus* in the Old World. They are among the smallest water scavenger beetles worldwide. The lack of elytral serial or sutural striae and the antennae with eight antennomeres also separate *Nanosaphes* from all other Neotropical Acidocerinae genera except the co-occurring *Globulosis*. *Nanosaphes* can be easily separated from *Globulosis* by its smaller size and narrower, more parallel sided body form (broader and almost rotund in *Globulosis*, Fig. 32).

##### Distribution.

**Neotropical**: Brazil (Pará), Guyana, Suriname; Fig. 5.

##### Natural history.

Species are associated with stream margins, particularly where there are marginal banks of sand and roots.

##### Larvae.

Immature stages are not known for *Nanosaphes*.

##### Taxonomic history.

*Nanosaphes* was only recently described.

##### Remarks.

There are four known species of *Nanosaphes*, which can be differentiated from each other by external morphological features (e.g., elytral punctation, coloration, shape of the posterior elevation of the mesoventrite), which is somewhat unusual by acidocerine standards. We have seen additional material of *Nanosaphes* from other regions within the Guiana Shield.

##### Species examined.

Holotypes and paratypes of all known species were available for this study.

##### Selected references.

Girón and Short 2018: original description of the genus and all its known species; Short et al. 2021: phylogenetic placement.

#### Genus Novochares

Taxon classificationAnimaliaColeopteraHydrophilidae

Girón & Short gen. nov.

http://zoobank.org/9E46D713-DA7C-46B6-B407-E99C490CFD32

**Figure 42.**
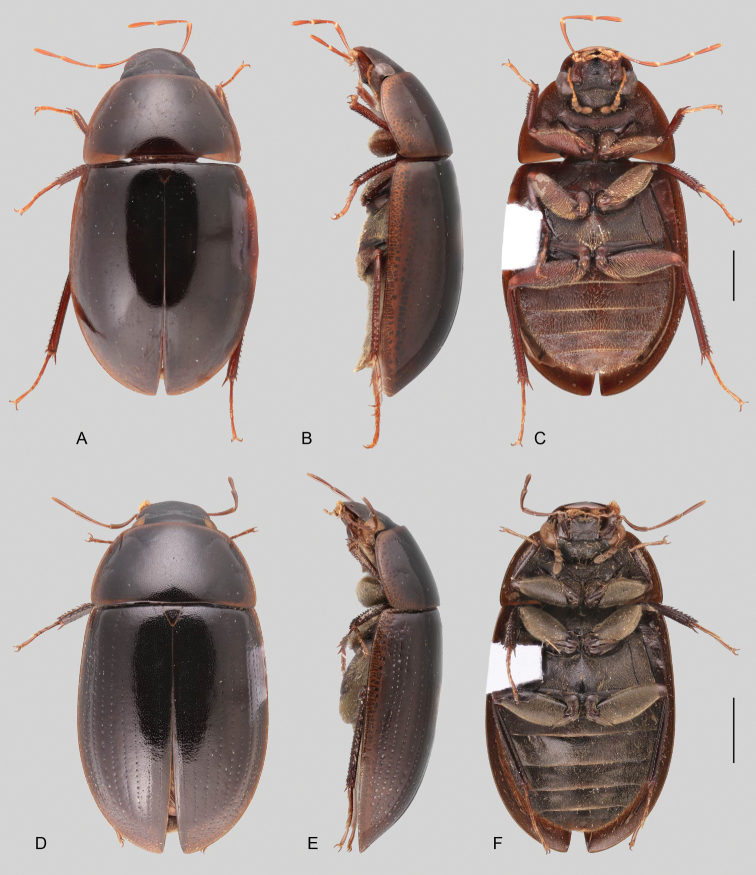
Habitus of *Novochares* spp. **A–C***N. sallaei*: **A** dorsal habitus **B** lateral habitus **C** ventral habitus **D–F***N.* sp. (Peru): **D** dorsal habitus **E** lateral habitus **F** ventral habitus. Scale bars: 1 mm.

**Figure 43.**
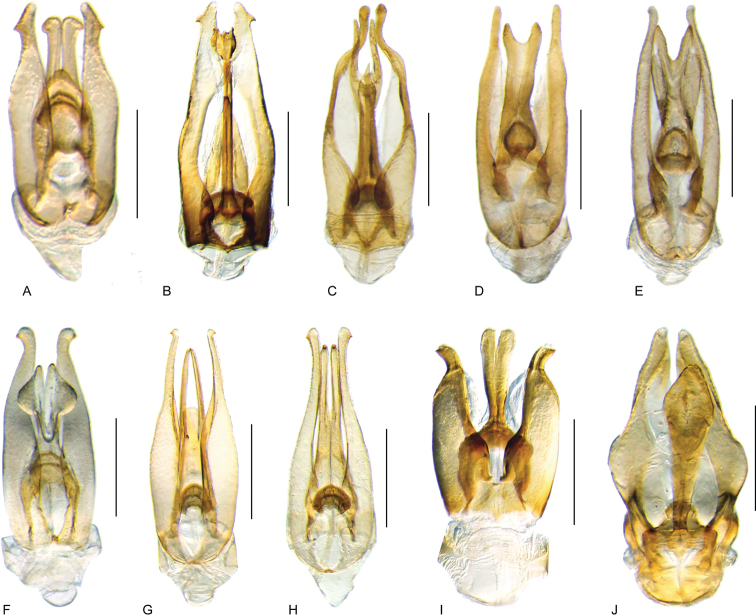
Aedeagi of *Novochares* spp. **A***N.* sp. (Ecuador) **B***N. abbreviatus***C***N. pallipes***D***N. chaquensis***E***N. atratus***F***N. pichilingue***G**N. cf. tectiformis**H**N. cf. coya**I**N. cf. guadelupensis**J**N. cf. cochlearis. Scale bars: 0.5 mm.

Figs 1G , 2 , 6 , 42 , 43

Helochares “Clade D”, Short et al. (2021)

##### Gender.

Masculine.

##### Type species.

*Helochares tectiformis* Fernández, 1982b; by present designation.

##### Etymology.

From the Latin word *novus*, meaning new, in reference to the genus being restricted to the New World, combined with the ending *chares*, expressing affinity with *Helochares*. Masculine.

##### Diagnosis.

Medium sized beetles, body length 4.5–9.0 mm. Body shape oval in dorsal view; slightly to moderately convex in lateral view, with dorsal outline nearly flat along anterior half of elytra, or somewhat evenly curved (Fig. 42). Coloration usually uniformly dark brown, sometimes orange or pale brown; ground punctation shallow to moderately marked (Fig. 42). Shape of head trapezoid. Eyes relatively large, not emarginated anteriorly, usually projected from outline of head. Clypeus trapezoid, with anterior margin broadly and roundly emarginate. Labrum fully exposed. Mentum with lateral longitudinal crenulations, lateral oblique ridges, and transverse crenulations along antero-medial area (Fig. 42C, F). Antennae with nine antennomeres; cupule strongly asymmetric, with rounded outline; antennomere 9 slightly to 2 × longer than antennomere 7. Maxillary palps slender, moderately long, 1.1–1.5 × the width of head; inner margin of maxillary palpomere 2 weakly and evenly curved to nearly straight, outer margin evenly curved or curved along apical half; maxillary palpomere 3 slightly longer than 4 (Fig. 42C, F). Prosternum flat to weakly convex. Elytra without sutural striae, with ground punctures usually shallowly marked; usually at least one row of systematic punctures visible along midline of each elytron; serial punctures sometimes visible along posterior half of elytra (e.g., Fig. 42D). Posterior elevation of mesoventrite, usually simply bulging, sometimes bulge impressed posteriorly, sometimes bulge extends anteriorly as low, shiny, and glabrous longitudinal ridge; anapleural sutures concave, separated at anterior margin by distance 0.6–0.9 × the width of anterior margin of mesepisternum. Metaventrite with medial glabrous patch, sometimes very narrow and extending along entire length of metaventrite (e.g., Fig. 42C, F). Protibiae with spines of anterior row extremely reduced to tiny appressed denticles. Metafemora with tibial grooves well developed; hydrofuge pubescence covering basal 6/7 of anterior surface. Tarsomeres 1–4 with long, thick, and rather dense setae on ventral face, sometimes with only rows of short spines on metatarsomeres 2–4; metatarsomere 2 as long or slightly longer than 5 and as 3 and 4 combined. Fifth abdominal ventrite apically emarginate, with fringe of stout setae. Aedeagus divided (Fig. 43); parameres separated from each other for most of their lengths; median lobe divided in dorsal and ventral plates; dorsal plate usually strongly sclerotized and elongated, often bifurcated or otherwise shaped along apical region; ventral plate sometimes reduced, usually simple and of variable length; basal piece 0.3 × or less than length of parameres, usually clearly noticeable; gonopore usually clearly visible.

##### Differential diagnosis.

*Novochares* includes medium sized, pale to dark brown species that are somewhat dorsoventrally compressed and highly polished (smooth, and often shiny) to the naked eye. In the New World the most similar genus is *Aulonochares*, from which it can be differentiated by the shape of the head [trapezoid in *Novochares*, subquadrate in *Aulonochares* (Fig. 11J)], and the sculpture of the mentum (variously striate in *Novochares*, punctate in *Aulonochares*). Some members of the New World *Helochares* may resemble *Novochares* in their external features, but the aedeagal form is completely different (tubular in *Helochares*, Figs 16E, F, 37; divided in *Novochares*, Figs 16G, H, 43).

From the rest of acidocerines, *Novochares* externally is strikingly similar to the dark and highly polished members of the Old World genus *Peltochares* (compare Fig. 1B vs 1G), from which *Novochares* can be distinguished by the shape of the posterior elevation of the mesoventrite (simply and broadly bulging, often with additional anterior low longitudinal ridge in *Novochares*, longitudinally elevated in *Peltochares*), in addition to characteristics of the male genitalia (divided aedeagus in *Novochares* (Figs 16G, H, 43), spiked aedeagus in *Peltochares* (Figs 16C, D, 45); see also explanation under the aedeagus section of Morphological variation in Acidocerinae and its taxonomic importance).

To differentiate *Novochares* from dark brown, relatively flattened, highly polished, and 4–5 mm long species of *Helochares*, the most reliable feature for identification would be the male genitalia: *Novochares* always exhibit divided aedeagi (Figs 16G, H, 43; parameres separated from each other for most of their lengths, dorsal plate of the median lobe usually strongly sclerotized, elongated, often bifurcated or otherwise shaped along its apical region), whereas in *Helochares* the aedeagi are always tubular (Figs 16E, F, 37A–H; parameres fused to each other for most of their lengths, median lobe with very long basal apodemes; see also explanation under the aedeagus section of Morphological variation in Acidocerinae and its taxonomic importance).

##### Distribution.

**Nearctic**: U.S.A. (Florida; thought to be introduced). **Neotropical**: Argentina, Belize, Bolivia, Brazil (Amazonas, Espírito Santo, Mato Grosso, Mato Grosso do Sul, Minas Gerais, Pernambuco, Piauí, Rio de Janeiro, São Paulo), Colombia, Costa Rica, Cuba, Ecuador, French Guiana, Guatemala, Lesser Antilles (Grenada, Guadeloupe, St. Vincent), Mexico, Panama, Paraguay, Suriname, Uruguay, Venezuela; Fig. 6.

##### Natural history.

Species of *Novochares* occur in a broad range of both lentic and lotic habitats; we are not aware of any seepage specialists in this lineage. Some species such as the widespread *N. abbreviatus* (Fabricius) are found in lentic habitats including marshes, swamps, and pond margins (Short 2005). Forest pools with abundant leaf litter detritus are often very productive for a variety of species. *Novochares atlanticus* (Clarkson and Ferreira-Jr.) was collected at temporary ponds with leaf litter and aquatic vegetation, either covered and shaded in the border of the forest (Clarkson and Ferreira-Jr. 2014), or in open areas. Some species come to lights. Fernández (1983), in describing the immature stages of *N. pallipes* (Brullé), indicated that the species was found on coastal zones, associated with swamp plants (*Spirodela intermedia*; Araceae).

##### Larvae.

The immature stages are only known for *Novochares pallipes* (Brullé) (described as Helochares (s. str.) pallipes Brullé in Fernández 1983: 444); egg sac, first, second and third instar larvae, and pupa are described and illustrated. From each egg sac, 80–103 larvae emerged (Fernández 1983).

##### Taxonomic history.

Species of *Novochares* have been described since as early as 1801, but it was only with the investigations by Fernández in the 1980’s (Fernández 1981, 1982a, 1982b, 1983, 1989) that the group was studied in a comparative taxonomic framework beyond the description of single species.

##### Remarks.

There are 15 species of *Novochares* described to date. Species of *Novochares* tend to have moderate to shallow punctation and serial punctures are usually absent. There is a group of species with serial punctures visible along the posterior half to third of the elytra (Clade D1 in Short et al. 2021).

##### Species examined.

*Novochares abbreviatus* (Fabricius), *N. carmona* (Short), *N. chaquensis* (Fernández), *N. cochlearis* (Fernández), *N. coya* (Fernández), *N. guadelupensis* (d’Orchymont), *N. pallipes* (Brullé), *N. sallaei* (Sharp), *N. tectiformis* (Fernández). Paratypes of *N. carmona* were examined for this study.

##### Selected references.

Fernández 1982a: notes on the taxonomic status of some of the previously described species; Fernández 1982b: description of four new species; Fernández 1983: description of immature stages for *Novochares pallipes* (Brullé); Fernández 1989: one new species and identification key; Short 2005: one new species with review of Central American species; Clarkson and Ferreira-Jr 2014: one new species and new records from southern Brazil; Short et al. 2021: phylogenetic placement.

#### Genus Peltochares

Taxon classificationAnimaliaColeopteraHydrophilidae

Régimbart, 1907

**Figure 44.**
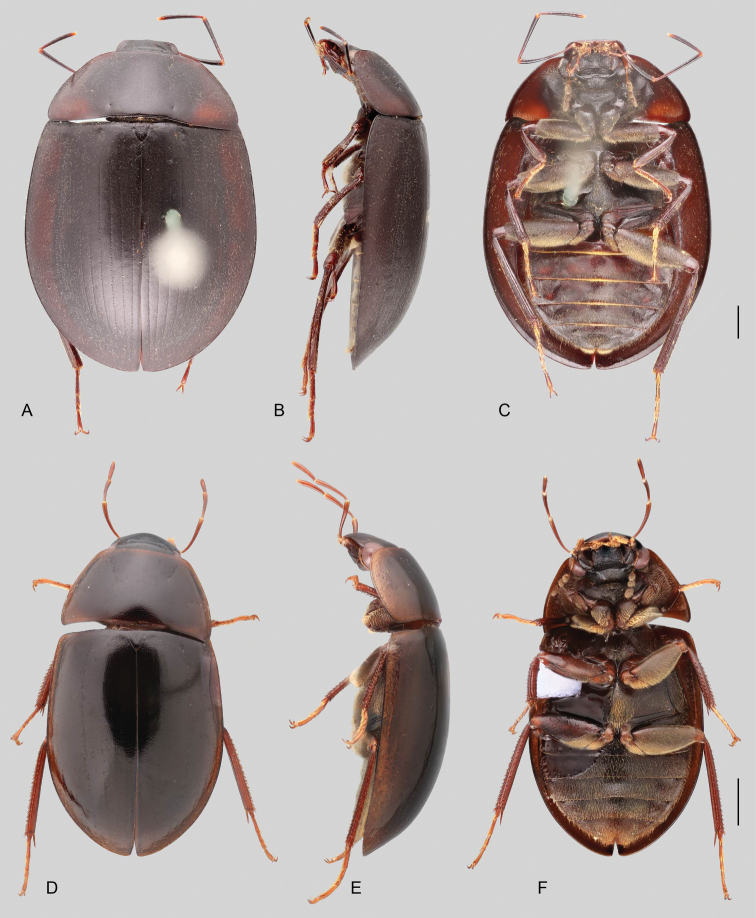
Habitus of *Peltochares* spp. **A–C***P. conspicuus*: **A** dorsal habitus **B** lateral habitus **C** ventral habitus **D–F***P.* sp. (Tanzania): **D** dorsal habitus **E** lateral habitus **F** ventral habitus. Scale bars: 1 mm.

**Figure 45.**
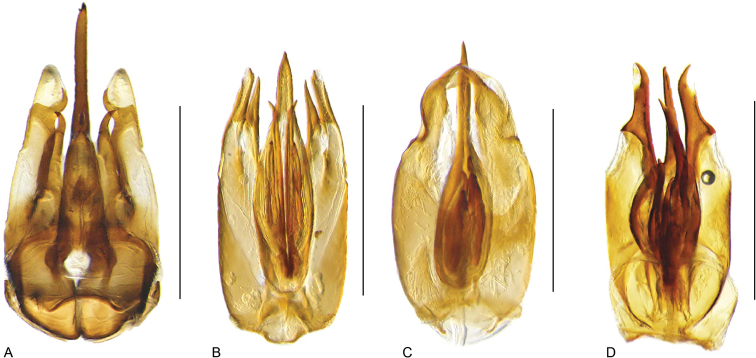
Aedeagi of *Peltochares* spp. **A***P. conspicuus***B***P. foveicollis***C***P.* sp. (Australia) **D***P.* sp. (Tanzania;). Scale bars: 1 mm.

Figs 1B, C , 4 , 11K , 44 , 45

Peltochares Régimbart, 1907: 49.
Type species. Peltochares conspicuus Régimbart, 1907: 49; by monotypy.
Stagnicola Montrouzier, 1860: 246 [preoccupied name by Stagnicola Gray, 1840 (Mollusca)]
Type species: Stagnicola foveicollis Montrouzier, 1860: 246; by monotypy; Bedel 1880: CXLVIII [synonymy].
Neohydrobius Blackburn, 1898: 221.
Type species: Philhydrus burrundiensis Blackburn, 1890: 447; by monotypy; d’Orchymont 1919b: 228 [synonymy].
Helochares “Clade C” in Short et al. 2021.

##### Gender.

Masculine.

##### Type species.

*Peltochares conspicuus* Régimbart, 1907: 49; by monotypy.

##### Diagnosis.

Body length 6–14 mm. Body shape oval in dorsal view, weakly to moderately convex in lateral view (Fig. 44). Dorsal surfaces even and smooth, either uniformly covered by short setae (Fig. 44A), or with scarce long setae along particular areas of surface (associated with systematic punctures; Fig. 44D), dark brown in coloration, usually uniform; ground punctation fine and shallow to moderate; ventral surfaces densely covered by fine golden setae (Fig. 44C, F). Head subquadrate (Fig. 11K). Eyes not emarginate, moderate in size, subquadrate, separated by 4.5–5.5 × width of eye, strongly projected from outline of head. Clypeus with anterior margin broadly emarginate, either roundly or acutely, sometimes further medially notched; membranous preclypeal area visible when clypeus strongly emarginated. Labrum fully exposed, often medially convex. Antennae with nine antennomeres, with moderately asymmetric and round cupule; antennomere 9 slightly to 2 × longer than antennomere 7. Maxillary palps slender, 1.3–1.8 × longer than maximum width of head, with palpomere 4 nearly 0.8 × as long as palpomere 3; maxillary palpomere 2 with inner margin slightly and evenly curved, and outer margin curved along apical half (Fig. 44C, F). Mentum slightly depressed mesally, surface laterally punctate, mesally and anteriorly striate, with anteromedial region depressed (Fig. 44C, F). Submentum punctate to crenulate. Pronotum evenly convex, usually with systematic punctures forming distinct anterolateral semicircles. Elytra without sutural striae, with margins usually only slightly flared (explanate in *P. conspicuus*; Fig. 44A); serial punctures usually absent (visible along entire length of elytra in *P. conspicuus*; Fig. 44A); ground punctation usually shallow (moderate to strongly marked in *P. foveicollis*). Surface of prosternum flat to broadly convex, with anterior margin roundly projected anteriorly (Fig. 44C, F). Posterior elevation of mesoventrite usually with longitudinal or somewhat longitudinal elevation, sometimes forming acute posterior point; apical region of elevation usually with long fine setae; anapleural sutures forming obtuse angle, nearly parallel along anterior section, separated anteriorly by distance 0.3–0.7 × anterior margin of mesepisternum. Metaventrite densely covered by hydrofuge pubescence, except for posterolateral patches (Fig. 44C, F). Protibiae with anterior row of spines reduced to extremely reduced (Fig. 44C); apical spurs of protibiae stout, ranging from very large (larger spur considerably larger and thicker than tarsal claws, e.g., *P. foveicollis*), or very short (barely reaching apex of protarsomere 1, e.g., *P. conspicuus*); pro- and mesotarsal claws are sexually dimorphic in some species (e.g., *P. foveicollis*). Metafemora with tibial grooves sharply marked; metafemora with hydrofuge pubescence covering at least basal 3/4 of anterior surface (Fig. 44C, F). Metatarsomeres 5 and 2 similar in length or 2 slightly longer, metatarsomere 2 slightly longer than metatarsomeres 3 and 4 combined; all tarsomeres with ventral surface rather densely covered by long spiniform setae on ventral surface (sparser on tarsomere 5). Abdomen with five pubescent ventrites. Fifth abdominal ventrite with apex emarginate, fringed by stout setae. Aedeagus spiked (Figs 16C, D, 45); main component of median lobe strongly sclerotized, slender, and apically acute, usually accompanied by additional shorter slender sclerotizations; apical region of parameres usually partly heavily sclerotized and partly membranous, often bifurcated; basal piece strongly reduced; gonopore usually not clearly visible.

##### Differential diagnosis.

The type species of *Peltochares* is easily recognized by its external morphology alone: laterally explanate pronotum and elytra, well defined serial punctures along elytra (Fig. 44A), which somewhat resembles *Helobata* (Fig. 33A), from which *P. conspicuus* can be distinguished by the exposed labrum of *Peltochares* (Fig. 11K; concealed labrum in *Helobata*, Fig. 11L). The most common forms of *Peltochares* more closely resemble *Novochares* and some *Helochares*, because of their darkly colored and highly polished dorsal habitus. Besides being distributed (although widespread) in the Old World, *Peltochares* species can be distinguished from the New World *Novochares* by the shape of the posterior elevation of the mesoventrite (longitudinally elevated in *Peltochares*, simply and broadly bulging, often with additional anterior low longitudinal ridge in *Novochares*), in addition to characteristics of the male genitalia (spiked aedeagus in *Peltochares*, Figs 16C, D, 45; divided aedeagus in *Novochares*, Figs 16G, H, 43; see also explanation under the aedeagus section of Morphological variation in Acidocerinae and its taxonomic importance). From dark brown, highly polished, and relatively large species of *Helochares*, *Peltochares* can be distinguished by their slender maxillary palps, that are 1.3–1.8 × longer than the width of the head (Fig. 44C, F), as opposed to shorter (0.6–1.2 × the width of the head) and relatively stout maxillary palps in *Helochares* (Figs 35C, F, 36C, F), in addition to the aedeagal form (spiked in *Peltochares*, Figs 16C, D, 45; tubular in *Helochares*, Figs 16E, F, 37A–H; see also explanation under the aedeagus section of Morphological variation in Acidocerinae and its taxonomic importance).

##### Distribution.

**Afrotropical**: Angola, Benin, Botswana, Burkina Faso, Burundi, Cameroon, Central African Republic, Chad, Democratic Republic of the Congo, Ethiopia, Gabon, Gambia, Ghana, Guinea, Ivory Coast, Kenya, Liberia, Madagascar, Malawi, Mozambique, Namibia, Niger, Nigeria, Republic of the Congo, Rwanda, Senegal, Sierra Leone, Somalia, Republic of South Africa, South Sudan, Tanzania, Togo, Uganda, Western Sahara, Zambia, Zimbabwe. **Australasian**: Australia (Australian Capital Territory, New South Wales, Northern Territory, Queensland, Western Australia), Indonesia (Papua), New Caledonia, Papua New Guinea. **Indo-Malayan**: Bangladesh, Cambodia, China (Guangdong, Guangxi, Guizhou, Hong Kong, Jiangxi, Macao), Indonesia (Borneo, Sumatra), Laos, Malaysia, Nepal, Sri Lanka, Thailand, Vietnam. **Palearctic**: Canary Islands, Egypt, Israel, Japan (Nansei Islands); Fig. 6.

##### Natural history.

Even though species currently placed in *Peltochares* have been treated in faunistic and taxonomic studies (e.g., Watts 1995, Hebauer 2001b), little is known about their ecology. Jia and Tang (2018) recently reported that *P. atropiceus* (Régimbart) was living in natural ponds with leaf litter or water grass, sometimes collected on wet ground with plenty of grass; it can be collected at light in May and June in South China and has never been collected from the edges of rivers and streams. The female carries the egg case under the abdominal ventrites (Jia and Tang 2018).

##### Larvae.

Larval stages of *Peltochares conspicuus* Régimbart, were described by Bertrand (1962) from larvae collected along with adults on the surface of rocks in Madagascar. Fikáček (2003) provides a diagnosis of the larvae described by Bertrand (1962), but questions their identification, given that *P. conspicuus* has never been recorded from Madagascar. It seems most probable the description is of another species now placed *Peltochares*, as *P. longipalpis* has been recorded from Madagascar, but only future rearing or DNA sequencing of putative larvae will confirm this.

##### Lectotype designation.

We examined Régimbart’s syntype series for *Peltochares conspicuous*, consisting of nine specimens, that are deposited in the Muséum national d’Histoire naturelle, Paris, France. We determined all nine to be conspecific. It includes two specimens labeled ‘Cape Lopez’, one of them labeled ‘*Peltochares conspicuus* Rég.’; five specimens labeled Rembo N’Comi Fernand Vaz, one of them missing prothorax and head, and another one is missing the left elytron; one specimen labeled Rembo N’Comi Fernand Vaz (Gabon), missing prothorax and head; and one specimen labeled ‘Gabon’. All specimens, except the last one, are pinned; the specimen labeled ‘Gabon’ is glued by its abdomen in a small pinned card. To stabilize the identity of the type species of *Peltochares*, we here designate as the Lectotype the specimen that bears the ‘*Peltochares conspicuus* Rég.’ label, which even though is not completely clean, has all its appendages complete. The following red label has been attached: “LECTOTYPE/ Peltochares/ conspicuus/ Régimbart/ des. Girón and Short”. The remaining eight specimens are designated as paralectotypes. One of the specimens missing its prothorax and head was dissected to reveal the male genitalia, which is illustrated in Fig. 45A.

##### Taxonomic history.

The circumscription of *Peltochares* as used here is changed from its original meaning. *Peltochares* was originally described as a monotypic genus by Régimbart in 1907, from specimens collected in Gabon of a very unusual species (*P. conspicuus*) which was a rather large, circular beetle with extremely explanate margins of the pronotum and elytra (Fig. 44A–C). A morphologically similar species was much later described from Indonesia and Malaysia, although that species was placed in the nominal subgenus of *Helochares* (Helochares (s. str.) discus Hebauer, Hendrich & Balke). In their molecular phylogeny, Short et al. (2021) recovered *H. discus* in a clade (*Helochares* Clade C) with some other larger, darkly colored (but not explanate) Old World species that were also placed in *Helochares* (s. str.), which showed that this clade was not closely related to the “true” *Helochares* but indeed represented an independent lineage. Examination of the male genitalia of one of the syntypes of *P. conspicuus* (the type of *Pelotochares*) and members of “*Helochares* Clade C” in Short et al. (2021) revealed that they share a quite unique and similar configuration of the male genitalia (spiked genitalia, Figs 16C, D, 45; see also the aedeagus section of Morphological variation in Acidocerinae and its taxonomic importance above), even though they do not share the same extremely explanate body form.

Although the monophyly and morphological circumscription of “*Helochares* Clade C” is strongly supported, the proper genus name to assign to his lineage is not straightforward, as there are several generic names that had been long synonymized with *Helochares* that potentially come into play with the new circumscription of the genus. The genus *Stagnicola* Montrouzier, 1860 was based on what is now Helochares (s. str.) foevicollis, a species which is a definitive member of *Helochares* Clade C. However, *Stagnicola* is a preoccupied name and thus unavailable. More complicated is *Neohydrobius* Blackburn, 1898 and its type species, *Philhydrus burrundiensis* Blackburn, which is now considered a junior synonym of H. (s. str.) foevicollis. *Neohydrobius*, although eight years older than *Peltochares*, had a very short shelf-life, as it was synonymized with *Helochares* just 21 years after it was proposed by d’Orchymont (1919b) and therefore has not been used in more than a century. Meanwhile, *Peltochares* has been in continuous usage since 1907 and therefore we believe it is the best and most stable name to apply to this clade.

We had hoped to unilaterally maintain prevailing usage of *Peltochares* over *Neohydrobius* by invoking ICZN Article 23.9.1. However, not all the required criteria to apply this article appear to be met in this case. Although *Neohydrobius* appears to meet the first criterion (the senior synonym not being used as valid since 1899), we were only able to identify 19 works (by more than 10 authors) in the immediately preceding 50 years, but 25 works are required. Therefore, we will formally appeal to the commission for a ruling to maintain *Peltochares* over *Neohydrobius*. Accordingly, ICZN Article 82.1 states that prevailing usage is to be maintained until the ruling of the Commission is published and therefore, we use *Peltochares* in this work.

##### Remarks.

The group of species previously assigned to *Helochares* (s. str.), hereby transferred to *Peltochares*, was first recognized by Hebauer (2001b) as a discrete unit in morphological terms within *Helochares*. There are currently eight described species of *Peltochares*, including the following seven species that are transferred from *Helochares* for the first time: *P. atropiceus* (Régimbart) comb. nov., *P. ciniensis* (Hebauer, Hendrich & Balke) comb. nov., *P. discus* (Hebauer, Hendrich & Balke) comb. nov., *P. foveicollis* (Montrouzier) comb. nov., *P. longipalpis* (Murray) comb. nov., *P. papuensis* (Hebauer) comb. nov., and *P. taprobanicus* (Sharp) comb. nov.

##### Species examined.

*Peltochares atropiceus*, *P. ciniensis* (including a paratype), *P. conspicuus* (including syntypes), *P. foveicollis*, *P. longipalpis*, and *P. taprobanicus*.

##### Selected references.

Régimbart 1907: 49: original description of the genus; Hebauer 2001b: taxonomic treatment of *P. taprobanicus* (as *Helochares taprobanicus*) and allied species; Jia and Tang 2018: faunistic review of Chinese species including a redescription and some biological notes on *P. atropiceus*; Short et al. 2021: phylogenetic placement.

#### Genus Primocerus

Taxon classificationAnimaliaColeopteraHydrophilidae

Girón & Short, 2019

**Figure 46.**
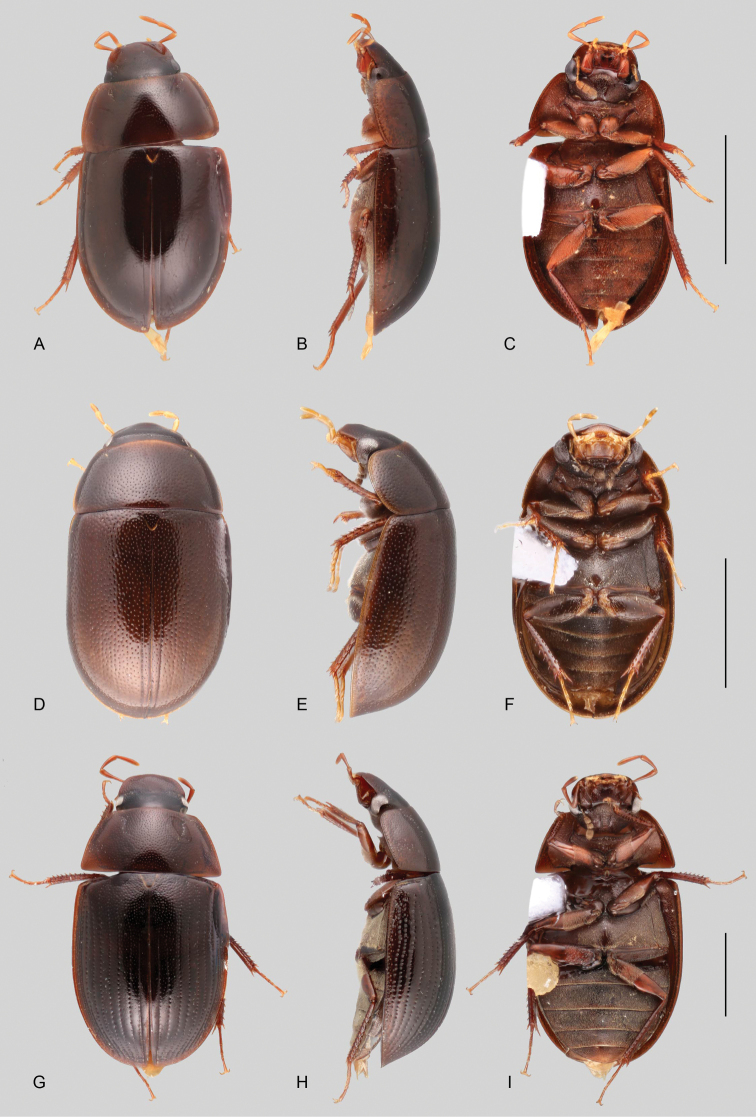
Habitus of *Primocerus* spp. **A–C***P. neutrum*: **A** dorsal habitus **B** lateral habitus **C** ventral habitus **D–F***P. maipure*: **D** dorsal habitus **E** lateral habitus **F** ventral habitus **G–I***P. semipubescens*: **G** dorsal habitus **H** lateral habitus **I** ventral habitus. Scale bars: 1 mm.

**Figure 47.**
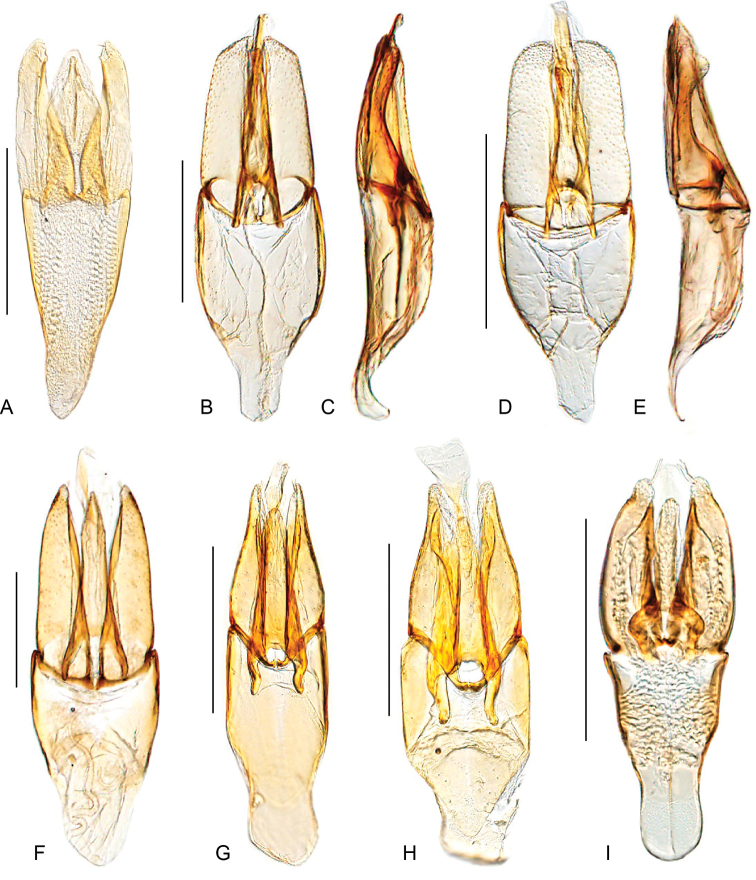
Aedeagi of *Primocerus* spp. **A***P. neutrum***B, C***P. maipure*: **B** dorsal view **C** lateral view **D, E***P. pijiguaense*: **D** dorsal view **E** lateral view **F***P. gigas***G***P. petilus***H***P. striatolatus***I***P. cuspidis*. Scale bars: 0.25 mm.

Figs 1R , 2 , 6 , 46 , 47

Primocerus Girón & Short, 2019: 133.

##### Gender.

Masculine.

##### Type species.

*Primocerus neutrum* Girón & Short, 2019: 147; by original designation.

##### Diagnosis.

Small to medium sized beetles, body length 2.4–4.9 mm. Body shape elongated oval in dorsal view; moderate to strongly convex in lateral view; dorsal outline uniformly convex or nearly straight and anteriorly inclined along anterior half (Fig. 46). Color brown, dark brown, reddish brown, or rather orange, usually uniform along body regions, but sometimes with slightly paler margins, pronotum or ventral surfaces and appendages; ground punctation shallow to moderately marked (Fig. 46). Shape of head trapezoid. Eyes small to moderate, seldom very small, not emarginated anteriorly, usually projected from outline of head. Clypeus trapezoid, with anterior margin broadly and roundly emarginate. Labrum fully exposed. Mentum rather flat and smooth, sometimes with lateral oblique ridges, and few crenulations; median anterior depression sometimes marked by a transverse carina (Fig. 46C, F, I). Antennae with eight antennomeres; cupule slightly asymmetric, with rounded outline. Maxillary palps moderately stout, shorter to nearly as long as width of head; inner margin of maxillary palpomere 2 nearly straight, outer margin curved along apical 2/3; maxillary palpomeres 3 and 4 similar in length (Fig. 46C, F, I). Prosternum flat to mesally only slightly produced (Fig. 46C, F, I). Elytra with sutural striae; elytral punctures from shallow to sharply marked; ground punctures rather uniformly distributed; some species with serial punctures; outer margins of elytra slightly flared (Fig. 46A, D, G). Posterior elevation of mesoventrite usually with curved transverse ridge, rather sharp and low, or bearing sharp, pyramidal (triangular) projection; anapleural sutures concave to forming obtuse angle, separated at anterior margin by distance 0.3–0.4 × width of anterior margin of mesepisternum (Fig. 46C, F, I). Metaventrite with posteromesal glabrous patch nearly as wide as long (Fig. 46C, F, I). Protibiae with spines of anterior row as thick, long semi-erect setae; apical spurs of protibiae moderately stout, reaching midlength of protarsomere 3. Metafemora with tibial grooves moderately developed; hydrofuge pubescence coverage ranging from sparse (nearly glabrous metafemora) to dense along basal 3/4 (Fig. 46C, F, I). Tarsomeres 1–4 with long spiniform setae on ventral face; metatarsomere 2 nearly as long as 5 and as 3 and 4 combined. Fifth abdominal ventrite apically rounded, truncate, or slightly emarginate, usually with fringe of stout setae. Aedeagus trilobed (Fig. 47); basal piece as long or longer than parameres; median lobe triangular, nearly as wide at base as basal width of each paramere, with apical projection; gonopore absent.

##### Differential diagnosis.

At first sight, the smoother members of *Primocerus* (e.g., Fig. 46A–C) can be mistaken for *Chasmogenus* (Fig. 24), given that both genera exhibit sutural striae. The presence of a transverse curved ridge (sometimes very low) on the posterior elevation of the mesoventrite distinguishes *Primocerus* from *Chasmogenus*, in which the mesoventrite is either flat, broadly elevated or with a longitudinal elevation; maxillary palps of most *Chasmogenus* species are nearly 1.5 × longer than the maximum width of the head, whereas in *Primocerus* the maxillary palps are shorter, nearly as long as the width of the head.

Punctate members of *Primocerus* (e.g., Fig. 46D–F) may resemble some species of *Tobochares* (Kohlenberg and Short 2017, Girón and Short 2021a); striate *Primocerus* (e.g., Fig. 46G–I) may resemble *Radicitus* (Fig. 50; Short and García 2014). In those cases, *Primocerus* can be easily recognized by the presence of sutural striae. Some species of *Primocerus* may also superficially resemble certain New World cylomine genera, such as *Andotypus* Spangler (Fikáček et al. 2014), from which it may be distinguished by the fully exposed labrum of *Primocerus*.

##### Distribution.

**Neotropical**: Brazil (Pará), Guyana, Suriname, and Venezuela; Fig. 6. We have seen additional specimens that slightly expand the range of the genus, but all still fall within the Guiana Shield region of South America.

##### Natural history.

The habitats occupied by members of *Primocerus* range from forested pools to seepages. One specimen was collected with a flight intercept trap. Specimens of *Primocerus* are relatively rare, given that so far have only been found in low numbers of specimens per collecting event (Girón and Short 2019).

##### Larvae.

Immature stages are not known for *Primocerus*.

##### Taxonomic history.

*Primocerus* was only recently described.

##### Remarks.

With only nine known species in the genus, *Primocerus* is one of the most variable genera of New World acidocerines in terms of their external morphology. Additional recent study and collections have revealed that the species described as *P. neutrum* likely represents a species complex (Short pers. obs.).

##### Species examined.

Holotypes and paratypes of all known species were examined for this study.

##### Selected references.

Girón and Short 2019: original description of the genus and all its known species; Short et al. 2021: phylogenetic placement.

#### Genus Quadriops

Taxon classificationAnimaliaColeopteraHydrophilidae

Hansen, 1999

**Figure 48.**
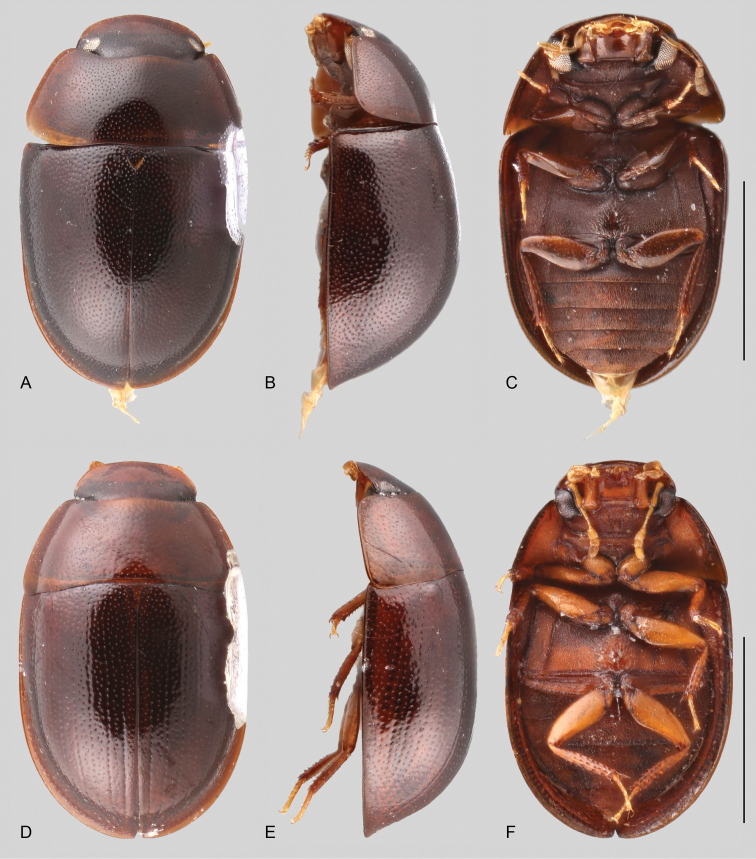
Habitus of *Quadriops* spp. **A–C***Q. acroreius*: **A** dorsal habitus **B** lateral habitus **C** ventral habitus **D–F***Q. clusia*: **D** dorsal habitus **E** lateral habitus **F** ventral habitus. Scale bars: 1 mm.

**Figure 49.**
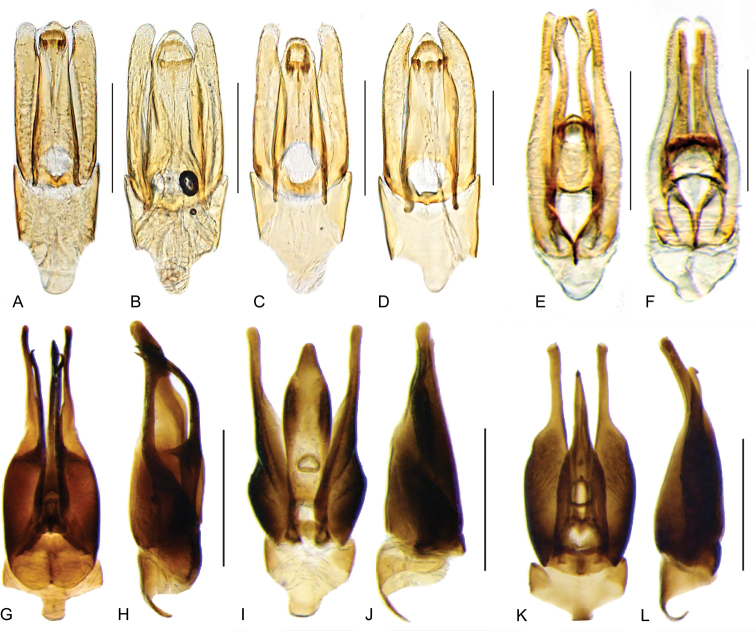
Aedeagi of *Quadriops*, *Radicitus* and *Sindolus* spp. **A***Q. clusia***B***Q. depressus***C***Q. reticulatus***D***Q. similaris***E***S.* sp. (Venezuela) **F**. *S.* sp. (Venezuela) **G, H***R. ayacucho*: **G** dorsal view **H** lateral view **I, J**R. cf. granitum (Suriname): **I** dorsal view **J** lateral view **K, L***R. surinamensis*: **K** dorsal view **L** lateral view. Scale bars: 0.1 mm (**A–D**); 0.5 mm (**E–L**).

Figs 1P , 2 , 6 , 11C , 48 , 49A–D

Quadriops Hansen, 1999a: 131.

##### Gender.

Masculine.

##### Type species.

*Quadriops depressus* Hansen, 1999a: 136; by original designation.

##### Diagnosis.

Small to very small beetles, body length 1.6–2.6 mm. Body shape oval in dorsal view; moderate to strongly convex in lateral view, dorsal outline evenly convex or nearly straight along median region (Fig. 48). Color orange brown to dark brown, uniform along body regions; ground punctation shallow to moderately marked (Fig. 48). Shape of head somewhat rectangular. Frons lateral and posteriorly expanded, forming canthus completely dividing eyes in dorsal and ventral portions (Fig. 11C). Eyes very small in dorsal view. Clypeus laterally expanded in front and around outer margin of eyes; anterior margin of clypeus straight (Fig. 11C). Labrum partly exposed. Mentum rather smooth and medially depressed; median anterior depression marked by a transverse carina (Fig. 48C, F). Antennae with nine antennomeres, cupule slightly asymmetric with rounded outline. Maxillary palps rather short and stout, nearly half as long as width of head; maxillary palpomere 4 slightly longer than palpomere 3; inner margin of maxillary palpomere 2 straight to convex, outer margin strongly curved along apical 2/3. Elytra without sutural striae, with punctures either irregularly distributed or forming well defined longitudinal rows; elytra narrowly explanate anteriorly, explanation gradually broader towards apex (Fig. 48). Surface of prosternum flat. Posterior elevation of mesoventrite, usually with well-defined transverse ridge, seldom with acute tooth; anapleural sutures concave, separated at anterior margin by distance nearly 0.7 × width of anterior margin of mesepisternum. Metaventrite usually uniformly densely pubescent, sometimes with reduced posteromedian glabrous patch. Protibiae with spines of anterior row hair-like, semi erect, relatively long, and thick; apical spurs of protibia moderately stout, reaching apex of protarsomere 3. All tarsomeres with thick hair-like spines on ventral face of tarsomeres 2–4; metatarsomeres 1–4 similar in length, 5 nearly as long as 3 and 4 combined. Metafemora with tibial grooves moderately developed; anterior surface of metafemora mostly glabrous, with few very scattered small setae (Fig. 48C, F). Fifth abdominal ventrite apically rounded and without fringe of stout setae. Aedeagus trilobed (Fig. 49A–D), with basal piece about half length of parameres; median lobe wider than base of each paramere, with narrow, triangular, longitudinal sclerite, usually extending along apical third; parameres as long as, to longer than median lobe, and nearly half as wide; gonopore situated preapically; basal piece with lateral margins straight to sinuate, apically slightly diverging.

##### Differential diagnosis.

*Quadriops* is the only known acidocerine with fully divided eyes. Species with uniformly distributed punctures along the elytra may resemble *Globulosis*, but the moderate punctation of *Quadriops* is very evident (punctation only shallowly marked in *Globulosis*; Fig. 32). Some species of *Tobochares* have nearly divided eyes, and lack impressed striae along the elytra (*emarginatus* species group, Girón and Short 2021a), resembling species of *Quadriops* with uniformly distributed punctures along the elytra, but they differ in the shape of the posterior elevation of the mesoventrite (sharply elevated as a tooth or a blunt transverse carina in *Quadriops*, medially bulging in *T. canthus* Kohlenberg & Short).

##### Distribution.

**Neotropical**: Brazil (Amazonas), Costa Rica, Ecuador, French Guiana, Guyana, Panama, Peru, Suriname, Venezuela; Fig. 6.

##### Natural history.

Specimens have been caught using flight intercept traps, many long series have been collected on decaying *Clusia* fruits, which can be somewhat used as bait (Fig. 10). Additional specimens have been collected in rotten logs, sap flows on freshly cut trees, and in the refuse piles of leafcutter ants (Girón and Short 2017).

##### Larvae.

The immature stages of *Quadriops* remain unknown.

##### Taxonomic history.

Hansen (1999a) described the genus with five species, differentiated mostly by the presence and degree of impression of reticulation on the head and clypeus. When he originally described it, Hansen (1999a) was unsure of the taxonomic affinity of the genus, as the morphology of the lineage was somewhat unusual. He placed it in the Acidocerina (now Acidocerinae) almost by default as it shared no characters in common with other lineages, but ultimately, he was correct as this placement as verified by Short et al. (2021). García (2000b) described an additional species from Venezuela. The genus was revised by Girón and Short (2017): two species were synonymized with *Quadriops depressus* Hansen; two new species were described.

##### Remarks.

*Quadriops* is the only fully terrestrial genus of Acidocerinae. There are six described species within the genus.

##### Species examined.

*Quadriops acroreius* Girón & Short (holotype and paratype), *Q. clusia* Girón & Short (holotype, paratypes and additional specimens), *Q. dentatus* Hansen (holotype and additional specimens), *Q. depressus* Hansen (holotype and additional specimens), *Q. reticulatus* Hansen (holotype and additional specimens), *Q. similaris* Hansen (holotype and additional specimens).

##### Selected references.

Hansen 1999a: original description; García 2000b: description of one additional species from Venezuela; Girón and Short 2017: generic revision including two synonymies and two new species; Short et al. 2021: phylogenetic placement.

#### Genus Radicitus

Taxon classificationAnimaliaColeopteraHydrophilidae

Short & García, 2014

**Figure 50.**
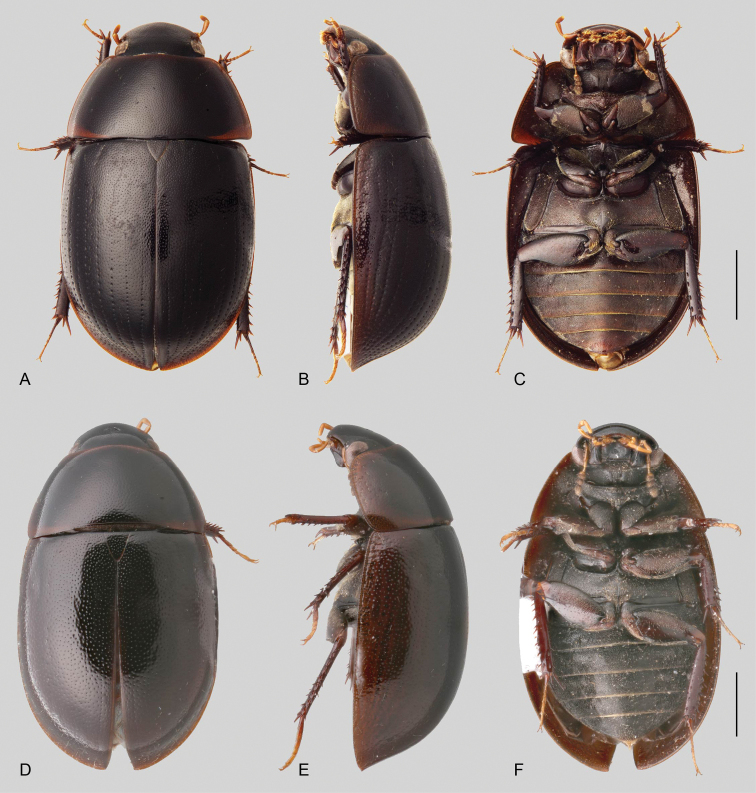
Habitus of *Radicitus* spp. **A–C***R. ayacucho*: **A** dorsal habitus **B** lateral habitus **C** ventral habitus **D–F***R. granitum*: **D** dorsal habitus **E** lateral habitus **F** ventral habitus. Scale bars: 1 mm.

Figs 1K , 2 , 6 , 49G–L , 50

Radicitus Short & García, 2014: 252.

##### Gender.

Masculine.

##### Type species.

*Radicitus ayacucho* Short & García, 2014: 252; by original designation.

##### Diagnosis.

Medium sized beetles, body length 4.5–6.2 mm. Body shape oval in dorsal view; moderate to strongly convex in lateral view; dorsal outline nearly straight and anteriorly inclined along anterior half (Fig. 50). Color dark brown, usually uniform along body regions, sometimes margins of pronotum and elytra slightly paler; ground punctation fine, moderately marked (Fig. 50A, D). Shape of head trapezoid and rather wide. Eyes moderate in size, not emarginated anteriorly, slightly projected from outline of head. Clypeus trapezoid, with anterior margin broadly, roundly, and weakly emarginate. Labrum fully exposed. Mentum medially rather broadly depressed, laterally longitudinally elevated; median anterior depression marked by transverse nearly straight carina (Fig. 50C, F). Antennae with nine antennomeres; cupule slightly asymmetric, with rounded outline. Maxillary palps short and stout, nearly as long as half width of head (e.g., Fig. 50C); inner margin of maxillary palpomere 2 nearly straight, outer margin strongly curved along apical 2/3; maxillary palpomere 4 slightly shorter than 3. Prosternum flat, only slightly carinate along midline of anterior projection. Elytra without sutural striae; elytral punctures shallow to moderately marked; ground punctures rather uniformly distributed; some species with serial punctures clearly visible along posterior third of elytra; outer margins of elytra slightly flared (Fig. 50A, D). Posterior elevation of mesoventrite with median longitudinal carina elevated and forming posteriorly pointing process; anapleural sutures strongly concave, separated at anterior margin by distance nearly half width of anterior margin of mesepisternum. Metaventrite sometimes with posteromesal glabrous patch. Protibiae with anterior row of spines completely reduced; apical spurs of protibiae stout, reaching apex of protarsomere 3. Metafemora with tibial grooves very sharply marked and covered by hydrofuge pubescence; hydrofuge pubescence restricted to dorsal half on basal three-quarters of anterior surface of metafemora (Fig. 50C, F). Tarsomeres 1–4 with long spiniform setae on ventral face; metatarsomere 2 nearly as long as 5 and as 3 and 4 combined. Fifth abdominal ventrite evenly rounded, without apical emargination or fringe of stout setae. Aedeagus either trilobed (Fig. 49I–L) or divided (Fig. 49G, H), with basal piece short and rather simple parameres separated from each other for most of their lengths; gonopore well developed.

##### Differential diagnosis.

*Radicitus* may resemble some punctate *Novochares* but can be recognized by the short and stout maxillary palps, along with metafemora only partly covered by pubescence (long and slender maxillary palps with metafemora mostly covered by pubescence in *Novochares*).

##### Distribution.

**Neotropical**: Guyana, Suriname, Venezuela; Fig. 6.

##### Natural history.

Species of *Radicitus* have been found on a variety of habitats associated with streams and seeps on rock outcrops. Some have been collected by submerging root mats found along streams, and in the roots of vegetation growing on seepage areas on granite outcrops (Short and García 2014).

##### Larvae.

The immature stages of *Radicitus* remain unknown.

##### Taxonomic history.

*Radicitus* was only recently described.

##### Remarks.

There are three known species of *Radicitus*, all currently endemic to the Guiana Shield.

##### Selected references.

Short and García 2014: original description of the genus and all known species; Short et al. 2021: phylogenetic placement.

#### Genus Sindolus

Taxon classificationAnimaliaColeopteraHydrophilidae

Sharp, 1882

**Figure 51.**
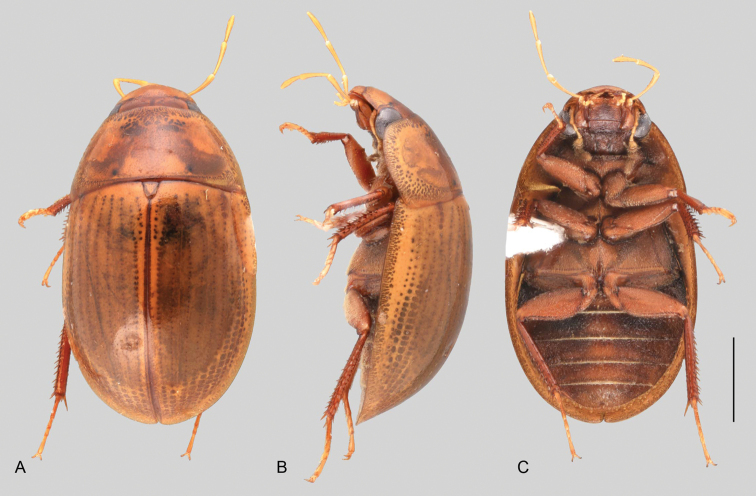
Habitus of *Sindolus optatus***A** dorsal habitus **B** lateral habitus **C** ventral habitus. Scale bar: 1 mm.

Figs 6 , 49E, F , 51

Sindolus Sharp, 1882: 72.
Helochares (Sindolus) Sharp; d’Orchymont 1919c: 148; Knisch 1924: 199; Hansen 1999b: 158.

##### Gender.

Masculine.

##### Type species.

*Sindolus optatus* 1882: 72; by subsequent designation (Hansen 1991: 292).

##### Diagnosis.

Small to medium sized beetles, body length 2.5–5.0 mm. Body shape oval in dorsal view, moderately to strongly convex in lateral view (Fig. 51); dorsal outline usually evenly curved. Dorsal surfaces even and smooth, yellowish, orange brown to brown and rather uniform in coloration; ground punctation fine and extremely shallow (Fig. 51A). Shape of head trapezoid. Eyes not emarginate, moderate to relatively large in size, subquadrate, separated by nearly 5 × width of eye, only slightly projected from outline of head. Clypeus trapezoid, with anterior margin broadly and slightly emarginate. Labrum fully exposed, convex, and anteriorly emarginate. Mentum rather flat, with few shallow transverse crenulations on anterior region; median anterior depression relatively shallow, sometimes marked by transverse carina (Fig. 51C). Submentum smooth to very shallowly sculptured. Antennae with nine antennomeres, with strongly asymmetric and round cupule; antennomere 9 nearly 3 × longer than antennomere 8. Maxillary palps slender, 1.2–1.5 × longer than maximum width of head; inner margin of maxillary palpomere 2 usually evenly weakly curved, outer margin curved along apical third; palpomere 4 nearly 0.8 × as long as palpomere 3 (Fig. 51C). Pronotum evenly convex, usually with systematic punctures forming distinct anterolateral semicircles. Elytra without sutural striae, with margins only slightly flared; serial punctures absent; scarce systematic punctures, bearing moderately long setae (Fig. 51A). Surface of prosternum somewhat longitudinally elevated, sometimes with low and blunt longitudinal carina; anterior margin acutely to roundly projected anteriorly. Posterior elevation of mesoventrite with sharp and strongly elevated (laminar) longitudinal carina, with the ventral edge of the carina usually straight and parallel to the body (Fig. 51C); anapleural sutures concave, separated at anterior margin by distance nearly half width of anterior margin of mesepisternum. Metaventrite densely and uniformly covered by hydrofuge pubescence (Fig. 51C). Protibiae with anterior row of spines reduced (short appressed spines) to extremely reduced (tiny denticles); apical spurs of protibiae moderate, broad and reaching apex of protarsomere 2. Metafemora with tibial grooves sharply marked, and hydrofuge pubescence covering at least basal four fifths of anterior surface (Fig. 51C). Metatarsomere 2 slightly shorter or similar in length to metatarsomere 5, metatarsomere 2 similar in length to metatarsomeres 3 and 4 combined; ventral surface of all tarsomeres with long setiform setae on ventral surface (tarsomeres 1 and 2 with small stout spines). Abdomen with five pubescent ventrites. Fifth abdominal ventrite emarginate at apex; emargination fringed by stout setae. Aedeagus divided (Fig. 49E, F), somewhat pear-shaped, with basal piece nearly 0.3 × length of parameres; parameres slender, narrowing apically, with outer margins at least slightly sinuated, usually apically rounded; median lobe divided into dorsal and ventral plates; dorsal plate of median lobe medially bifurcate, with narrow, slender and apically rounded lobes; ventral lobe of median lobe varying in width and length, usually very lightly sclerotized; gonopore well-developed, usually positioned at midlength of aedeagus.

##### Differential diagnosis.

*Sindolus* is the only known genus of acidocerines that bears a sharp and strongly elevated (laminar) longitudinal carina.

##### Distribution.

**Neotropical**: Argentina, Bolivia, Brazil (Amazonas, Mato Grosso do Sul, Rio de Janeiro, Rio Grande do Sul), Colombia [in doubt; d’Orchymont, 1943d: 56], Costa Rica, French Guiana [in doubt; d’Orchymont, 1943d: 56], Guatemala, Lesser Antilles (Antigua), Mexico, Nicaragua, Paraguay, Uruguay; Fig. 6.

##### Natural history.

*Sindolus mundus* Sharp and *S. optatus* Sharp have been collected in stagnant waters at low elevations in dry areas; both species have been collected at mercury vapor lights in a drying lowland marsh where *S. optatus* Sharp was extremely abundant (Short 2005). Fernández and Kehr studied the annual life cycle (1994) and the spatial and temporal distribution (1995) of a population of *S. femoratus* in Argentina.

##### Larvae.

Immature stages are known for *Sindolus talarum* (Fernández) (as Helochares (Sindolus) talarum); egg case, first, second and third instar larvae and pupae were all described and illustrated by Fernández (1983). From each egg case between 25 and 40 larvae emerged; some larvae perforated and entered the aerenchyma of *Spirodella intermedia* (Araceae) and spent some time in there, apparently breathing the air stored in the plant tissues (Fernández 1983). In Argentina (Buenos Aires Province) first instar larvae start appearing in September, become abundant in October, and in November and the first two months of the summer all larval stages are abundant; at the end of March third instar larvae are the most common. Fernández (2004) also described the egg case and third instar larva of *Sindolus femoratus* (Brullé) (as Helochares (Sindolus) femoratus).

##### Taxonomic history.

Originally described as a genus by Sharp (1882) to accommodate two species from Central America; downgraded to subgenus of *Helochares* by d’Orchymont (1919c); Hansen (1991): designates type species.

##### Remarks.

There are eight species of *Sindolus* described. The genus is among the most easily recognized acidocerines in the New World.

##### Species examined.

*Sindolus femoratus* (Brullé), *S. mundus* Sharp, *S. optatus* Sharp. One of the available specimens of *S. mundus* had been previously compared wit the holotype by A. Shohrt.

##### Selected references.

Sharp 1882: original description of the genus and two species; Fernández 1981: description of two new species; Fernández 1983: description of immature stages for *Sindolus talarum* (Fernández); Fernández 2004: description of immature stages for *Sindolus femoratus* (Brullé); Short et al. 2021: phylogenetic placement.

#### Genus Tobochares

Taxon classificationAnimaliaColeopteraHydrophilidae

Short & García, 2007

Figs 1N, O , 2 , 6 , 11A, B , 52 , 53 , 54 , 55

Tobochares Short & García, 2007: 2.

##### Gender.

Masculine.

##### Type species.

*Tobochares sulcatus* Short & García, 2007: 4; by original designation.

##### Diagnosis.

**Figure 52.**
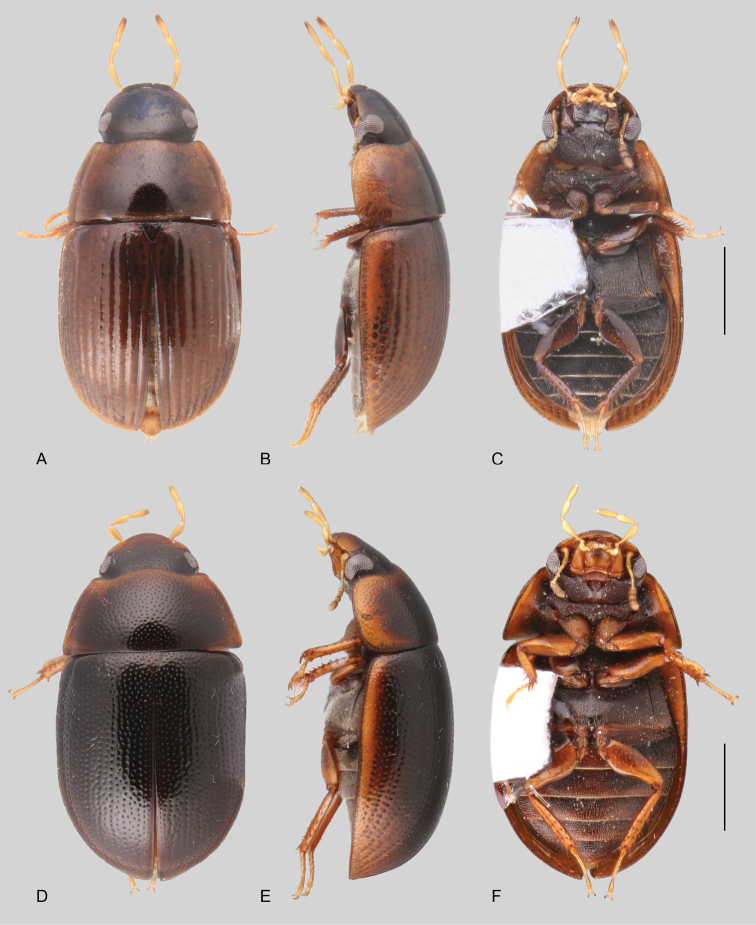
Habitus of *Tobochares* spp. **A–C***T. sulcatus*: **A** dorsal habitus **B** lateral habitus **C** ventral habitus **D–F***T. luteomargo*: **D** dorsal habitus **E** lateral habitus **F** ventral habitus. Scale bars: 0.5 mm.

**Figure 53.**
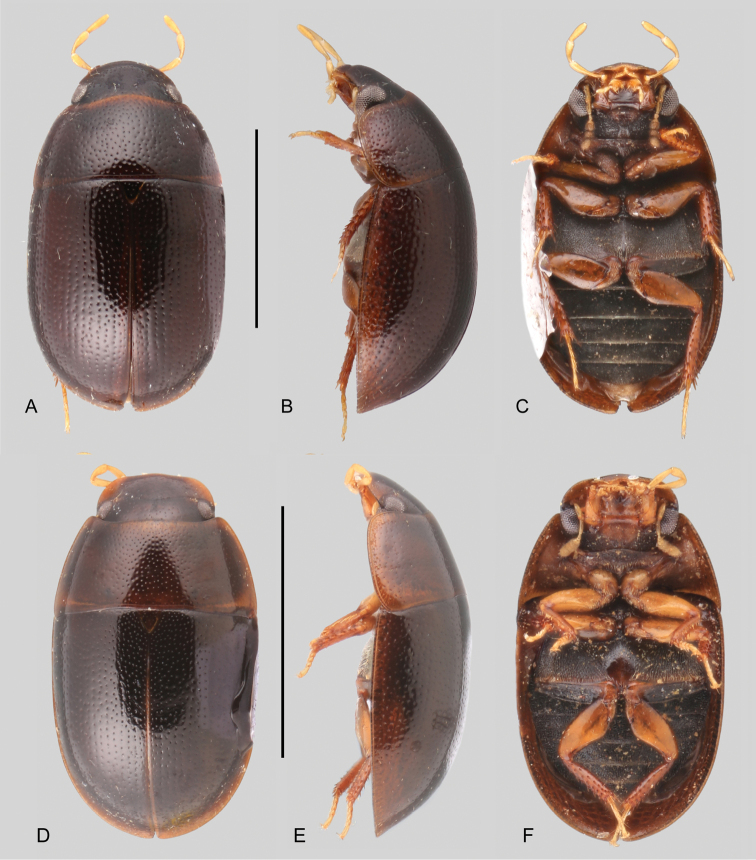
Habitus of *Tobochares* spp. **A–C***T. communis*: **A** dorsal habitus **B** lateral habitus **C** ventral habitus **D–F***T. fusus*: **D** dorsal habitus **E** lateral habitus **F** ventral habitus. Scale bars: 1 mm.

**Figure 54.**
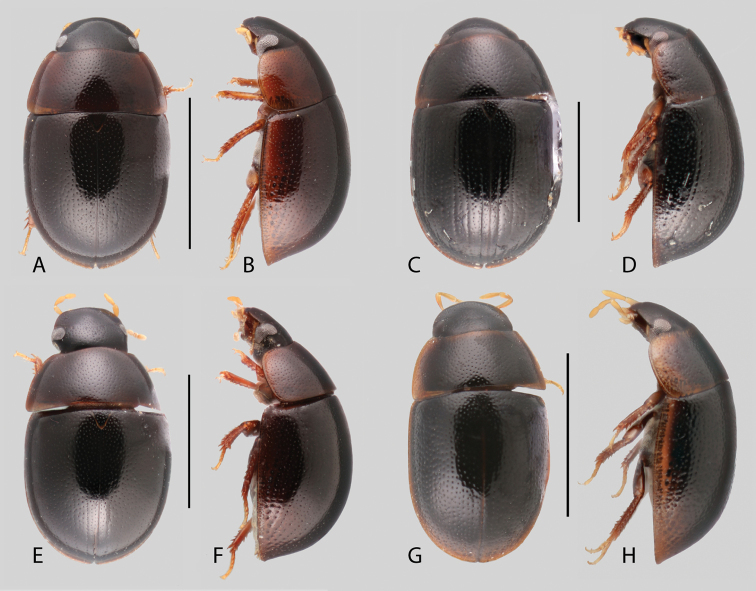
Habitus of *Tobochares* spp. **A, B***T. kappel*: **A** dorsal habitus **B** lateral habitus **C, D***T. akoerio*: **C** dorsal habitus **D** lateral habitus **E, F***T. kolokoe*: **E** dorsal habitus **F** lateral habitus **G, H***T. goias*: **G** dorsal habitus **H** lateral habitus. Scale bars: 1 mm.

**Figure 55.**
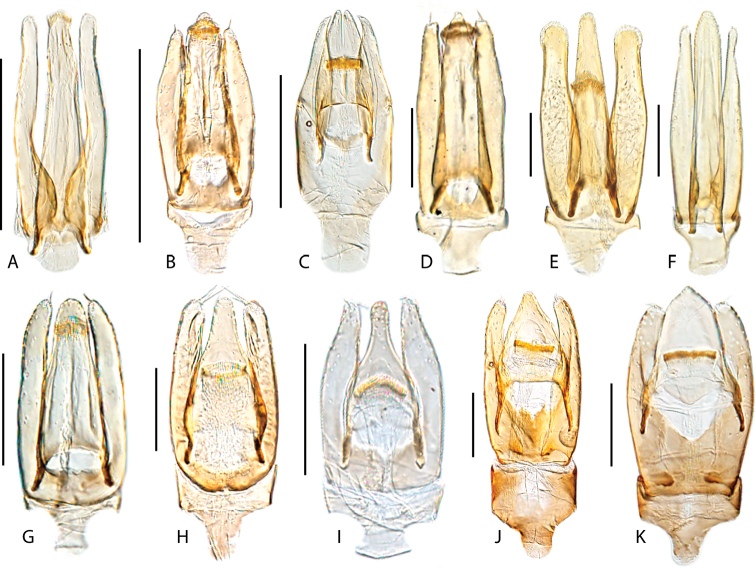
Aedeagi of *Tobochares* spp. **A***T. benettii***B***T. fusus***C***T. luteomargo***D***T. emarginatus***E***T. kusad***F***T. kasikasima***G***T. anthonyae***H***T. autures***I***T. communis***J***T. romanoae***K***T. akoreio*. Scale bars: 0.5 mm (**A–C**); 0.1 mm (**D–K**).

Small beetles, total body length 1.5–2.6 mm. Body shape oval in dorsal view; moderately to strongly convex in lateral view (Fig. 52–54); dorsal outline usually evenly curved. Color yellowish brown, orange brown to dark brown, sometimes with paler spots on head, or paler margins of pronotum and elytra; ground punctation moderate to shallow. Shape of head somewhat oval. Eyes not emarginate (e.g., Fig. 11A) to strongly emarginate (e.g., Fig. 11B), moderate to small in size, somewhat oval, slightly to strongly projected from outline of head. Clypeus trapezoid, with anterior margin broadly emarginate; membranous preclypeal area often visible. Labrum fully exposed, convex, and anteriorly emarginate. Mentum rather smooth, often medially depressed, or anteriorly shallowly crenulated; median anterior depression marked by transverse carina (e.g., Fig. 53C). Submentum anteriorly smooth and shiny. Antennae with eight antennomeres, cupule slightly asymmetric with rounded outline. Maxillary palps from short and slender (slightly shorter than the width of the head; e.g., Fig. 53C) to very short and stout (nearly half the width of the head; Fig. 54E); maxillary palpomere 4 similar in length to slightly longer than palpomere 3; inner margin of maxillary palpomere 2 straight, outer margin strongly curved along apical 2/3. Elytra without sutural striae (in some species, stria 1 more strongly impressed along posterior half of elytra; Fig. 54C); elytral punctures seemingly arranged in rows, in some species more pronounced; interserial punctures occasionally longitudinally aligned; serial punctures sometimes impressed into distinct grooves (e.g., Fig. 52A). Prosternum flat. Posterior elevation of mesoventrite either flat, bulging or with transverse or longitudinal ridge (Fig. 14F, G); anapleural sutures concave, separated at anterior margin by distance nearly 0.3–0.5 × width of anterior margin of mesepisternum. Metaventrite densely pubescent, except for median glabrous patch, either ovoid and broad (Fig. 14G) or longitudinal and narrow (Fig. 14F). Protibiae with spines of anterior row hair-like, semi erect, relatively long and thick; apical spurs of protibia from very short and stout, to enlarged to reach apex of protarsomere 3. Tarsomeres 2–4 densely covered by hair-like spines on ventral face; metatarsomeres 1–4 similar in length, 5 nearly as long as 3 and 4 combined, or metatarsomere 2 similar in length to 5. Metafemora mostly glabrous, with only few scattered setae, sometimes with hydrofuge pubescence along basal half of anterodorsal margin (e.g., Figs 52C, F, 53 C, F). Fifth abdominal ventrite apically evenly rounded, without fringe of stout setae. Aedeagus trilobed (Fig. 55), with basal piece usually very short (nearly 1/3 length of parameres); median lobe usually broader than each paramere; median lobe and parameres apically rounded to truncate; apex of median lobe seldom medially emarginated; gonopore well developed.

##### Differential diagnosis.

*Tobochares* are among the smallest acidocerines. Some members of the group are unique in the presence of impressed elytral striae (*striatus* species group; Girón and Short 2021a). *Tobochares* without elytral striae may resemble some *Agraphydrus* (with eight antennomeres and mostly glabrous femora), and other than their distributions (*Tobochares* in the New World, *Agraphydrus* in the Old World) and slight differences in overall body shape, they can only be differentiated by the shape of the aedeagus (slender in *Tobochares*, Fig. 55; overall broader in *Agraphydrus*, Fig. 20). Within the New World, *Tobochares* is most likely to be confused with *Ephydrolithus*, which also contains small, seepage-inhabiting species, although currently the ranges of the two genera do not quite overlap. However, the difference in the number of antennomeres (nine in *Ephydrolithus*) provides a clear point of separation.

##### Distribution.

**Neotropical**: Brazil (Amapá, Amazonas, Goiás, Roraima), French Guiana, Guyana, Suriname, Venezuela; Fig. 6.

##### Natural history.

Most *Tobochares* specimens have been collected at hygropetric habitats, including isolated hygropetric seeps as well as wet rock surfaces along rivers and waterfalls. They can sometimes be found in large numbers. One species, *T. fusus*, has been collected in both seepage habitats as well as terrestrially in the rotten fruits of *Clusia* (see Kohlenberg and Short 2017 and Girón and Short 2021a for more details).

##### Larvae.

The immature stages of *Tobochares* remain unknown.

##### Taxonomic history.

Short and García (2007) described the genus and one species from Venezuela. Additional species were described from Suriname, one by Short and Kadosoe (2011) and two more by Short (2013). The genus was revised by Kohlenberg and Short (2017), including the description of five new species and the characterization of one specimen from Tobogán de la Selva (Venezuela) left undescribed until additional material can be studied. The genus was reviewed again just a few years later by Girón and Short (2021), in the light of new molecular evidence, describing 15 additional new species and establishing four diagnosable species groups.

##### Remarks.

There are 24 described species of *Tobochares*. The genus is rather highly variable in its external morphology: there is variation in coloration, the degree of emargination of the eyes and the degree of development and extension of the elytral striae. The form of the aedeagus is also somewhat variable, although not as extreme as in some genera such as *Chasmogenus* or *Helochares*.

The genus is much richer in species and more broadly distributed in the Amazon region than as currently published. We have examined numerous additional specimens from around the Amazonian region, particularly the southern Amazon (e.g., Brazil: Rondonia) from where the genus is currently unknown. We would not be surprised if the genus exceeded 50 species when more attention is paid to seepage habitats in this region.

##### Species examined.

Holotypes, paratypes, and additional specimens of all described species, as well as several undescribed species were examined for this study.

##### Selected references.

Short and García 2007: original description of the genus and its type species; Short and Kadosoe 2011: description of one additional species; Short 2013: description of two additional species; Kohlenberg and Short 2017: revision of the genus and description of five new species; Girón and Short 2021a: review of the genus with description of 15 new species and establishment of four species groups; Short et al. 2021: phylogenetic placement.

#### Genus Troglochares

Taxon classificationAnimaliaColeopteraHydrophilidae

Spangler, 1981

Figs 6 , 56

Troglochares Spangler, 1981a: 316.

##### Gender.

Masculine.

##### Type species.

*Troglochares ashmolei* Spangler, 1981a: 318; by original designation and monotypy.

##### Diagnosis.

**Figure 56.**
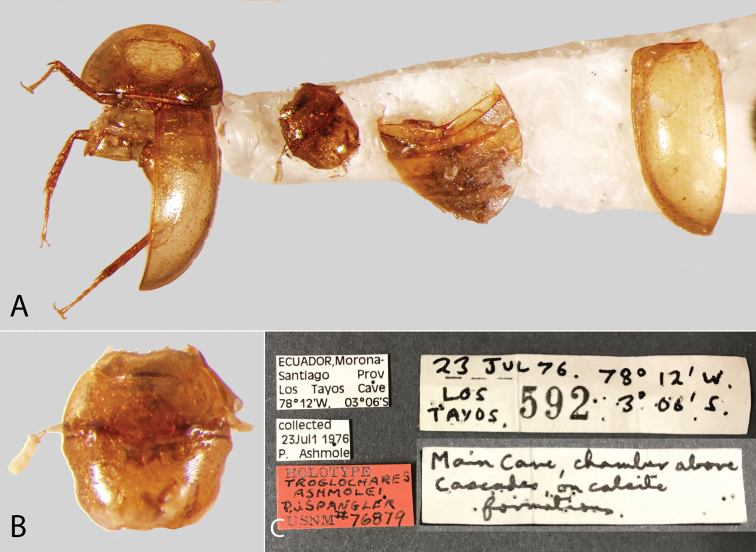
Holotype and labels of *Troglochares ashmolei***A** mount of holotype **B** head, dorsal view **C** labels.

Small beetles, body length 1.9 mm. Body shape oval in dorsal view; moderately convex in lateral view (Hansen 1991: fig. 39). Color yellowish light brown; ground punctation extremely shallowly marked. Shape of head somewhat oval. Eyes absent (Fig. 56B). Clypeus trapezoidal, with anterior margin broadly emarginate, with medial region of emargination nearly straight (Fig. 56B). Labrum fully exposed, convex. Mentum rather smooth and antero-medially depressed; median anterior depression broad. Antennae with nine antennomeres (Spangler 1981a: fig. 3); cupule slightly asymmetric, with rounded outline. Maxillary palps slender, nearly as long as width of head; inner margin of maxillary palpomere 2 nearly straight, outer margin curved along apical third; maxillary palpomere 3 slightly shorter than 4. Prosternum non carinate, slightly convex. Elytra without sutural striae; ground punctation fine, shallow; outer margins slightly flared (Fig. 56A). Posterior elevation of mesoventrite with curved, transverse ridge (Spangler 1981a: fig. 8); anapleural sutures concave, separated at anterior margin by distance 0.7 × width of anterior margin of mesepisternum. Metaventrite densely pubescent except for median short and narrow posterior glabrous patch; metaventrite short (nearly as long as first abdominal ventrite; Spangler 1981a: fig. 8). Protibiae with spines of anterior row long; apical spurs of protibiae moderately slender, reaching apex of protarsomere 2; metatarsomeres 2–4 slightly decreasing in size; metatarsomere 5 nearly as long as 2–4 combined. Posterior femora densely covered by hydrofuge pubescence along basal 2/3 (Spangler 1981a: fig. 8). Fifth abdominal ventrite apically truncate, without stout setae (Spangler 1981a: fig. 9).

##### Differential diagnosis.

*Troglochares* is the only genus of acidocerines (and Hydrophilids) lacking eyes.

##### Distribution.

**Neotropical**: Ecuador; Fig. 6.

##### Natural history.

The only known specimen was collected in a cave on calcite formations and is presumably aquatic (Spangler 1981a).

##### Larvae.

The immature stages are unknown for *Troglochares*.

##### Taxonomic history.

The genus and its only known species were described by Spangler (1981a).

##### Remarks.

The genus is only known from a single female specimen, which is pin-mounted in pieces (Fig. 56). This species was not included in the molecular phylogeny by Short et al. (2021). Its assignment to the *Tobochares* group is based primarily on its tiny size (excluding the *Helochares* group), presence in the Neotropical region (excluding the *Agraphydrus* group), and lack of a sutural stria (excluding the *Primocerus* and *Chasmogenus* groups).

##### Species examined.

The holotype specimen of *Troglochares ashmolei* Spangler was examined.

##### Selected references.

Spangler 1981a: original description; Short et al. 2021: morphological affinities discussed in a phylogenetic context.

## Catalog of the subfamily Acidocerinae

The following species list is based for the most part on Hansen (1999b), and therefore follows its format. Species described between 15 December 1999 and 1 April 2021 have been added to the present catalog. Generic synonyms are omitted here as those are listed for each genus above. For each species the currently valid name is provided, followed by the original name with a reference to the original description, including page number and full type locality as provided in the original publication. The full checklist of valid names is available online via GBIF (https://doi.org/10.15468/ypcrsp). For countries which current names are different from those indicated in the original description the name of the country has been updated, leaving in square brackets the country names that have been previously cited (e.g., Sri Lanka [Ceylon]).

For each name that has been used, a list of references including page number and details on the nature/content of the reference in square brackets (e.g., [catalog], [checklist], [new record], etc.) is also provided. ‘Catalog’ refers to publications listing synonyms and references, whereas ‘checklist’ only presents the name of a species for a particular region. ‘Faunistic treatment’ is used for works revising the fauna of a particular country or region, which sometimes include discussions on taxonomic status of certain species, whereas ‘taxonomic treatment’ is used when the reference includes a taxonomic revision for a particular group. ‘New record’ is used for new country records, as opposed to new localities from a previously recorded country. The currently known distribution (extracted from the literature) is summarized for each valid name.

### *Acidocerus* Klug, 1855

***Acidocerus aphodioides* Klug, 1855**

*Acidocerus aphodioides* Klug, 1855: 649 – Mozambique, Tete [“Mossambique: Tette”]; Knisch 1924: 222 [catalog]; Hansen 1999b: 158 [catalog]; Hebauer 2006a: 25 [checklist].

Distribution: Afrotropical: Mozambique.

### *Agraphydrus* Régimbart, 1903

***Agraphydrus abrasus* Komarek & Freitag, 2020**

*Agraphydrus* sp. D (in part); Freitag and Zettel 2013: 19, 30.

*Agraphydrus abrasus* Komarek & Freitag, 2020: 204 – Philippines, Luzon Island, Aurora Province, Maria Aurora Municipality, Barangay Wenceslao, Bingwangan River flowing through extensive coconut plantation, 60 m a.s.l., 15°45'48"N, 121°25'21"E.

Distribution: Indo-Malayan: Philippines (Luzon, Mindoro, Palawan).

***Agraphydrus activus* Komarek & Hebauer, 2018**

*Agraphydrus activus* Komarek & Hebauer, 2018: 18 – China, Hong Kong Admin. Reg., New Territories, Tai Mo Shan Country Park, SW Tai Po New Town, Lam Tsuen River; Komarek 2019: 157 [new record].

Distribution: Indo-Malayan: China (Fujian, Hong Kong, Guangdong, Jiangxi). Palearctic: China (Anhui), Thailand.

***Agraphydrus acutus* Komarek, 2020**

*Agraphydrus acutus* Komarek, 2020: 132 – Namibia, Karas Region, Aar Farm Waterhole.

Distribution: Afrotropical: Namibia, Republic of South Africa.

***Agraphydrus aethiopicus* Komarek, 2020**

*Agraphydrus aethiopicus* Komarek, 2020: 134 – Ethiopia, Amhara Region, Simien Mountains.

Distribution: Afrotropical: Ethiopia.

***Agraphydrus agilis* Komarek & Hebauer, 2018**

*Agraphydrus agilis* Komarek & Hebauer, 2018: 20 – China, Guangxi Province, Liuzhou Prefecture, 10 km N Liuzhou City, ca. 2 km E Shanmenjiang Forest Station; Komarek 2019: 158 [taxonomic treatment].

Distribution: Indo-Malayan: China (Guangxi, Yunnan), Vietnam.

***Agraphydrus albescens* (Régimbart, 1903)**

*Helochares albescens* Régimbart, 1903a: 27 – Madagascar, “Centre-Sud”.

Helochares (s. str.) albescens Régimbart, 1903; Knisch 1924a: 196 [catalog].

Helochares (Agraphydrus) albescens Régimbart; d’Orchymont 1939c: 198 [taxonomic discussion; new record].

Agraphydrus (Agraphydrus) albescens (Régimbart, 1903); Hansen 1999b: 156 [new combination; catalog]; Hebauer 2006a: 27 [checklist, new records].

*Agraphydrus albescens* (Régimbart, 1903); Hebauer 2005: 39 [checklist]; Komarek 2020: 135 [faunistic treatment; lectotype designation; new records].

Distribution: Afrotropical: Botswana, Cameroon, Democratic Republic of the Congo, Kenya, Madagascar, Malawi, Namibia, Republic of South Africa, Tanzania [Zanzibar], Zimbabwe. Sudan is excluded (Komarek 2020).

***Agraphydrus ampullatus* Komarek & Freitag, 2020**

*Agraphydrus ampullatus* Komarek & Freitag, 2020: 206 – Philippines, Leyte Island and Province, Baybay Municipality, creek 2 km east of Visayas State University, ca. 10°44'46"N, 124°48'50"E, ca. 140 m a.s.l.

Distribution: Indo-Malayan: Philippines (Leyte).

***Agraphydrus anacaenoides* Komarek, 2019**

*Agraphydrus anacaenoides* Komarek, 2019: 158 – Malaysia, Penang, Southwest Penang Island District, Pantai Aceh Forest Reserve (= Penang N.P.).

Distribution: Indo-Malayan: Malaysia.

***Agraphydrus anatinus* Komarek, 2018**

*Agraphydrus anatinus* Komarek, 2018: 107 – India, Goa, South Goa District, Salcete (= Salcette or Saxti) Subdivision.

Distribution: Indo-Malayan: India (Goa, Kerala, Maharashtra).

***Agraphydrus andamanicus* Komarek, 2018**

*Agraphydrus andamanicus* Komarek, 2018: 108 – India, North Andaman Island, Diglipur.

Distribution: Indo-Malayan: India (North Andaman Island).

***Agraphydrus andringitra* Komarek, 2020**

*Agraphydrus andringitra* Komarek, 2020: 137 – Madagascar, Fianarantsoa Province, Haute Matsiatra Region, Andringitra N.P., Mount Ambatoberger, 22°7'52.0"S, 46°51'51.1"E.

Distribution: Afrotropical: Madagascar.

***Agraphydrus angulatus* Komarek, 2019**

*Agraphydrus angulatus* Komarek, 2019: 159 – Laos, Khammouan Province, Nakai District, Nakai, 17°43'N, 105°09'E.

Distribution: Indo-Malayan: Laos.

***Agraphydrus angustatus* Komarek, 2020**

*Agraphydrus angustatus* Komarek, 2020: 138 – Namibia, Kunene Region, Uniab River, Palmwag N.P., near Palmwag Lodge, 19°53'S, 13°50'W.

Distribution: Afrotropical: Angola, Namibia.

***Agraphydrus angustipenis* Komarek, 2018**

*Agraphydrus angustipenis* Komarek, 2018: 109 – Sri Lanka, “Dambuwa Estate”.

Distribution: Indo-Malayan: Sri Lanka.

***Agraphydrus anhuianus* (Hebauer, 2000)**

*Megagraphydrus anhuianus* Hebauer, 2000: 15 – China, Anhui, Huang Shan 30 km W Tunxi. Hansen 2004: 52 [catalog]; Short and Hebauer 2006: 337 [catalog]; Fikáček et al. 2015: 62 [catalog].

Agraphydrus (Agraphydrus) anhuianus (Hebauer, 2000); Minoshima et al. 2015: 12 [new combination; redescription; new record].

*Agraphydrus anhuianus* (Hebauer, 2000); Komarek and Hebauer 2018: 21 [excludes only known specimen from Hong Kong].

Distribution: Indo-Malayan: Thailand. Palearctic: China (Anhui).

***Agraphydrus annapurnensis* Komarek, 2018**

*Agraphydrus annapurnensis* Komarek, 2018: 110 – Nepal, Western Region, Gandaki Zone, Kaski District, Annapurna Mountains, ca. 10 km ENE Pokhara, tributary of Madi Khola River below Kwinkal (village), ca. 28°13'55"N, 84°5'16"E.

Distribution: Indo-Malayan: Nepal.

***Agraphydrus arduus* Komarek & Hebauer, 2018**

*Agraphydrus arduus* Komarek & Hebauer, 2018: 22 – China Yünnan Prov., Xishuangbanna Dai Autonomous Prefecture, Mengla County, Wushiwu He River, ca. 10 km NW Menglun Town; Komarek 2019: 160 [new record].

Distribution: Indo-Malayan: China (Guangdong, Yunnan), Laos. Palearctic: China (Hubei).

***Agraphydrus ater* Komarek, 2018**

*Agraphydrus ater* Komarek, 2018: 111 – Nepal, Western Region, Gandaki Zone, Annapurna, N Pokhara, Kali Khola, below Garlang, ca. 28°17'10"N, 83°59'39"E.

Distribution: Indo-Malayan: Nepal.

***Agraphydrus atripalpis* Komarek, 2020**

*Agraphydrus atripalpis* Komarek, 2020: 139 – Republic of South Africa: KwaZulu-Natal Province, Port Shepstone, Oribi Gorge.

Distribution: Afrotropical: Republic of South Africa.

***Agraphydrus attenuatus* (Hansen, 1999)**

*Megagraphydrus attenuatus* Hansen, 1999a: 141 – Vietnam, Vĩnh Phúc Province (N Viertnam), Tam Dao. Hansen 1999b: 157 [catalog]; Hebauer 2000: 15 [taxonomic treatment].

Agraphydrus (Agraphydrus) attenuatus (Hansen, 1999); Minoshima et al. 2015: 16 [new combination; redescription; new records].

*Agraphydrus attenuatus* (Hansen, 1999); Komarek and Hebauer 2018: 23 [redescription]; Komarek 2019: 161 [taxonomic treatment].

Distribution: Indo-Malayan: China (Yunnan), Laos, Vietnam.

***Agraphydrus audax* Komarek & Hebauer, 2018**

*Agraphydrus audax* Komarek & Hebauer, 2018: 24 – China Hunan Prov., Xiangxi Prefecture; Dayong County; Zhangjiajie Forest National Park, Suoxiyü Nature Reserve, Wulingyüan section, 30 km N Dayong City.

Distribution: Indo-Malayan: China (Guizhou, Hunan). Palearctic: China (Hubei, Shaanxi, Sichuan).

***Agraphydrus avita* (Hansen, 1997)**

*Horelophopsis avita* Hansen, 1997: 109 – Indonesia, Papua [New Guinea; Irian Jaya], Japen Island, SSE Sumberbaba, Dawai R. Hansen 1999b: 68 [catalog].

*Agraphydrus avita* (Hansen); Short et al. 2021: 11 [new combination].

Distribution: Australasian: Indonesia (Papua (Yapen Island)).

***Agraphydrus bacchusi* Komarek, 2019**

*Agraphydrus bacchusi* Komarek, 2019: 162 – Papua New Guinea, Central Province, road between Port Moresby and Brown River.

Distribution: Australasian: Papua New Guinea (Central Province).

***Agraphydrus balkeorum* Komarek, 2019**

*Agraphydrus balkeorum* Komarek, 2019: 163 – West Sumatra Province, Solok Regency, Solok – Alahan Panjang road, ca. 0°56'20"S, 100°46'24"E.

Distribution: Indo-Malayan: Indonesia (Sumatra).

***Agraphydrus batak* Komarek & Freitag, 2020**

*Agraphydrus* sp. I; Freitag and Zettel 2013: 20, 30.

*Agraphydrus batak* Komarek & Freitag, 2020: 208 – Philippines, Palawan Island and Province, Puerto Princesa City, Barangay Concepcion, Tarabanan River upstream of Batak village, secondary forest, ca. 100 m a.s.l., 10°2'7"N, 119°1'10"E.

Distribution: Indo-Malayan: Philippines (Palawan Island).

***Agraphydrus bhutanensis* Komarek, 2018**

*Agraphydrus bhutanensis* Komarek, 2018: 113 – Bhutan, Sarpang Province, 11 km NW Sarpang, Bhur Khola, 26°55'23"N, 90°23'51"E.

Distribution: Indo-Malayan: Bhutan.

***Agraphydrus bicoloratus* Komarek, 2020**

*Agraphydrus bicoloratus* Komarek, 2020: 141 – Gabon, Estuaire Province, near Kinguélé Waterfall.

Distribution: Afrotropical: Gabon.

***Agraphydrus bilardoi* Komarek, 2020**

*Agraphydrus bilardoi* Komarek, 2020: 142 – Gabon, Ngounié Province, Ndolou Distr., near Mandji, Pény Village, 2°1.804'S, 10°29.372'E.

Distribution: Afrotropical: Gabon.

***Agraphydrus biltoni* Komarek, 2020**

*Agraphydrus biltoni* Komarek, 2020: 143 – Republic of South Africa: Northern Cape Province, Kamiesberg, 30°23'43.0"S, 18°8'8.4"E.

Distribution: Afrotropical: Republic of South Africa.

***Agraphydrus biprojectus*** Minoshima, Komarek & Ôhara, 2015

Agraphydrus (Agraphydrus) biprojectus Minoshima, Komarek & Ôhara, 2015: 36 – Vietnam, Lào Cai Province, Sa Pa, Ô Quy Hồ; Komarek 2019: 164 [taxonomic treatment].

Distribution: Indo-Malayan: Laos, Vietnam.

***Agraphydrus borneensis* Komarek, 2019**

*Agraphydrus borneensis* Komarek, 2019: 165 – Malaysia, Sabah, West Coast Division, Kota Kinabalu District, Crocker Range, km 56 of road Kota Kinabalu – Tambunan, near Sunsuron Waterfall.

Distribution: Indo-Malayan: Malaysia (Borneo).

***Agraphydrus boukali* Komarek, 2018**

*Agraphydrus boukali* Komarek, 2018: 114 – India, Kerala, Thiruvananthapuram District, Cardamom Hills, 50 km NW Pathanamthitta, near Pambaiyar River, ca. 9°25'N, 77°05'E.

Distribution: Indo-Malayan: India (Kerala, Karnataka, Tamil Nadu).

***Agraphydrus brevilobatus* Komarek & Freitag, 2020**

*Agraphydrus brevilobatus* Komarek & Freitag, 2020: 209 – Philippines, Negros Occidental, Silay, Patag, small mountain river, downstream Dumalabdab Falls, secondary forest, 800 m a.s.l., 10°41'10"N, 123°10'43"E.

Distribution: Indo-Malayan: Philippines (Negros, Panay).

***Agraphydrus brevipenis* Komarek, 2019**

*Agraphydrus brevipenis* Komarek, 2019: 167 – Malaysia, Pahang, Cameron Highlands District, Mt. Jasar.

Distribution: Indo-Malayan: Malaysia.

***Agraphydrus burmensis* Komarek, 2019**

*Agraphydrus burmensis* Komarek, 2019: 168 – Myanmar, Mandalay Region, Pyin Oo Lwin District, Mogok Township, NW Mogok, S Panlin village, west slope of Mt. Taung Mae, 22°57'57"N, 96°27'29"E.

Distribution: Indo-Malayan: Myanmar.

***Agraphydrus calvus* Komarek & Hebauer, 2018**

*Agraphydrus calvus* Komarek & Hebauer, 2018: 25 – China, Hong Kong Admin. Reg., New Territories, Tai Mo Shan Country Park, SW Tai Po New Town, Lam Tsuen River.

Distribution: Indo-Malayan: China (Guangdong, Guangxi, Hong Kong, Jiangxi).

***Agraphydrus camerunensis* Komarek, 2020**

*Agraphydrus camerunensis* Komarek, 2020: 144 – Cameroon, Southwest Region, 25 km west of Limbe (City), Bakingili.

Distribution: Afrotropical: Cameroon.

***Agraphydrus cantonensis* Komarek & Hebauer, 2018**

*Agraphydrus cantonensis* Komarek & Hebauer, 2018: 27 – China, Guangdong Prov., Zhaoqing Pref., Fengkai County, ca. 50 km E of Fengkai, ca. 5 km W of Qixing, Heishiding Nature Reserve, 23°27'04"N, 111°53'53"E.

Distribution: Indo-Malayan: China (Guangdong).

***Agraphydrus carinatulus* Komarek, 2019**

*Agraphydrus carinatulus* Komarek, 2019: 169 – Indonesia, East Kalimantan Province, Kutai Kartanegara Regency, Tabang District, ca. 200 km NW of Samarinda City near Ritan Baru village.

Distribution: Indo-Malayan: Indonesia.

***Agraphydrus cervus* Komarek, 2019**

*Agraphydrus cervus* Komarek, 2019: 170 – Malaysia, Sarawak, Kapit Division, Kapit District, ca. 25 km E of Kapit.

Distribution: Indo-Malayan: Malaysia (Borneo).

***Agraphydrus ceylonensis* Komarek, 2018**

*Agraphydrus ceylonensis* Komarek, 2018: 115 – Sri Lanka [Ceylon], Sabaragamuwa Province, Kegalle District, a few km E Kitulgala.

*Helochares* sp.: Jäch 1984: 243.

Distribution: Indo-Malayan: Sri Lanka.

***Agraphydrus chinensis* Komarek & Hebauer, 2018**

*Agraphydrus chinensis* Komarek & Hebauer, 2018: 27 – China, Fujian Prov., Jianyuan Prefecture, Chong’an City Region, Chong’an Wuyi Shan.

Distribution: Indo-Malayan: China (Fujian, Zhejiang). Palearctic: China (Anhui).

***Agraphydrus cinnamum* Komarek, 2018**

*Agraphydrus cinnamum* Komarek, 2018: 117 – India, Kerala, Thiruvananthapuram District, Cardamom Hills, 50 km NW Pathanamthitta, near Pambaiyar River, ca. 9°25'N, 77°05'E.

Distribution: Indo-Malayan: India (Kerala).

***Agraphydrus clarus* Komarek, 2019**

*Agraphydrus clarus* Komarek, 2019: 171 – Malaysia, Sabah, West Coast Division, Kota Kinabalu District, Crocker Range, km 56 of road between Kota Kinabalu and Tambunan, near Sunsuron Waterfall.

Distribution: Indo-Malayan: Malaysia (Borneo).

***Agraphydrus comes* Komarek & Hebauer, 2018**

*Agraphydrus comes* Komarek & Hebauer, 2018: 28 – China, Hainan Prov., Ledong County, foot of Jianfeng Mountain, ca. 4 km E Jianfeng Town.

Distribution: Indo-Malayan: China (Hainan).

***Agraphydrus communis* Komarek, 2018**

*Agraphydrus communis* Komarek, 2018: 118 – Nepal, Central Region, Sindhupalchok District, torrent above Tatobani near Kodari.

Distribution: Indo-Malayan: Bhutan, Nepal, India (Uttarakhand).

***Agraphydrus confusus* Komarek & Hebauer, 2018**

*Agraphydrus confusus* Komarek & Hebauer, 2018: 29 – China, Hong Kong Admin. Reg., Tai Po Kau Nature Reserve; Komarek 2019: 173 [new record].

Distribution: Indo-Malayan: China (Guizhou, Hong Kong, Yunnan), Laos, Vietnam.

***Agraphydrus congolensis* Komarek, 2020**

*Agraphydrus congolensis* Komarek, 2020: 145 – Democratic Republic of the Congo, Ituri (former Orientale) Province, Ituri Rainforest, Epulu River.

Distribution: Afrotropical: Democratic Republic of the Congo.

***Agraphydrus conicus* Komarek & Hebauer, 2018**

*Agraphydrus conicus* Komarek & Hebauer, 2018: 30 – China Jiangxi Prov., Jinggangshan Mountains, Jingzhushan, 26°31.0'N, 114°05.9'E.

Distribution: Indo-Malayan: China (Hunan, Jiangxi). Palearctic: China (Anhui).

***Agraphydrus connexus* Komarek & Hebauer, 2018**

*Agraphydrus connexus* Komarek & Hebauer, 2018: 31 – Malaysia, Pahang, Kuala Lipis [Town] surround. Komarek 2018: 120 [new records]; Komarek 2019: 173 [taxonomic treatment].

Distribution: Indo-Malayan: Bhutan, China (Hainan), India (Madhya Pradesh), Laos, Malaysia, Myanmar, Nepal, Thailand, Vietnam.

***Agraphydrus constrictus* Komarek, 2018**

*Agraphydrus constrictus* Komarek, 2018: 121 – India, Uttarakhand, Chamoli District, Nandakini River, below Sedoli, ca. 10 km E Nandaprayag, 30°15'50"N, 79°26'32"E.

Distribution: Indo-Malayan: India (Assam, Uttarakhand), Nepal.

***Agraphydrus contractus* Komarek & Hebauer, 2018**

*Agraphydrus contractus* Komarek & Hebauer, 2018: 33 – China, Fujian Prov., Jianyuan Prefecture; Yong’an City Region; ca. 20 km SE Yong’an City, 5 km SW Xiyang Village, Ziyungdong Shan.

Distribution: Indo-Malayan: China (Fujian, Guangdong).

***Agraphydrus coomani* (d’Orchymont, 1927)**

Helochares (Agraphydrus) coomani d’Orchymont, 1927a: 248 – Vietnam, [Tonkin], Lac Tho, nr. Hoa Binh Province; d’Orchymont 1928: 108 [faunistic treatment].

*Agraphydrus coomani* (d’Orchymont, 1927); Watts 1995: 115 [new records]; Komarek and Hebauer 2018: 34 [new records; redescription]; Komarek 2018: 122 [new records]; Komarek 2019: 174 [new records]; Komarek and Freitag 2020: 211 [new records].

Agraphydrus (Agraphydrus) coomani (d’Orchymont, 1927); Hansen 1999b: 156 [catalog].

*Enochrus ryukyuensis* Matsui, 1994: 217 – Japan, Amami Islands (Kagoshima Pref.), Tokuno-shima Is., Tokunoshima Town, Kamize Dam.

*Agraphydrus ryukyuensis* (Matsui, 1994); Gentili et al. 1995: 208 [checklist]; Komarek and Hebauer 2018: 34 [synonym of *A. coomani* (d’Orchymont, 1927)].

Agraphydrus (Agraphydrus) ryukyuensis (Matsui, 1994); Hansen 1999b: 157 [catalog]; Hansen 2004: 49 [checklist]; Fikáček et al. 2015: 61 [catalog]; Minoshima 2016: 361 [redescription].

Distribution: Indo-Malayan: Brunei, China (Fujian, Guangdong, Hainan, Taiwan), Indonesia, Laos, Malaysia (Peninsula), Myanmar, Philippines (Leyte, Luzon), Sri Lanka, Thailand, Vietnam. Palearctic: Japan. Australasian: Australia (New South Wales, Northern Territory, Queensland, Western Australia), Papua New Guinea.

***Agraphydrus coronarius* Minoshima, Komarek & Ôhara, 2015**

Agraphydrus (Agraphydrus) coronarius Minoshima, Komarek & Ôhara, 2015: 41 – Laos, Bolikhamsai Province, Lak Sao; Komarek 2019: 179 [taxonomic treatment].

Distribution: Indo-Malayan: Laos.

***Agraphydrus crassipenis* Komarek, 2018**

*Agraphydrus crassipenis* Komarek, 2018: 123 – Nepal, Eastern Region, Kosi (= Koshi) Zone, Sunsari District, Dharan (city) environment.

Distribution: Indo-Malayan: Bhutan, Nepal.

***Agraphydrus decipiens* Minoshima, Komarek & Ôhara, 2015**

Agraphydrus (Agraphydrus) decipiens Minoshima, Komarek & Ôhara, 2015: 44 – Taiwan, Taichung City, Heping District, Basian-shan National Forest Recreation Area, 24°11.55'N, 121°00.83'E.

*Agraphydrus decipiens* Minoshima, Komarek & Ôhara, 2015; Komarek and Hebauer 2018: 36 [redescription]; Angus et al. 2020: 19 [karyotype].

Distribution: Indo-Malayan: China (Taiwan).

***Agraphydrus delineatus* Komarek, 2019**

*Agraphydrus delineatus* Komarek, 2019: 180 – Malaysia, Sarawak, Kuching Division, Mt. Serapi, ca. 19 km W Kuching.

Distribution: Indo-Malayan: Malaysia (Borneo).

***Agraphydrus elongatus* Ribera, Hernando & Cieslak, 2019**

*Agraphydrus elongatus* Ribera, Hernando & Cieslak, 2019: 264 – Oman, Murri, Wadi Bani Ghafir, N23 29 46.2 E56 53 34.8; Komarek 2020: 146 [faunistic treatment].

Distribution: Afrotropical: Oman, United Arab Emirates.

***Agraphydrus engkari* Komarek, 2019**

*Agraphydrus engkari* Komarek, 2019: 181 – Malaysia, Sarawak, Sri Aman Division, Lubok Antu District, Batang Ai N.P., E of Bandar Sri Aman, Engkari River.

Distribution: Indo-Malayan: Malaysia (Borneo).

***Agraphydrus excisus* Komarek, 2019**

*Agraphydrus excisus* Komarek, 2019: 182 – Malaysia, Sarawak, Kapit Division, Kapit District, ca. 25 km of E Kapit.

Distribution: Indo-Malayan: Malaysia (Borneo).

***Agraphydrus exedis* (d’Orchymont, 1937)**

Helochares (Agraphydrus) exedis d’Orchymont, 1937a: 29 – India, Maharashtra [Bombay Presidency], Pune distr. [“Poona distr.”], Khandala.

Agraphydrus (Agraphydrus) exedis (d’Orchymont, 1937); Hansen 1999b: 156 [new combination].

*Agraphydrus exedis* (d’Orchymont, 1937); Komarek 2018: 124 [new records].

Distribution: Indo-Malayan: India (Madhya Pradesh, Maharashtra).

***Agraphydrus exiguus* Komarek, 2019**

*Agraphydrus exiguus* Komarek, 2019: 183 – Malaysia, Pahang, Cameron Highlands District, Tanah Rata (town), Sungai Ruil near village of Orang Asli.

Distribution: Indo-Malayan: Malaysia (Peninsula).

***Agraphydrus falcatus* Komarek, 2018**

*Agraphydrus falcatus* Komarek, 2018: 125 – India, Tamil Nadu, Dindigul District, Palni Hills, Kodaikanal, Pallangi, ca. 10°15'N, 77°30'E.

Distribution: Indo-Malayan: India (Kerala, Tamil Nadu).

***Agraphydrus fasciatus* Komarek & Hebauer, 2018**

*Agraphydrus fasciatus* Komarek & Hebauer, 2018: 37 – China, Hong Kong Admin. Reg., New Territories, Plover Cove Reservoir.

Distribution: Indo-Malayan: China (Guangdong, Hong Kong, Jiangxi).

***Agraphydrus fikaceki* Komarek & Hebauer, 2018**

*Agraphydrus fikaceki* Komarek & Hebauer, 2018: 38 – China, Jiangxi Prov., Jinggangshan Mts., Pingshui Shan, 26°30.4'N, 114°06.9'E.

Distribution: Indo-Malayan: China (Hong Kong, Jiangxi).

***Agraphydrus flavescens* Komarek, 2020**

*Agraphydrus flavescens* Komarek, 2020: 147 – Ghana, Ashanti Region, Bobiri Forest Reserve.

Distribution: Afrotropical: Cameroon, Ghana.

***Agraphydrus flavipes* Komarek, 2020**

*Agraphydrus flavipes* Komarek, 2020: 148 – Madagascar, Fianarantsoa Province, Vatovavy-Fitovinany Region, Ionilahy (village), Ionilahy River.

Distribution: Afrotropical: Madagascar.

***Agraphydrus flavonotus* Komarek, 2018**

*Agraphydrus flavonotus* Komarek, 2018: 127 – Bhutan, Sarpang Province, Geylephug – Shemgang road, 26°56'43"N, 90°31'29"E.

Distribution: Indo-Malayan: Bhutan.

***Agraphydrus floresinus* Komarek, 2019**

*Agraphydrus floresinus* Komarek, 2019: 185 – Indonesia, East Nusa Tenggara Province, East Manggarai Regency, Borong District, Flores Island, Lake Ranamese, between Ruteng and Borong.

Distribution: Indo-Malayan: Indonesia (Flores).

***Agraphydrus fontis* Komarek, 2020**

*Agraphydrus fontis* Komarek, 2020: 149 – Madagascar, Fianarantsoa Province, Atsimo-Atsinanana Region, Ranomena (town), 21°29'45.9"S, 47°24'7.5"E.

Distribution: Afrotropical: Madagascar.

***Agraphydrus forcipatus* Komarek & Hebauer, 2018**

*Agraphydrus forcipatus* Komarek & Hebauer, 2018: 39 – China, Anhui Prov., Weizhou Prefecture; Huang Shan NP; 60 km NNW Huang Shan City (= Tunxi), near Tang Kou.

Distribution: Indo-Malayan: (Fujian, Guangdong, Guizhou, Hunan, Jiangxi, Zhejiang). Palearctic: China (Anhui, Hubei).

***Agraphydrus fortis* Komarek, 2018**

*Agraphydrus fortis* Komarek, 2018: 128 – Sri Lanka [Ceylon], Uva Province, Monaragala District, Gowinda Hela (a giant rock mountain known also as Westminster Abbey).

Distribution: Indo-Malayan: Sri Lanka.

***Agraphydrus fujianensis* Komarek & Hebauer, 2018**

*Agraphydrus fujianensis* Komarek & Hebauer, 2018: 41 – China, Fujian Prov., Jianyuan Prefecture, Chong’an City Region, Wuyi Shan, 3 km SW Wuyi Gong Village (= Shanqian).

Distribution: Indo-Malayan: China (Fujian).

***Agraphydrus geminus* (d’Orchymont, 1932)**

Helochares (Gymnhelochares) geminus d’Orchymont, 1932: 694 – Indonesia, W. Java, “Tjibodas-Bach”.

Agraphydrus (Gymnhelochares) geminus (d’Orchymont, 1932); Hansen 1991: 292 [subgenus transferred from *Helochares* to *Agraphydrus*]; Hansen 1999b: 157 [catalog].

*Agraphydrus geminus* (d’Orchymont, 1932); Komarek 2019: 186 [taxonomic treatment].

Distribution: Indo-Malayan: Indonesia (Java, Sumatra).

***Agraphydrus gereckei* Komarek, 2020**

*Agraphydrus gereckei* Komarek, 2020: 150 – Madagascar, Antsiranana Province, Sava Region, Antalaha District, Maromandia (town), above Marofinatra (village), Ankavia River.

Distribution: Afrotropical: Madagascar.

***Agraphydrus gilvus* Komarek, 2018**

*Agraphydrus gilvus* Komarek, 2018: 129 – India, Kerala, Kallar Valley, 10 km WSW Munnar, 10°3'N, 76°59'E.

Distribution: Indo-Malayan: India (Kerala).

***Agraphydrus glaber* Komarek, 2018**

*Agraphydrus glaber* Komarek, 2018: 130 – India, Madhya Pradesh, Hoshangabad District, ca. 5 km NE Hoshangabad, ca. 60 km SSE Bhopal, Bandrabhan, Narmada River, 22°48'1"N, 77°46'45"E.

Distribution: Indo-Malayan: India (Madhya Pradesh).

***Agraphydrus globipenis* Komarek & Hebauer, 2018**

*Agraphydrus globipenis* Komarek & Hebauer, 2018: 41 – China, Hunan Prov., Huaihua Pref., Huitong County, Jinlong Shan, ca. 30 km NE Huitong City.

Distribution: Indo-Malayan: China (Guangxi, Hunan).

***Agraphydrus goldschmidti* Komarek, 2020**

*Agraphydrus goldschmidti* Komarek, 2020: 151 – Madagascar, Toliara Province, Anosy Region, Tsimelahy, Antarantsa River.

Distribution: Afrotropical: Madagascar.

***Agraphydrus gracilipalpis* Komarek & Hebauer, 2018**

*Agraphydrus gracilipalpis* Komarek & Hebauer, 2018: 42 – China, Guangdong Prov., Zhaoqing Prefecture, Dinghu Nature Reserve, 23°11'03"N, 112°33'06"E.

Distribution: Indo-Malayan: China (Fujian, Guangdong).

***Agraphydrus hamatus* Komarek, 2019**

*Agraphydrus hamatus* Komarek, 2019: 187 – Vietnam, Hòa Binh Province, Lac Tho.

Distribution: Indo-Malayan: Vietnam.

***Agraphydrus hanseni* (Satô & Yoshitomi, 2004)**

*Horelophopsis hanseni* Satô & Yoshitomi, 2004: 42 – Japan, Ôura-gawa Kakou, Okinawa-jima, Ryukyus. Yoshitomi and Nakajima 2005: 376 [new record]; Short and Hebauer 2006: 321 [catalog]; Minoshima et al. 2013: 711 [description of larvae; phylogenetic placement]; Short and Fikáček 2013: 731 [phylogenetic placement]; Fikáček et al. 2015: 62 [catalog].

*Agraphydrus hanseni* (Satô and Yoshitomi); Short et al. 2021: 11 [new combination].

Distribution: Palearctic: Japan.

***Agraphydrus heinrichi* Komarek, 2018**

*Agraphydrus heinrichi* Komarek, 2018: 131 – India, Kerala, Thiruvananthapuram District, Cardamom Hills, 50 km NW Pathanamthitta, near Pambaiyar River, ca. 9°25'N, 77°5'E

Distribution: Indo-Malayan: India (Kerala).

***Agraphydrus helicopter* Komarek, 2019**

*Agraphydrus helicopter* Komarek, 2019: 188 – Malaysia, Johor, Gunung Ledang N.P., Gunung Ledang (= Mt. Ophir), Hutan (= forest) Lipur.

Distribution: Indo-Malayan: Malaysia (Peninsula).

***Agraphydrus hendrichi* Komarek, 2019**

*Agraphydrus hendrichi* Komarek, 2019: 189 – Malaysia, Pahang, Taman Negara N.P., surroundings of Nusa Camp.

Distribution: Indo-Malayan: Malaysia (Peninsula).

***Agraphydrus heterochromatus* Komarek, 2019**

*Agraphydrus heterochromatus* Komarek, 2019: 190 – Malaysia, Penang, George Town City, Botanic Gardens (= Waterfall Gardens).

Distribution: Indo-Malayan: Malaysia (Peninsula), Thailand.

***Agraphydrus hortensis* Komarek, 2019**

*Agraphydrus hortensis* Komarek, 2019: 192 – Malaysia, Penang, George Town City, Botanic Garden.

Distribution: Indo-Malayan: Malaysia (Peninsula)

***Agraphydrus hygropetricus* Komarek, 2018**

*Agraphydrus hygropetricus* Komarek, 2018: 132 – Sri Lanka [Ceylon], Western Province, 24 miles ESE Colombo, Labugama (village).

Distribution: Indo-Malayan: Sri Lanka.

***Agraphydrus igneus* Komarek & Hebauer, 2018**

*Agraphydrus igneus* Komarek & Hebauer, 2018: 43 – China, Hong Kong, Lantau Island, Ngong Ping village, Po Lin Monastery environment, 22°15.25'N, 113°54.6"E; Komarek 2019: 193 [taxonomic treatment].

Distribution: Indo-Malayan: China (Guangdong, Hong Kong), Laos.

***Agraphydrus imitans* Komarek, 2019**

*Agraphydrus imitans* Komarek, 2019: 193 – Myanmar, Mandalay Region, ca. 50 km NW Kalaw, Myitsone River, 20°48'27.42"N, 96°21'36.6"E.

Distribution: Indo-Malayan: Laos, Myanmar, Thailand, Vietnam.

***Agraphydrus indicus* (d’Orchymont, 1932)**

Helochares (Gymnhelochares) indicus d’Orchymont, 1932: 694 – India, Uttar Pradesh, Kumaon, Haldwani distr.

Agraphydrus (Gymnhelochares) indicus (d’Orchymont, 1932); Hansen 1999b: 157 [new combination]; Hebauer 2002a: 20 [new records]; Hansen 2004: 49 [checklist]; Fikáček et al. 2015: 61 [catalog].

*Agraphydrus indicus* (d’Orchymont, 1932); Komarek 2018: 133 [new records; redescription].

Distribution: Indo-Malayan: Bhutan, India (Arunachal Pradesh, Himachal Pradesh, Meghalaya, Uttarakhand, Uttar Pradesh), Nepal.

***Agraphydrus inflatus* Komarek, 2018**

*Agraphydrus inflatus* Komarek, 2018: 136 – India, Kerala, Idukki District, Cardamom Hills, Kallar Valley, 15 km SW Munnar, ca. 10°02'N, 76°58'E.

Distribution: Indo-Malayan: India (Kerala, Tamil Nadu).

***Agraphydrus infuscatus* Komarek, 2019**

*Agraphydrus infuscatus* Komarek, 2019: 195 – Thailand, Phang Nga Province, Khuraburi District, Baan Tumnang, west of Si Phang Nga N.P.

Distribution: Indo-Malayan: Thailand.

***Agraphydrus insidiator* Minoshima, Komarek & Ôhara, 2015**

Agraphydrus (Agraphydrus) insidiator Minoshima, Komarek & Ôhara, 2015: 48 – Taiwan: Taichung City, Heping District, Basian-shan National Forest Recreation Area, 24°11.55'N, 121°00.83'E.

*Agraphydrus insidiator* Minoshima, Komarek, & Ôhara, 2015; Komarek and Hebauer 2018: 44 [redescription].

Distribution: Indo-Malayan: China (Taiwan).

***Agraphydrus ishiharai* (Matsui, 1994)**

*Enochrus ishiharai* Matsui, 1994: 215 – Japan, Kyushu, Kumamoto Pref., Ue Village, Menda River.

Agraphydrus (Agraphydrus) ishiharai (Matsui, 1994); Hansen 1999b: 156 [new combination]; Hansen 2004: 49 [checklist]; Fikáček et al. 2015: 60 [catalog]; Minoshima 2016: 353 [redescription]; Lee and Ahn 2017: 39 [new record].

Distribution: Palearctic: Japan, Korea.

***Agraphydrus jaechi* (Hansen, 1999)**

*Megagraphydrus jaechi* Hansen, 1999a: 140 – Malaysia, Penang Aceh Forest Reserve 2 km W Telok Bahang; Hansen 1999b: 157 [catalog].

Agraphydrus (Agraphydrus) jaechi (Hansen, 1999); Minoshima et al. 2015: 18 [new combination; redescription].

*Agraphydrus jaechi* (Hansen, 1999); Komarek 2019: 196 [taxonomic treatment].

*Megagraphydrus superans* Hebauer, 2000: 16 – Malaysia, Pahang, Taman Negara National Park, Nusa Camp; Short and Hebauer 2006: 337 [catalog]; Komarek 2019 [synonymy].

Agraphydrus (Agraphydrus) superans (Hebauer, 2000); Minoshima et al. 2015: 35 [new combination].

Distribution: Indo-Malayan: Malaysia (Peninsula).

***Agraphydrus jankodadai* Komarek, 2019**

*Agraphydrus jankodadai* Komarek, 2019: 197 – Malaysia, Sabah, Interior Division, Nabawan District, near Batu Punggul Resort.

Distribution: Indo-Malayan: Malaysia (Borneo).

***Agraphydrus jilanzhui* Komarek & Hebauer, 2018**

*Agraphydrus jilanzhui* Komarek & Hebauer, 2018: 45 – China, Shaanxi Prov., Qin Ling Shan, 33°55'N, 108°49'E.

Distribution: Palearctic: China (Gansu, Hubei, Shaanxi, Sichuan).

***Agraphydrus kallar* Komarek, 2018**

*Agraphydrus kallar* Komarek, 2018: 137 – India, Kerala, Thiruvananthapuram District, 30 km NNE Thiruvananthapuram, Kallar, ca. 8°45'N, 77°5'E.

Distribution: Indo-Malayan: India (Kerala).

***Agraphydrus kathapa* Komarek, 2019**

*Agraphydrus kathapa* Komarek, 2019: 198 – Myanmar, Sagaing Region, Alaungdaw Kathapa N.P., 22°19'5.64"N, 94°28'49.38"E.

Distribution: Indo-Malayan: Myanmar.

***Agraphydrus kempi* (d’Orchymont, 1922)**

Helochares (s. str.) kempi d’Orchymont, 1922: 626 – India, Arunachal Pradesh, Abors, “Yembung”.

Helochares (Agraphydrus) kempi (d’Orchymont, 1922); d’Orchymont 1927a: 5 [transferred from subgenus (s. str.) to subgenus (*Agraphydrus*)]; d’Orchymont 1928: 108 [faunistic treatment].

Agraphydrus (Agraphydrus) kempi (d’Orchymont, 1922); Hansen 1999b: 156 [new combination]; Hebauer 2002a: 21 [new record]; Hansen 2004: 60 [checklist]; Fikáček et al. 2015: 60 [catalog].

*Agraphydrus kempi* (d’Orchymont, 1922); Komarek 2018: 138 [new records; redescription].

Distribution: Indo-Malayan: Bhutan, India (Arunachal Pradesh, Meghalaya, Uttar Pradesh, Uttarakhand), Nepal.

***Agraphydrus khasiensis* Komarek, 2018**

*Agraphydrus khasiensis* Komarek, 2018: 141 – India, Meghalaya, Khasi Hills District, Shillong Peak, 25°32.8'N, 91°52.5'E.

Distribution: Indo-Malayan: India (Meghalaya).

***Agraphydrus kodaguensis* Komarek, 2018**

*Agraphydrus kodaguensis* Komarek, 2018: 142 – India, Karnataka, Kodagu District, Tadiyendamol Mountain, ca. 12°14'N, 75°36'E.

Distribution: Indo-Malayan: India (Karnataka).

***Agraphydrus laocaiensis* Komarek, 2019**

*Agraphydrus laocaiensis* Komarek, 2019: 200 – Vietnam, Lào Cai Province, Sa Pa District, near Sa Pa (District capital), Cát Cát (village), 22°19'N, 103°50'E.

Distribution: Indo-Malayan: Vietnam.

***Agraphydrus latus* Komarek, 2019**

*Agraphydrus latus* Komarek, 2019: 201 – Malaysia, Perak, Manjung District, Pangkor Island, Teluk Nipah (village).

Distribution: Indo-Malayan: Malaysia (Peninsula).

***Agraphydrus longipalpus* (Jia, 1998)**

*Pseudopelthydrus longipalpus* Jia, 1998: 229 – China, Hainan, Jianfengling, Tianchi; Hansen 1999b: 126 [catalog].

*Agraphydrus longipalpis* (Jia, 1998) [incorrect subsequent spelling]; Komarek 2003: 384 [new combination]; Short and Hebauer 2006: 330 [catalog].

Agraphydrus (Gymnhelochares) longipalpis (Jia, 1998) [incorrect subsequent spelling]; Hansen 2004: 49 [checklist].

Agraphydrus (Agraphydrus) longipalpus (Jia, 1998); Fikáček et al. 2015: 60 [catalog].

*Agraphydrus longipalpus* (Jia, 1998); Komarek and Hebauer 2018: 46 [redescription].

Distribution: Indo-Malayan: China (Hainan).

***Agraphydrus longipenis* Komarek & Hebauer, 2018**

*Agraphydrus longipenis* Komarek & Hebauer, 2018: 47 – Laos, Luang Nam Tha Prov., Luang Nam Tha [City] environment; Komarek 2019: 202 [taxonomic treatment].

Distribution: Indo-Malayan: China (Yunnan), Laos.

***Agraphydrus lunaris* Komarek, 2019**

*Agraphydrus lunaris* Komarek, 2019: 202 – Laos, Khammouan Province, Khoun Ngeun (village), 18°07'N, 104°29'E.

Distribution: Indo-Malayan: Laos.

***Agraphydrus luteilateralis* (Minoshima & Fujiwara, 2009)**

*Megagraphydrus luteilateralis* Minoshima & Fujiwara, 2009: 55 – Japan, Okinawa Prefecture, Iriomote-jima Island, Shirahama, 24°21'59"N, 123°45'22"E; Short and Fikáček 2011: 91 [checklist].

Agraphydrus (Agraphydrus) luteilateralis (Minoshima & Fujiwara, 2009); Minoshima et al. 2015: 22 [new combination]; Minoshima 2016: 355 [taxonomic treatment].

Agraphydrus (Agraphydrus) luteimarginalis (Minoshima & Fujiwara, 2009) [incorrect subsequent spelling]; Fikáček et al. 2015: 62 [catalog].

Distribution: Palearctic: Japan.

***Agraphydrus madagascarensis* Komarek, 2020**

*Agraphydrus madagascarensis* Komarek, 2020: 152 – Madagascar, Toamasina Province, Atsinanana Region, Toamasina (town), Parc Ivoloina.

Distribution: Afrotropical: Madagascar.

***Agraphydrus maehongsonensis* Komarek, 2019**

*Agraphydrus maehongsonensis* Komarek, 2019: 203 – Thailand, Mae Hong Son Province.

Distribution: Indo-Malayan: Thailand.

***Agraphydrus malayanus* (Hebauer, 2000)**

*Megagraphydrus malayanus* Hebauer, 2000: 15 – Malaysia, Kedah, SW Langkawi, Telaga Tujuh; Short and Hebauer 2006: 337 [catalog].

Agraphydrus (Agraphydrus) malayanus (Hebauer, 2000); Minoshima et al. 2015: 22 [new combination; record from Thailand in doubt].

*Agraphydrus malayanus* (Hebauer, 2000); Komarek 2019: 158 [taxonomic treatment; excluded from Thailand].

Distribution: Indo-Malayan: Malaysia.

***Agraphydrus malkini* Komarek, 2020**

*Agraphydrus malkini* Komarek, 2020: 154 – Cameroon, Southwest Region, Manyu Division, Mamfe.

Distribution: Afrotropical: Cameroon.

***Agraphydrus manfredjaechi* Komarek, 2019**

*Agraphydrus manfredjaechi* Komarek, 2019: 206 – Indonesia, North Sulawesi Province, Dua Saudara N.P., E of Manado (capital city).

Distribution: Indo-Malayan: Indonesia (Seram, Sulawesi).

***Agraphydrus masatakai* Minoshima, Komarek & Ôhara, 2015**

Agraphydrus (Agraphydrus) masatakai Minoshima, Komarek & Ôhara, 2015: 49 – Houaphanh Province, Xam Neua, Ban Saleui.

*Agraphydrus masatakai* Minoshima, Komarek & Ôhara, 2015; Komarek and Hebauer 2018: 48 [redescription]; Komarek 2019: 207 [new records].

Distribution: Indo-Malayan: China (Guangdong, Hainan, Hong Kong, Yunnan), Laos, Malaysia, Myanmar, Thailand, Vietnam.

***Agraphydrus matoposensis* Komarek, 2020**

*Agraphydrus matoposensis* Komarek, 2020: 155 – Zimbabwe, Matabeleland South Province, Matopos N.P., 20°33'S, 28°30'E.

Distribution: Afrotropical: Zimbabwe.

***Agraphydrus mazzoldii* Komarek, 2019**

*Agraphydrus mazzoldii* Komarek, 2019: 209 – Thailand, Mukdahan Province, Phu Pha Thoep N.P.

Distribution: Indo-Malayan: Thailand.

***Agraphydrus meghalayanus* Komarek, 2018**

*Agraphydrus meghalayanus* Komarek, 2018: 143 – India, Meghalaya, East Khasi Hills District, 11 km SW Cherrapunjee, Laitkynsew, 25°12'N, 91°40'E.

Distribution: Indo-Malayan: India (Meghalaya).

***Agraphydrus microphthalmus* Komarek, 2019**

*Agraphydrus microphthalmus* Komarek, 2019: 210 – Malaysia, Sarawak, Kapit Division, Kapit District, ca. 25 km E of Kapit.

Distribution: Indo-Malayan: Malaysia (Borneo).

***Agraphydrus minutissimus* (Kuwert, 1890)**

Helochares (s. str.) minutissimus Kuwert, 1890b: 304 – Syria; d’Orchymont 1923a: 9 [faunistic treatment]; Hebauer 1994: 112 [faunistic treatment; identification doubtful].

*Helochares minutissimus* Kuwert, 1890; d’Orchymont 1926a: 379 [as synonym of *H. pallens*].

Helochares (Agraphydrus) minutissimus Kuwert; d’Orchymont 1939c: 197 [not synonym of *Helochares pallens* (MacLeay, 1825) as in d’Orchymont 1926a: 379); Balfour-Browne 1951: 213 [new record].

*Agraphydrus minutissimus* (Kuwert, 1890); Hebauer 1995a: 265 [new combination; new record]; Hebauer 1997: 264 [new record]; Fikáček et al. 2010: 149 [faunistic treatment]; Przewoźny 2019 [checklist]; Komarek 2020: 156 [faunistic treatment; new records].

Agraphydrus (Agraphydrus) minutissimus (Kuwert, 1890); Hansen 1999b: 156 [catalog]; Hansen 2004: 49 [checklist]; Hebauer 2006a: 27 [checklist]; Fikáček et al. 2015: 60 [catalog]; Ribera et al. 2019: 264 [checklist].

Distribution: Palearctic: Syria. Afrotropical: Djibouti, Eritrea, Ethiopia (in doubt), Iran, Oman, Saudi Arabia, Sudan, Yemen. Excluded from Kenya, Madagascar, Namibia, and Republic of South Africa (Komarek 2020).

***Agraphydrus mirabilis* Komarek, 2019**

*Agraphydrus mirabilis* Komarek, 2019: 212 – Thailand, Chiang Mai Province, Doi (= mountain) Suthep N.P., Huai Sa Lad, 18°48'18.6"N, 98°54'31.2"E.

Distribution: Indo-Malayan: Thailand.

***Agraphydrus montanus* Minoshima, Komarek & Ôhara, 2015**

Agraphydrus (Agraphydrus) montanus Minoshima, Komarek & Ôhara, 2015: 54 – India, West Sikkim, Sikkim State, Yuksom.

*Agraphydrus montanus* Minoshima, Komarek, & Ôhara, 2015; Komarek 2018: 144 [redescription].

Distribution: Indo-Malayan: India (Sikkim).

***Agraphydrus muluensis* Komarek, 2019**

*Agraphydrus muluensis* Komarek, 2019: 213 – Malaysia, Sarawak, Miri Division, Gunung Mulu National Park.

Distribution: Indo-Malayan: Malaysia (Borneo).

***Agraphydrus musculus* Komarek, 2019**

*Agraphydrus musculus* Komarek, 2019: 214 – Malaysia, Sarawak, Kapit Division, Kapit District, ca. 25 km E of Kapit.

Distribution: Indo-Malayan: Malaysia (Borneo).

***Agraphydrus namthaensis* Komarek, 2019**

*Agraphydrus namthaensis* Komarek, 2019: 215 – Laos, Luang Nam Tha Province, Muang Sing District, ca. 20 km SE Muang Sing (town).

Distribution: Indo-Malayan: Laos.

***Agraphydrus nanus* Komarek, 2019**

*Agraphydrus nanus* Komarek, 2018: 145 – India, Kerala, Thiruvananthapuram District, Cardamom Hills, 50 km NW Pathanamthitta, Pambaiyar River, 9°25'N, 77°05'E.

Distribution: Indo-Malayan: India (Karnataka, Kerala, Madhya Pradesh).

***Agraphydrus narusei* (Satô, 1960)**

*Pseudohelochares narusei* Satô, 1960: 77 – Japan, Shikoku, Kôchi Pref., Kurosongawa River.

*Agraphydrus narusei* (Satô, 1960); Satô, 1965: 128 [new combination]; Hansen 1999b: 156 [checklist]; Hansen 2004: 49 [checklist]; Lee and Ahn 2009: 317 [redescription; new record]; Minoshima and Hayashi 2011: 17 [description of larvae]; Fikáček et al. 2015: 60 [catalog]; Minoshima 2016: 356 [redescription].

Distribution: Palearctic: Japan, South Korea.

***Agraphydrus nemorosus* Komarek, 2019**

*Agraphydrus nemorosus* Komarek, 2019: 216 – Laos, Houaphan Province, 25 km SE (by road) of Vieng Xai City, Kangpabong (village), 20°19'N, 104°25'E.

Distribution: Indo-Malayan: Laos.

***Agraphydrus nepalensis* Komarek, 2018**

*Agraphydrus nepalensis* Komarek, 2018: 146 – Nepal, Eastern Region, Koshi Zone, 2 km E Mangsingma.

Distribution: Indo-Malayan: Nepal.

***Agraphydrus niger* Komarek & Hebauer, 2018**

*Agraphydrus niger* Komarek & Hebauer, 2018: 50 – China, Fujian Prov., Jianyuan Prefecture, Chong’an City Region, ca. 1 km W Wuyi Gong Village (= Shanqian, ca. 10 km S Chong’an City).

Distribution: Indo-Malayan: China (Fujian, Zheijang).

***Agraphydrus nigroflavus* Komarek, 2019**

*Agraphydrus nigroflavus* Komarek, 2019: 217 – Indonesia, North Kalimantan Province [formerly part of East Kalimantan Province], Malinau Regency, Kayan Selatan District, Apokayan Highlands, Sungai Barang (village), Lalut Wai.

Distribution: Indo-Malayan: Indonesia (Borneo).

***Agraphydrus obesus* Komarek, 2019**

*Agraphydrus obesus* Komarek, 2019: 218 – Vietnam, Central Highlands, Lâm Đồng Province, 12 km N Đà Lạt, Lang Bian.

Distribution: Indo-Malayan: Vietnam.

***Agraphydrus obscuratus* Komarek, 2018**

*Agraphydrus obscuratus* Komarek, 2018: 148 – India, Kerala, Thiruvananthapuram District, Cardamom Hills, 50 km NW Pathanamthitta, near Pambaiyar River, ca. 9°25'N, 77°5'E.

Distribution: Indo-Malayan: India (Karnataka, Kerala, Maharashtra).

***Agraphydrus obsoletus* Komarek, 2018**

*Agraphydrus obsoletus* Komarek, 2018: 149 – India, Kerala, Idukki District, 10 km WSW Munnar, Kallar Valley, ca. 10°3'N, 76°58'E.

Distribution: Indo-Malayan: India (Karnataka, Kerala, Tamil Nadu).

***Agraphydrus occultus* Komarek & Freitag, 2020**

*Agraphydrus occultus* Komarek & Freitag, 2020: 214 – Philippines, Luzon Island, Laguna Province, Majayjay Municipality, Barangay Burgos, Taytay River downstream of Imelda Falls, secondary forest, 510 m a.s.l., 14°6'42"N, 121°30'19"E.

Distribution: Indo-Malayan: Philippines (Luzon, Mindoro, Palawan?, Panay?).

***Agraphydrus ogatai* Minoshima, 2016**

*Agraphydrus* sp. Inoue et al. 2009: 76 [photo, as an undescribed species similar to *A. narusei*; in Japanese].

Agraphydrus (Agraphydrus) ogatai Minoshima, 2016: 359 – Japan, Fukuoka Pref., Koga-shi, Taniyama, Taniyamagawa River [about 33°42'N, 130°30'E].

Distribution: Palearctic: Japan.

***Agraphydrus orbicularis* Komarek, 2019**

*Agraphydrus orbicularis* Komarek, 2019: 219 – Malaysia, Sarawak, Kuching Division, Semengoh, 30 km S Kuching, Semengoh Nature Reserve.

Distribution: Indo-Malayan: Malaysia (Borneo).

***Agraphydrus orientalis* (d’Orchymont, 1932)**

Helochares (Agraphydrus) orientalis d’Orchymont, 1932: 690 – Indonesia, E. Java, “Ranu Bedali”.

*Agraphydrus orientalis* (d’Orchymont, 1932); Satô 1965: 128 [*Agraphydrus* re-established as genus]; Gentili et al. 1995: 208 [checklist]; Komarek and Hebauer 2018: 65 [taxonomic treatment]; Komarek 2019: 220 [taxonomic treatment].

Agraphydrus (Agraphydrus) orientalis (d’Orchymont, 1932); Hansen 1999b: 156 [catalog]; Hansen 2004: 49 [checklist]; Fikáček et al. 2015: 60 [catalog].

Distribution: Indo-Malayan: China (Yunnan, Taiwan; in doubt, Komarek and Hebauer 2018: 65–66), Indonesia (Bali, Java, Lombok, Siberut, Sumatra).

***Agraphydrus palawanensis* Komarek & Freitag, 2020**

*Agraphydrus* sp. F; Freitag and Zettel 2013: 19, 30.

*Agraphydrus palawanensis* Komarek & Freitag, 2020: 216 – Palawan Island and Province, Puerto Princesa City, Barangay Cabayugan, presumably Cabayugan River tributary, primary forest, ca. 100 m a.s.l., ca. 10°9'N, 118°52'E.

Distribution: Indo-Malayan: Philippines (Palawan, Busuanga).

***Agraphydrus pallidus* Komarek, 2019**

*Agraphydrus pallidus* Komarek, 2019: 222 – Vietnam, Vĩnh Phúc Province, Tam Đảo.

Distribution: Indo-Malayan: Vietnam.

***Agraphydrus papuanus* Komarek, 2019**

*Agraphydrus papuanus* Komarek, 2019: 223 – Indonesia, West Papua, Pegunungan Bintang Regency, Central Range, Kali Takime, 4°24'S, 140°25'E.

Distribution: Australasian: Indonesia (New Guinea), Papua New Guinea.

***Agraphydrus pauculus* (Knisch, 1924)**

Helochares (Helocharimorphus) pauculus Knisch, 1924b: 36 – India, Uttar Pradesh, Kumaun, W. Almora.

*Helochares panculus* Knisch, 1924 [incorrect subsequent spelling]; d’Orchymont 1927a: 5 [taxonomic treatment].

Helochares (Agraphydrus) pauculus Knisch, 1924; d’Orchymont 1928: 108 [faunistic treatment].

*Agraphydrus pauculus* (Knisch, 1924); Hansen 1991: 148 [examined species]; Komarek 2018: 151 [new record; redescription].

*Agraphilydrus pauculus* Knisch, 1924; Chiesa 1967: 275 [incorrect identification, Komarek 2018: 153]

Agraphydrus (Agraphydrus) pauculus (Knisch, 1924); Hansen 1999b: 156 [catalog]; Hebauer 2002a: 22 [new records]; Hansen 2004: 49 [checklist]; Fikáček et al. 2015: 60 [checklist].

Distribution: Palearctic: China (Tibet, Komarek 2018: 153). Indo-Malayan: India (Uttarakhand), Nepal.

***Agraphydrus pauper* Komarek, 2020**

*Agraphydrus pauper* Komarek, 2020: 159 – Madagascar, Antsiranana Province, Sava Region, Andapa District, riparian springs at Masiaposa River, crossing Route National 3b at km 5–6.

Distribution: Afrotropical: Madagascar.

***Agraphydrus pelingeni* Komarek & Freitag, 2020**

Agraphydrus (Agraphydrus) cf. orientalis (d’Orchymont, 1932); Freitag and Zettel 2013: 19, 30.

*Agraphydrus pelingeni* Komarek & Freitag, 2020: 216 – Philippines, Palawan Island and Province, Puerto Princesa City, Barangay Concepcion, Tarabanan River upstream of Batak village, secondary forest, ca. 30 m a.s.l., ca. 10°1'N, 119°1'E.

Distribution: Indo-Malayan: Philippines (Palawan).

***Agraphydrus penangensis* Komarek, 2019**

*Agraphydrus penangensis* Komarek, 2019: 225 – Malaysia, Penang, Southwest Penang Island, Pantai Aceh Forest Reserve (= Penang National Park).

Distribution: Indo-Malayan: Malaysia (Peninsula).

***Agraphydrus piceus* Komarek, 2019**

*Agraphydrus piceus* Komarek, 2019: 226 – Malaysia, Sabah, West Coast Division, Ranau District, Ranau (town), Liwagu River.

Distribution: Indo-Malayan: Malaysia (Borneo).

***Agraphydrus politus* (Hansen, 1999)**

*Megagraphydrus politus* Hansen, 1999a: 138 – Taiwan, Taipei Wulai; Hansen 1999b: 158 [checklist]; Hebauer 2000: 18 [checklist]; Hansen 2004: 52 [checklist]; Fikáček et al. 2015: 62 [catalog].

Agraphydrus (Agraphydrus) politus (Hansen, 1999); Minoshima et al. 2015: 24 [new combination; redescription].

*Agraphydrus politus* (Hansen, 1999); Komarek and Hebauer 2018: 51 [redescription].

*Megagraphydrus wangi* Hebauer, 2000: 17 – Taiwan, Taipei Hsien, Sanhsia, 24°51'21"N, 121°24'33"E; Hansen 2004: 52 [checklist]; Short and Hebauer 2006: 337 [catalog]; Fikáček et al. 2015: 63 [catalog]; Minoshima et al. 2015: 25 [synonym with *A. politus*].

Distribution: Indo-Malayan: China (Taiwan).

***Agraphydrus praecipuus* (d’Orchymont, 1937)**

Helochares (Agraphydrus) praecipuus d’Orchymont, 1937b: 252 – Madagascar, Toliara Province, Androy Region [(Sud), Pays Androy (Nord)].

Agraphydrus (Agraphydrus) praecipuus (d’Orchymont, 1937); Hansen 1999b: 157 [new combination; catalog]; Hebauer 2006a: 27 [checklist].

*Agraphydrus praecipuus* (d’Orchymont, 1937); Komarek 2020: 160 [faunistic treatment].

Distribution: Afrotropical: Madagascar.

***Agraphydrus protentus* Komarek, 2018**

*Agraphydrus protentus* Komarek, 2018: 153 – India, Uttarakhand, Nainital.

Distribution: Indo-Malayan: India (Uttarakhand), Nepal.

***Agraphydrus pullus* Komarek, 2018**

*Agraphydrus pullus* Komarek, 2018: 154 – Nepal, Eastern Region, Koshi Zone, Sunsari District, Dharan (city) environment.

Distribution: Indo-Malayan: Nepal.

***Agraphydrus punctatellus* Régimbart, 1903**

*Agraphydrus punctatellus* Régimbart, 1903a: 34 – Madagascar [“Diégo-Suarez; forêt de la côte Est de Madagascar”); Komarek 2020: 161 [faunistic treatment; new record].

Enochrus (Agraphydrus) punctatellus Régimbart, 1903; Knisch 1924a: 219 [catalog].

Agraphydrus (Agraphydrus) punctatellus Régimbart, 1903; Satô, 1965: 128 [subgenus transferred from *Enochrus* to *Agraphydrus*]; Hansen 1999b: 157 [catalog]; Hebauer 2006a: 27 [checklist; new records].

Distribution: Afrotropical: Eswatini, Madagascar, Mozambique, Republic of South Africa, Tanzania.

***Agraphydrus punctulatus* Komarek, 2018**

*Agraphydrus punctulatus* Komarek, 2018: 155 – India, Madhya Pradesh, Hoshangabad District, Pachmarhi Wildlife Sanctuary, Satpura Mountain Range, Apsara Vihar (stream), ca. 3 km SSE Pachmarhi, 22°27'7"N, 78°26'39"E.

Distribution: Indo-Malayan: India (Madhya Pradesh).

***Agraphydrus puzhelongi* (Jia, 2010)**

*Megagraphydrus puzhelongi* Jia, 2010: 65 – China, Jiangxi Province, Shangrao, Sanqingshan mount, Upper Xinjiang river; Short and Fikáček 2011: 91 [catalog]; Fikáček et al. 2015: 63 [catalog].

Agraphydrus (Agraphydrus) puzhelongi (Jia, 2010); Minoshima et al. 2015: 30 [new combination].

*Agraphydrus puzhelongi* (Jia, 2010); Komarek and Hebauer 2018: 52 [redescription].

Distribution: Indo-Malayan: China (Guizhou, Jiangxi).

***Agraphydrus pygmaeus* (Knisch, 1924)**

Helochares (Helocharimorphus) pygmaeus Knisch, 1924b: 38 – India, Kumaon, W Almora; d’Orchymont 1927a: 5 [taxonomic treatment].

Helochares (Agraphydrus) pygmaeus Knisch, 1924; d’Orchymont 1928: 108 [checklist].

Agraphydrus (Agraphydrus) pygmaeus Knisch, 1924; Hansen 1999b: 157 [new combination]; Hebauer 2002a: 22 [new record]; Hansen 2004: 49 [checklist]; Fikáček et al. 2015: 60 [catalog].

*Agraphydrus pygmaeus* (Knisch, 1924); Komarek 2018: 156 [new record].

Distribution: Indo-Malayan: Bhutan, India (Meghalaya, Uttarakhand), Nepal. Palearctic: China (Tibet, Komarek 2018: 158).

***Agraphydrus raucus* Komarek, 2019**

*Agraphydrus raucus* Komarek, 2019: 227 – Indonesia, West Sumatra Province, Lima Puluh Kota Regency, Lembah Harau Nature Reserve, 15 km NE of Payakumbu City.

Distribution: Indo-Malayan: Indonesia (Sumatra).

***Agraphydrus reductus* Komarek & Hebauer, 2018**

*Agraphydrus reductus* Komarek & Hebauer, 2018: 53 – China, Yünnan Prov., Xishuangbanna Dai Autonomous Prefecture, Mengla County, Menglun Town, ca. 10 km NW Menglun, Wushiwu He River.

Distribution: Indo-Malayan: China (Yunnan).

***Agraphydrus regularis* (Hansen, 1999)**

*Megagraphydrus regularis* Hansen, 1999a: 140 – Thailand, Phetchabun, 36 km SE Sila, Ban Pala Yai; Hansen 1999b: 158 [catalog].

Agraphydrus (Agraphydrus) regularis (Hansen, 1999); Minoshima et al. 2015: 30 [new combination; redescription].

*Agraphydrus regularis* (Hansen, 1999); Komarek 2019: 228 [taxonomic treatment].

Distribution: Indo-Malayan: Thailand.

***Agraphydrus reticulatus* Komarek, 2019**

*Agraphydrus reticulatus* Komarek, 2019: 230 – Thailand, Surat Thani Province, Khao Sok N.P.

Distribution: Indo-Malayan: Thailand.

***Agraphydrus reticuliceps* Komarek & Hebauer, 2018**

*Agraphydrus reticuliceps* Komarek & Hebauer, 2018: 53 – China, Hunan Prov., Zhangjiajie Pref., Wulingyuan, N Dayong City, Suoxiyu Nature Reserve.

Distribution: Indo-Malayan: China (Guizhou, Hunan). Palearctic: China (Hubei).

***Agraphydrus rhodesiensis* Komarek, 2020**

*Agraphydrus rhodesiensis* Komarek, 2020: 163 – Zimbabwe, Mashonaland East Province, Doboshava, 27 km N Harare.

Distribution: Afrotropical: Zimbabwe.

***Agraphydrus rhomboideus* Komarek, 2019**

*Agraphydrus rhomboideus* Komarek, 2019: 231 – Malaysia, Sarawak, Miri Division, Kelabit Highlands, 5 km E Bario (village community), Pa’Ukat (village).

Distribution: Indo-Malayan: Brunei, Indonesia (Borneo), Malaysia (Borneo).

***Agraphydrus rivalis* Komarek, 2020**

*Agraphydrus rivalis* Komarek, 2020: 164 – Madagascar, Fianarantsoa Province, Haute Matsiatra Region, Madiorano near Ranomena (villages), stream crossing the railroad at km 51.2.

Distribution: Afrotropical: Madagascar.

***Agraphydrus robustus* Komarek & Hebauer, 2018**

*Agraphydrus robustus* Komarek & Hebauer, 2018: 55 – China, Yünnan Prov., Simao Pref., 54 km SW Simao, Jian Shan River.

Distribution: Indo-Malayan: China (Guangdong, Yunnan).

***Agraphydrus rostratus* Komarek, 2018**

*Agraphydrus rostratus* Komarek, 2018: 158 – India, Tamil Nadu, Nilgiris District, Nilgiri Hills, Kotagiri (town) environment, Honnatti, ca. 11°25'N, 76°55'E.

Distribution: Indo-Malayan: India (Kerala, Tamil Nadu).

***Agraphydrus rugosus* Komarek, 2018**

*Agraphydrus rugosus* Komarek, 2018: 160 – India, Tamil Nadu, Nilgiris District, Nilgiri Hills, 15 km SE Kotagiri (town), Kunjapanai (village), ca. 11°22'N, 76°56'E.

Distribution: Indo-Malayan: India (Kerala, Tamil Nadu).

***Agraphydrus sarawakensis* Komarek, 2019**

*Agraphydrus sarawakensis* Komarek, 2019: 232 – Malaysia, Sarawak, Kapit Division, Kapit District, 25 km E of Kapit.

Distribution: Indo-Malayan: Malaysia (Borneo).

***Agraphydrus schoedli* Komarek, 2019**

*Agraphydrus schoedli* Komarek, 2019: 233 – Indonesia, North Sumatra Province, Toba Samosir Regency, Lumban Julu.

Distribution: Indo-Malayan: Indonesia (Sumatra).

***Agraphydrus schoenmanni* Komarek & Hebauer, 2018**

*Agraphydrus schoenmanni* Komarek & Hebauer, 2018: 56 – China, Yünnan Prov., Xishuangbanna Dai Autonomous Prefecture, Mengla County, Menglun Town, near Mangmo Village, road Menglun–Ganlanba, ca. 15 km W Menglun.

Distribution: Indo-Malayan: China (Yunnan).

***Agraphydrus scintillans* Komarek, 2019**

*Agraphydrus scintillans* Komarek, 2019: 235 – Vietnam, Vĩnh Phúc Province, Tam Đảo.

Distribution: Indo-Malayan: Vietnam.

***Agraphydrus scutifer* Komarek, 2020**

*Agraphydrus scutifer* Komarek, 2020: 165 – Madagascar, Fianarantsoa Province, Haute Matsiatra Region, Andringitra N.P., Amboahisy River, 22°7'54"S, 46°53'30"E.

Distribution: Afrotropical: Madagascar.

***Agraphydrus setifer* Komarek & Hebauer, 2018**

*Agraphydrus setifer* Komarek & Hebauer, 2018: 57 – Vietnam, Lào Cai Prov., Cat Cat, near Sa Pa, 22°19'43"N, 103°50'E; Komarek 2019: 236 [taxonomic treatment].

Distribution: Indo-Malayan: China (Yunnan), Vietnam.

***Agraphydrus shaverdoae* Komarek, 2019**

*Agraphydrus shaverdoae* Komarek, 2019: 236 – Myanmar, Shan State, Taunggyi District, NW Kalaw (town), km 23 on road between Kalaw and Thazi, 20°42'22.68"N, 96°30'13.08"E.

Distribution: Indo-Malayan: Myanmar, Thailand.

***Agraphydrus siamensis* (Hansen, 1999)**

*Megagraphydrus siamensis* Hansen, 1999a: 140 – Thailand, “Prae Siam”; Hansen 1999b: 158 [checklist]; Hebauer 2000: 18 [checklist].

Agraphydrus (Agraphydrus) siamensis (Hansen, 1999); Minoshima et al. 2015: 33 [new combination; redescription].

*Agraphydrus siamensis* (Hansen, 1999); Komarek 2019: 238 [taxonomic treatment].

Distribution: Indo-Malayan: Thailand.

***Agraphydrus sipekorum* Komarek, 2018**

*Agraphydrus sipekorum* Komarek, 2018: 161 – India, Meghalaya, East Khasi Hills District, 11 km SW Cherrapunjee, Laitkynsew, 25°12'48"N, 91°39'48"E.

Distribution: Indo-Malayan: India (Meghalaya).

***Agraphydrus skalei* Komarek, 2019**

*Agraphydrus skalei* Komarek, 2019: 239 – Indonesia, West Papua Province, Raja Ampat Regency, Waigeo Island, Lopintol, Rowery River, ca. 0°7'S, 130°53'E.

Distribution: Australasian: Indonesia (Waigeo Island).

***Agraphydrus spadix* Komarek, 2019**

*Agraphydrus spadix* Komarek, 2019: 240 – Thailand, Kanchanaburi Province, Sangkhla Buri District, Thung Yai Naresuan Wildlife Sanctuary.

Distribution: Indo-Malayan: Thailand.

***Agraphydrus spinosus* Komarek, 2019**

*Agraphydrus spinosus* Komarek, 2019: 241 – Malaysia, Selangor, Gombak District, Rawang Subdistrict, Templer Park.

Distribution: Indo-Malayan: Malaysia (Peninsula).

***Agraphydrus splendens* Komarek & Hebauer, 2018**

*Agraphydrus splendens* Komarek & Hebauer, 2018: 58 – Laos, Saisombun Special Zone, Mount Phu Bia.

Distribution: Indo-Malayan: China (Yunnan), Laos.

***Agraphydrus stagnalis* (d’Orchymont, 1937)**

Helochares (Agraphydrus) stagnalis d’Orchymont, 1937c: 37 – Pakistan, Punjab, Salt Range, Khewra Gorge.

Agraphydrus (Agraphydrus) stagnalis d’Orchymont, 1937; Hansen 1999b: 157 [new combination]; Hebauer 2002a: 22 [new record]; Hansen 2004: 49 [checklist]; Fikáček et al. 2015: 60 [catalog].

*Agraphydrus stagnalis* (d’Orchymont, 1937); Komarek 2018: 162 [new records].

Distribution: Indo-Malayan: Bhutan, India (Himachal, Uttar, Uttarakhand), Nepal. Palearctic: Pakistan.

***Agraphydrus stramineus* Komarek, 2019**

*Agraphydrus stramineus* Komarek, 2019: 242 – Malaysia, Sarawak, Miri Division, 30 km S Miri, Lambir Hills National Park.

Distribution: Indo-Malayan: Malaysia (Borneo).

***Agraphydrus sucineus* Komarek, 2019**

*Agraphydrus sucineus* Komarek, 2019: 244 – Malaysia, Pahang, Taman Negara N.P., surroundings of Nusa Camp.

Distribution: Indo-Malayan: Malaysia (Peninsula).

***Agraphydrus sundaicus* Komarek, 2019**

*Agraphydrus sundaicus* Komarek, 2019: 245 – Indonesia, West Sumatra Province, Padang City, 25 km E Padang, Taman Raya Bung Hatta Nature Reserve.

Distribution: Indo-Malayan: Indonesia (Java, Sumatra).

***Agraphydrus tamdao* Komarek, 2019**

*Agraphydrus tamdao* Komarek, 2019: 246 – Vietnam, Vĩnh Phúc Province, Tam Đảo.

Distribution: Indo-Malayan: Vietnam.

***Agraphydrus taprobanensis* Komarek, 2018**

*Agraphydrus taprobanensis* Komarek, 2018: 164 – Sri Lanka, Sabaragamuwa Province, Ratnapura District, Ratnapura (city).

Distribution: Indo-Malayan: Sri Lanka.

***Agraphydrus tenuipalpis* Komarek & Freitag, 2020**

*Agraphydrus tenuipalpis* Komarek & Freitag, 2020: 216 – Philippines, Leyte Island and Province, Baybay Municipality, secondary forest near Visayas State University, ca. 10°45'N, 124°48'E, ca. 100–200 m a.s.l.

Distribution: Indo-Malayan: Philippines (Leyte, Mindanao).

***Agraphydrus thaiensis* Minoshima, Komarek & Ôhara, 2015**

Agraphydrus (Agraphydrus) thaiensis Minoshima, Komarek & Ôhara, 2015: 56 – Thailand, Songkhla Province, Ton Nga Chang Wildlife Sanctuary.

*Agraphydrus thaiensis* Minoshima, Komarek, and Ôhara; Komarek 2019: 247 [taxonomic treatment].

Distribution: Indo-Malayan: Thailand.

***Agraphydrus tristis* Komarek, 2019**

*Agraphydrus tristis* Komarek, 2019: 248 – Myanmar, Mandalay Region, Pyin Oo Lwin District, Mogok Township, S Panlin village, west slope of Mt. Taung Mae, ca. 22°58'9"N, 96°27'11"E.

Distribution: Indo-Malayan: Myanmar.

***Agraphydrus tulipa* Komarek, 2019**

*Agraphydrus tulipa* Komarek, 2019: 250 – Thailand, Chiang Mai Province, Chiang Dao District, Doi (Luang) Chiang Dao (mountain).

Distribution: Indo-Malayan: Thailand.

***Agraphydrus tumidus* Komarek, 2020**

*Agraphydrus tumidus* Komarek, 2020: 166 – Madagascar, Toliara Province, Anosy Region, Tsimelahy, Antarantsa River, ca. 1 km upstream from village.

Distribution: Afrotropical: Madagascar.

***Agraphydrus tumulosus* Komarek, 2018**

*Agraphydrus tumulosus* Komarek, 2018: 165 – India, Kerala, Pathanamthitta District, Cardamom Hills, 50 km NW Pathanamthitta, Pambaiyar River, 9°25'N, 77°5'E.

Distribution: Indo-Malayan: India (Kerala).

***Agraphydrus umbrosus* Komarek & Hebauer, 2018**

*Agraphydrus umbrosus* Komarek & Hebauer, 2018: 59 – China, Fujian Prov., Jianyuan Prefecture, Yong’an City Region, ca. 20 km SE Yong’an City, 5 km SW Xiyang Village, Ziyungdong Shan.

Distribution: Indo-Malayan: China (Fujian, Guangdong).

***Agraphydrus uncinatus* Komarek & Hebauer, 2018**

*Agraphydrus uncinatus* Komarek & Hebauer, 2018: 60 – China, Yünnan Prov., Xishuangbanna Dai Autonomous Prefecture, Mengla County, along Mengla–Mengyüan road, ca. 6 km NW Mengla.

Distribution: Indo-Malayan: China (Yunnan).

***Agraphydrus usambaraensis* Komarek, 2020**

*Agraphydrus usambaraensis* Komarek, 2020: 168 – Tanzania, Tanga Region, East Usambara Mountains, Amani, Sigi River.

Distribution: Afrotropical: Tanzania.

***Agraphydrus uvaensis* (Hebauer, 2000)**

*Megagraphydrus uvaensis* Hebauer, 2000: 17 – Sri Lanka [Ceylon], Prov. of Uva, Gampaha Estate, 9 miles W Badulla; Short and Hebauer 2006: 337 [catalog].

Agraphydrus (Agraphydrus) uvaensis (Hebauer, 2000); Minoshima et al. 2015: 36 [new combination; redescription].

*Agraphydrus uvaensis* (Hebauer, 2000); Komarek 2018: 166 [redescription].

Distribution: Indo-Malayan: Sri Lanka.

***Agraphydrus vadoni* Komarek, 2020**

*Agraphydrus vadoni* Komarek, 2020: 193 – Analanjirofo Region, Toamasina Province, Maroantsetra.

Distribution: Afrotropical: Madagascar.

***Agraphydrus variabilis* Komarek & Hebauer, 2018**

*Agraphydrus variabilis* Komarek & Hebauer, 2018: 61 – China, Hong Kong, Lantau Island, Pak Kung Au, NW Cheung Sha; Angus et al. 2020: 19 [karyotype].

Distribution: Indo-Malayan: China (Fujian, Guangdong, Guangxi, Guizhou, Hong Kong, Hunan, Jiangxi, Yunnan, Zhejiang). Palearctic: China (Anhui, Gansu, Hubei, Shaanxi, Shandong, Sichuan, Taiwan).

***Agraphydrus vietnamensis* Komarek, 2019**

*Agraphydrus vietnamensis* Komarek, 2019: 251 – Vietnam, Lâm Đồng Province, 14 km SW Bao Loc.

Distribution: Indo-Malayan: Vietnam.

***Agraphydrus villiersi* (Balfour-Browne, 1958)**

Helochares (Gymnhelochares) villiersi Balfour-Browne, 1958a: 184 – Ivory Coast, Tonkoui.

Agraphydrus (Gymnhelochares) villiersi (Balfour-Browne, 1958); Hansen 1999b: 157 [new combination]; Hebauer 2006a: 27 [checklist; new records].

*Agraphydrus villiersi* (Balfour-Browne, 1958); Komarek 2020: 194 [redescription, new and corrected records].

Distribution: Afrotropical: Guinea [French Guinea], Ivory Coast, Nigeria (prior records in Cameroon and Gabon are erroneous).

***Agraphydrus wangmiaoi* Komarek & Hebauer, 2018**

*Agraphydrus wangmiaoi* Komarek & Hebauer, 2018: 63 – China, Hainan Prov., Ledong County, Jianfeng Mountains, ca. 5 km E Tian Chi Village.

Distribution: Indo-Malayan: China (Hainan).

***Agraphydrus yunnanensis* Komarek & Hebauer, 2018**

*Agraphydrus yunnanensis* Komarek & Hebauer, 2018: 64 – China, Yünnan Prov., Xishuangbanna Dai Autonomous Prefecture, Mengla County, ca. 50 km SSE Menglun, Mengyüan.

Distribution: Indo-Malayan: China (Yunnan).

***Agraphydrus zetteli* Komarek & Freitag, 2020: 221**

Agraphydrus (Agraphydrus) cf. orientalis (d’Orchymont, 1932); Freitag and Zettel 2013: 19, 30.

*Agraphydrus zetteli* Komarek & Freitag, 2020: 221 – Philippines, Mindoro Island, Province Oriental Mindoro, Victoria Municipality, Barangay Malayas, Malayas Creek (Lake Naujan affluent) flowing through secondary vegetation, ca. 20 m a.s.l., ca. 13°9'26"N, 121°18'29"E.

Distribution: Indo-Malayan: Philippines (Busuanga, Leyte, Luzon, Mindoro, Negros, Panay, Samar, Sibuyan).

### *Aulonochares* Girón & Short, 2019

***Aulonochares lingulatus* Girón & Short, 2019**

*Aulonochares lingulatus* Girón & Short, 2019: 119 – Suriname, Sipaliwini District; 2.97731N, 55.38500W; Camp 4 (low), Kasikasima; sandy stream on trail to METS camp.

Distribution: Neotropical: French Guiana, Suriname.

***Aulonochares novoairensis* Girón & Short, 2019**

*Aulonochares novoairensis* Girón & Short, 2019: 119 – Brazil, Amazonas: Novo Airão; 2°41'2.2878"S, 60°56'18.24"W.

Distribution: Neotropical: Brazil (Amazonas).

***Aulonochares tubulus* Girón & Short, 2019**

*Aulonochares tubulus* Girón & Short, 2019: 120 – Suriname, Sipaliwini District; 2°00.342'N, 55°58.149'W; 337 m; Sipaliwini Savanna nature Res., 4-Brothers Mts.

Distribution: Neotropical: Brazil (Roraima), Guyana, Suriname, Venezuela.

### *Batochares* Hansen, 1991

***Batochares burgeoni* (d’Orchymont, 1939)**

Helochares (Batochares) burgeoni d’Orchymont, 1939b: 293 – Democratic Republic of the Congo [Congo Belge], Haut Uélé, Moto; Balfour-Browne 1950b: 54 [faunistic treatment]; Hebauer 1996: 10 [taxonomic treatment]; Hansen 1999b: 172 [catalog]; Hebauer 2006a: 27 [checklist, new records].

*Batochares burgeoni* (d’Orchymont, 1939); Short et al. 2021: 11 [new combination].

Distribution: Afrotropical: Burundi/Rwanda, Democratic Republic of the Congo [Congo Belge; Zaire], Guinea, Kenya, Republic of the Congo [Congo/Brazzaville], Uganda.

***Batochares byrrhus* (d’Orchymont, 1939)**

Helochares (Batochares) byrrhus d’Orchymont, 1939b: 294 – Democratic Republic of the Congo [Congo Belge], Mayumbe, Sanzulu; Hebauer 1996: 10 [taxonomic treatment]; Hansen 1999b: 172 [catalog]; Hebauer 2006a: 27 [checklist, new records].

*Batochares byrrhus* (d’Orchymont, 1939); Short et al. 2021: 11 [new combination].

Distribution: Afrotropical: Central African Republic, Democratic Republic of the Congo [Congo Belge; Zaire], Gabon, Republic of the Congo [Congo/Brazzaville].

***Batochares corrugatus* (Balfour-Browne, 1958)**

Helochares (Batochares) corrugatus Balfour-Browne, 1958a: 183 – Guinea, Mount Nimba, “Camp de Ya”; Hebauer 1996: 10 [taxonomic treatment]; Hansen 1999b: 172 [catalog]; Hebauer 2006a: 27 [checklist].

*Batochares corrugatus* (Balfour-Browne, 1958); Short et al. 2021: 11 [new combination].

Distribution: Afrotropical: Guinea.

### *Chasmogenus* Sharp, 1882

***Chasmogenus acuminatus* Smith & Short, 2020**

*Chasmogenus acuminatus* Smith & Short, 2020: 32 – Suriname: Sipaliwini District 2°21.776'N, 56°41.861'W, 237 m, Camp 3 Wehepai.

*Chasmogenus* sp. X Short 2013: 87 (in part); Short and Kadosoe 2011: 87 (in part); Short et al. 2018: 193 (in part).

Distribution: Neotropical: Brazil (Amapá, Pará), French Guiana, Guyana, Suriname.

***Chasmogenus amplius* Smith & Short, 2020**

*Chasmogenus amplius* Smith & Short, 2020: 35 – Venezuela, Amazonas State, 4°58.838'N, 67°44.341'W; 95m, Comunidad Caño Gato, on Rio Sipapo.

Distribution: Neotropical: Venezuela.

***Chasmogenus australis* García, 2000**

*Chasmogenus australis* García, 2000a: 52 – Venezuela, Apure, Samán de Apure, Achaguas, 50 km NW of San Fernando de Apure; Short and Hebauer 2006: 331 [catalog]; Smith and Short 2020: 37 [new records].

Distribution: Neotropical: Brazil (Roraima), French Guiana, Guyana, Venezuela.

***Chasmogenus bariorum* García, 2000**

*Chasmogenus bariorum* García, 2000a: 49 – Venezuela, Zulia, Machiques de Perijá, Misión Angeles de Tukuko, El Manantial, 36 km SW of Machiques; Short and Hebauer 2006: 331 [catalog]; Smith and Short 2020: 40 [taxonomic treatment].

*Chasmogenus occidentalis* García, 2000a: 49; Venezuela, Zulia, Machiques de Perijá, Misión Angeles de Tukuko, El Manantial, 35 km SW of Machiques; Short and Hebauer 2006: 331 [catalog]; Smith and Short 2020: 40 [synonym].

*Chasmogenus yukparum* García, 2000a: 50 – Venezuela, Zulia, Machiques de Perijá, Misión Angeles de Tukuko, El Manantial, 35 km SW of Machiques; Short and Hebauer 2006: 331 [catalog]; Smith and Short 2020: 40 [synonym].

Distribution: Neotropical: Venezuela.

***Chasmogenus barrae* Short, 2005**

*Chasmogenus barrae* Short, 2005: 194 – Costa Rica, Guanacaste Prov. road to Barra Honda National Park, 6.6 km after junction with route 13; Short and Hebauer 2006: 331 [catalog].

Distribution: Neotropical: Costa Rica.

***Chasmogenus berbicensis* Smith & Short, 2020**

*Chasmogenus berbicensis* Smith & Short, 2020: 47 – Guyana, Region 6, 4°08.809'N, 58°14.232'W, Upper Berbice, Basecamp 1, margin of Berbice river.

*Chasmogenus* sp. B Short et al. 2018: 193.

Distribution: Neotropical: Guyana.

***Chasmogenus brownsbergensis* Smith & Short, 2020**

*Chasmogenus brownsbergensis* Smith & Short, 2020: 48 – Suriname, Brokopondo District, 04°56.871'N, 55°10.911'W, 462 m, Brownsberg Nature Park.

Distribution: Neotropical: Suriname.

***Chasmogenus cajuina* Alves, Clarkson & Lima, 2020**

*Chasmogenus cajuina* Alves, Clarkson & Lima, 2020: 580 – Brazil, Piauí, Castelo do Piaui, Cachoeira das Arraias, 5°11'28.5"S, 41°42'03.2"W.

Distribution: Neotropical: Brazil (Piauí).

***Chasmogenus castaneus* Smith & Short, 2020**

*Chasmogenus castaneus* Smith & Short, 2020: 50 – Venezuela, Zulia State, 09°50.490'N, 72°49.310'W, 270m, Perijá National Park, Tukuko, Rio Manantial.

Distribution: Neotropical: Venezuela.

***Chasmogenus clavijoi* Smith & Short, 2020**

*Chasmogenus clavijoi* Smith & Short, 2020: 53 – Venezuela, Guárico State, 8°8.296'N, 66°24.459'W, San Nicolasito Field Station.

Distribution: Neotropical: Venezuela.

***Chasmogenus cremnobates* (Spangler, 1979)**

*Dieroxenus cremnobates* Spangler, 1979: 754 – Ecuador, Napo, Baeza, 72 km E; Hansen 1999: 173 [catalog].

*Chasmogenus cremnobates* (Spangler, 1979); Girón and Short 2018: 155 [new combination].

Distribution: Neotropical: Ecuador.

***Chasmogenus cuspifer* Smith & Short, 2020**

*Chasmogenus cuspifer* Smith & Short, 2020: 54 – Venezuela, Zulia State, 9°50.490'N, 72°49.310'W, 270 m, Perijá N.P. Tukuko, Río Manantial.

Distribution: Neotropical: Venezuela.

***Chasmogenus flavomarginatus* Smith & Short, 2020**

*Chasmogenus flavomarginatus* Smith & Short, 2020: 55 – Venezuela, Barinas State, 8°48.424'N, 70°31.139'W, 992m, ca. 13km NW Barinitas.

Distribution: Neotropical: Venezuela.

***Chasmogenus fluminensis* Clarkson & Ferreira-Jr, 2014**

*Chasmogenus fluminensis* Clarkson & Ferreira-Jr, 2014b: 484 – Brazil Rio de Janeiro, Rio de Janeiro, Parque Nacional da Tijuca, 22°58'13"S, 43°15'25"W.

Distribution: Neotropical: Brazil (Rio de Janeiro).

***Chasmogenus fragilis* Sharp, 1882**

*Chasmogenus fragilis* Sharp, 1882: 73 – Guatemala, San Gerónimo; Fernández, 1986: 190 [lectotype designation; redescription]; Hansen 1999b: 174 [catalog]; Short 2005: 195 [taxonomic treatment].

Helochares (Chasmogenus) fragilis (Sharp, 1882); Knisch 1924a: 195 [catalog].

Chasmogenus (Chasmogenus) fragilis (Sharp, 1882); Hebauer 1992: 84 [taxonomic treatment].

Distribution: Neotropical: Guatemala, Panama.

***Chasmogenus gato* Smith & Short, 2020**

*Chasmogenus gato* Smith & Short, 2020: 56 – Venezuela, Amazonas State, 4°58.838'N, 67°44.341'W, 95m, Comunidad Caño Gato on Rio Sipapo.

Distribution: Neotropical: Venezuela.

***Chasmogenus guianensis* Smith & Short, 2020**

*Chasmogenus guianensis* Smith & Short, 2020: 58 – Suriname, Sipaliwini District, 2.47700°N, 55.62941°W, 275 m, Camp 1, Upper Palumeu.

*Chasmogenus* sp. X Short 2013: 87 (in part); Short and Kadosoe 2011: 87 (in part).

Distribution: Neotropical: Guyana, Suriname.

***Chasmogenus ignotus* Smith & Short, 2020**

*Chasmogenus ignotus* Smith & Short, 2020: 60 – Brazil, Amazonas, Manaus, -2.93079, -59.97514, 75 m, Ducke Reserve, near Station.

Distribution: Neotropical: Brazil (Amazonas).

***Chasmogenus itatiaia* Clarkson & Ferreira-Jr, 2014**

*Chasmogenus itatiaia* Clarkson & Ferreira-Jr, 2014b: 487 – Brazil – Rio de Janeiro, Itatiaia, Parque Nacional de Itatiaia, Poça no caminho das Agulhas Negras, 22°23'05.4"S, 44°40'41.7"W.

Distribution: Neotropical: Brazil (Minas Gerais, Rio de Janeiro).

***Chasmogenus ligulatus* Smith & Short, 2020**

*Chasmogenus ligulatus* Smith & Short, 2020: 61 – Suriname, Sipaliwini District, 2.97731N, 55.38500W, 200 m, Camp 4 (low), Kasikasima.

*Chasmogenus* sp. X Short 2013: 87 (in part).

Distribution: Neotropical: Suriname.

***Chasmogenus lilianae* Clarkson & Ferreira-Jr, 2014**

*Chasmogenus lilianae* Clarkson & Ferreira-Jr, 2014b: 489 – Brazil, Rio de Janeiro, Nova Friburgo, Macaé de Cima, Tributário de 1a Ordem do Rio Macaé, Casa amarela, campo das hortênsias.

Distribution: Neotropical: Brazil (Rio de Janeiro).

***Chasmogenus lineatus* Smith & Short, 2020**

*Chasmogenus lineatus* Smith & Short, 2020: 64 – Venezuela, Guárico State, 9°46.320'N, 67°21.177'W, 280m, Río San Antonio, N. Dos Caminos.

Distribution: Neotropical: Venezuela.

***Chasmogenus lorenzo* Short, 2005**

*Chasmogenus lorenzo* Short, 2005: 195; Costa Rica – Alajuela Province, small stream near Rio San Lorenzo, 6km from Los Lagos; Short and Hebauer 2006: 331 [catalog].

Distribution: Neotropical: Costa Rica.

***Chasmogenus pandus* Smith & Short, 2020**

*Chasmogenus pandus* Smith & Short, 2020: 68 – Suriname, Para District, Zanderij, near Guesthouse, 05°27.5'N, 055°13.0'W.

Distribution: Neotropical: Brazil (Amapá), French Guiana, Suriname.

***Chasmogenus rufinasus* (Knisch, 1924)**

Helochares (Chasmogenus) rufinasus Knisch, 1924c: 124 – Ecuador (Guayaquil).

*Chasmogenus rufinasus* (Knisch, 1924); Fernández 1986: 193 [new combination; taxonomic treatment]; Hansen 1999b: 175 [catalog].

Distribution: Neotropical: Ecuador.

***Chasmogenus ruidus* Short, 2005**

*Chasmogenus ruidus* Short, 2005: 196 – Costa Rica, Limón Province, Sector Cerro Cocori, Farm of Elias Rojas, A. C. Tortuguero; Short and Hebauer 2006: 331 [catalog].

Distribution: Neotropical: Costa Rica.

***Chasmogenus sapucay* Fernández, 1986**

*Chasmogenus sapucay* Fernández, 1986: 192 – Paraguay, Sapucay; Hansen 1999b: 176 [checklist]; Clarkson and Ferreira-Jr 2014b: 492 [new record].

Distribution: Neotropical: Argentina, Brazil (Pará, Rio de Janeiro), Paraguay.

***Chasmogenus schmits* Smith & Short, 2020**

*Chasmogenus schmits* Smith & Short, 2020: 69 – Suriname, Sipaliwini District, 2°10.521'N, 56°47.244'W, 228 m, on Kutari River.

*Chasmogenus* sp. X Short and Kadosoe 2011: 87 (in part).

Distribution: Neotropical: Suriname.

***Chasmogenus schoedli* Short, 2005**

*Chasmogenus schoedli* Short, 2005: 197 – Costa Rica, Guanacaste, 9 km S Santa Cecilia, Pitilla Station; Short and Hebauer 2006: 331 [catalog].

Distribution: Neotropical: Costa Rica.

***Chasmogenus sinnamarensis* Smith & Short, 2020**

*Chasmogenus sinnamarensis* Smith & Short, 2020: 70 – French Guyana, Road Petit Saut, Crique Eau Claire.

Distribution: Neotropical: French Guyana.

***Chasmogenus tafelbergensis* Smith & Short, 2020**

*Chasmogenus tafelbergensis* Smith & Short, 2020: 71 – Suriname, Sipaliwini District, 3°55.600'N, 56°11.300'W, 600 m, CSNR: Tafelberg Summit, nr Augustus Creek Camp, pools & creeks on trail into Arrowhead basin.

Distribution: Neotropical: Suriname.

***Chasmogenus ubatuba* Clarkson & Ferreira-Jr, 2014**

*Chasmogenus ubatuba* Clarkson & Ferreira-Jr, 2014b: 491 – Brasil, São Paulo, Ubatuba, Parque Estadual da Serra do Mar, Núcleo Picinguaba.

Distribution: Neotropical: Brazil (São Paulo).

***Chasmogenus undulatus* Smith & Short, 2020**

*Chasmogenus undulatus* Smith & Short, 2020: 73 – Guyana, Region VIII, 5°18.261'N, 59°50.257'W, 687 m, Ayanganna Airstrip, trail from airstrip to Ayanganna.

*Chasmogenus* sp. A Short et al. 2018: 193.

Distribution: Neotropical: Guyana.

### *Colossochares* Girón & Short, gen. nov.

***Colossochares ellipticus* (d’Orchymont, 1933) comb. nov.**

*Helochares ellipticus* Régimbart, 1907: 47 – Gabon, Lambarené, Cape Lopez, Rembo Nkomi; [misinterpretation of *Hydrophilus ellipticus* Fabricius, 1801].

*Helochares ellipticus* Régimbart, 1907; d’Orchymont 1933: 306 [new name]; Hebauer 2003: 129.

Helochares (s. str.) ellipticus d’Orchymont, 1933; Hansen 1999b: 160 [catalog].

Helochares (s. str.) ellipticus Régimbart, 1907; Balfour-Browne 1950b: 59 [faunistic treatment]; Hebauer 1996: 6 [taxonomic treatment]; Hebauer 2006a: 25 [checklist].

Distribution: Afrotropical: Benin, Burkina Faso, Cameroon, Democratic Republic of the Congo, Ethiopia, Gabon, Ghana, Guinea, Ivory Coast, Liberia, Nigeria, Republic of the Congo, Uganda.

***Colossochares satoi* (Hebauer, 2003) comb. nov.**

Helochares (s. str.) satoiHebauer 2003a: 129 – Malawi: “Balaka env.”; Hebauer 2005: 39; Hebauer 2006a: 25 [checklist]; Short and Hebauer 2006: 336 [catalog].

Distribution: Afrotropical: Malawi.

### *Crephelochares* Kuwert, 1890

***Crephelochares abnormalis* (Sharp, 1890)**

*Philydrus abnormalis* Sharp, 1890: 351 – Sri Lanka, Colombo [“Ceylon: Colombo”]; [specific rank confirmed by d’Orchymont 1937d: 7; not synonym of *livornicus* Kuwert, as in d’Orchymont 1925: 70].

Helochares (Chasmogenus) abnormalis (Sharp, 1890); Knisch 1921: 68 [catalog].

Helochares (Crephelochares) abnormalis (Sharp, 1890); d’Orchymont 1937d: 7 [checklist]; d’Orchymont 1939a: 159 [taxonomic treatment].

Chasmogenus (Crephelochares) abnormalis (Sharp, 1890); Hebauer 1992: 68 [taxonomic treatment].

Enochrus (Lumetus) abnormicollis (Sharp, 1890); Zaitzev 1908: 385 [catalog – error for *abnormalis* Sharp, 1890].

*Phylhydrus ferrugatus* Régimbart, 1903b: 57 – Vietnam [“Cochinchine”] (My Tho); Indonesia (Sumatra); d’Orchymont 1939a: 159 [synonymy; not synonym of *livornicus* Kuwert, as in d’Orchymont 1925: 70).

Enochrus (Lumetus) ferrugatus Régimbart, 1903; Zaitzev 1908: 386 [catalog].

Helochares (Chasmogenus) ferrugatus Régimbart, 1903; Knisch 1924a: 195 [catalog].

*Philhydrus nigritulus* Régimbart, 1903b: 57 – Vietnam (Ho Chi Minh [“Saigon”], My Tho); Cambodia (Phnom Penh); Indonesia (Sumatra); Knisch 1924a: 195 [transferred to *Helochares*, thereby becoming a junior secondary homonym of *Helochares nigritulus* Kuwert, 1889]. Permanently invalid: replaced before 1961 (ICZN Code Art. 59b); d’Orchymont 1939a: 159 [synonymy].

Enochrus (Lumetus) nigritulus Régimbart, 1903; Zaitzev 1908a: 388 [catalog].

Helochares (Chasmogenus) regimbarti Knisch, 1924a: 195 (replacement name for *nigritulus* Régimbart); d’Orchymont 1939a: 159 [synonymy].

*Chasmogenus abnormalis* (Sharp, 1890); Gentili et al. 1995: 210 [checklist]; Hansen 1999b: 173 [catalog]; Hansen 2004: 49 [checklist]; Hebauer and Ryndevich 2005: 46 [new record]; Fikáček et al. 2015: 61 [catalog]; Devi et al. (2016) [redescription; lectotype designation]; Jia and Tang 2018a: 63 [new record].

*Crephelochares abnormalis* (Sharp, 1890); Short et al. 2021: 12 [new combination].

Distribution: Indo-Malayan: Cambodia, China (Guangdong, Taiwan), Indonesia (Borneo, Java, Sulawesi, Sumatra), Laos, Sri Lanka, Thailand, Vietnam. Palearctic: Japan.

***Crephelochares africanus* (d’Orchymont, 1937)**

Helochares (Crephelochares) africanus d’Orchymont, 1937d: 7 – Mozambique, Nova Chupanga nr Chemba; d’Orchymont 1939a: 163 [taxonomic treatment]; Balfour-Browne 1950b: 58 [faunistic treatment].

Chasmogenus (Crephelochares) africanus (d’Orchymont, 1937); Hebauer 1992: 69 [taxonomic treatment]; Hebauer 1995a: 265 [faunistic treatment]; Hebauer 2006a: 27 [checklist].

*Chasmogenus africanus* (d’Orchymont, 1937); Hansen 1999b: 174 [catalog].

*Crephelochares africanus* (d’Orchymont, 1937); Short et al. 2021: 12 [new combination].

Distribution: Afrotropical: Botswana, Cameroon, Democratic Republic of the Congo, Gambia, Ghana, Guinea, Mozambique, Namibia, Niger, Nigeria, Senegal, Republic of South Africa, Sudan, Uganda, Zimbabwe.

***Crephelochares balkei* (Short, 2010)**

*Chasmogenus balkei* Short, 2010: 301 – Fiji (Vanua Levu); Short and Fikáček 2011: 89 [catalog].

*Crephelochares balkei* (Short); Short et al. 2021: 12 [new combination].

Distribution: Australasian: Fiji (Vanua Levu).

***Crephelochares cattienus* (Hebauer, 2002)**

*Chasmogenus cattienus* Hebauer, 2002b: 9 – Vietnam, S Cát Tiên, 120 km NNE Ho Chi Minh, Cát Tiên National Park.

*Crephelochares cattienus* (Hebauer, 2002); Short et al. 2021: 12 [new combination].

Distribution: Indo-Malayan: Vietnam.

***Crephelochares irianus* (Hebauer, 2001)**

*Chasmogenus irianus* Hebauer, 2001a: 15 – Indonesia, Papua [West New Guinea], Fak-Fak, IR 27, Kali Mati 4 km N of Fak-Fak.

*Crephelochares irianus* (Hebauer, 2001); Short et al. 2021: 12 [new combination].

Distribution: Indo-Malayan: Indonesia (Papua).

***Crephelochares larsi* (Hebauer, 1995)**

Chasmogenus (Crephelochares) larsi Hebauer, 1995b: 8 – Malaysia, Cameron Highlands, Tanah Rata, G. Jasar track 11.

*Chasmogenus larsi* Hebauer, 1995; Hansen 1999b: 174 [catalog].

*Crephelochares larsi* (Hebauer, 1995); Short et al. 2021: 12 [new combination].

Distribution: Indo-Malayan: Malaysia (Peninsula).

***Crephelochares livornicus* (Kuwert, 1890)**

Helochares (Crephelochares) livornicus Kuwert, 1890a: 38 – Italy, Livorno; Heyden 1891: 67 [catalog]; d’Orchymont 1939a: 158 [taxonomic treatment].

*Crephelochares livornicus* (Kuwert, 1890); Kuwert 1890b: 327 (also as “n. sp.”).

Helochares (Crepidelochares) livornicus Kuwert, 1890; Ganglbauer 1904: 248 [faunistic treatment].

Helochares (Chasmogenus) livornicus Kuwert, 1890; Knisch 1924a: 195 [catalog]; d’Orchymont 1925: 70 [taxonomic treatment]; d’Orchymont 1928: 106 [faunistic treatment].

Chasmogenus (Crephelochares) livornicus (Kuwert, 1890); Hebauer 1992: 70 [taxonomic treatment]

*Chasmogenus livornicus* (Kuwert, 1890); Hebauer 1994: 111 [faunistic treatment]; Hansen 1999b: 174 [catalog]; Hansen 2004: 49 [checklist]; Fikáček et al. 2015: 61 [catalog].

Distribution: Palearctic: Bosnia, Croatia, Greece, Israel, Italy, Serbia and Montenegro, Spain, Tunisia, Turkey.

***Crephelochares luctuosus* (d’Orchymont, 1939)**

Helochares (Crephelochares) luctuosus d’Orchymont, 1939a: 164 – Gabon; Hebauer 1988: 157 [faunistic treatment].

Chasmogenus (Crephelochares) luctuosus (d’Orchymont, 1939); Hebauer 1992: 71 [taxonomic treatment]; Hebauer 2006a: 27 [checklist].

*Chasmogenus luctuosus* (d’Orchymont, 1939); Hansen 1999: 174 [catalog].

*Crephelochares luctuosus* (d’Orchymont, 1939); Short et al. 2021: 12 [new combination].

Distribution: Afrotropical: Cameroon, Democratic Republic of the Congo (in doubt, Hebauer 2006a: 27), Gabon, Ghana (in doubt, Hebauer 2006a: 27), Guinea, Namibia, Senegal.

***Crephelochares lycetus* (d’Orchymont, 1939)**

Helochares (Crephelochares) lycetus d’Orchymont, 1939a: 163; Kenya [“Afrique orientale anglaise”], Taveta.

Chasmogenus (Crephelochares) lycetus (d’Orchymont, 1939); Hebauer 1992: 72 [taxonomic treatment]; Hebauer 1995a: 266 [faunistic treatment]; Hebauer 2006a: 27 [checklist].

*Chasmogenus lycetus* (d’Orchymont, 1939); Hansen 1999: 174 [catalog].

*Crephelochares lycetus* (d’Orchymont, 1939); Short et al. 2021: 12 [new combination].

Distribution: Afrotropical: Angola, Benin, Botswana, Kenya, Namibia, Republic of South Africa, Tanzania, Zambia, Zimbabwe.

***Crephelochares mauritiensis* (Balfour-Browne, 1958)**

Helochares (Crephelochares) mauritiensis Balfour-Browne, 1958b: 143 – Mauritius, Les Mares.

Chasmogenus (Crephelochares) mauritiensis (Balfour-Browne, 1958); Hebauer 1992: 72 [taxonomic treatment]; Hebauer 2006a: 27 [checklist].

*Chasmogenus mauritiensis* (Balfour-Browne, 1958); Hansen 1999: 174 [catalog].

*Crephelochares mauritiensis* (Balfour-Browne, 1958); Short et al. 2021: 12 [new combination].

Distribution: Afrotropical: Mauritius.

***Crephelochares molinai* (Hebauer, 1992)**

Chasmogenus (Crephelochares) molinai Hebauer, 1992: 73 – Congo, Loudima; Hebauer 1995a: 266 [faunistic treatment]; Hebauer 2006a: 27 [checklist].

*Chasmogenus molinai* Hebauer, 1992; Hansen 1999: 174 [catalog].

*Crephelochares molinai* (Hebauer, 1992); Short et al. 2021: 12 [new combination].

Distribution: Afrotropical: Democratic Republic of the Congo, Namibia.

***Crephelochares mollis* (Régimbart, 1903)**

*Philhydrus mollis* Régimbart, 1903a: 32 – Madagascar, “Baie d’Antongil; pays Androy”; (specific rank confirmed by d’Orchymont, 1937d: 7; not synonym of *abnormalis* Sharp, as in Scott 1913: 205; not synonym of *livornicus* Kuwert, as in d’Orchymont 1925: 70).

Enochrus (Lumetus) mollis (Régimbart, 1903); Zaitzev, 1908: 387 [catalog].

Helochares (Crephelochares) mollis (Régimbart, 1903); d’Orchymont, 1937d: 7; d’Orchymont 1939a: 161 [taxonomic treatment]; Hebauer 1988: 157 [faunistic treatment].

*Philydrus abnormalis*; Scott 1913: 205 [misinterpret. of *Philydrus abnormalis* Sharp]; d’Orchymont 1939a: 161 [synonymy].

Chasmogenus (Crephelochares) mollis (Régimbart, 1903); Hebauer, 1992: 74 [taxonomic treatment]; Hebauer 2006a: 27 [checklist].

*Chasmogenus mollis* (Régimbart, 1903); Hansen 1999: 174 [catalog].

*Crephelochares mollis* (Régimbart, 1903); Short et al. 2021: 12 [new combination].

Distribution: Afrotropical: Madagascar, Seychelles (Aldabra).

***Crephelochares molluscus* (Hebauer, 1992)**

Chasmogenus (Crephelochares) molluscus Hebauer, 1992: 75 – Tanzania (Lake Manyara); Hebauer 2006a: 27 [checklist].

*Chasmogenus molluscus* Hebauer, 1992; Hansen 1999: 175 [catalog].

*Crephelochares molluscus* (Hebauer), 1992; Short et al. 2021: 12 [new combination].

Distribution: Afrotropical: Tanzania.

***Crephelochares nitescens* (Fauvel, 1883)**

*Philydrus nitescens* Fauvel, 1883: 354 – New Caledonia (Anse Vata).

Enochrus (Lumetus) nitescens Fauvel, 1883; Zaitzev 1908: 388.

Helochares (Crephelochares) nitescens (Fauvel, 1883); d’Orchymont 1939a: 157 [taxonomic treatment].

Helochares (Chasmogenus) nitescens (Fauvel, 1883); Balfour-Browne 1945: 117 [checklist].

*Helochares nitescens* (Fauvel, 1883); Anderson 1976: 223 [description of immature stages].

*Chasmogenus nitescens* (Fauvel, 1883); Hansen 1991: 156 [examined species]; Watts 1995: 116 [lectotype designated; redescription]; Archangelsky 1997: 55 [redescription of immature stages]; Hansen 1999: 175 [catalog]; Short (2010) [new record].

Chasmogenus (Crephelochares) nitescens (Fauvel, 1883); Hebauer 1992: 75 [taxonomic treatment].

*Crephelochares nitescens* (Fauvel, 1883); Short et al. 2021: 12 [new combination].

Distribution: Australasian: Australia (New South Wales, Northern Territory, Queensland), Fiji (Viti Levu), New Caledonia, Papua New Guinea.

***Crephelochares omissus* (Hebauer, 1995)**

Chasmogenus (Crephelochares) omissus Hebauer, 1995a: 266 – Namibia, East Caprivi, Mudumu National Park, Nakatwa, 18°10'S, 23°26'E; Hebauer 1995a: 266 [faunistic treatment]; Hebauer 2006a: 27 [checklist].

*Chasmogenus omissus* Hebauer, 1995; Hansen 1999: 175 [catalog].

*Crephelochares omissus* (Hebauer, 1995); Short et al. 2021: 12 [new combination].

Distribution: Afrotropical: Namibia.

***Crephelochares orbus* (Watanabe, 1987)**

Helochares (Crephelochares) orbus Watanabe, 1987: 12; Japan, Honshu, Gumma-ken, Tatebayashi-shi, Hanetsuku.

Chasmogenus (Crephelochares) orbus (Watanabe, 1987); Hebauer, 1992: 76 [taxonomic treatment].

*Chasmogenus orbus* (Watanabe, 1987); Hansen 1999: 175 [catalog]; Hansen 2004: 49 [checklist]; Fikáček et al. 2015: 61 [catalog]; Jia and Tang 2018a: 63 [new record].

*Crephelochares orbus* (Watanabe, 1987); Short et al. 2021: 12 [new combination].

Distribution: Indo-Malayan: China (Hong Kong). Palearctic: Japan.

***Crephelochares paramollis* (Hebauer, 1992)**

Chasmogenus (Crephelochares) paramollis Hebauer, 1992: 76 – Tanzania, Usa river; Hebauer 1995a: 266 [faunistic treatment; new records]; Hebauer 2006a: 27 [checklist; new records].

*Chasmogenus paramollis* Hebauer, 1992; Hansen 1999: 175 [catalog].

*Crephelochares paramollis* (Hebauer, 1992); Short et al. 2021: 12 [new combination].

Distribution: Afrotropical: Cameroon, Democratic Republic of the Congo, Gabon, Ghana, Guinea, Kenya, Namibia, Republic of South Africa [Transvaal], Zambia, Zimbabwe.

***Crephelochares parorbus* (Jia & Tang, 2018)**

*Chasmogenus parorbus* Jia & Tang, 2018a: 61 – China, Yünnan Prov., Yingjiang, Tongbiguan, Kaibangyahu, 24.58°N, 97.67°E.

*Crephelochares parorbus* (Jia & Tang); Short et al. 2021: 12 [new combination].

Distribution: Indo-Malayan: China (Yunnan).

***Crephelochares patrizii* (Balfour-Browne, 1948)**

Helochares (Crephelochares) patrizii Balfour-Browne, 1948: 830 – Somalia [Italian Somaliland], Giuba, Belet Amin.

Chasmogenus (Crephelochares) patrizii (Balfour-Browne, 1948); Hebauer 1992: 77 [taxonomic treatment]; Hebauer 2006a: 27 [checklist].

*Chasmogenus patrizii* (Balfour-Browne, 1948); Hansen 1999: 175 [catalog].

*Crephelochares patrizii* (Balfour-Browne, 1948); Short et al. 2021: 12 [new combination].

Distribution: Afrotropical: Cameroon, Kenya, Mozambique, Somalia, Republic of South Africa, Sudan, Tanzania, Uganda, Zambia, Zimbabwe.

***Crephelochares punctulatus* (Short, 2010)**

*Chasmogenus punctulatus* Short, 2010: 303 – Fiji, Viti Levu, Nadarivatu; Short and Fikáček 2011: 89 [checklist].

*Crephelochares punctulatus* (Short, 2010); Short et al. 2021: 12 [new combination].

Distribution: Australasian: Fiji (Viti Levu).

***Crephelochares rhodesiensis* (Hebauer, 2006)**

Chasmogenus (Crephelochares) rhodesiensis Hebauer, 2006b: 18 – Zambia, Copperbelt, W of Kapiri Mposhi.

*Chasmogenus rhodesiensis* Hebauer, 2006; Short and Fikáček 2011: 89 [checklist].

*Crephelochares rhodesiensis* (Hebauer, 2006); Short et al. 2021: 12 [new combination].

Distribution: Afrotropical: Zambia.

***Crephelochares ruandanus* (Balfour-Browne, 1957)**

Helochares (Crephelochares) ruandanus Balfour-Browne, 1957: 22 – Rwanda, Kibuye.

Chasmogenus (Crephelochares) ruandanus (Balfour-Browne, 1957); Hebauer 1992: 78 [taxonomic treatment]; Hebauer 2006a: 27 [checklist].

*Chasmogenus ruandanus* (Balfour-Browne, 1957); Hansen 1999: 175 [catalog].

*Crephelochares ruandanus* (Balfour-Browne, 1957); Short et al. 2021: 12 [new combination].

Distribution: Afrotropical: Burundi, Kenya, Rwanda.

***Crephelochares rubellus* (Hebauer, 1992)**

Chasmogenus (Crephelochares) rubellus Hebauer, 1992: 79 – Senegal, village Sare Sara, 21 km ESE Kolda; Hebauer 2006a: 27 [checklist].

*Chasmogenus rubellus* Hebauer, 1992; Hansen 1999: 175 [catalog].

*Crephelochares rubellus* (Hebauer, 1992); Short et al. 2021: 12 [new combination].

Distribution: Afrotropical: Gambia, Senegal.

***Crephelochares rubricollis* (Régimbart, 1903)**

*Philhydrus rubricollis* Régimbart, 1903b: 58 – Indonesia, Sumatra, Palembang; (specific rank confirmed by d’Orchymont, 1925: 71; not synonym of *abnormalis* Kuwert, as in Knisch 1921: 68).

Enochrus (Lumetus) rubricollis (Régimbart, 1903); Zaitzev 1908: 389.

Helochares (Chasmogenus) rubricollis (Régimbart, 1903); d’Orchymont 1925: 71 [taxonomic treatment].

Helochares (Crephelochares) rubricollis (Régimbart, 1903); d’Orchymont 1939a: 162 [taxonomic treatment].

Chasmogenus (Crephelochares) rubricollis (Régimbart, 1903); Hebauer 1992: 79 [taxonomic treatment].

Helochares (Chasmogenus) abnormalis Sharp, 1890; Knisch 1921: 68; misinterpret. of *Philydrus abnormalis* Sharp, 1890; d’Orchymont, 1939a: 162 [synonymy].

*Chasmogenus rubricollis* (Régimbart, 1903); Hansen 1999: 175 [catalog].

*Crephelochares rubricollis* (Régimbart, 1903); Short et al. 2021: 12 [new combination].

Distribution: Indo-Malayan: Indonesia (Borneo, Sumatra).

***Crephelochares rudis* (Hebauer, 1992)**

Chasmogenus (Crephelochares) rudis Hebauer, 1992: 80 – Congo, Kindamba, Meya, Bangou forest; Hebauer 2006a: 27 [checklist].

*Chasmogenus rudis* Hebauer, 1992; Hansen 1999: 175 [catalog].

*Crephelochares rudis* (Hebauer, 1992); Short et al. 2021: 12 [new combination].

Distribution: Afrotropical: Congo [Kindamba locality in both Democratic Republic of the Congo and Republic of the Congo].

***Crephelochares rusticus* (d’Orchymont, 1939)**

Helochares (Crephelochares) rusticus d’Orchymont, 1939a: 165 – Gabon.

Chasmogenus (Crephelochares) rusticus (d’Orchymont, 1939); Hebauer 1992: 81 [taxonomic treatment]; Hebauer 2006a: 27 [checklist].

*Chasmogenus rusticus* (d’Orchymont, 1939); Hansen 1999: 175 [catalog].

*Crephelochares rusticus* (d’Orchymont, 1939); Short et al. 2021: 12 [new combination].

Distribution: Afrotropical: Gabon, Ghana.

***Crephelochares rutiloides* (d’Orchymont, 1939)**

Helochares (Crephelochares) rutiloides d’Orchymont, 1939a: 323 – Gabon.

Chasmogenus (Crephelochares) rutiloides (d’Orchymont, 1939); Hebauer 1992: 82 [taxonomic treatment]; Hebauer 1995a: 266 [faunistic treatment]; Hebauer 2006a: 27 [checklist].

*Chasmogenus rutiloides* (d’Orchymont, 1939); Hansen 1999: 175 [catalog].

*Crephelochares rutiloides* (d’Orchymont, 1939); Short et al. 2021: 12 [new combination].

Distribution: Afrotropical: Botswana, Cameroon, Democratic Republic of the Congo, Gabon, Gambia, Ghana, Namibia, Zambia.

***Crephelochares rutilus* (d’Orchymont, 1925)**

Helochares (Chasmogenus) rutilus d’Orchymont, 1925a: 71. – Gabon; d’Orchymont 1939a: 163 [taxonomic treatment].

Helochares (Crephelochares) rutilus d’Orchymont, 1925; d’Orchymont 1928: 107 [faunistic treatment]; d’Orchymont 1937d: 7 [checklist].

Chasmogenus (Crephelochares) rutilus (d’Orchymont, 1925); Hebauer 1992: 82 [new combination; taxonomic treatment]; Hebauer 2006a: 27 [checklist; new records].

*Chasmogenus rutilus* (d’Orchymont, 1925); Hansen 1991: 156 [examined species]; Hansen 1999: 176 [catalog].

Helochares (Chasmogenus) abnormalis Sharp, 1890; Knisch 1921a: 68 [misinterpretation of *Philydrus abnormalis* Sharp, 1890]; d’Orchymont, 1939a: 163 [synonymy].

*Crephelochares rutilus* (d’Orchymont, 1925); Short et al. 2021: 12 [new combination].

Distribution: Afrotropical: Cameroon, Democratic Republic of the Congo, Gabon, Ghana, Nigeria, Republic of South Africa.

***Crephelochares szeli* (Hebauer, 1992)**

Chasmogenus (Crephelochares) szeli Hebauer, 1992: 84 – Ghana, Ashanti region, Kumashi, Nhiasu, 6°43'N, 1°36'W; Hebauer 2006a: 27 [checklist; new records].

*Chasmogenus szeli* Hebauer, 1992; Hansen 1999: 176 [catalog].

*Crephelochares szeli* (Hebauer, 1992); Short et al. 2021: 12 [new combination].

Distribution: Afrotropical: Democratic Republic of the Congo, Ghana, Liberia, Nigeria, Sierra Leone, Uganda.

### *Crucisternum* Girón & Short, 2018

***Crucisternum escalera* Girón & Short, 2018**

*Crucisternum escalera* Girón & Short, 2018: 120 – Venezuela, Bolívar State, along La Escalera, 6°2'10.5"N, 61°23'57.8"W.

Distribution: Neotropical: Venezuela.

***Crucisternum ouboteri* Girón & Short, 2018**

*Crucisternum ouboteri* Girón & Short, 2018: 121 – Suriname, Sipaliwini District, Brownsberg Nature Park, 04°56.871'N, 55°10.911'W.

Distribution: Neotropical: French Guiana, Guyana, Suriname, Venezuela.

***Crucisternum queneyi* Girón & Short, 2018**

*Crucisternum queneyi* Girón & Short, 2018: 123 – French Guiana, Sinnamary.

Distribution: Neotropical: French Guiana.

***Crucisternum sinuatus* Girón & Short, 2018**

*Crucisternum sinuatus* Girón & Short, 2018: 124 – Brazil, Minas Gerais, Lassance, Cachoeira da Palmeira, -17.83384, -44.50515.

Distribution: Neotropical: Brazil (Minas Gerais, Pará).

***Crucisternum toboganensis* Girón & Short, 2018**

*Crucisternum toboganensis* Girón & Short, 2018: 126 – Venezuela, Amazonas, Puerto Ayacucho (40 km S), El Tobogán, Caño Coromoto.

Distribution: Neotropical: Venezuela.

***Crucisternum vanessae* Girón & Short, 2018**

*Crucisternum vanessae* Girón & Short, 2018: 127 – Suriname, Sipaliwini District, Central Suriname Nature Reserve: Tafelberg Summit, near Caiman Creek Camp, 3°53.942'N, 56°10.849'W.

Distribution: Neotropical: Suriname.

***Crucisternum xingu* Girón & Short, 2018**

*Crucisternum xingu* Girón & Short, 2018: 131 – Brazil, Pará, Rio Xingu Camp, ca. 60 km S Altamira, 52°22'W, 3°39'S.

Distribution: Neotropical: Brazil (Pará).

### *Ephydrolithus* Girón & Short, 2019

***Ephydrolithus hamadae* Girón & Short, 2019**

*Ephydrolithus hamadae* Girón & Short, 2019: 130 – Brazil, Minas Gerais, Lassance, Cachoeira da Palmeira; 17.83384S, 44.50515W.

Distribution: Neotropical: Brazil (Minas Gerais).

***Ephydrolithus minor* Girón & Short, 2019**

*Ephydrolithus minor* Girón & Short, 2019: 130 – Brazil, Bahia, Abaíra, Pico do Barbado W of Catolés, 13.29053S, 41.90489W.

Distribution: Neotropical: Brazil (Bahia).

***Ephydrolithus ogmos* Girón & Short, 2019**

*Ephydrolithus ogmos* Girón & Short, 2019: 131- Brazil, Brazil, Bahia, Abaíra, Pico do Barbado W of Catolés, 13.29053S, 41.90489W.

Distribution: Neotropical: Brazil (Bahia).

***Ephydrolithus spiculatus* Girón & Short, 2019**

*Ephydrolithus spiculatus* Girón & Short, 2019: 132 – Brazil, Minas Gerais, Lassance, Cachoeira da Palmeira, 17.83384S, 44.50515W.

Distribution: Neotropical: Brazil (Minas Gerais).

***Ephydrolithus teli* Girón & Short, 2019**

*Ephydrolithus teli* Girón & Short, 2019: 132 – Brazil, Bahia, Abaíra, Pico do Barbado, W of Catolés; 13.29053S, 41.90489W.

Distribution: Neotropical: Brazil (Bahia, Minas Gerais).

### *Globulosis* García, 2001

***Globulosis hemisphericus* García, 2001**

*Globulosis hemisphericus* García, 2001: 156 – Venezuela, Bolívar, Municipio Sifontes, Tierra Blanca Pantano; Short et al. 2017: 275 [new records].

*Globulosis hemisphaericus* García [incorrect subsequent spelling]; Short and Hebauer 2006: 338 [catalog].

*Globulosis* sp. 1 Short and Kadosoe 2011: 89 [checklist]; Short 2013: 87 [checklist].

Distribution: Neotropical: Venezuela, Guyana, Suriname, Brazil (Amazonas, Pará).

***Globulosis flavus* Short, García & Girón, 2017**

*Globulosis flavus* Short, García & Girón, 2017: 277 – Venezuela, Amazonas State, nr. Iboruwa: “Tobogancito”, 5 48.141'N, 67 26.313'W.

Distribution: Neotropical: Venezuela.

### *Helobata* Bergroth, 1888

***Helobata amazonensis* Clarkson, Santos & Ferreira-Jr, 2016**

*Helobata amazonensis* Clarkson, Santos & Ferreira-Jr, 2016: 550 – Brazil, Amazonas, Itacoatiara, Ilha da Trinidade; Clarkson and Almeida 2018 [new records].

Distribution: Neotropical: Brazil (Amazonas, Roraima).

***Helobata aschnakiranae* Makhan, 2007**

*Helobata aschnakiranae* Makhan, 2007: 1 – Suriname (District Commwijne); Short and Fikáček 2011: 90 [catalog].

Distribution: Neotropical: Suriname.

***Helobata bitriangulata* García, 2000**

*Helobata bitriangulata* García, 2000c: 244 – Venezuela, Apure State, Achaguas, Samán de Apure; Short and Hebauer 2006: 335 [catalog].

Distribution: Neotropical: Venezuela.

***Helobata confusa* Fernández & Bachmann, 1987**

*Helobata confusa* Fernández & Bachmann, 1987: 155 – Paraguay (Asunción); Hansen 1999b: 173 [catalog].

Distribution: Neotropical: Argentina, Paraguay.

***Helobata corumbaensis* Fernández & Bachmann, 1987**

*Helobata corumbaensis* Fernández & Bachmann, 1987: 155 – Brazil (Mato Grosso, Corumbá); Hansen 1999b: 173 [catalog]; Clarkson et al. 2016: 555 [taxonomic treatment].

Distribution: Neotropical: Brazil (Mato Grosso, Mato Grosso do Sul).

***Helobata cossyphoides* (Bruch, 1915)**

*Helopeltis cossyphoides* Bruch, 1915: 458 – Argentina, Buenos Aires Province, La Plata, “Tiro Federal”; Fernández and Bachmann 1987: 153 [lectotype designation].

*Helobata cossyphoides* (Bruch, 1915); Fernández and Bachmann 1987: 151 [specific rank confirmed; not synonym of *striata* Brullé (= *larvalis* Horn), as in Knisch, 1924a: 223]; Hansen 1999b: 173 [catalog].

Distribution: Neotropical: Argentina.

***Helobata cuivaum* García, 2000**

*Helobata cuivaum* García, 2000c: 242 – Venezuela (Apure State, Achaguas, Samán de Apure); Short and Hebauer 2006: 335 [catalog].

Distribution: Neotropical: Venezuela.

***Helobata larvalis* (Horn, 1873)**

*Helopeltis larvalis* Horn, 1873: 137 – U.S.A. (Louisiana, California (Sonora)).

*Helopeltina larvalis* (Horn, 1873); Cockerell 1906a: 240.

*Helobata larvalis* (Horn, 1873); Cockerell, 1906b: 349; Hansen 1991: 293 [reinstated as valid name]; Jasper and Vogtsberger 1996: 56 [checklist]; Archangelsky 1997: 50 [description of immature stages]; Clarkson et al. 2016: 557 [taxonomic treatment]; Clarkson and Almeida 2018 [new records].

Hydrophilus (Philydrus) striatus Brullé, 1841: 58 (primary homonym of *Hydrophilus striatus* Turton, 1802 and *Hydrophilus striatus* Say, 1825).

*Helopeltis striatus* (Brullé, 1841); Bedel 1881b: XCIV [new combination].

Enochrus (Lumetus) striatus (Brullé, 1841); Zaitzev 1908: 389 [checklist].

*Helobata striata* (Brullé, 1841); Knisch, 1924a: 223 [catalog]; Spangler and Cross 1972 [description of eggs, egg case and first instar larva]; Fernández and Bachmann 1987: 53 [taxonomic treatment].

Distribution: Neotropical: Argentina, Bolivia, Brazil (Amazonas, Ceará, Mato Grosso, Mato Grosso do Sul, Minas Gerais), Cuba, Guatemala, Mexico, Paraguay, Venezuela. Nearctic: U.S.A. (California, Florida, Louisiana, Mississippi, North Carolina, South Carolina, Texas, Virginia).

***Helobata lilianae* García, 2000**

*Helobata lilianae* García, 2000c: 239 – Venezuela, Apure State, Achaguas, Saman de Apure; Short and Hebauer 2006: 335 [catalog].

Distribution: Neotropical: Venezuela.

***Helobata pantaneira* Clarkson, Santos & Ferreira-Jr, 2016**

*Helobata pantaneira* Clarkson, Santos & Ferreira-Jr, 2016: 553 – Brazil, Mato Grosso, Poconé.

Distribution: Neotropical: Brazil (Mato Grosso).

***Helobata perpunctata* Fernández & Bachmann, 1987**

*Helobata perpunctata* Fernández & Bachmann, 1987: 156 – Argentina (Chaco Province, San Bernardo); Hansen 1999b: 173.

Distribution: Neotropical: Argentina.

***Helobata quatipuru* Fernández & Bachmann, 1987**

*Helobata quatipuru* Fernández & Bachmann, 1987: 158 – Brazil, Pará State, Quatipurú; Hansen 1999b: 173 [catalog]; Clarkson et al. 2016: 558 [taxonomic treatment]; Clarkson and Almeida 2018 [new records].

Distribution: Neotropical: Brazil (Minas Gerais, Pará, Rio de Janeiro).

***Helobata soesilae* Makhan, 2007**

*Helobata soesilae* Makhan, 2007: 3 – Suriname, Nieuw Amsterdam; Short and Fikáček 2011: 90 [catalog].

Distribution: Neotropical: Suriname.

### *Helochares* Mulsant, 1844

***Helochares aeacus* Balfour-Browne, 1952**

*Helochares aeacus* Balfour-Browne, 1952b: 515 – Mauritania, “Hamdoun”.

Helochares (Hydrobaticus) aeacus Balfour-Browne, 1952; Hebauer 1996: 11 [listed]; Hansen 1999b: 164 [catalog]; Hebauer 2006a: 26 [checklist].

Distribution: Afrotropical: Mauritania.

***Helochares aethiopicus* d’Orchymont, 1939**

Helochares (Hydrobaticus) aethiopicus d’Orchymont, 1939c: 309 – Ethiopia [“Abyssinie”]; Hebauer 1996: 11 [taxonomic treatment]; Hansen 1999b: 164 [catalog]; Hebauer 2006a: 26 [checklist]; Salah and Régil Cueto 2017: 270 [excluded from Egypt checklist].

Distribution: Afrotropical: Ethiopia.

***Helochares alberti* d’Orchymont, 1943**

Helochares (Hydrobaticus) alberti d’Orchymont, 1943a: 10 – Zaire [Congo Belge], Madimba; Hebauer 1996: 11 [taxonomic treatment]; Hansen 1999b: 164 [catalog]; Hebauer 2006a: 26 [checklist].

Distribution: Afrotropical: Democratic Republic of the Congo [Zaire], Gabon, Republic of the Congo, “West Africa (Uelleburg)”.

***Helochares alcimus* d’Orchymont, 1943**

Helochares (Hydrobaticus) alcimus d’Orchymont, 1943a: 12 – Democratic Republic of the Congo [Zaire; Congo Belge], Haut Uélé, Yebo (Moto); Hebauer 1996: 11 [listed]; Hansen 1999b: 164 [catalog]; Hebauer 2006a: 26 [checklist].

Distribution: Afrotropical: Democratic Republic of the Congo [Zaire].

Remarks: Based on the general description and the male genitalia drawing presented by d’Orchymont (1943a: 11), this species likely belongs in *Agraphydrus*.

***Helochares alcinous* Balfour-Browne, 1948**

*Helochares (Hydrobaticus) alcinöus* Balfour-Browne, 1948: 831 – Kenya, Mombasa; Hebauer 1996: 11 [listed]; Hansen 1999b: 164 [catalog]; Hebauer 2006a: 26 [checklist].

Distribution: Afrotropical: Kenya, Tanzania.

***Helochares altus* d’Orchymont, 1943**

Helochares (Hydrobaticus) altus d’Orchymont, 1943f: 5 – India, Tamil Nadu, Nilgiri, southern border of Lake Oatacamund; Hansen 1999b: 164 [catalog].

Distribution: Indo-Malayan: India (Tamil Nadu).

***Helochares anchoralis* Sharp, 1890**

*Helochares anchoralis* Sharp, 1890: 352 – Sri Lanka [Ceylon], Colombo; Gentili et al. 1995: 211 [checklist].

Helochares (Grapidelochares) anchoralis Sharp, 1890; Zaitzev 1908: 381 [catalog].

Helochares (Hydrobaticus) anchoralis Sharp, 1890; d’Orchymont 1923a: 9 [faunistic treatment]; d’Orchymont 1928: 105 [faunistic treatment]; d’Orchymont 1943a: 6 [faunistic treatment]; Hebauer 1995b: 4 [faunistic treatment]; Hansen 1999b: 164 [catalog]; Hebauer 2002a: 23 [new record]; Hebauer and Ryndevich 2005: 45 [new record]; Minoshima and Hayashi 2011: 61 [description of larva]; Dong and Bian 2021: 167 [checklist].

Helochares (Hydrovaticus) anchoralis Sharp, 1890; Matsui 1995: 320 [new record; misspelled subgenus name; year in error].

Distribution: Indo-Malayan: Bangladesh, Cambodia, China (Fujian, Guangdong, Hainan, Jiangxi, Taiwan, Yunnan), India, Indonesia (Sumatra), Laos, Philippines, Sri Lanka, Thailand, Vietnam. Palearctic: China (Hubei, Sichuan), Japan.

**Helochares anchoralis ssp. expansus Knisch, 1921**

Helochares (Hydrobaticus) crenatus ssp. expansus Knisch, 1921: 67 – New Guinea.

Helochares (Hydrobaticus) anchoralis ssp. expansus Knisch, 1921; d’Orchymont 1943a: 6 [taxonomic treatment]; Hansen 1999b: 164 [catalog]; Hansen 2004: 52 [checklist]; Fikáček et al. 2015: 62 [catalog].

Helochares (Hydrobaticus) anchoralis Sharp, 1890; Watts 1995: 119 [faunistic treatment].

Distribution: Australasian: Papua New Guinea.

***Helochares ancoroides* Hebauer, 2001**

Helochares (Hydrobaticus) ancoroidesHebauer 2001a: 13 – Indonesia, Papua, [W. Neuguinea], Paniai Province, Wanggar-Kali Bumi, IR 14; Short and Hebauer 2006: 335 [catalog].

Distribution: Indo-Malayan: Indonesia (Papua).

***Helochares andreinii* d’Orchymont, 1939**

Helochares (Hydrobaticus) andreinii d’Orchymont, 1939f: 320 – Eritrea, Sabarguma; Balfour-Browne 1951: 212 [new records]; Hebauer 1997: 263 [new record]; Hansen 1999b: 165 [catalog]; Hansen 2004: 52 [checklist]; Fikáček et al. 2015: 62 [catalog].

Helochares (Hydrobaticus) andreini d’Orchymont, 1939; Hebauer 1996: 11 [listed; misspelled]; Hebauer 2006a: 26 [checklist; new record; misspelled].

Distribution: Afrotropical: Eritrea, Oman, Saudi Arabia, Yemen, Zimbabwe.

***Helochares androgynus* Hebauer, 1996**

Helochares (Hydrobaticus) androgynus Hebauer, 1996: 11 – Tanzania [“Tanganyika”], 2 mi to Lake Manyara, SE shore; Hansen 1999b: 165 [catalog]; Hebauer 2006a: 26 [new records].

Distribution: Afrotropical: Republic of South Africa, Tanzania, Zambia.

***Helochares anthonyae* Watts, 1995**

Helochares (Hydrobaticus) anthonyae Watts, 1995: 120 – Papua New Guinea, Morobe District, 11 km Lae-Bulolo Rd.; Hansen 1999b: 165 [catalog].

Distribution: Australasian: Australia (Northern Territory), Papua New Guinea.

***Helochares balfourbrownei* Hansen, 1999**

Helochares (Hydrobaticus) balfourbrownei Hansen, 1999b: 165 [nomen novum]; Hebauer 2006a: 26 [checklist].

Helochares (Hydrobaticus) rusticus Balfour-Browne, 1952a: 132 – Ivory Coast, River Lerabara; (primary homonym of *Helochares rusticus* d’Orchymont, 1939 – currently in *Crephelochares*); Balfour-Browne 1959: 311 [faunistic treatment]; Hebauer 1996: 21 [new records].

Distribution: Afrotropical: Benin, Burkina Faso, Ghana, Guinea, Ivory Coast, Liberia, Nigeria, Senegal, Sierra Leone.

***Helochares basilewskyi* Balfour-Browne, 1957**

Helochares (Hydrobaticus) basilewskyi Balfour-Browne, 1957: 23 – Rwanda, Rutovu, forêt du Rugege; Hebauer 1996: 12 [faunistic treatment]; Hansen 1999b: 165 [catalog]; Hebauer 2006a: 26 [checklist].

Distribution: Afrotropical: Rwanda.

***Helochares bilardoi* Hebauer, 2009**

Helochares (Hydrobaticus) bilardoi Hebauer, 2009: 4 – Gabon, Monts de Cristal National Park, Andok Village, Foula; Short and Fikáček 2011: 90 [catalog].

Distribution: Afrotropical: Gabon.

***Helochares blaesus* d’Orchymont, 1936**

Helochares (Hydrobaticus) blaesus d’Orchymont, 1936b: 111 (112) – Botswana [Kalahari], Tsotsoroga Pan; Hebauer 1995a: 262 [faunistic treatment]; Hebauer 1996: 12 [faunistic treatment]; Hansen 1999b: 165 [catalog]; Hebauer 2005: 39 [checklist], 2006a: 26 [checklist].

Distribution: Afrotropical: Botswana [Kalahari], Democratic Republic of the Congo, Ethiopia, Kenya, Malawi, Mozambique, Namibia, Republic of South Africa.

***Helochares bohemani* d’Orchymont, 1936**

Helochares (Hydrobaticus) bohemani d’Orchymont, 1936b: 111 – Namibia [“South-West Africa”], Eenfelsbach 25 km SSE Okahandja; Hebauer 1995a: 262 [faunistic treatment]; Hebauer 1996: 12 [faunistic treatment; new records]; Hansen 1999b: 165 [catalog]; Hebauer 2006a: 26 [checklist].

Distribution: Afrotropical: Angola, Botswana, Cameroon, Ethiopia, Kenya, Madagascar, Namibia, Republic of South Africa, Zambia, Zimbabwe.

***Helochares camerunensis* d’Orchymont, 1939**

Helochares (Hydrobaticus) camerunensis d’Orchymont, 1939b: 303 – Cameroon, Douala [Duala]; Balfour-Browne 1952a: 130 [faunistic treatment]; Hebauer 1996: 13 [faunistic treatment]; Hansen 1999b: 165 [catalog]; Hebauer 2006a: 26 [checklist].

Distribution: Afrotropical: Benin, Cameroon, Democratic Republic of the Congo, Gabon, Gambia, Ghana, Guinea, Ivory Coast, Nigeria, Republic of the Congo, Senegal.

***Helochares cancellatus* Hebauer, 1998**

Helochares (Hydrobaticus) cancellatus Hebauer, 1998: 42 – Sri Lanka [Ceylon], Labugama, 24 mi ESE of Colombo; Hansen 1999b: 165 [catalog].

Distribution: Indo-Malayan: Sri Lanka.

***Helochares championi* Sharp, 1882**

Helochares (Hydrobaticus) championi Sharp, 1882: 75 – Guatemala (Guatemala City, Dueñas, San Géronimo) and Nicaragua (Chontales); Balfour-Browne, 1939: 293 [faunistic treatment]; Hansen 1999b: 165; Short 2005: 217 [faunistic treatment]; Short and Girón 2018: 34 [new record; faunistic treatment].

Distribution: Neotropical: Costa Rica, Guatemala, Nicaragua.

***Helochares chappuisi* Balfour-Browne, 1952**

Helochares (Hydrobaticus) chappuisi Balfour-Browne, 1952a: 132; Hansen 1999b: 165 [catalog].

Helochares (Hydrobaticus) chappiusi Balfour-Browne, 1952; Hebauer 1996: 13 [listed; misspelled]; Hebauer 2006a: 26 [listed; misspelled].

Distribution: Afrotropical: Benin, Mali, Niger.

***Helochares clypeatus* (Blackburn, 1891)**

*Hydrobaticus clypeatus* Blackburn, 1891: 305 – Australia, Northern Territory, Burrundie.

Helochares (Hydrobaticus) clypeatus (Blackburn, 1891); Knisch 1924a: 193 [catalog]; d’Orchymont 1943a: 4 [faunistic treatment]; Watts 1995: 120 [redescription]; Hansen 1999b: 165 [catalog]; Watts 2002: 120 [description of larva with *Helochares tristis* MacLeay].

Distribution: Australasian: Australia (New South Wales, Northern Territory, Queensland, Western Australia).

***Helochares collarti* d’Orchymont, 1939**

Helochares (Hydrobaticus) collarti d’Orchymont, 1939b: 315 – Democratic Republic of the Congo [Congo Belge; Zaire], Blukwa; Balfour-Browne 1950b: 56 [faunistic treatment]; Hebauer 1996: 13 [new record]; Hansen 1999: 165 [catalog]; Hebauer 2006a: 26 [checklist].

Distribution: Afrotropical: Democratic Republic of the Congo, Rwanda.

***Helochares compactus* Hebauer, 2001**

Helochares (Hydrobaticus) compactusHebauer 2001a: 13 – Indonesia, Papua [Irian Jaya], Paniai Province, Nabire – Kali Bobo; Short and Hebauer 2006: 336 [catalog].

Distribution: Indo-Malayan: Indonesia (Papua).

***Helochares conformis* Hebauer, 1995**

Helochares (Hydrobaticus) conformis Hebauer, 1995a: 263 – Namibia, East Caprivi, Katima Mulilo, 17°29'S, 24°17'E; Hebauer 1996: 13 [faunistic treatment]; Hansen 1999b: [catalog]; Hebauer 2006a: 26 [new records].

Distribution: Afrotropical: Namibia, Republic of South Africa, Zambia, Zimbabwe.

***Helochares congoensis* d’Orchymont, 1939**

Helochares (Hydrobaticus) congoensis d’Orchymont, 1939b: 304 – Democratic Republic of the Congo [Congo Belge; Zaire], Boma; Hebauer 1996: 13 [faunistic treatment]; Hansen 1999b: 165 [catalog]; Hebauer 2006a: 26 [checklist].

Distribution: Afrotropical: Democratic Republic of the Congo.

***Helochares congruens* d’Orchymont, 1939**

Helochares (Hydrobaticus) congruens d’Orchymont, 1939b: 304 – Senegal, Thiès; Hebauer 1988: 156 [faunistic treatment]; Hebauer 1996: 13 [faunistic treatment]; Hansen 1999b: 166 [catalog]; Hebauer 2005: 39 [checklist]; Hebauer 2006a: 26 [checklist].

Distribution: Afrotropical: Democratic Republic of the Congo, Ghana, Kenya [in doubt], Madagascar, Malawi, Namibia, Senegal, Republic of South Africa, Tanzania, Uganda, Zambia [in doubt], Zimbabwe.

***Helochares conjectus* d’Orchymont, 1939**

Helochares (Hydrobaticus) conjectus d’Orchymont, 1939b: 305 – Tanzania, Lake Victoria, Ukerewe I.; Balfour-Browne 1950a: 394 [faunistic treatment]; Hebauer 1996: 13 [faunistic treatment]; Hebauer 1996: 14 [faunistic treatment]; Hansen 1999b: 166 [catalog]; Hebauer 2006a: 26 [checklist].

Distribution: Afrotropical: Ethiopia, Tanzania, Zambia, Zimbabwe.

***Helochares crenatostriatus* Régimbart, 1903**

Helochares (Graphelochares) melanophthalmus var. crenatostriatus Régimbart, 1903a: 28 – Madagascar; Seychelles (Aldabra).

Helochares (Hydrobaticus) crenatostriatus Régimbart, 1903; d’Orchymont, 1939e: 298; Hebauer 1996: 14 [faunistic treatment]; Hebauer 1996: 14 [faunistic treatment]; Hansen 1999b: 166 [catalog]; Hebauer 2006a: 26 [checklist].

Distribution: Afrotropical: Cameroon, Gabon, Ghana, Kenya [in doubt], Madagascar, Republic of the Congo, Seychelles (Aldabra).

***Helochares crenatuloides* d’Orchymont, 1943**

Helochares (Hydrobaticus) crenatuloides d’Orchymont, 1943e: 2 – India, “Bengal, Tetara”; Hebauer 1997: 263; Hansen 1999b: 166 [catalog]; Hansen 2004: 52 [checklist]; Fikáček et al. 2010: 151 [new record]; Fikáček et al. 2015: 62 [catalog]; Ribera et al. 2019: 264 [faunistic treatment].

Distribution: Afrotropical: Oman, United Arab Emirates. Indo-Malayan: India (“Bengal”, Madhya Pradesh, Uttar Pradesh).

***Helochares crenatus* Régimbart, 1903**

Helochares (Graphelochares) crenatus Régimbart, 1903b: 54 – India, Tamil Nadu, Pondicherry; d’Orchymont 1940: 168 [lectotype designation].

Helochares (Hydrobaticus) crenatus Régimbart, 1903; d’Orchymont, 1923a: 9 [faunistic treatment]; d’Orchymont 1928: 105 [faunistic treatment]; Hebauer, 1995b: 4 [faunistic treatment]; Hansen 1999b: 166 [catalog]; Hansen 2004: 52 [checklist]; Fikáček et al. 2015: 62 [catalog]; Dong and Bian 2021: 167 [checklist].

*Helochares crenatus* Régimbart, 1903; Gentili et al. 1995: 211 [checklist].

Distribution: Indo-Malayan: China (Yunnan), India (Tamil Nadu, West Bengal), Thailand.

***Helochares crepitus* Balfour-Browne, 1950**

Helochares (Hydrobaticus) crepitus Balfour-Browne, 1950a: 395 – Zambia [“Northern Rhodesia”], “Mwengwa”; Balfour-Browne 1950a: 395 [faunistic treatment]; Hebauer 1996: 14 [faunistic treatment]; Hansen 1999b: 166 [catalog]; Hebauer 2006a: 26 [checklist].

Distribution: Afrotropical: Ghana, Tanzania, Zambia.

***Helochares cresphontes* d’Orchymont, 1939**

Helochares (Hydrobaticus) cresphontes d’Orchymont, 1939b: 313 – Uganda, Kampala; Balfour-Browne 1957: 23 [faunistic treatment]; Hebauer 1996: 14 [faunistic treatment]; Hansen 1999b: 166 [catalog]; Hebauer 2006a: 26 [checklist].

Distribution: Afrotropical: Ghana, Rwanda, Tanzania, Uganda.

***Helochares crespulus* d’Orchymont, 1939**

Helochares (Hydrobaticus) crespulus d’Orchymont, 1939b: 313 – Zaire [“Congo Belge”], Haut Uélé, Watsa; Hebauer 1996: 14 [listed]; Hansen 1999b: 166 [catalog]; Hebauer 2006a: 26 [checklist].

Distribution: Afrotropical: Democratic Republic of the Congo, Gabon.

***Helochares crispus* d’Orchymont, 1939**

Helochares (Hydrobaticus) crispus d’Orchymont, 1939b: 311 – “Zanguebar”; Hebauer 1996: 14 [faunistic treatment]; Hansen 1999b: 166 [catalog]; Hebauer 2005: 39 [new record]; Hebauer 2006a: 26 [checklist].

Distribution: Afrotropical: Ethiopia, Kenya, Malawi, Namibia, Republic of South Africa, Rwanda, Tanzania, Zimbabwe.

***Helochares dalhuntyi* Watts, 1995**

Helochares (Hydrobaticus) dalhuntyi Watts, 1995: 121 – Australia, Queensland, Dalhunty River.

Helochares (Hydrobaticus) anthonyae Watts, 1995; Hansen 1999b: 166 [synonym in error].

Distribution: Australasian: Australia (Northern Territory, Queensland).

***Helochares densepunctus* Régimbart, 1907**

*Helochares densepunctus* Régimbart, 1907: 48 – Guinea Bissau [Guinée Portugaise] (Bolama); Madagascar (Helodrano Antongila [Baie d’Antongil]; “Pays Androy”.

Helochares (Hydrobaticus) densepunctatus Régimbart, 1907; Knisch 1924: 193 [catalog; misspelled]; Hebauer 1996: 14 [faunistic treatment; misspelled].

Helochares (Hydrobaticus) densepunctus Régimbart, 1907; Hansen 1999: 166 [catalog]; Hebauer 2006a: 26 [checklist].

Distribution: Afrotropical: Cameroon, Gabon, Gambia, Guinea, Guinea Bissau, Ivory Coast, Kenya, Liberia, Madagascar, Senegal, Tanzania, Zambia.

***Helochares densus* Sharp, 1890**

*Helochares densus* Sharp, 1890: 352 – Sri Lanka [Ceylon]: Kandy; Dikoya; Bogawantalawa; d’Orchymont 1943e: 7 [specific rank confirmed: not synonym of *lentus* Sharp, as in Zaitzev 1908: 381 (as synonym dubious) and d’Orchymont 1913a: 5].

Helochares (Hydrobaticus) densus Sharp, 1890; d’Orchymont 1923a: 9 [faunistic treatment]; d’Orchymont 1943e: 7 [faunistic treatment]; Hebauer 1995b: 4 [faunistic treatment]; Hansen 1999b: 166 [catalog]; Hebauer 2002a: 23 [new record]; Hansen 2004: 52 [checklist]; Fikáček et al. 2015: 62 [catalog]; Dong and Bian 2021: 167 [checklist].

Distribution: Indo-Malayan: China (Fujian, Guangdong, Guangxi, Hainan, Hunan, Jiangxi, Yunnan, Zhejiang), India (Andaman Is., “Bengal”, Madhya Pradesh, Nicobar Is., Tamil Nadu, Uttarakhand, Uttar Pradesh), Nepal, Thailand, Vietnam. Palearctic: China (Sichuan).

***Helochares dentalus* d’Orchymont, 1943**

Helochares (Hydrobaticus) dentalus d’Orchymont, 1943e: 8 – Malaysia, Sabah [“Borneo septentrional”], Bettotan nr Sandakan; Hansen 1999b: 166 [catalog].

Distribution: Indo-Malayan: Malaysia (Sabah).

***Helochares denudatus* d’Orchymont, 1943**

Helochares (Hydrobaticus) denudatus d’Orchymont, 1943e: 9 – Indonesia, Sumatra, Bedagei NE of Tebingtinggi; Hansen 1999b: 166 [catalog].

Distribution: Indo-Malayan: Indonesia (Sumatra), Malaysia (Peninsula).

***Helochares depactus* d’Orchymont, 1939**

Helochares (Hydrobaticus) depactus d’Orchymont, 1939b: 302 – Kenya, Aberdare Ra. (eastside), Kigangop; Hebauer 1996: 15 [faunistic treatment]; Hansen 1999b: 167 [catalog]; Hebauer 2006a: 26 [checklist].

Distribution: Afrotropical: Kenya.

***Helochares diductus* d’Orchymont, 1939**

Helochares (Hydrobaticus) diductus d’Orchymont, 1939b: 318 – Gabon, Cape Lopez; Hebauer 1996: 15 [faunistic treatment]; Hansen 1999b: 167 [catalog]; Hebauer 2006a: 26 [checklist].

Distribution: Afrotropical: Gabon.

Remarks: Based on original description, probably *Agraphydrus*: small size, pronotal punctures of two different sizes; aedeagus with median lobe spatulate, arched on the sides and truncated in a straight line at apex.

***Helochares didymoides* Balfour-Browne, 1947**

Helochares (Hydrobaticus) didymoides Balfour-Browne, 1947: 141 – Sudan, Didinga Hills, Nagishot; Hebauer 1996: 15 [faunistic treatment]; Hansen 1999b: 167; Hebauer 2006a: 26 [checklist].

Distribution: Afrotropical: Cameroon, Gabon, Sudan.

***Helochares didymus* d’Orchymont, 1939**

Helochares (Hydrobaticus) didymus d’Orchymont, 1939b: 318 – Uganda, Kampala; Hebauer 1996: 15 [faunistic treatment]; Hansen 1999b: 167 [catalog]; Hebauer 2006a: 26 [checklist].

Distribution: Afrotropical: Cameroon, Democratic Republic of the Congo, Gabon, Ghana, Guinea, Kenya, Republic of the Congo, Uganda.

***Helochares difficilis* d’Orchymont, 1939**

Helochares (Hydrobaticus) difficilis d’Orchymont, 1939b: 314 – Uganda (central), “rivière Kizoungou”; Hebauer 1996: 15 [faunistic treatment]; Hansen 1999b: 167 [catalog]; Hebauer 2006a: 26 [checklist].

Distribution: Afrotropical: Democratic Republic of the Congo [Zaire], Kenya, Sudan, Tanzania, Uganda, Zambia.

***Helochares dilutus* (Erichson, 1843)**

*Hydrobius dilutus* Erichson, 1843: 228 – Angola, Benguela; d’Orchymont, 1943c: 1 [specific rank confirmed: not synonym of *Helochares lividus* Forster, as in Bedel 1881a: 330).

*Philhydrus dilutus* (Erichson, 1843); Gemminger and Harold 1868: 481 [catalog].

*Helochares dilutus* (Erichson), 1843; Reiche and Saulcy 1856: 358 [faunistic treatment]; Heyden 1891: 67 [catalog]; Bird et al. 2017 [faunistic treatment].

Helochares (s. str.) dilutus (Erichson, 1843); d’Orchymont, 1943c: 1 [taxonomic treatment]; Balfour-Browne 1950a: 393 [faunistic treatment]; Balfour-Browne 1950b: 59 [faunistic treatment]; Balfour-Browne 1957: 21 [faunistic treatment]; Hebauer 1988: 156 [faunistic treatment]; Hebauer 1995a: 264 [faunistic treatment]; Hebauer 1996: 5 [faunistic treatment]; Hansen 1999b: 160 [catalog]; Hebauer 2005: 39 [new record]; Hebauer 2006a: 25 [checklist]; Fikáček et al. 2015: 61 [catalog; new record].

*Helochares niloticus* Sharp, 1903: 7 – Sudan, Jebel Ahmed Agha [Gebel Ahmed Agha]; d’Orchymont, 1943c: 1 [synonymy].

Distribution: Afrotropical: Angola, Botswana, Cameroon, Democratic Republic of the Congo, Ethiopia, Gambia, Ghana, Ivory Coast, Kenya, Liberia, Madagascar, Malawi, Mauritius (incl. Rodrigues), Mozambique, Namibia, Republic of the Congo, Réunion, Rwanda, Senegal, Republic of South Africa, Sudan, Tanzania, Uganda, Yemen (Socotra), Zambia, Zimbabwe.

**Helochares dilutus ssp. consputus Boheman, 1851**

*Hydrobius consputus* Boheman, 1851: 598 – Republic of South Africa [Caffraria], Orange river reg. [regione fluvii Gariepis]; Hebauer 1988: 156 [as synonym of *dilutus* Erichson]; Hebauer 1996: 5 [as synonym of *dilutus* Erichson].

*Helochares consputus* (Boheman, 1851); Bedel 1880: CXLVIII [new combination].

Enochrus (Lumetus) consputus (Boheman, 1851); Knisch 1924: 208 [catalog].

Helochares (s. str.) dilutusssp. consputus (Boheman, 1851); d’Orchymont 1943c: 6 [taxonomic treatment]; Hansen 1999b: 160 [catalog]; Salah and Régil Cueto 2017: 269 [excluded from Egypt checklist].

*Helochares variabilis* Régimbart, 1903a: 25 – Madagascar, pays Androy, Fort-Dauphin, bassin du Mandraré, Centre-Sud, forêts de la côte Est, Tananarive, baie d’Antongil; Mascarene Is., Réunion (Salazie); d’Orchymont, 1926b: 232 [synonymy].

Distribution: Afrotropical: Madagascar, Mauritius (Mascarene Is.), Namibia, Republic of South Africa.

***Helochares dimorphus* d’Orchymont, 1939**

Helochares (Hydrobaticus) dimorphus d’Orchymont, 1939b: 322 – Democratic Republic of the Congo [Congo Belge; Zaire], Lower Uele, Buta; Balfour-Browne 1950b: 57 [faunistic treatment]; Hebauer 1996: 15 [faunistic treatment]; Hansen 1999b: 167 [catalog]; Hebauer 2006a: 26 [checklist; new records].

Distribution: Afrotropical: Cameroon [in doubt]; Democratic Republic of the Congo, Ghana, Guinea, Kenya, Liberia, Nigeria, Republic of the Congo, Uganda.

***Helochares dollmani* Balfour-Browne, 1950**

Helochares (s. str.) dollmani Balfour-Browne, 1950a: 393 – Zambia [Northern Rhodesia], Namwala, Kafue River; Hebauer 1995a: 265 [faunistic treatment]; Hebauer 1996: 6 [faunistic treatment]; Hansen 1999b: 160 [catalog]; Hebauer 2005: 39 [checklist; new record]; Hebauer 2006a: 26 [checklist; new record].

Distribution: Afrotropical: Madagascar, Malawi, Namibia, Zambia, Zimbabwe.

***Helochares dolus* d’Orchymont, 1939**

Helochares (Hydrobaticus) dolus d’Orchymont, 1939b: 319 – Mali [Haut Sénégal; Senegal], Khayes; Balfour-Browne 1952a: 130 [faunistic treatment]; Hebauer 1996: 15 [faunistic treatment]; Hansen 1999b: 167 [catalog]; Hebauer 2006a: 26 [checklist].

Distribution: Afrotropical: Benin, Cameroon, Democratic Republic of the Congo [Zaire], Gambia, Ghana, Ivory Coast, Mali, Nigeria, Republic of the Congo [Congo-Brazzaville], Senegal, Sierra Leone, Sudan, Tanzania.

***Helochares egregius* Balfour-Browne, 1952**

Helochares (Hydrobaticus) egregius Balfour-Browne, 1952a: 131 – Ivory Coast, Toumodi; Hebauer 1995a: 264 [faunistic treatment]; Hebauer 1996: 16 [new records]; Hansen 1999b: 167 [catalog]; Hebauer 2006a: 26 [checklist; new record].

Distribution: Afrotropical: Benin, Democratic Republic of the Congo, Ghana, Ivory Coast, Namibia, Nigeria, Republic of the Congo, Senegal.

***Helochares endroedyi* Hebauer, 1996**

Helochares (Hydrobaticus) endroedyi Hebauer, 1996: 16 – Ghana, Ashanti Region, Bobiri forest res., 6°40'N, 1°15'W; Hansen 1999b: 167 [catalog]; Hebauer 2006a: 26 [checklist]; Short and Hebauer 2006: 336 [catalog].

Distribution: Afrotropical: Democratic Republic of the Congo, Ghana, Guinea, Zambia.

***Helochares fratris* Hebauer, 2003**

Helochares (Hydrobaticus) fratris Hebauer, 2003b: 68 – SW Madagascar, Morondave district, Miandrivazo, 246 km W of Antsirabe; Hebauer 2006a: 26 [checklist]; Short and Hebauer 2006: 336 [catalog].

Distribution: Afrotropical: Madagascar.

***Helochares fulgurans* Hebauer, 1995**

Helochares (s. str.) fulgurans Hebauer, 1995b: 7 – Thailand, Chantaburi Khao Sabap NP; Hansen 1999b: 160 [catalog].

Distribution: Indo-Malayan: Thailand.

Remarks: Described from a single female specimen, as similar (related) to *Helochares fuliginosus* and *Agraphydrus*.

***Helochares fuliginosus* d’Orchymont, 1932**

Helochares (s. str.) fuliginosus d’Orchymont, 1932: 689 – Indonesia, West Java, Bogor [“Buitenzorg”]; Hebauer 1995b: 7 [faunistic treatment]; Hansen 1999b 160 [catalog]; Jia and Tang 2018: 6 [redescription; new records].

Distribution: Indo-Malayan: China (Fujian, Guangdong, Guangxi, Hong Kong, Macao), Indonesia (Java, Sumatra), Laos, Malaysia (Peninsula).

***Helochares goticus* Hebauer, 1996**

Helochares (Hydrobaticus) goticus Hebauer, 1996: 16 – Democratic Republic of the Congo [Congo-Brazzaville], Kindamba, Meya settlement; Hansen 1999b: 167 [catalog]; Hebauer 2006a: 26 [checklist].

Distribution: Afrotropical: Democratic Republic of the Congo.

***Helochares hainanensis* Dong & Bian, 2021**

Helochares (Hydrobaticus) hainanensis Dong & Bian, 2021: 168 – China: Hainan Province, Qionghai City, Wanquan Town, 19°11'N, 110°23'E.

*Helochares hainanensis* Dong & Bian, 2021.

Distribution: Indo-Malayan: China (Hainan).

***Helochares hiekei* Hebauer, 1995**

Helochares (Hydrobaticus) hiekei Hebauer, 1995b: 5 – India, Karnataka, Ablathi; Hansen 1999b: 167 [catalog].

Distribution: Indo-Malayan: India (Karnataka).

***Helochares insolitus* d’Orchymont, 1925**

Helochares (s. str.) pallens-insolitus d’Orchymont, 1925b: 202 (and 1926a: 380) – Philippines, Manila; Short and Hebauer 2006: 336 [catalog].

Helochares (s. str.) insolitus d’Orchymont, 1925; Hebauer 2002b: 15 [elevated to species; not subspecies of *Helochares pallens* (MacLeay), as in Hansen 1999b: 163]; Hebauer and Ryndevich 2005: 45 [new record].

Distribution: Indo-Malayan: Philippines (Manila), Vietnam.

***Helochares interjectus* Hebauer, 1998**

Helochares (Hydrobaticus) interjectus Hebauer, 1998: 42 – Madagascar, Morarano, “Chrome-Ambakireni”, 10 km W Maheriara; Hansen 1999b: 167 [catalog]; Hebauer 2006a: 26 [checklist].

Distribution: Afrotropical: Madagascar.

***Helochares iteratus* Hebauer, 1996**

Helochares (Hydrobaticus) iteratus Hebauer, 1996: 17 – Republic of the Congo, “Uamgebiet Bosum”; Hansen 1999b: 167 [catalog]; Hebauer 2006a: 26 [checklist].

Distribution: Afrotropical: Democratic Republic of the Congo [in doubt], Republic of the Congo, Tanzania [in doubt].

***Helochares itylus* Balfour-Browne, 1952**

Helochares (Hydrobaticus) itylus Balfour-Browne, 1952a: 131 – Benin [“Dahomey”], Ketou forest; Hebauer 1996: 17 [faunistic treatment]; Hansen 1999b: 167 [catalog]; Hebauer 2006a: 26 [checklist].

Distribution: Afrotropical: Benin, Cameroon, Democratic Republic of the Congo [in doubt], Gambia, Ghana, Ivory Coast, Republic of the Congo [Congo-Brazzaville], Senegal.

***Helochares ivani* Hebauer, 1996**

Helochares (Hydrobaticus) ivani Hebauer, 1996: 18 – Ghana, Kumasi; Hansen 1999b: 167; Hebauer 2006a: 26 [checklist].

Distribution: Afrotropical: Benin, Cameroon, Ghana, Ivory Coast, Liberia, Nigeria, Republic of the Congo [Congo-Brazzaville], Zambia [in doubt].

***Helochares kerstinneumanni* Hebauer, 2009**

Helochares (Hydrobaticus) kerstinneumanni Hebauer, 2009: 4 – Gabon, Makokou-Riv. Ivindo Chutes Kongou; Short and Fikáček 2011: 91 [catalog].

Distribution: Afrotropical: Gabon.

***Helochares knischi* d’Orchymont, 1939**

Helochares (Hydrobaticus) knischi d’Orchymont, 1939b: 320 – Democratic Republic of the Congo [Belg. Congo; Zaire]; Hebauer 1996: 18 [faunistic treatment]; Hansen 1999b: 167; Hebauer 2006a: 26 [checklist].

Distribution: Afrotropical: Democratic Republic of the Congo.

***Helochares laevis* Short & Girón, 2018**

Helochares (Hydrobaticus) laevis Short & Girón, 2018: 36 – Mexico, Chiapas, San Cristobal de las Casas.

Distribution: Neotropical: Mexico.

***Helochares lamprus* d’Orchymont, 1940**

Helochares (Hydrobaticus) lamprus d’Orchymont, 1940: 169 – Indonesia, [Sumatra], Lampong, “Wai Lima”; Hansen 1999b: 167 [catalog].

Distribution: Indo-Malayan: Indonesia (Sumatra).

Remarks: Described as similar to *Helochares nebridus* and/or *H. crenatus*; the aedeagal form as illustrated by d’Orchymont (1940: 170, fig. 8) is rather unusual among *Helochares*.

***Helochares lentus* Sharp, 1890**

*Helochares lentus* Sharp, 1890: 352 – Sri Lanka [Ceylon], Dikoya; Gentili et al. 1995: 211 [checklist].

Helochares (Grapidelochares) lentus Sharp, 1890; Zaitzev 1908: 381 [catalog].

Helochares (Hydrobaticus) lentus Sharp, 1890; d’Orchymont 1923a: 9 [faunistic treatment]; d’Orchymont 1928: 105 [faunistic treatment]; d’Orchymont, 1943e: 3 [taxonomic treatment]; Hebauer 1995b: 5 [faunistic treatment]; Hansen 1999b: 168 [catalog]; Hebauer 2002a: 23 [faunistic treatment]; Hansen 2004: 52 [checklist]; Hebauer and Ryndevich 2005: 45 [new record]; Fikáček et al. 2015: 62 [catalog]; Dong and Bian 2021: 167 [checklist].

Distribution: Indo-Malayan: Bangladesh, Cambodia, China (Fujian, Guangdong, Guangxi, Guizhou, Hunan, Jiangxi, Taiwan, Yunnan), India, Indonesia (Borneo, Java, Lombok, Sumatra), Laos, Malaysia (Peninsula), Nepal, Sri Lanka, Thailand, Vietnam. Palearctic: China (Sichuan, Tibet).

***Helochares lepidus* d’Orchymont, 1943**

Helochares (Hydrobaticus) lentus lepidus d’Orchymont, 1943e: 5 – Philippines, Luzon, Montalban.

Helochares (Hydrobaticus) lepidus d’Orchymont, 1943; Hebauer 1995b: 4 [elevated to species; not subspecies of *lentus* as in d’Orchymont, 1943e]; Hansen 1999b: 168 [catalog].

Distribution: Indo-Malayan: Philippines.

***Helochares leptinus* d’Orchymont, 1943**

Helochares (Hydrobaticus) lentus ssp. leptinus d’Orchymont, 1943e: 5 – Philippines, Luzon, Balbalan.

Helochares (Hydrobaticus) leptinus d’Orchymont, 1943; Hebauer 1995b: 5 [specific rank confirmed; not subspecies of *lentus* as in d’Orchymont, 1943e]; Hansen 1999b: 168 [catalog]; Hebauer 2002a: 23 [new record].

Distribution: Indo-Malayan: Bangladesh, Nepal, Philippines.

***Helochares letus* d’Orchymont, 1943**

Helochares (Hydrobaticus) lentus ssp. letus d’Orchymont, 1943e: 6. – Philippines.

Helochares (Hydrobaticus) letus d’Orchymont, 1943; Hebauer, 1995b: 4 [elevated to species; not subspecies of *lentus* as in d’Orchymont, 1943e]; Hansen 1999: 168 [catalog].

Distribution: Indo-Malayan: Philippines.

***Helochares livianus* d’Orchymont, 1939**

Helochares (Hydrobaticus) livianus d’Orchymont, 1939b: 317 – Uganda, Kampala, Hoima Rd.; Balfour-Browne, 1950b: [faunistic treatment]; Balfour-Browne 1957: 22 [faunistic treatment]; Hebauer 1996: 18 [faunistic treatment]; Hansen 1999b 168 [catalog]; Hebauer 2006a: 26 [checklist].

Distribution: Afrotropical: Democratic Republic of the Congo, Rwanda, Tanzania, Uganda.

***Helochares lividoides* Hansen & Hebauer, 1988**

Helochares (s. str.) lividoides Hansen & Hebauer, 1988: 27 – Israel, Golan, Ein Sha’abanyia; Hebauer 1994: 112 [faunistic treatment]; Hansen 1999b: 160 [catalog]; Hansen 2004: 52 [catalog]; Hebauer and Ryndevich 2005: 45 [new record]; Mart et al. 2010: 298 [faunistic treatment]; Darilmaz and İncekara 2011: 710 [checklist]; Fikáček et al. 2015: 61 [catalog].

Distribution: Palearctic: Israel, Turkey.

***Helochares lividus* (Forster, 1771)**

*Dytiscus lividus* Forster, 1771: 52 (Official Specific Name No. 1992, cf. ICZN, 1964: 242); England and Germany [Anglia; Gallia].

*Hydrophilus lividus* (Forster, 1771); Olivier 1792: 127 [faunistic treatment].

*Philydrus lividus* (Forster, 1771); Solier 1834: 316 [taxonomic treatment].

*Helophilus lividus* (Forster, 1771); Mulsant 1844b: 134 [faunistic treatment].

*Helocharis lividus* (Forster, 1771); Thomson 1859: 18 [faunistic treatment; misspelled].

*Helophygas lividus* (Forster, 1771); Motschulsky 1853: 11 [faunistic treatment].

*Philhydrus lividus* (Forster, 1771); Fairmaire and Laboulbène 1854: 230 [faunistic treatment].

*Hydrophilus fulvus* Fourcroy, 1785: 66 – France, Paris [Parisiensis]; Hansen 1982: 203 [synonymy; not synonym of *obscurus* Müller, as in d’Orchymont 1936a: 10].

*Hydrophilus griseus* Fabricius, 1787: 188 – Germany, Sachsen [Saxonia]; Illiger 1798: 246 [synonym]; Hansen 1982: 203 [not synonym of *obscurus* Müller, as in d’Orchymont, 1933: 304).

*Dytiscus griseus* (Fabricius, 1787); de Villers 1789: 342 [faunistic treatment].

*Philydrus griseus* (Fabricius, 1787); Solier 1834: 316 [faunistic treatment].

*Philhydrus griseus* (Fabricius, 1787); Brullé 1835: 278 [faunistic treatment].

*Hydrobius griseus* (Fabricius, 1787); Erichson 1837: 211 [faunistic treatment].

*Phylidrus griseus* (Fabricius, 1787); Castelnau 1840: 52 [faunistic treatment].

*Pylophilus griseus* (Fabricius, 1787); Motschulsky 1845: 32 [faunistic treatment].

Helochares (s. str.) griseus (Fabricius, 1787); Ganglbauer 1904: 249 [faunistic treatment].

*Helochares griseus* (Fabricius, 1787); Panzera 1932: 54 [description of larvae].

*Hydrophilus pallidus* Rossi, 1792: 66 – NW Italy [Etruria]; Bedel 1881a: 330 [synonymy]; Hansen 1982: 203 [synonym of *griseus* Fabricius: Paykull 1798: 183].

Helophilus lividus var. pallidus (Rossi, 1792); Mulsant 1844a: 135 [faunistic treatment].

Philhydrus lividus var. pallidus (Rossi, 1792); Gemminger and Harold 1868a: 481 [catalog].

Helochares dilutus var. pallidus (Rossi, 1792); Rey 1885b: 287 [faunistic treatment].

? *Hydrophilus chrysomelinus* Herbst, 1797: 313 (primary homonym of *Hydrophilus chrysomelinus* Müller, 1776); Germany; Schönherr, 1808: 7 [synonymy; sub nom. *griseus*); Knisch, 1924: 197 [as synonym dubious of *Helochares griseus*].

*Hydrophilus lividus* Herbst, 1797: 316 (secondary homonym of *Dytiscus lividus* Forster, 1771). – Germany; Schönherr, 1808: 7 [synonymy; sub nom. *griseus*].

*Hydrophilus bicolor*; Paykull, 1798: 184 [misinterpretation of *Hydrophilus bicolor* Fabricius, 1792); Bedel 1878a: CLXXVII [synonymy].

*Helochares ludovici* Schaufuss, 1869: 11 – Spain, Ibiza [Ibiza, Llano de Villa]; Heyden 1891: 67 [catalog]; Ganglbauer 1904: 249 [synonymy]; Hansen 1982: 203 [taxonomic treatment].

Helochares lividus var. pallide-testaceus Stierlin, 1900: 219 [ascribed to Heer, who merely used “pallide” and “testaceus” as the first two adjectives in a description of an unnamed variety [Heer 1841: 485]] – Switzerland [Helvetiae]; Knisch 1924: 198 [synonymy].

Helochares (s. str.) lividus (Forster, 1771); Hansen 1982: 203 [taxonomic treatment]; Hebauer 1994: 111 [faunistic treatment; identification doubtful]; Hebauer 1996: 7 [faunistic treatment]; Ribera et al. 1996: 10 [checklist]; Hansen 1999b: 161 [catalog]; Hansen 2004: 52 [catalog]; Hebauer and Ryndevich 2005: 45 [new record]; Darilmaz and Kiyak 2006: 79 [new record]; Hebauer 2006a: 25 [checklist]; Mart et al. 2010: 298 [faunistic treatment]; Darilmaz and İncekara 2011: 710 [checklist]; Fikáček et al. 2015: 61 [catalog]; Salah and Régil Cueto 2017: 269 [record from Egypt in doubt]; Gentili et al. 2018: 23 [faunistic treatment]; Benamar et al. 2021: 34 [checklist].

*Helochares lividus* (Forster, 1771); Reiche 1854: 9 [catalog]; Heyden 1891: 67 [catalog]; d’Orchymont 1913b: 200 [description of larva]; Panzera 1932: 60 [description of larvae]; Mabrouki et al. 2018 [faunistic treatment]; Angus et al. 2020: 21 [karyotype].

Distribution: Palearctic: Algeria, Austria, Belarus, Bosnia Herzegovina, Bulgaria, Canary Islands, Croatia, Czech Republic, Egypt [in doubt], France, Germany, Great Britain, Greece, Hungary, Iran, Italy, Luxembourg, Macedonia, Morocco, Netherlands, Poland, Portugal, Serbia and Montenegro, Slovakia, Slovenia, Spain, Switzerland, Syria, Tunisia, Turkey, Ukraine.

***Helochares lobatus* d’Orchymont, 1948**

Helochares (s. str.) lobatus d’Orchymont, 1948: 730 – Ethiopia, Abyssinian Highlands, Muger Wenz, “Mulu”; Hebauer 1996: 7 [faunistic treatment]; Hansen 1999b: 161 [catalog]; Hebauer 2006a: 25 [checklist].

Distribution: Afrotropical: Ethiopia.

Remarks: This species was described as similar to *Helochares lividus*, but the aedeagus is remarkably different; it needs to be studied in detail, as the drawing provided by d’Orchymont (1948: fig. 5A) is not entirely clear and does not allow to establish affinities with other *Helochares* groups.

***Helochares lollius* d’Orchymont, 1939**

Helochares (Hydrobaticus) lollius d’Orchymont, 1939b: 321 – Uganda, Kampala; Hebauer 1996: 18 [faunistic treatment]; Hansen 1999b: 168 [catalog]; Hebauer 2006a: 26 [checklist].

Distribution: Afrotropical: Gabon, Uganda.

***Helochares loticus* Hebauer, 1998**

Helochares (Hydrobaticus) loticus Hebauer, 1998: 43 – Thailand (north), Lom Sak, 40 km N Phetchabun; Hansen 1999b: 168 [catalog].

Distribution: Indo-Malayan: Thailand.

***Helochares loweryae* Watts, 1995**

Helochares (Hydrobaticus) loweryae Watts, 1995: 122 – Papua New Guinea, Mt. Lamington; Hansen 1999b: 168 [catalog].

Distribution: Australasian: Australia (Northern Territory), Papua New Guinea.

***Helochares luridus* (MacLeay, 1871)**

*Hydrobaticus luridus* MacLeay, 1871: 131 – Australia, Queensland, Gayndah.

Hydrobaticus tristis var. luridus MacLeay, 1871; Blackburn, 1893: 99 [faunistic treatment].

Helochares (Hydrobaticus) luridus (MacLeay, 1871); Watts, 1995: 122 [valid species, not synonym of *Helochares tristis* MacLeay, 1871, as in Zaitzev 1908: 390); Hansen 1999b: 168 [catalog]; Watts 2002: 120 [description of larva with *Helochares tristis* MacLeay].

Distribution: Australasian: Australia (New South Wales, Northern Territory, Queensland, Western Australia).

***Helochares lutulentus* Balfour-Browne, 1952**

Helochares (Hydrobaticus) lutulentus Balfour-Browne, 1952b: 516 – Mauritania, Kédia d’Idjil; Hebauer 1996: 18 [faunistic treatment]; Hansen 1999b: 168 [catalog]; Hebauer 2006a: 26 [checklist].

Distribution: Afrotropical: Benin, Mauritania, Morocco [in doubt].

***Helochares maculatus* Hebauer, 1988**

Helochares (Helocharimorphus) maculatus Hebauer, 1988: 157 – Namibia, Okavango, Nyangana; Hebauer 1995a: 265 [faunistic treatment]; Hebauer 1996: 9 [faunistic treatment]; Hansen 1999b: 164 [catalog]; Hebauer 2006a: 27 [checklist].

Distribution: Afrotropical: Namibia.

***Helochares maculicollis* Mulsant, 1844**

*Helochares maculicollis* Mulsant, 1844b: 379 – U.S.A., Louisiana [Louisiane]; Richmond 1920: 62 [description of immature stages]; Archangelsky 1997: 49 [redescription of immature stages].

*Philhydrus maculicollis* (Mulsant, 1844); Lacordaire, 1854: 457 [faunistic treatment].

Philhydrus (s. str.) maculicollis (Mulsant, 1844); LeConte 1855: 370 [faunistic treatment].

Helochares (Grapidelochares) maculicollis Mulsant, 1844; Zaitzev 1908: 381 [catalog].

Helochares (Hydrobaticus) maculicollis Mulsant, 1844; Hansen 1999b: 168 [catalog]; Short 2005: 218 [faunistic treatment]; Short and Girón 2018: 36 [taxonomic review].

? *Helochares bipunctatus* Sharp, 1882: 76. – Mexico (Cordova) and Guatemala (Torola); d’Orchymont 1943b: 3 [synonymy in doubt].

Helochares (Grapidelochares) bipunctatus Sharp, 1882; Zaitzev, 1908a: 381.

Distribution: Nearctic: U.S.A. (Alabama, Arkansas, Delaware, District of Columbia, Florida, Georgia, Illinois, Indiana, Iowa, Kansas, Kentucky, Louisiana, Maryland, Mississippi, Missouri, North Carolina, Ohio, Oklahoma, Pennsylvania, South Carolina, Tennessee, Texas, Virginia). Neotropical: Guatemala [in doubt], Mexico.

***Helochares madli* Hebauer, 2002**

Helochares (s. str.) madli Hebauer, 2002b: 15 – Madagascar, Mahajanga Katsepi; Hebauer 2006a: 25 [checklist]; Short and Hebauer 2006: 336 [catalog].

Distribution: Afrotropical: Madagascar.

Remarks: This species was described from a single female specimen. According to Hebauer (2002b) it is similar to a small *Helochares dilutus*, but with shorter maxillary palps and different elytral punctation. Given that the male of this species remains unknown, the placement of this species in *Helochares* needs to be confirmed.

***Helochares marreensis* Watts, 1995**

Helochares (Hydrobaticus) marreensis Watts, 1995: 123 – Australia, Northern Territory, 7 km NW by N of Cahills Crossing, East Alligator River, 12°23'S, 132°56'E; Hansen 1999b: 168 [catalog].

Distribution: Australasian: Australia (New South Wales, Northern Territory, Queensland, South Australia, Victoria, Western Australia).

***Helochares mecarus* d’Orchymont, 1939**

Helochares (Hydrobaticus) mecarus d’Orchymont, 1939b: 310 – Ethiopia, Arussi Galla, A. Ganale Gudda; Hebauer 1996: 19 [faunistic treatment]; Hansen 1999b: 169 [catalog]; Hebauer 2006a: 26 [checklist].

Distribution: Afrotropical: Benin, Botswana, Ethiopia, Kenya, Namibia, Zambia.

***Helochares mediastinus* d’Orchymont, 1939**

Helochares (Hydrobaticus) mediastinus d’Orchymont, 1939b: 311 – Ethiopia, Arussi Galla, A. Ganale Gudda; Hebauer 1996: 19 [faunistic treatment]; Hansen 1999b: 169 [catalog]; Hebauer 2006a: 26 [checklist].

Distribution: Afrotropical: Angola, Benin, Ethiopia, Kenya, Madagascar, Namibia, Tanzania.

***Helochares melanophthalmus* (Mulsant, 1844)**

*Helophilus melanophthalmus* Mulsant, 1844a: 137 (ascribed to Dufour) – Sudan [in doubt: type locality probably Sudan (d’Orchymont 1936a), not Spain [Espagne] as stated in the original description].

*Hydrobius melanophthalmus* (ascribed to Dufour); Dejean 1833: 134 [nomen nudum].

*Helochares melanophthalmus* (Mulsant, 1844); Rey 1885b: 288 [specific rank confirmed; not synonym of *dilutus* Erichson, 1843, as in Reiche and Saulcy 1856: 358].

Helochares (Graphelochares) melanophthalmus (Mulsant, 1844); Kuwert 1890: 39 [catalog]; Heyden 1891: 67 [catalog].

Helochares (Hydrobaticus) melanophthalmus (Mulsant, 1844); Hebauer 1996: 19 [faunistic treatment]; Hansen 1999b: 169 [catalog]; Hebauer 2006a: 26 [checklist]; Salah and Régil Cueto 2017: 270 [excluded from Egypt].

Distribution: Afrotropical: Cameroon, Ethiopia, Ghana, Ivory Coast, Nigeria, Senegal, Seychelles, Sudan.

***Helochares mendosus* Hebauer, 1996**

Helochares (Hydrobaticus) mendosus Hebauer, 1996: 19 – Ghana, Ashanti region, Bobiri forest reserve 6°40'N, 1°15'W; Hansen 1999b: 19 [catalog]; Hebauer 2006a: 26 [checklist].

Distribution: Afrotropical: Ghana.

***Helochares mentinotus* Kuwert, 1888**

*Helochares mentinotus* Kuwert, 1888: 292 – Egypt [Aegyptus].

Helochares (Crephelochares) mentinotus Kuwert, 1888; Kuwert 1890a: 38 [faunistic treatment].

Helochares (Chasmogenus) mentinotus Kuwert, 1888; Knisch 1824a: 195 [checklist].

Helochares (Hydrobaticus) mentinotus Kuwert, 1888; d’Orchymont 1936d: 6 [taxonomic treatment]; Balfour-Browne, 1950b: 57 [faunistic treatment]; Hebauer 1994: 112 [faunistic treatment]; Hebauer 1996: 20 [faunistic treatment]; Hansen 1999b: 169 [catalog]; Hansen 2004: 52 [catalog]; Hebauer 2006a: 26 [checklist]; Fikáček et al. 2015: 62 [catalog]; Salah and Régil Cueto 2017: [faunistic treatment].

*Helochares squalidus* Sharp, 1903: 7 – South Sudan (White Nile River; Jebel Ahmed Agha; north of Jebel Ahmed Agha; north of Kaka; d’Orchymont 1936d: 6 [synonymy].

Helochares (Grapidelochares) squalidus Sharp, 1903; Zaitzev 1908: 381 [checklist].

Distribution: Afrotropical: Chad, Democratic Republic of the Congo [Zaire; DR Congo], Ethiopia [Abyssinia], Kenya, South Sudan, Uganda. Palearctic: Egypt, Israel.

***Helochares menulus* d’Orchymont, 1943**

Helochares (Hydrobaticus) menulus d’Orchymont, 1943a: 10 – Democratic Republic of the Congo [Congo Belge; Zaire], Nizi-Blukwa; Hebauer 1996: 20 [faunistic treatment]; Hansen 1999b: 169 [catalog]; Hebauer 2006a: 26

Distribution: Afrotropical: Democratic Republic of the Congo [Zaire; DR Congo], Kenya, Nigeria, Tanzania.

***Helochares meracus* Balfour-Browne, 1950**

Helochares (Hydrobaticus) meracus Balfour-Browne, 1950a: 395 – Zambia [Northern Rhodesia], Nama-ula; Hebauer 1996: 20 [faunistic treatment]; Hansen 1999b: 169 [catalog]; Hebauer 2005: 39 [checklist]; Hebauer 2006a: 26 [checklist].

Distribution: Afrotropical: Ethiopia, Malawi, Republic of South Africa [in doubt], Zambia.

***Helochares mersus* d’Orchymont, 1939**

Helochares (Hydrobaticus) mersus d’Orchymont, 1939b: 307 – Ethiopia [Abyssinie]; Balfour-Browne 1950b: 56 [faunistic treatment]; Hebauer 1988: 156 [faunistic treatment]; Hebauer 1995a: 264 [faunistic treatment]; Hebauer 1996: 20 [faunistic treatment]; Hansen 1999b: 169 [catalog]; Hebauer 2005: 39 [checklist]; Hebauer 2006a: 26 [checklist].

Distribution: Afrotropical: Botswana [in doubt; “Kalahari”], Democratic Republic of the Congo [Zaire; DR Congo], Ethiopia, Kenya, Malawi, Namibia, Rwanda, Tanzania, Uganda, Zimbabwe.

***Helochares minax* d’Orchymont, 1939**

Helochares (Hydrobaticus) minax d’Orchymont, 1939b: 316 – Uganda, Kampala; Balfour-Browne 1950b: 57 [faunistic treatment]; Hebauer 1996: 20 [faunistic treatment]; Hansen 1999b: 169 [catalog]; Hebauer 2006a: 26 [checklist].

Distribution: Afrotropical: Rwanda, Uganda, Gabon [in doubt], Kenya, Tanzania.

***Helochares minor* d’Orchymont, 1925**

Helochares (Hydrobaticus) minor d’Orchymont, 1925c: 293 – Vietnam [Indo-Chine], Cha Pa; d’Orchymont 1928: 106 [faunistic treatment]; d’Orchymont 1943e: 9 [faunistic treatment]; Hansen 1999b: 189 [catalog]; Dong and Bian 2021: 167 [new record].

Distribution: Indo-Malayan: China (Hainan), India (Bihar), Vietnam.

***Helochares minusculus* d’Orchymont, 1943**

Helochares (Hydrobaticus) minusculus d’Orchymont, 1943e: 10 – Indonesia, North Sumatra, Danau Toba region, nr Huta Gindjang; Hansen 1999b: 169 [catalog]; Hebauer and Ryndevich 2005: 46 [new record].

Distribution: Indo-Malayan: Burma, Indonesia (Sumatra), Laos.

***Helochares namcatensis* Hebauer, 2002**

Helochares (Hydrobaticus) namcatensisHebauer 2002b: 12 – Vietnam, Nam Cat Tien National Park; Hebauer 2002b: 12 [faunistic treatment]; Short and Hebauer 2006: 336 [catalog].

Distribution: Indo-Malayan: Vietnam.

***Helochares nebridius* d’Orchymont, 1940**

Helochares (Hydrobaticus) nebridius d’Orchymont, 1940: 169 – Indonesia, Sumatra, Palembang; Hebauer 1995b: 5 [faunistic treatment]; Hansen 1999b: 169 [catalog].

Distribution: Indo-Malayan: Indonesia (Java, Lombok, Sumatra), Singapore.

***Helochares negatus* Hebauer, 1995**

Helochares (Hydrobaticus) negatus Hebauer, 1995b: 5 – Bangladesh, Dinajpur; Hansen 1999b: 169 [catalog]; Hebauer 2002a: 24 [new record]; Hansen 2004: 52 [catalog]; Hebauer and Ryndevich 2005: 46 [new record]; Fikáček et al. 2015: 62 [catalog].

Distribution: Indo-Malayan: Bangladesh, India (Tamil Nadu), Nepal.

***Helochares neglectus* (Hope, 1845)**

*Hydrobius neglectus* Hope, 1845: 16 – China, Guangdong, Guangzhou, Canton; Gentili et al. 1995: 211 [catalog].

Helochares (Hydrobaticus) neglectus (Hope, 1845); d’Orchymont 1919c: 150 [new combination in doubt]; d’Orchymont 1940b: 166 [new combination confirmed]; Hebauer 1995b: 6 [faunistic treatment]; Hansen 1999b: 169 [catalog]; Hansen 2004: 52 [catalog]; Fikáček et al. 2015: 62 [catalog]; Dong and Bian 2021: 167 [checklist].

Distribution: Indo-Malayan: Cambodia, China (Fujian, Guangdong, Guangxi, Hainan, Jiangxi, Yunnan, Zhejiang), Malaysia (Peninsula), Thailand, Vietnam. Palearctic: China (Hubei, Jiangsu, Shanghai, Sichuan).

***Helochares nexus* Short & Girón, 2018**

Helochares (Hydrobaticus) nexus Short & Girón, 2018: 39 – Panama, Coclé Province, 8°39'05.2"N, 80°35'18.7"W.

Distribution: Neotropical: Ecuador, Panama, Venezuela.

***Helochares nigrifrons* Brancsik, 1893**

Helochares melanophthalmus var. nigrifrons Brancsik, 1893: 219 – Madagascar, Nosy Bé [Nossibé]; Régimbart 1900: 50 [faunistic treatment].

Helochares (Grapidelochares) nigrifrons Brancsik, 1893; Zaitzev 1908: 381 [catalog].

Helochares (Hydrobaticus) melanophthalmus var. nigrifrons Brancsik, 1893; Knisch 1924: 194 [catalog].

Helochares (Hydrocaticus) nigrifrons Brancsik, 1893; d’Orchymont 1939b: 297 [specific rank confirmed; subgeneric name misspelled].

Helochares (Hydrobaticus) nigrifrons Brancsik, 1893; d’Orchymont 1941: 15 [list]; Hebauer 1996: 20 [new records]; Hansen 1999b: 170 [catalog]; Hebauer 2006a: 26 [checklist].

Distribution: Afrotropical: Madagascar, Seychelles (Aldabra), Tanzania.

***Helochares nigripalpis* Hebauer & Hendrich, 1999**

Helochares (Hydrobaticus) nigripalpis Hebauer & Hendrich, 1999: 48 – Australia, Northern Territory, Kakadu National Park, Jim Jim Falls Camp Area, 13°16.218'S, 132°49.276'E; Short and Hebauer 2006: 336 [catalog].

Distribution: Australasian: Australia (Northern Territory).

***Helochares nigritulus* Kuwert, 1889**

*Helochares nigritulus* Kuwert, 1889: 8 [and 1890a: 34] – Italy, Sicily

Helochares (s. str.) nigritulus Kuwert, 1889; Heyden 1891: 67 [catalog]; Hansen 1999b: 162 [catalog]; Hansen 2004: 52 [catalog]; Fikáček et al. 2015: 61 [catalog].

Distribution: Palearctic: Italy.

***Helochares nigroseriatus* Hebauer, 1998**

Helochares (Hydrobaticus) nigroseriatus Hebauer, 1998c: 43 – Zimbabwe, vicinity of Kotwa, “Broken Causeway”, 17°0'S, 32°45'E; Hansen 1999b: 170 [catalog]; Hebauer 2006a: 26 [checklist].

Distribution: Afrotropical: Zambia, Zimbabwe.

Remarks: Hebauer (2002) indicates that the aedeagus of *Helochares nigroseriatus* corresponds to fig. 5 in Hebauer 1998.

***Helochares niobelus* d’Orchymont, 1939**

Helochares (Hydrobaticus) niobelus d’Orchymont, 1939b: 308 – Democratic Republic of the Congo [Congo Belge; Zaire], Haut Uélé, Watsa; Hebauer 1996: 20 [faunistic treatment]; Hansen 1999b: 170 [catalog]; Hebauer 2006a: 26 [checklist].

Distribution: Afrotropical: Cameroon [in doubt], Democratic Republic of the Congo, Republic of South Africa, Uganda.

***Helochares nipponicus* Hebauer, 1995**

*Helochares striatus* Sharp, 1873: 60 [secondary homonym of *Hydrobius striatus* Boheman, 1851: 599]; Hebauer 1995b: 6 [synonymy; not synonym of *Helochares lepidus* d’Orchymont, *Helochares leptinus* d’Orchymont or *Helochares lentus* Sharp, as in d’Orchymont 1943e: 6].

Helochares (Hydrobaticus) nipponicus Hebauer, 1995b: 6 [replacement name for *Helochares striatus* Sharp, 1873]; Hansen 1999b: 170 [catalog]; Hansen 2004: 52 [catalog]; Minoshima and Hayashi 2011: 64 [description of immature stages]; Fikáček et al. 2015: 62 [catalog]; Dong and Bian 2021: 167 [new record].

Distribution: Palearctic: China (Jilin), Japan, South Korea.

***Helochares normatus* (LeConte, 1861)**

*Philhydrus normatus* LeConte, 1861: 341 – U.S.A., California, Bodega.

*Helochares normatus* (LeConte, 1861); Horn 1890: 252 [faunistic treatment].

*Chasmogenus normatus* (LeConte, 1861); Zaitzev 1908: 383 [catalog].

Helochares (Hydrobaticus) normatus (LeConte, 1861); Knisch 1924: 194 [catalog]; Hansen 1999b: 170 [catalog]; Short 2005: 218 [new records]; Short and Girón 2018: 42 [taxonomic treatment].

*Helochares seriatus* Sharp, 1882: 76 – Guatemala (Guatemala City; Pantaleon; Coatepeque; Rio Naranjo; San Gerónimo); d’Orchymont 1943b: 4 [synonymy].

Helochares (Grapidelochares) seriatus Sharp, 1882; Zaitzev 1908: 381 [catalog].

? *Helochares regularis* Sharp, 1882: 76 – Mexico – d’Orchymont 1943d: 4 [synonymy in doubt].

? Helochares (Grapidelochares) regularis Sharp, 1882; Zaitzev 1908a: 381 [catalog].

Distribution: Nearctic: USA (Arizona, California, Nevada, Oregon, Texas). Neotropical: Costa Rica, El Salvador, Guatemala, Honduras, Mexico, Nicaragua.

***Helochares notaticollis* Régimbart, 1906**

Helochares melanophthalmus var. notaticollis Régimbart, 1906: 260 – Kenya, Nairobi.

Helochares (Hydrobaticus) notaticollis Régimbart; Balfour-Browne 1950a: 394 [faunistic treatment]; Balfour-Browne 1950b: 54 [faunistic treatment]; d’Orchymont, 1936b: 111 [specific rank confirmed]; Hebauer 1996: 20 [faunistic treatment]; Hansen 1999b: 170 [catalog]; Hebauer 2005: 39 [checklist]; Hebauer 2006a: 26 [checklist].

Distribution: Afrotropical: Kenya, Malawi, Rwanda, Tanzania, Uganda.

**Helochares notaticollis ssp. curtus Régimbart, 1906**

Helochares melanophthalmus var. curtus Régimbart, 1906: 260 – Kenya, Bura.

Helochares (Hydrobaticus) notaticollis var. curtus Régimbart, 1906; d’Orchymont, 1936a: 111.

Helochares (Hydrobaticus) notaticollis ssp. curtus Régimbart, 1906; Hansen 1999b: 170 [catalog]; Hebauer 2006a: 26 [checklist].

Distribution: Afrotropical: Kenya.

***Helochares obliquus* Mart, İncekara & Karaca, 2010**

*Helochares obliquus* Mart, İncekara & Karaca, 2010: 299 – Turkey, Ordu province, Mesudiye, Lake Ulugöl, 40°24'N, 37°49'E.

Helochares (s. str.) obliquus Mart, İncekara & Karaca, 2010; Darilmaz and İncekara 2011: 711 [checklist]; Short and Fikáček 2011 [catalog]; Fikáček et al. 2015: 61 [catalog].

Distribution: Palearctic: Turkey.

***Helochares obscurus* (Müller, 1776)**

*Hydrophilus obscurus* Müller, 1776: 69 – Denmark and Norway [Dania et Norvegia].

Helochares (s. str.) obscurus (Müller, 1776); d’Orchymont 1933: 306 [specific rank confirmed; not synonym of *Helochares griseus* Fabricius, as in Illiger 1798: 246; not synonym of *Helochares lividus* Forster, as in Mulsant 1844a: 134]; Hebauer 1994: 113 [faunistic treatment]; Hansen 1999b: 162 [catalog]; Hansen 2004: 52 [catalog]; Hebauer and Ryndevich 2005: 45 [new records]; Mart et al. 2010: 299 [faunistic treatment]; Darilmaz and İncekara 2011: 711 [checklist]; Fikáček et al. 2015: 62 [catalog]; Jia and Tang 2018b: 12 [redescription; new record].

*Hydrophilus erythrocephalus* Fabricius, 1792: 185 – No type locality given; Hansen 1982: 207 [synonymy; not synonym of *Helochares griseus* Fabricius, as in Erichson 1837: 211].

Helophilus lividus var. erythrocephalus (Fabricius, 1792); Mulsant 1844a: 135 [faunistic treatment].

Philhydrus lividus var. erythrocephalus (Fabricius, 1792); Gemminger and Harold 1868: 481 [catalog].

Helochares (s. str.) erythrocephalus (Fabricius, 1792); Kuwert 1890a: 37 [taxonomic treatment].

*Helochares erythrocephalus* (Fabricius, 1792); Heyden 1891: 67 [catalog].

*Hydrophilus variegatus* Herbst, 1797: 304 – Germany [... in hiesigen Gewässern (i.e., German waters)]; Hansen 1982: 207 [synonymy; not synonym of *Helochares griseus* Fabricius, as in Illiger 1801a: 60].

Hydrophilus griseus var. variegatus Herbst, 1797; Gyllenhal, 1808: 122 [faunistic treatment].

Philhydrus lividus var. variegatus (Herbst, 1797); Gemminger and Harold 1868: 481 [catalog].

*Hydrobius lividus*; Stephens, 1829: 130 [misinterpretation of *Dytiscus lividus* Forster].

*Philhydrus lividus*; Stephens, 1839: 91 [misinterpretation of *Dytiscus lividus* Forster].

*Helochares subcompressus* Rey, 1885a: 14 – France, Lille; Hansen 1982: 207 [synonymy; (Fauvel, 1895: 92 [synonym of *erythrocephalus* Fabricius]); not synonym of *Helochares griseus* Fabricius, as in Ganglbauer, 1904: 249)]; Heyden 1891: 67 [catalog].

Helochares erythrocephalus var. substriatus Sahlberg, 1903: 20 – Greece, Corfu, Stravopotamos [(Corcyra): prope flumen Stravopotamos]; Hansen 1982: 207 [synonymy].

Helochares (s. str.) griseus (?) var. substriatus Sahlberg, 1903; Zaitzev 1908: 382 [catalog].

*Helochares griseus* a. Mülleri Reitter, 1909a: 364 [infrasubspecific name; unavailable under ICZN Code Art. 1b (5), 45f)]; Hansen 1982: 207 [synonymy].

? *Hydrophilus chrysomelinus*; Panzer, 1795: 72 [misinterpretation of *Dytiscus chrysomelinus* Fabricius]. Hansen, 1982: 202 [synonymy in doubt; not synonym of *griseus* Fabricius, as in Schönherr 1808: 7 – in doubt; not synonym of *Helochares pallidus* Rossi, as in Mulsant 1844a: 135].

? Philhydrus lividus var. chrysomelinus (Panzer, 1795); Gemminger and Harold 1868: 481 [catalog].

*Helochares obscurus* (Müller, 1776); Angus et al. 2020: 21 [karyotype].

Distribution: Palearctic: Austria, Azerbaijan, Belarus, China (Xinjiang), Croatia, Czech Republic, Denmark, Estonia, Finland, France, Germany, Georgia, Great Britain, Greece, Hungary, Iran, Israel, Italy, Latvia, Lithuania, Luxembourg, Montenegro, Netherlands, Norway, Poland, Russia, Slovakia, Sweden, Switzerland, Turkey.

***Helochares opacus* Hebauer, 2009**

Helochares (Hydrobaticus) opacus Hebauer, 2009: 5 – Gabon, Monts de Cristal National Park, Asseng Assala Village; Short and Fikáček 2011: 91 [catalog].

Distribution: Afrotropical: Gabon.

***Helochares pallens* (MacLeay, 1825)**

*Enhydrus pallens* MacLeay, 1825: 35 – Indonesia, Java.

*Philhydrus pallens* (MacLeay, 1825); Gemminger and Harold 1868: 482 [catalog].

Enochrus (Lumetus) pallens (MacLeay, 1825); Zaitzev 1908: 388 [catalog].

*Helochares pallens* (MacLeay, 1825); Gentili et al. 1995: 211 [catalog].

Helochares (s. str.) pallens (MacLeay, 1825); d’Orchymont 1926b: 232 [new combination]; d’Orchymont 1928: 107 [faunistic treatment]; Balfour-Browne 1950b: 59 [faunistic treatment]; Balfour-Browne 1951: 213 [faunistic treatment]; Balfour-Browne 1952a: 129 [faunistic treatment]; Balfour-Browne 1957: 21 [faunistic treatment]; Hebauer 1988: 156 [faunistic treatment]; Hebauer 1994: 113 [faunistic treatment]; Hebauer 1995a: 265 [faunistic treatment]; Hebauer 1995b: 7 [faunistic treatment]; Hebauer 1996: 8 [faunistic treatment]; Hebauer 1997: 263 [faunistic treatment]; Hansen 1999b: 162 [catalog]; Hebauer 2002a: 24 [new record]; Hansen 2004: 52 [catalog]; Hebauer 2005: 39 [checklist]; Hebauer and Ryndevich 2005: 46 [new record]; Hebauer 2006a: 25 [checklist; new records]; Mart et al. 2010: 298 [new record]; Short 2010: 312 [faunistic treatment]; Darilmaz and İncekara 2011: 711 [checklist]; Minoshima and Hayashi 2011: 53 [description of larva]; Fikáček et al. 2015: 62 [catalog]; Jia and Tang 2018: 15 [redescription].

*Helochares parvulus* Reiche and Saulcy [in Reiche 1854: 9 – nomen nudum].

*Helochares parvulus* Reiche & Saulcy, 1856: 359 – Lebanon, Beirut [Beyrouth]; d’Orchymont 1927b: 6 [synonymy]; d’Orchymont 1932: 688 [faunistic treatment].

*Philhydrus parvulus* (Reiche & Saulcy, 1856); Gemminger and Harold 1868: 482 [catalog].

Enochrus (Methydrus) parvulus (Reiche & Saulcy, 1856); Zaitzev 1908: 384 [catalog].

? *Helochares simplex* Wollaston, 1867: 44 [published in synonymy with *dilutus* Erichson; unavailable under ICZN Code Art. 11e]; d’Orchymont 1943e: 8 [synonymy in doubt].

*Helochares lewisius* Sharp, 1873: 60 – Japan (Kyushu (Nagasaki), and Honshu (Hyogo)) [Nagasaki and Hiogo]; Balfour-Browne 1939: 293 [synonymy].

Helochares (s. str.) lewisianus Sharp, 1873; Zaitzev 1908: 382 [catalog; misspelled].

? *Philhydrus parvulus* Guillebeau, 1896: 226 – “Le Cuire” [secondary homonym of *Helochares parvulus* Reiche & Saulcy, 1856; possibly synonym of the same, as in Knisch 1924: 219]; Handen 1999b: 162 [synonymy confirmed].

*Helochares dispar* Sharp, 1903: 7 – Sudan (White Nile River; Jebel Ahmed Agha; north of Jebel Ahmed Agha; north of Kaka); d’Orchymont 1926b: 232 [synonymy].

*Helochares laeviusculus* Régimbart, 1906: 261 – Kenya, Lake Victoria, Winam Gulf [Baie de Kavirondo]; Hebauer 1996: 8 [synonymy].

Helochares (s. str.) pallensssp. laeviusculus Régimbart, 1906 – Democratic Republic of the Congo, Ishango, Semliki River; Balfour-Browne 1950b: 60 [new combination]; Hebauer 2006a: 25 [checklist].

Distribution: Afrotropical: Benin, Botswana, Cameroon, Chad, Democratic Republic of the Congo, Ethiopia, Ghana, Guinea, Ivory Coast, Kenya, Liberia, Madagascar, Namibia, Rwanda, Republic of South Africa, Sudan, Tanzania, Uganda, Yemen, Zambia, Zimbabwe. Indo-Malayan: Bangladesh, Burma, China (Fujian, Guangdong, Guangxi, Guizhou, Hainan, Hong Kong, Hunan, Jiangxi, Macao, Yunnan), India (Assam, Bihar), Indonesia (Java, Sumatra), Laos, Malaysia (Peninsula), Nepal, Philippines, Sri Lanka, Thailand. Palearctic: China (Chongqing, Hubei, Shaanxi, Sichuan, Xizang [Tibet]), Egypt, Israel, Japan, Lebanon, Pakistan, Syria, Turkey. Australasian: Papua New Guinea (New Guinea), Vanuatu.

***Helochares parallelus* Hebauer, 1999**

Helochares (Hydrobaticus) parallelusHebauer 1999: 11 – Botswana, Kasane Chobe Safari Lodge, Chobe Banks; Hebauer 2006a: 26 [checklist]; Short and Hebauer 2006: 336 [catalog].

Distribution: Afrotropical: Botswana, Republic of South Africa.

***Helochares percyi* Watts, 1995**

Helochares (Hydrobaticus) percyi Watts, 1995: 125 – Australia, Queensland (N.), Boar Pocket Road; Hansen 1999b: 170 [catalog].

Distribution: Australasian: Australia (Australian Capital Territory, New South Wales, Northern Territory, Queensland, Western Australia).

***Helochares perminutus* Hebauer, 1996**

Helochares (Hydrobaticus) perminutus Hebauer, 1996: 20 – Nigeria [Nig.], Pandam W.P. River Li; Hebauer 1996: 20 [faunistic treatment]; Hansen 1999b: 170 [catalog]; Hebauer 2006a: 26 [checklist].

Distribution: Afrotropical: Benin, Ghana, Nigeria, Sierra Leone.

***Helochares phallicus* d’Orchymont, 1936**

Helochares (Hydrobaticus) phallicus d’Orchymont, 1936b: 111 – Botswana, Makgadikgadi [Makarikari], Nkate; Hebauer 1995a: 264 [faunistic treatment]; Hebauer 1996: 21 [faunistic treatment]; Hansen 1999b: 170 [checklist]; Hebauer 2005: 39 [checklist]; Hebauer 2006a: 26 [checklist].

Distribution: Afrotropical: Angola, Botswana, Malawi, Namibia, Republic of South Africa, Zambia, Zimbabwe.

***Helochares politus* Short & Girón, 2018**

Helochares (Hydrobaticus) politus Short & Girón, 2018: 45 – Guatemala, Departamento de Huehuetenango, 11 km N. Santa Eulalia on road to San Mateo Ixtatán.

Distribution: Neotropical: Guatemala.

***Helochares punctatus* Sharp, 1869**

*Helochares punctatus* Sharp, 1869: 241 – England (Whittlesea, Mere, Cambridge, London and the New Forest); Hansen 1982: 206 [specific rank confirmed; not synonym of *erythrocephalus* Fabricius, as in Heyden 1891: 67; not synonym of *griseus* Fabricius, as in Ganglbauer 1904: 249]; Angus et al. 2020: 21 [karyotype].

*Helochares punctulatus* Sharp, 1869 [misspelling]; Bedel 1881a: 312 [catalog]; Heyden 1891: 67 [catalog].

Helochares (s. str.) punctatus Sharp, 1869; Hansen 1999b: 163 [catalog]; Hansen 2004: 52 [catalog]; Hebauer and Ryndevich 2005: 45 [new records]; Darilmaz and İncekara 2011: 711 [checklist]; Fikáček et al. 2015: 62 [catalog]; Benamar et al. 2021: 35 [checklist].

Distribution: Palearctic: Belarus, Denmark, France, Germany, Great Britain, Hungary, Ireland, Lithuania, Luxembourg, Netherlands, Portugal, Russia, Spain, Turkey, Ukraine.

***Helochares rugipennis* Balfour-Browne, 1958**

Helochares (Hydrobaticus) rugipennis Balfour-Browne, 1958a: 183 – Mali [“French Sudan”], Source Sanga; Hebauer 1996: 21 [faunistic treatment]; Hansen 1999b: 171 [catalog]; Hebauer 2006a: 26 [checklist].

Distribution: Afrotropical: Guinea, Ivory Coast, Mali, Nigeria, Sierra Leone.

***Helochares salvazai* d’Orchymont, 1919**

Helochares (Hydrobaticus) salvazai d’Orchymont, 1919a: 76 (and 1921: 11) – Cambodia; d’Orchymont 1928: 106 [faunistic treatment]; d’Orchymont 1943e: 10 [faunistic treatment]; Hansen 1999b: 171 [catalog].

Distribution: Indo-Malayan: Cambodia.

***Helochares sauteri* d’Orchymont, 1943**

Helochares (Hydrobaticus) sauteri d’Orchymont, 1943e: 6 – Taiwan [Formose], “Kosempo”; Hansen 1999b: 171 [catalog]; Hansen 2004: 52 [catalog]; Fikáček et al. 2015: 62 [catalog]; Dong and Bian 2021: 167 [checklist].

*Helochares sauteri* d’Orchymont; Gentili et al. 1995 [catalog]; Angus et al. 2020: 21 [karyotype].

Distribution: Indo-Malayan: China (Guangdong, Guizhou, Jiangxi, Taiwan, Zhejiang). Palearctic: China (Hubei, Sichuan).

***Helochares schoedli* Hebauer, 1996**

Helochares (Hydrobaticus) schoedli Hebauer, 1996: 22 – Democratic Republic of the Congo [Zaire; Haut-Zaire], Dungu; Hansen 1999b: 171 [catalog]; Hebauer 2006a: 26 [checklist].

Distribution: Afrotropical: Democratic Republic of the Congo.

***Helochares schwendingeri* Hebauer, 1995**

Helochares (Hydrobaticus) schwendingeri Hebauer, 1995b: 7 – Thailand, Chiang Mai; Hansen 1999b: 171 [catalog]; Hebauer and Ryndevich 2005: 46 [new record].

Helochares (Hydrobaticus) ubudensis Hebauer, 1998: 44 – Indonesia, Bali, Ubud; Hansen 1999b: 171; Hebauer 2002b: 13 [synonymy]; Short and Hebauer 2006: 337 [catalog].

Distribution: Indo-Malayan: Indonesia (Bali), Laos, Malaysia (Peninsula), Thailand, Vietnam.

Remarks: Hebauer (2002b) indicates that the aedeagus of *Helochares schwendingeri* (as *Helochares ubudensis*) corresponds to fig. 4 in Hebauer 1998.

***Helochares scitulus* Balfour-Browne, 1952**

Helochares (Hydrobaticus) scitulus Balfour-Browne, 1952a: 130 – Benin [Dahomey], Bassila; Hebauer 1996: 22 [faunistic treatment]; Hansen 1999b: 171 [catalog]; Hebauer 2006a: 26 [checklist].

Distribution: Afrotropical: Benin, Gambia, Ghana, Mali, Senegal, Sudan.

***Helochares sechellensis* Régimbart, 1903**

Helochares (Graphelochares) melanophthalmus var. sechellensis Régimbart, 1903a: 27 – Seychelles [Iles Séchelles].

Helochares (Hydrobaticus) sechellensis Régimbart, 1903; d’Orchymont 1939b: 297 [specific rank confirmed]; Hansen 1999b: 171 [catalog]; Hebauer 2006a: 26 [checklist].

Distribution: Afrotropical: Seychelles.

***Helochares serpentinus* Hebauer, 1998**

Helochares (Hydrobaticus) serpentinus Hebauer, 1998: 44 – Republic of South Africa, Wilderness National Park, Lang Wie, 33°59'0"S, 22°40'6"E); Hansen 1999b: 171 [catalog]; Hebauer 2006a: 26 [checklist].

Distribution: Afrotropical: Republic of South Africa.

***Helochares sharpi* (Kuwert, 1890)**

*Helocharimorphus sharpi* Kuwert, 1890a: 63 (and 1890b: 306) – Egypt [Aegypten]; Syria, Lebanon or Israel [Syria]; Iraq [Mesopotamien].

Helochares (Helocharimorphus) sharpi (Kuwert, 1890); Knisch 1924: 195 [catalog]; Hebauer 1994: 113 [faunistic treatment]; Hansen and Hebauer 1988: 29 [in key]; Hansen 1999b: 164 [catalog]; Hansen 2004: 52 [catalog]; Hebauer 2006a: 27 [checklist]; Fikáček et al. 2015: 62 [catalog]; (İncekara et al. 2016: 22 [new record]; Salah and Régil Cueto 2017: 265 [faunistic treatment].

Distribution: Afrotropical: Ghana, Madagascar, Tanzania, Togo, Uganda, Zambia. Palearctic: Egypt, Iraq, Israel, Turkey.

***Helochares silvester* Hebauer, 2009**

Helochares (Hydrobaticus) silvester Hebauer, 2009: 5 – Republic of the Congo, Brazzaville, d’Odzala Mboko National Park; Short and Fikáček 2011: 91 [catalog].

Distribution: Afrotropical: Republic of the Congo.

***Helochares simulator* Knisch, 1922**

Helochares (Hydrobaticus) simulator Knisch, 1922: 104 – Papua New Guinea, Bismarck Archipelago, Duke of York [not “Duke of York” (= Atafu) in Polynesia]; d’Orchymont 1943a: 7 [faunistic treatment]; Hansen 1999b: 171 [catalog]; Short 2010: 313 [faunistic treatment].

Distribution: Australasian: Fiji, Papua New Guinea (Duke of York). Oceanian: Samoa, Tonga.

***Helochares skalei* Hebauer, 2002**

Helochares (Hydrobaticus) skalei Hebauer, 2002b: 13 – South Africa, Mpumalanga White River, White River behind Staudamm, Quelle; Hebauer 2005: 39 [checklist]; Hebauer 2006a: 27 [checklist]; Short and Hebauer 2006: 336 [catalog].

Distribution: Afrotropical: Malawi, Republic of South Africa, Zimbabwe.

***Helochares songi* Jia & Tang, 2018**

Helochares (s. str.) songi Jia & Tang, 2018b: 3 – China, Guangxi Province, Shiwandashan, Nalin River.

Distribution: Indo-Malayan: China (Guangxi).

***Helochares steffani* Hebauer, 2002**

Helochares (Hydrobaticus) steffani Hebauer, 2002b: 13 – Namibia, Ongongo falls, 19°08'S, 13°49'W, ca. 6 km upp. Warmquelle; Hebauer 2006a: 27 [catalog]; Short and Hebauer 2006: 336 [catalog].

Distribution: Afrotropical: Namibia.

***Helochares stenius* d’Orchymont, 1943**

Helochares (Hydrobaticus) stenius d’Orchymont, 1943a: 8 – Democratic Republic of the Congo [Congo Belge; Zaire], Lubutu nr Kisangani [Stanleyville]; Hebauer 1996: 22 [faunistic treatment]; Hansen 1999b: 171 [catalog]; Hebauer 2006a: 27 [checklist].

Distribution: Afrotropical: Democratic Republic of the Congo, Gabon, Republic of the Congo.

***Helochares striatus* (Boheman, 1851)**

*Hydrobius striatus* Boheman, 1851: 599 – Republic of South Africa, Natal [terra Natalensi].

*Helochares striatus* (Boheman, 1851); Bedel 1880: CXLVIII [new combination].

Helochares (Hydrobaticus) striatus (Boheman, 1851); d’Orchymont 1919c: 150 [faunistic treatment]; d’Orchymont 1943e: 6 [faunistic treatment]; Balfour-Browne 1950a: 394 [faunistic treatment]; Hebauer 1996: 22 [faunistic treatment]; Hansen 1999b: 171 [catalog]; Hebauer 2006a: 27 [checklist].

Distribution: Afrotropical: Democratic Republic of the Congo, Gambia, Senegal, Sierra Leone, Republic of South Africa, Uganda.

***Helochares strictus* d’Orchymont, 1939**

Helochares (Hydrobaticus) strictus d’Orchymont, 1939b: 306 – Tanzania, Lake Victoria, Ukerewe I; Balfour-Browne 1950b: 55 [faunistic treatment]; Hebauer 1996: 22 [faunistic treatment]; Hansen 1999b: 171 [catalog]; Hebauer 2006a: 27 [checklist].

Distribution: Afrotropical: Cameroon, Democratic Republic of the Congo, Ghana, Guinea, Kenya, Rwanda, Senegal, Tanzania, Uganda.

***Helochares strigellus* Hebauer, 2002**

Helochares (Hydrobaticus) strigellus Hebauer, 2002b: 14 – Liberia, Saclepea; Hebauer 2006a: 27 [checklist]; Short and Hebauer 2006: 336 [catalog].

Distribution: Afrotropical: Kenya, Liberia.

***Helochares structus* d’Orchymont, 1936**

Helochares (Hydrobaticus) structus d’Orchymont, 1936b: 112 – Botswana, Kasane; Hebauer 1988: 156 [faunistic treatment]; Hebauer 1995a: 264 [faunistic treatment]; Hebauer 1996: 23 [faunistic treatment]; Hansen 1999b: 171 [catalog].

Distribution: Afrotropical: Benin [in doubt], Botswana, Cameroon [in doubt], Congo, Gambia, Ghana [in doubt], Guinea, Ivory Coast, Liberia, Namibia, Republic of South Africa, Sudan, Tanzania, Zambia.

***Helochares sublineatus* Hebauer, 2002**

Helochares (s. str.) sublineatusHebauer 2002b: 15 – Ghana, Tamale; Hebauer 2006a: 25 [checklist]; Short and Hebauer 2006: 337 [catalog].

Distribution: Afrotropical: Ghana, Nigeria.

Remarks: The aedeagus in this species is quite unusual among *Helochares* (Hebauer 2002b: fig. 8).

***Helochares subseriatus* Hebauer, 2009**

Helochares (Hydrobaticus) subseriatus Hebauer, 2009: 5 – Gabon, Bateke Plateau National Park, Camp, Mbie; Short and Fikáček 2011: 91 [catalog].

Distribution: Afrotropical: Gabon.

Remarks: The species is described from a single female specimen.

***Helochares subtilis* d’Orchymont, 1936**

Helochares (Hydrobaticus) subtilis d’Orchymont, 1936b: 112 – ? Botswana [“Kalahari”], “Tsotsoroga Pan”; Hebauer 1995a: 264 [faunistic treatment]; Hebauer 1996: 23 [faunistic treatment]; Hansen 1999b: 171 [catalog]; Hebauer 2006a: 27 [checklist].

Distribution: Afrotropical: Botswana, Cameroon, Democratic Republic of the Congo, Ethiopia, Namibia, Republic of the Congo, Republic of South Africa, Zimbabwe.

***Helochares sufflavus* Balfour-Browne, 1952**

Helochares (Hydrobaticus) sufflavus Balfour-Browne, 1952a: 131 – Togo, Tohoun; Hebauer 1996: 23 [faunistic treatment]; Hansen 1999b: 171 [catalog]; Hebauer 2006a: 27 [checklist].

Distribution: Afrotropical: Togo.

***Helochares sylvaticus* Balfour-Browne, 1957**

Helochares (Hydrobaticus) sylvaticus Balfour-Browne, 1957: 24 – Burundi [“Urundi”], Bururi; Hebauer 1996: 23 [faunistic treatment]; Hansen 1999b: 171 [catalog]; Hebauer 2006a: 27 [checklist].

Distribution: Afrotropical: Burundi, Democratic Republic of the Congo, Republic of the Congo.

***Helochares tamsi* Balfour-Browne, 1947**

Helochares (Hydrobaticus) tamsi Balfour-Browne, 1947: 142 – São Tomé and Príncipe [West Africa], São Tomé; Hebauer 1996: 23 [faunistic treatment]; Hansen 1999b: 171 [catalog]; Hebauer 2006a: 27 [checklist].

Distribution: Afrotropical: Gabon, Kenya [in doubt], Republic of the Congo, São Tomé and Príncipe.

***Helochares tatei* (Blackburn, 1896)**

*Hydrobaticus tatei* Blackburn, 1896: 258 – Australia, Palm Creek; Watts 1995: 126 [Lectotype designated].

Helochares (Hydrobaticus) tatei (Blackburn, 1896); Knisch 1924: 194 [catalog]; d’Orchymont 1943a: 5 [faunistic treatment]; Hansen 1999b: 171 [catalog].

Distribution: Australasian: Australia (New South Wales, Northern Territory, Queensland, South Australia, Western Australia).

***Helochares tengchongensis* Dong & Bian, 2021**

Helochares (Hydrobaticus) tengchongensis Dong & Bian, 2021: 171 – China: Yunnan Province, Tengchong City, Lianghe County, Longhe Village, 1074 m, 24°48'21.158"N, 98°17'51.522"E.

*Helochares tengchongensis* Dong & Bian, 2021.

Distribution: Indo-Malayan: China (Yunnan).

***Helochares tenuistriatus* Régimbart, 1908**

Helochares (Hydrobaticus) tenuistriatus Régimbart, 1908: 315 – Australia, Western Australia, Perth, Lake Monger [“Mongers Lake, N. de Subiaco”]; Knisch 1924: 194 [catalog]; d’Orchymont 1943a: 5 [faunistic treatment]; Watts 1995: 127 [faunistic treatment]; Hansen 1999b: 172 [catalog]; Watts 2002: 120 [description of larva with *Helochares tristis* MacLeay].

Distribution: Australasian: Australia (Western Australia).

***Helochares tertius* Hebauer, 1996**

Helochares (Helocharimorphus) tertius Hebauer, 1996: 9 – Republic of the Congo, Mt. Fouari reservation, near Gabon; Hansen 1999b: 172 [catalog]; Hebauer 2006a: 27 [checklist].

Distribution: Afrotropical: Democratic Republic of the Congo, Republic of the Congo.

Remarks: The species is described from a unique female.

***Helochares thurmerae* Watts, 1995**

Helochares (Hydrobaticus) thurmerae Watts, 1995: 127 – Papua New Guinea, Morobe District, Gusap Markham Valley ca. 90 ml W of Lae; Hansen 1999b: 172 [catalog].

Distribution: Australasian: Papua New Guinea.

***Helochares tristis* (MacLeay, 1871)**

*Hydrobaticus tristis* MacLeay, 1871: 131 – Australia, Queensland, Gayndah; Anderson 1976: 220 [description of immature stages]; Watts 2002: 119 [description of larva].

Helochares (Hydrobaticus) tristis (MacLeay, 1871); Knisch 1924: 194 [checklist]; d’Orchymont 1943a: 2 [faunistic treatment]; Hansen 1999b: 172 [catalog].

*Hydrobaticus australis* Blackburn, 1888: 823 – Australia, South Australia, Port Lincoln; Watts 1995: 128 [lectotype designated; synonymy].

Helochares (Hydrobaticus) australis (Blackburn, 1888); Knisch 1924: 193 [catalog]; d’Orchymont 1943a: 3 [faunistic treatment].

Distribution: Australasian: Australia (Australian Capital Territory, New South Wales, Northern Territory, Queensland, South Australia, Tasmania, Victoria, Western Australia).

***Helochares trujillo* Short & Girón, 2018**

Helochares (Hydrobaticus) trujillo Short & Girón, 2018: 45 – Venezuela, Mérida State, Mérida, Monte Zerpa Area.

Distribution: Neotropical: Venezuela.

***Helochares uenoi* Matsui, 1995**

Helochares (Hydrobaticus) uenoi Matsui, 1995: 317 – Japan, Okinawa Islands, Yonaguni Island, Tindabana; Hansen 1999b: 172 [catalog]; Hansen 2004: 52 [catalog]; Fikáček et al. 2015: 62 [catalog].

Distribution: Palearctic: Japan.

***Helochares uhligi* Hebauer, 1999**

Helochares (s. str.) uhligiHebauer 1999: 11 – Republic of South Africa, Cape Province, Karoo National Park, Mountain View River; Hebauer 2006a: 26 [checklist]; Short and Hebauer 2006: 337 [catalog].

Distribution: Afrotropical: Republic of South Africa.

***Helochares vitalisi* d’Orchymont, 1919**

Helochares (s. str.) vitalisi d’Orchymont, 1919a: 78 (and 1921c: 13) – Cambodia, Phnom Penh; d’Orchymont 1928: 108 [faunistic treatment]; Hansen 1999b: 163 [catalog].

Distribution: Indo-Malayan: Cambodia.

***Helochares wagneri* Hebauer, 2002**

Helochares (Hydrobaticus) wagneri Hebauer, 2002b: 14 – Kenya, Kakamega Forest, 0°22'N, 34°50'E; Hebauer 2006a: 27 [checklist]; Short and Hebauer 2006: 337 [catalog].

Distribution: Afrotropical: Kenya.

***Helochares wattsi* Hebauer & Hendrich, 1999**

Helochares (Hydrobaticus) wattsi Hebauer & Hendrich, 1999: 50 – Australia: Northern Territory, Kakadu National Park, Jim Jim Hwy, Black Jungle Spring; Short and Hebauer 2006: 337 [catalog].

Distribution: Australasian: Australia (Northern Territory).

Remarks: The aedeagus in this species is quite unusual among *Helochares* (Hebauer and Hendrich 1999: fig. 4).

***Helochares wuzhifengensis* Dong & Bian, 2021**

Helochares (Hydrobaticus) wuzhifengensis Dong & Bian, 2021: 170 – China: Jiangxi Province, Ganzhou City, Shangyou County, Wuzhifeng Town, 25°57'N, 114°05'E.

*Helochares wuzhifengensis* Dong & Bian, 2021.

Distribution: Indo-Malayan: China (Jiangxi).

***Helochares yangae* Hebauer, Hendrich & Balke, 1999**

Helochares (Hydrobaticus) yangae Hebauer, Hendrich & Balke, 1999: 340 – Malaysia, Pahang, Lake Cini, lakeside near Rimba Resort; Short and Hebauer 2006: 337 [catalog].

Distribution: Indo-Malayan: Malaysia.

***Helochares zamora* Short & Girón, 2018**

Helochares (Hydrobaticus) zamora Short & Girón, 2018: 46 – Ecuador, Zamora-Chinchipe Province, Zamora.

Distribution: Neotropical: Ecuador.

### *Helopeltarium* d’Orchymont, 1943

***Helopeltarium ferrugineum* d’Orchymont, 1943**

*Helopeltarium ferrugineum* d’Orchymont, 1943f: 10 – Burma, Dawna Range (eastside), “Sukli”.

Distribution: Indo-Malayan: Myanmar [Burma].

### *Katasophistes* Girón & Short, 2018

***Katasophistes charynae* Girón & Short, 2018**

*Katasophistes charynae* Girón & Short, 2018: 136 – Peru, Madre de Dios, Parque Manu, Pakitza, 12°07'S, 70°58'W.

Distribution: Neotropical: Peru.

***Katasophistes cuzco* Girón & Short, 2018**

*Katasophistes cuzco* Girón & Short, 2018: 138 – Peru, Cuzco, Quita Calzón, at km 164, 13°09'S, 71°22'W.

Distribution: Neotropical: Peru.

***Katasophistes merida* Girón & Short, 2018: 138**

*Katasophistes merida* Girón & Short, 2018: 138 – Venezuela, Mérida State, ca. 12 km SE of Santo Domingo, 8°51.933'N, 70°37.131'W.

Distribution: Neotropical: Venezuela.

***Katasophistes superficialis* Girón & Short, 2018**

*Katasophistes superficialis* Girón & Short, 2018 – Ecuador, Pastaza Province: “AGIP platform Villano B, along transect 1 and 2.

Distribution: Neotropical: Ecuador.

### *Nanosaphes* Girón & Short, 2018

***Nanosaphes castaneus* Girón & Short, 2018**

*Nanosaphes castaneus* Girón & Short, 2018: 146 – Brazil, Pará, Rio Xingu Camp, Altamira ca. 60 km S, 3°39'S, 52°22'W.

Distribution: Neotropical: Brazil (Pará).

***Nanosaphes hesperus* Girón & Short, 2018**

*Nanosaphes hesperus* Girón & Short, 2018: 148 – Suriname, Sipaliwini District, Camp 1, on Kutari River, 2°10.521'N, 56°47.244'W.

Distribution: Neotropical: Suriname.

***Nanosaphes punctatus* Girón & Short, 2018**

*Nanosaphes punctatus* Girón & Short, 2018: 151 – Suriname, Sipaliwini District, Brownsberg Nature Park, 04°56.871'N, 55°10.911'W.

Distribution: Neotropical: Suriname.

***Nanosaphes tricolor* Girón & Short, 2018**

*Nanosaphes tricolor* Girón & Short, 2018: 151 – Suriname, Sipaliwini District, Camp 4 (low), Kasikasima, trail to Kasikasima, 2.97731°N, 55.38500°W.

Distribution: Neotropical: Suriname.

### *Novochares* Girón & Short gen. nov.

***Novochares abbreviatus* (Fabricius, 1801) comb. nov.**

*Hydrophilus abbreviatus* Fabricius, 1801: 251 – [America meridionali].

Helochares (s. str.) abbreviatus (Fabricius, 1801); d’Orchymont 1939e: 258 [taxonomic treatment]; d’Orchymont 1943d: 55 [faunistic treatment]; Fernández, 1982a: 34 [taxonomic treatment]; Hansen 1999b: 159 [catalog]; Short 2005: 215 [new record]; Clarkson and Ferreira-Jr. 2014: 400 [faunistic treatment]; Silva et al. 2018: 9 [faunistic treatment].

*Helochares abbreviatus* (Fabricius, 1801); Gonzalez-Rodriguez et al. 2017: 606 [checklist].

*Philydrus pallidus* Castelnau, 1840: 53 – Brazil (secondary homonym of *Hydrophilus pallidus* Rossi, 1792); d’Orchymont 1936a: 10 [synonymy].

*Philhydrus pallidus* Castelnau, 1840; Gemminger and Harold 1868: 482 [checklist].

*Helochares pallidus* (Castelnau, 1840); Fleutiaux and Sallé 1889: 376 [checklist].

Enochrus (Lumetus) pallidus (Castelnau, 1840); Zaitzev 1908: 388 [checklist].

Helochares (Hydrobaticus) rufobrunneus Balfour-Browne, 1939: 293. – Lesser Antilles, Grenada, Balthazar; Spangler 1981b: 158 [synonymy].

Distribution: Neotropical: Argentina, Bolivia, Brazil (Espírito Santo, Pernambuco, Piauí), Colombia, Costa Rica, Cuba, French Guiana, Lesser Antilles, Panama, Paraguay, Suriname, Venezuela.

***Novochares atlanticus* (Clarkson & Ferreira-Jr., 2014) comb. nov.**

Helochares (s. str.) atlanticusClarkson and Ferreira-Jr. 2014a: 401 – Brazil, São Paulo, Ubatuba, Parque Estadual da Serra do Mar, Núcleo Picinguaba.

Distribution: Neotropical: Brazil (Rio de Janeiro, São Paulo).

***Novochares atratus* (Bruch, 1915) comb. nov.**

*Helochares atratus* Bruch, 1915: 451 – Argentina, Buenos Aires province; Fernández 1982a: 35 [taxonomic treatment]; Hansen 1999b: 159 [catalog]; Gonzalez-Rodriguez et al. 2017: 609 [new record].

Helochares (s. str.) atratus Bruch, 1915; Clarkson and Ferreira-Jr. 2014: 400 [faunistic treatment].

Helochares (s. str.) parhedrus d’Orchymont, 1939: 259 – Argentina, Chaco de Santiago del Estero; not synonym of Helochares (Sindolus) gibbus Brullé, 1841 (= *Helochares ventricosus* Bruch), as in d’Orchymont 1926: 236); Fernández 1982a: 35 [synonymy; redescription].

Distribution: Neotropical: Argentina, Brazil (Mato Grosso do Sul, Minas Gerais), Colombia, Ecuador [in doubt]; Paraguay.

***Novochares bolivianus* (Fernández, 1989) comb. nov.**

Helochares (s. str.) bolivianus Fernández, 1989: 146 – Bolivia, Santa Cruz Department, Gutiérrez Province, Nueva Moka; Hansen 1999b: 158 [catalog].

Distribution: Neotropical: Bolivia.

***Novochares carmona* (Short, 2005) comb. nov.**

Helochares (s. str.) carmona Short, 2005: 215 – Costa Rica, Guanacaste Province, Laguna de Cocodrilo, near Carmona, 10°03'31.0"N, 85°14'25.6"W; Short and Hebauer 2006: 335 [catalog].

Distribution: Neotropical: Costa Rica.

***Novochares chaquensis* (Fernández, 1982) comb. nov.**

Helochares (s. str.) chaquensis Fernández, 1982b: 87 – Argentina, Chaco Province, San Bernardo; Hansen 1999b: 159 [catalog]; Clarkson and Ferreira-Jr. 2014: 400 [faunistic treatment].

Distribution: Neotropical: Argentina, Brazil (Mato Grosso do Sul).

***Novochares cochlearis* (Fernández, 1982) comb. nov.**

Helochares (s. str.) cochlearis Fernández, 1982b: 89 – Argentina, Corrientes, Santo Tomé; Hansen 1999b: 159 [catalog].

Distribution: Neotropical: Argentina, Paraguay.

***Novochares coya* (Fernández, 1982) comb. nov.**

Helochares (s. str.) coya Fernández, 1982b: 87 – Bolivia, Santa Cruz Department, Sara Province, Monteros; Hansen 1999b: 160 [catalog].

Distribution: Neotropical: Bolivia.

***Novochares guadelupensis* (d’Orchymont, 1926) comb. nov.**

Helochares (s. str.) guadelupensis d’Orchymont, 1926b: 233 – Lesser Antilles, Guadeloupe; Hansen 1999b: 160 [catalog].

Distribution: Neotropical: Lesser Antilles (Guadeloupe).

***Novochares inornatus* (d’Orchymont, 1926) comb. nov.**

Helochares (s. str.) inornatus d’Orchymont, 1926b: 235 – French Guiana, “Passoura”; Balfour-Browne 1939: 295 [faunistic treatment]; Hansen 1999: 160 [catalog]; Clarkson and Ferreira-Jr. 2014: 400 [faunistic treatment].

Distribution: Neotropical: Brazil (Amazonas, São Paulo), French Guiana, Guyana [British Guiana].

***Novochares oculatus* (Sharp, 1882) comb. nov.**

*Helochares oculatus* Sharp, 1882: 74 – Guatemala, Paso Antonio; Fernández, 1982a: 31 [specific rank confirmed; not synonym of *Helochares pallidus* Castelnau, as in d’Orchymont 1926b: 232; not a synonym of *abbreviatus* Fabricius, as in d’Orchymont 1936a: 10; lectotype designated].

Helochares (s. str.) oculatus Sharp, 1882: 74; Hansen 1999b: 162 [catalog]; Short 2005: 216 [new record]; Clarkson and Ferreira-Jr. 2014: 400 [faunistic treatment].

Distribution: Neotropical: Argentina, Brazil (Mato Grosso do Sul, Pernambuco, Rio de Janeiro), Costa Rica, Guatemala, Panama; according to Hansen (1999b: 162), records from Mexico and the Antilles (Grenada, St. Vincent) need confirmation.

***Novochares pallipes* (Brullé, 1841) comb. nov.**

Hydrophilus (Philydrus) pallipes Brullé, 1841: 58. – Uruguay, Montevideo.

*Philhydrus pallipes* (Brullé, 1841); Lacordaire 1854: 457.

*Helochares pallipes* (Brullé, 1841); Bedel, 1881: XCIV.

Helochares (s. str.) pallipes (Brullé, 1841); Fernández 1983: 444 [redescription; description of immature stages]; Hansen 1999b: 163 [catalog]; Clarkson and Ferreira-Jr. 2014: 400 [faunistic treatment].

Distribution: Neotropical: Argentina, Brazil (Mato Grosso do Sul, Minas Gerais), Paraguay, Uruguay.

***Novochares pichilingue* (Fernández, 1989) comb. nov.**

Helochares (s. str.) pichilingue Fernández, 1989: 147 – Ecuador, Los Ríos, Quevedo, Río Pichilingue; Hansen 1999b: 163 [catalog].

Distribution: Neotropical: Ecuador.

***Novochares sallaei* (Sharp, 1882) comb. nov.**

*Helochares sallæi* Sharp, 1882: 75 – Mexico, Cordova.

Helochares (s. str.) sellae Sharp, 1882; Knisch, 1924a: 199 [catalog; misspelled].

Helochares (s. str.) sallaei Sharp, 1882; Hansen 1999b: 163 [catalog]; Short 2005: 217 [faunistic treatment].

*Philhydrus estriatus* Blatchley, 1917: 139. – U.S.A., Florida (west coast); Winters, 1927a: 24 [synonymy].

Enochrus (Lumetus) estriatus (Blatchley, 1917); Knisch 1924a: 208 [catalog].

Distribution: Nearctic: U.S.A. (Florida). Neotropical: Belize, Costa Rica, Mexico.

NOTE: The occurrence of the species in Florida is thought to have been an introduction (Young 1954). If this is the case, the introduction happened more than 100 years ago, as it has been in Florida since at least 1917 when specimens were described as a new species of *Enochrus* (*E. estriatus* Blatchley, 1917). We reviewed the holotype of *E. estriatus* and confirmed this synonymy.

***Novochares tectiformis* (Fernández, 1982) comb. nov.**

Helochares (s. str.) tectiformis Fernández, 1982b: 88. – Argentina, Corrientes, Santo Tomé; Hansen 1999b: 163 [catalog]; Clarkson and Ferreira-Jr. 2014: 400 [faunistic treatment]; Silva et al. 2018: 9 [faunistic treatment].

Distribution: Neotropical: Argentina, Brazil (Mato Grosso do Sul, Piauí), Paraguay, Venezuela.

### *Peltochares* Régimbart, 1907

***Peltochares atropiceus* (Régimbart, 1903) comb. nov.**

*Helochares atropiceus* Régimbart, 1903b: 53 – Vietnam [“Cochinchine”] (Ho Chi Minh [“Saigon”]; My Tho); Cambodia (Phnom Penh); Indonesia (Sumatra, Borneo, New Guinea); not synonym of *Helochares taprobanicus* Sharp, as in d’Orchymont 1923b: 419 and Hansen 1999b: 163.

Helochares (s. str.) atropiceus Régimbart, 1903; Hebauer 2001: 10 [specific rank confirmed; lectotype designated]; Hansen 2004: 52 [checklist]; Hebauer and Ryndevich 2005: 45 [new record]; Fikáček et al. 2015: 61 [checklist]; Jia and Tang 2018b: 9 [redescription; new record].

Helochares (s. str.) atropiceus Sharp; Hebauer 2002a: 24 [author attribution in error; new record].

Helochares (s. str.) ohkurai Satô, 1976: 21 – Japan, Nansei-shoto archipelago [“Ryukyus”], Iriomote-jima Is., Ôhara-Ôtomi; Hansen 1999b: 162 [catalog]; Hebauer 2001: 11 [synonymy].

Distribution: Australasian: Papua New Guinea [“Nouvelle Guinée”]. Indo-Malayan: Bangladesh, Cambodia, China (Guangdong, Guangxi, Guizhou, Hong Kong, Jiangxi, Macao), Indonesia (Borneo, Sumatra), Nepal, Thailand, Vietnam. Palearctic: Japan (Nansei Islands).

***Peltochares ciniensis* (Hebauer, Hendrich & Balke, 1999) comb. nov.**

Helochares (s. str.) ciniensis Hebauer, Hendrich & Balke, 1999: 341 – Malaysia, Pahang, Lake Cini, lakeside nr. Rimba Resort; Short and Hebauer 2006: 335 [catalog].

Distribution: Indo-Malayan: Malaysia.

***Peltochares conspicuus* Régimbart, 1907**

*Peltochares conspicuus* Régimbart, 1907: 49 – Gabon, Cape Lopez, Rembo N’Comi; Balfour-Browne 1950b: 60 [faunistic treatment]; Bertrand 1962: 1101 [description of larva]; Hansen 1999b: 172 [catalog]; Hebauer 2006a: 27 [checklist].

Distribution: Afrotropical: Democratic Republic of the Congo, Gabon, Ghana, Ivory Coast.

***Peltochares discus* (Hebauer, Hendrich & Balke, 1999) comb. nov.**

Helochares (s. str.) discus Hebauer, Hendrich & Balke, 1999: 342; Hebauer 2001b: 11 [taxonomic treatment]; Short and Hebauer 2006: 336 [catalog].

Distribution: Indo-Malayan: Indonesia (Sumatra), Malaysia.

***Peltochares foveicollis* (Montrouzier, 1860) comb. nov.**

*Stagnicola foveicollis* Montrouzier, 1860: 247 – New Caledonia, Île Art [“Nouvelle-Calédonie, Art”].

*Helochares foveicollis* (Montrouzier, 1860); Bedel 1880: CXLVIII [synonymy].

*Philhydrus burrundiensis* Blackburn, 1890: 447 – Australia, Northern Territory, Burrundie; d’Orchymont 1943b: 6 [synonymy in doubt].

*Neohydrobius burrundiensis* (Blackburn, 1890); Blackburn 1898: 221 [new genus; new combination].

Helochares (s. str.) burrundiensis (Blackburn, 1890); d’Orchymont 1919b: 228 [synonymy].

Helochares (s. str.) foveicollis (Montrouzier, 1860); d’Orchymont 1937e: 154 [checklist]; Watts, 1995: 118 [taxonomic treatment]; Hansen 1999: 160 [catalog]; Watts 2002: 122 [description of larva]; Short 2010: 312 [catalog].

Distribution: Australasian: Australia (Australian Capital Territory, New South Wales, Northern Territory, Queensland, Western Australia), New Caledonia, Papua New Guinea.

***Peltochares longipalpis* (Murray, 1859) comb. nov.**

Philhydrus (s. str.) longipalpis Murray, 1859: 123 – Nigeria, Calabar [“Old Calabar”].

*Helochares longipalpis* (Murray, 1859); Régimbart 1903a: 26 [faunistic treatment]; Bird et al. 2017 [faunistic treatment].

Helochares (s. str.) longipalpis (Murray, 1859); Balfour-Browne 1950b: 58 [faunistic treatment]; Balfour-Browne 1952a: 129 [faunistic treatment]; Balfour-Browne 1957: 22 [faunistic treatment]; Hansen and Hebauer 1988: 29 [in key]; Hebauer 1994: 112 [faunistic treatment]; Hebauer 1995a: 265 [faunistic treatment]; Hebauer 1996: 7 [faunistic treatment]; Hansen 1999b: 161 [catalog]; Hebauer 2001: 12 [taxonomic treatment]; Hebauer 2005: 39 [checklist]; Hebauer 2006a: 25 [checklist]; Hansen 2004: 52 [checklist]; Fikáček et al. 2015: 61 [checklist]; Salah and Régil Cueto 2017: 265 [checklist].

*Helochares filipalpis* Sharp, 1903: 6 – South Sudan [Sudan], Jebel Ahmed Agha [“Gebel Ahmed Agha”]; d’Orchymont 1943c: 7 [synonymy].

Distribution: Afrotropical: Angola, Benin, Botswana, Burkina Faso, Burundi, Cameroon, Central African Republic, Chad, Democratic Republic of the Congo, Ethiopia, Gabon, Gambia, Ghana, Guinea, Ivory Coast, Kenya, Liberia, Madagascar, Malawi, Mozambique, Namibia, Niger, Nigeria, Republic of the Congo, Rwanda, Senegal, Sierra Leone, Somalia, Republic of South Africa, South Sudan, Tanzania, Togo, Uganda, Western Sahara, Zambia, Zimbabwe. Palearctic: Canary Islands, Egypt, Israel.

NOTE: This species almost certainly represents a species complex. Aside from *Peltochares conspicuus*, which is much more morphologically divergent, this is the only species of *Peltochares* currently recorded from sub-Saharan Africa (and Madagascar), although we have seen evidence for multiple species based on aedeagal and molecular data. It is likely that several species exist under this name, and they will need to be teased apart in a future revision of the genus.

***Peltochares papuensis* (Hebauer, 1995) comb. nov.**

Helochares (s. str.) papuensis Hebauer, 1995b: 8 – Indonesia, Papua [W. Neuguinea; Irian Jaya], Paniai province, Wanggar-Kali Bumi; Hansen 1999b: 163 [catalog].

Distribution: Australasian: Indonesia (Papua).

***Peltochares taprobanicus* (Sharp, 1890) comb. nov.**

Helochares (s. str.) taprobanicus Sharp, 1890: 351 – Sri Lanka, Colombo [“(Ceylon): Colombo”]; d’Orchymont 1928: 108 [faunistic treatment]; Hebauer 1995b: 8 [faunistic treatment]; Hansen 1999b: 163 [catalog]; Hebauer 2001: 11 [taxonomic treatment; lectotype designated].

Helochares (s. str.) lacustris Hebauer, Hendrich & Balke, 1999: 342; Hebauer 2001: 11 [synonymy]; Hebauer and Ryndevich 2005: 45 [new record]; Short and Hebauer 2006: 336 [catalog].

Distribution: Indo-Malayan: Indonesia (Sumatra), Laos, Malaysia, Nepal, Sri Lanka, Thailand, Vietnam.

### *Primocerus* Girón & Short, 2019

***Primocerus cuspidis* Girón & Short, 2019**

*Primocerus cuspidis* Girón & Short, 2019: 144 – Venezuela, Amazonas, Tobogán de la Selva, old “Tobogancito”, 5°23.207'N, 67°36.922'W.

Distribution: Neotropical: Venezuela.

***Primocerus gigas* Girón & Short, 2019**

*Primocerus gigas* Girón & Short, 2019: 145 – Venezuela, Amazonas, Cerro de la Neblina, camp II, 0°50'N, 65°59'W.

Distribution: Neotropical: Venezuela.

***Primocerus maipure* Girón & Short, 2019**

*Primocerus maipure* Girón & Short, 2019: 146 – Venezuela, Amazonas, ca. 15 km S of Puerto Ayacucho, 5°30.623'N, 67°36.109'W.

Distribution: Neotropical: Venezuela.

***Primocerus neutrum* Girón & Short, 2019**

*Primocerus neutrum* Girón & Short, 2019: 147 – Venezuela, Bolívar, along La Escalera, 6°2'10.5"N, 61°23'57.8"W.

Distribution: Neotropical: Guyana, Suriname, Venezuela.

***Primocerus ocellatus* Girón & Short, 2019**

*Primocerus ocellatus* Girón & Short, 2019: 148 – Venezuela, Amazonas, Cerro de la Neblina, Camp XII, near Pico Phelps.

Distribution: Neotropical: Venezuela.

***Primocerus petilus* Girón & Short, 2019**

*Primocerus petilus* Girón & Short, 2019: 148 – Brazil, Pará: Alenquer, Vale do Paraíso, ca. 55 km N of Alenquer, 1.49292S, 54.51566W.

Distribution: Neotropical: Brazil (Pará).

***Primocerus pijiguaense* Girón & Short, 2019**

*Primocerus pijiguaense* Girón & Short, 2019: 149 – Venezuela, Bolívar, Los Pijiguaos, 6°35.617'N, 66°49.238'W

Distribution: Neotropical: Venezuela.

***Primocerus semipubescens* Girón & Short, 2019**

*Primocerus semipubescens* Girón & Short, 2019: 150 – Guyana, Region VIII, Ayanganna Airstrip, trail from Blackwater Creek Camp to Potaro River, 5°17.823'N, 59°50.000'W.

Distribution: Neotropical: Guyana.

***Primocerus striatolatus* Girón & Short, 2019**

*Primocerus striatolatus* Girón & Short, 2019: 151 – Suriname, Sipaliwini District, Camp 4 (high) Kasikasima, 2°58'36.7782"N, 55°24'40.986"W.

Distribution: Neotropical: Suriname.

### *Quadriops* Hansen, 1999

***Quadriops acroreius* Girón & Short, 2017**

*Quadriops acroreius* Girón & Short, 2017: 123 – Suriname, Sipaliwini District, Camp 1: Upper Palemeu, 2°28'37.1994"N, 55°37'45.876"W.

Distribution: Neotropical: Suriname, French Guiana.

***Quadriops clusia* Girón & Short, 2017**

*Quadriops clusia* Girón & Short, 2017: 125 – Suriname, Brokopondo District, Brownsberg Nature Park, Leo Val trail, nr. pump station, 4.95069'N, -55.18599.

Distribution: Neotropical: Guyana, Suriname, Brazil (Amazonas).

***Quadriops dentatus* Hansen, 1999**

*Quadriops dentatus* Hansen, 1999a: 134 – Venezuela, Bolivar, 105 km S El Dorado; Hansen 1999b: 155 [catalog]; Girón and Short 2017: 127 [new records].

Distribution: Neotropical: Venezuela, French Guiana, Suriname.

***Quadriops depressus* Hansen, 1999**

*Quadriops depressus* Hansen, 1999: 136 – Peru, Departamento Loreto, 1.5 km N Teniente Lopez 2°35.66'S, 76°06.92'W; Hansen 1999b: 155 [catalog]; Girón and Short 2017: 128 [new records].

*Quadriops amazonensis* García, 2000: 59 – Venezuela, Amazonas, Municipio Guinia, Yavita, Caño Chivichi; Girón and Short 2017: 128 [synonymy]; Short and Hebauer 2006: 338 [catalog].

*Quadriops politus* Hansen, 1999: 135 – Peru, Departamento Loreto, Campamento San Jacinto, 2°18.75'S, 75°51.77'W; Hansen 1999b: 155; Girón and Short 2017: 128 [synonymy]

Distribution: Neotropical: Ecuador, Peru, Venezuela.

***Quadriops reticulatus* Hansen, 1999**

*Quadriops reticulatus* Hansen, 1999: 135 – Costa Rica, Puntarenas, Las Alturas (Stanford Biological Station), ca. 29 km NE San Vito; Hansen 1999b: 155 [catalog]; Girón and Short 2017: 130 [new records].

Distribution: Neotropical: Costa Rica, Panama.

***Quadriops similaris* Hansen, 1999**

*Quadriops similaris* Hansen, 1999: 136 – Venezuela, Bolivar, 105 km S El Dorado; Hansen 1999b: 155 [catalog]; Girón and Short 2017: 134 [new records].

Distribution: Neotropical: Venezuela, Guyana, Suriname, French Guiana.

### *Radicitus* Short & García, 2014

***Radicitus ayacucho* Short & García, 2014**

*Radicitus ayacucho* Short & García, 2014: 252 – Venezuela, Amazonas State, Tobogan de la Selva, 5°23.207'N, 67°36.922'W.

Distribution: Venezuela.

***Radicitus granitum* Short & García, 2014**

*Radicitus granitum* Short & García, 2014: 254 – Venezuela, Bolívar State, Los Pijiguaos, 6°35.617'N, 66°49.238'W.

Distribution: Venezuela.

***Radicitus surinamensis* Short & García, 2014**

*Radicitus surinamensis* Short & García, 2014: 257 – Suriname, Sipaliwini Department, Mt. Kasikasima, 2°58.613'N, 55°24.683'W.

Distribution: Suriname.

### *Sindolus* Sharp, 1882

***Sindolus femoratus* (Brullé, 1841)**

Hydrophilus (Philydrus) femoratus Brullé, 1841: 59 – Argentina [“province de Corrientes”].

*Hydrobius femoratus* (Brullé, 1841); Gemminger and Harold 1868a: 479 [checklist].

*Helochares femoratus* (Brullé, 1841); Bedel 1881: XCV.

Helochares (Sindolus) femoratus (Brullé, 1841); d’Orchymont 1926b: 236; Fernández and Kehr 1994 [annual life cycle]; Fernández and Kehr 1995 [spatial and temporal distribution]; Hansen 1999: 157 [catalog]; Fernández 2004 [description of immature stages]; Clarkson and Ferreira-Jr 2014a: 403 [faunistic treatment]; Alves et al. 2020: 583 [faunistic treatment].

? *Hydrobius spadiceus* Dejean, 1833: 134; nom. nud.; Mulsant 1844b: 380 [synonym of *Philhydrus spadiceus* Mulsant]

? *Philhydrus spadiceus* Mulsant, 1844b: 380 – French Guiana (Cayenne) and Colombia [“Nouvelle-Grenade”]; d’Orchymont 1929: 95 [synonym doubtful].

? Enochrus (Lumetus) spadiceus (Mulsant, 1844); Zaitzev 1908: 389 [catalog].

*Helochares gravidus* Bruch, 1915: 452 – Argentina, La Plata (“Tiro Federal”; Formosa (Puerto Bouvier); d’Orchymont 1926b: 236 [synonymy].

Helochares (Sindolus) gravidus Bruch, 1915; Knisch 1924: 199 [catalog].

*Sindolus femoratus* (Brullé, 1841); Short et al. 2021: 11 [new combination].

Distribution: Neotropical: Argentina, Brazil (Bahía, Pernambuco, Piauí, Rio de Janeiro, Rio Grande do Sul), Colombia [in doubt; d’Orchymont, 1943d: 56], French Guiana [in doubt; d’Orchymont, 1943d: 56], Lesser Antilles (Antigua).

***Sindolus mesostitialis* (Fernández, 1981)**

Helochares (Sindolus) mesostitialis Fernández, 1981: 189 – Argentina, Santa Fe, Dept. Garay, Colonia Mascias; Hansen 1999b: 158 [catalog]; Clarkson and Ferreira-Jr. 2014: 400 [faunistic treatment].

*Sindolus mesostitialis* (Fernández, 1981); Short et al. 2021: 11 [new combination].

Distribution: Neotropical: Argentina, Brazil (Mato Grosso do Sul).

***Sindolus mini* (Fernández, 1982)**

Helochares (Sindolus) mini Fernández, 1982b: 89 – Argentina, Santa Fe, Chaco prov., lag. La Cava, Barranqueras; Hansen 1999b: 158 [catalog].

*Sindolus mini* (Fernández, 1982); Short et al. 2021: 11 [new combination].

Distribution: Neotropical: Argentina, Paraguay.

***Sindolus mundus* Sharp, 1882**

*Sindolus mundus* Sharp, 1882: 73 – Mexico, Oaxaca.

Helochares (Sindolus) mundus (Sharp, 1882); Knisch 1924: 199 [checklist]; Hansen 1999b: 158 [catalog]; Short 2005: 219 [new records].

Distribution: Neotropical: Costa Rica, Mexico, Nicaragua.

***Sindolus optatus* Sharp, 1882**

*Sindolus optatus* Sharp, 1882: 72 – Guatemala, Paso Antonio.

Helochares (Sindolus) optatus (Sharp, 1882); Knisch 1924: 199 [checklist]; Hansen 1999b: 158 [catalog]; Short 2005: 220 [new records].

Helochares (s. str.) guatemalensis Knisch, 1921a: 68 – Guatemala; d’Orchymont 1937b: 253 [synonymy].

Helochares (Sindolus) guatemalensis Knisch, 1921; Knisch 1924: 199 [catalog].

Distribution: Neotropical: Costa Rica, Guatemala, Mexico.

***Sindolus spatulatus* (Fernández, 1981)**

Helochares (Sindolus) spatulatus Fernández, 1981: 191 – Argentina, Corrientes.

*Sindolus spatulatus* (Fernández, 1981); Short et al. 2021: 11 [new combination].

Distribution: Neotropical: Argentina, Paraguay.

***Sindolus talarum* (Fernández, 1983)**

Helochares (Sindolus) talarum Fernández, 1983: 440 – Argentina, Buenos Aires, lag. Los Talas [original description includes description of immature stages].

*Sindolus talarum* (Fernández, 1983); Short et al. 2021: 11 [new combination].

Distribution: Neotropical: Argentina.

***Sindolus ventricosus* (Bruch, 1915)**

Hydrophilus (Philydrus) gibbus Brullé, 1841: 58 (primary homonym of *Hydrophilus gibbus* Illiger, 1801 and *Hydrophilus gibbus* Thunberg, 1820); d’Orchymont, 1926b: 236 (sub nom. *gibbus*; not synonym of *atratus* Bruch, as in Balfour-Browne 1939: 293).

*Philhydrus gibbus* (Brullé, 1841); Lacordaire 1854: 457.

*Helochares gibbus* (Brullé, 1841); Bedel 1881: XCV.

Helochares (Sindolus) gibbus (Brullé, 1841); d’Orchymont 1926b: 236.

*Helochares ventricosus* Bruch, 1915: 452; Fernández, 1982a: 36 [specific rank confirmed; lectotype designated; not synonym of *atratus* Bruch, 1915, as in Balfour-Browne, 1939: 293].

Helochares (Sindolus) ventricosus Bruch, 1915; Clarkson and Ferreira-Jr. 2014: 400 [faunistic treatment].

*Sindolus ventricosus* (Bruch, 1915); Short et al. 2021: 11 [new combination].

Distribution: Neotropical: Argentina, Bolivia, Brazil (Amazonas, Mato Grosso do Sul, Pernambuco), Paraguay, Uruguay.

### *Tobochares* Short & García, 2007

***Tobochares akoerio* Girón & Short, 2021**

*Tobochares akoerio* Girón & Short, 2021: 120 – Suriname: Sipaliwini District, 2.46554°N, 55.7700°W, Camp 2, Grensgebergte Rock.

Distribution: Neotropical: Suriname.

***Tobochares arawak* Girón & Short, 2021**

*Tobochares arawak* Girón & Short, 2021: 122 – Guyana: Region VIII, 5°0.730'N, 59°38.965'W, Upper Potaro Camp I, ca. 7 km NW of Chenapau, top of falls on Potaro River.

Distribution: Neotropical: Guyana.

***Tobochares anthonyae* Girón & Short, 2021**

*Tobochares anthonyae* Girón & Short, 2021: 125 – Venezuela: Bolívar, 6°13'4.6"N, 67°14'26.4"W; ca. 25 km E of El Burro.

Distribution: Neotropical: Venezuela.

***Tobochares atures* Girón & Short, 2021**

*Tobochares atures* Girón & Short, 2021: 126 – Venezuela: T.F. Amazonas, Puerto Ayacucho (40 km S), El Tobogán, Caño Coromoto.

Distribution: Neotropical: Venezuela.

***Tobochares benettii* Girón & Short, 2021**

*Tobochares benettii* Girón & Short, 2021: 106 – Brazil: Amazonas: Rio Preto da Eva, -2.678466, -59.401714, ca. 32 km W of Rio Preto da Eva.

*Tobochares* sp. B, Short et al. 2021.

Distribution: Neotropical: Brazil (Amazonas).

***Tobochares canaima* Girón & Short, 2021**

*Tobochares canaima* Girón & Short, 2021: 128 – Venezuela: Bolívar: 5°51'N, 62°33'W, 1700 m, Auyan-tepui.

Distribution: Neotropical: Venezuela.

***Tobochares canaliculatus* Kohlenberg & Short, 2017**

*Tobochares canaliculatus* Kohlenberg & Short, 2017: 119 – Venezuela, Amazonas State, Tobogan de la Selva, old “tobogancito”, 5°23.207'N, 67°36.922'W.

Distribution: Neotropical: Venezuela.

***Tobochares canthus* Kohlenberg & Short, 2017**

*Tobochares canthus* Kohlenberg & Short, 2017: 122 – Venezuela, Amazonas State, Tobogan de la Selva, old “tobogancito”, 5°23.207'N, 67°36.922'W.

Distribution: Neotropical: Venezuela.

***Tobochares communis* Girón & Short, 2021**

*Tobochares communis* Girón & Short, 2021: 129 – Suriname: Sipaliwini District, 4°40.432'N, 56°11.079'W, Raleighvallen Nature Reserve, base of Voltzberg.

*Tobochares* sp. 1B, Short et al. 2021.

Distribution: Neotropical: Brazil (Amapá, Roraima), Guyana, Suriname, Venezuela.

***Tobochares emarginatus* Kohlenberg & Short, 2017**

*Tobochares emarginatus* Kohlenberg & Short, 2017: 123 – Suriname: Sipaliwini District, Camp 4 (high) Kasikasima, 2°58.613'N, 55°24.683'W.

Distribution: Neotropical: Suriname.

***Tobochares fusus* Girón & Short, 2021**

*Tobochares fusus* Girón & Short, 2021: 117 – Brazil: Amapá: Oiapoque, 3.85039, -51.81683, 17 m, Oiapoque (ca. 1 km E).

Distribution: Neotropical: Brazil (Amapá), French Guiana.

***Tobochares goias* Girón & Short, 2021**

*Tobochares goias* Girón & Short, 2021: 109 – Brazil: Goiás: Cristalina, -16.87004, -47.61716; 947 m; Cristalina Balneario Lajes.

*Tobochares* sp. C, Short et al. 2021.

Distribution: Neotropical: Brazil (Goiás).

***Tobochares kappel* Girón & Short, 2021**

*Tobochares kappel* Girón & Short, 2021: 133 – Suriname: Sipaliwini District, 3°47.479'N, 56°8.968'W, CSNR: near Kappel airstrip.

Distribution: Neotropical: Suriname.

***Tobochares kasikasima* Short, 2013**

*Tobochares kasikasima* Short, 2013: 83 – Suriname, Sipaliwini District, Camp 4 (high) Kasikasima, 2°58.613'N, 55°24.683'W; Kohlenberg and Short 2017: 124 [redescription].

Distribution: Neotropical: Suriname.

***Tobochares kolokoe* Girón & Short, 2021**

*Tobochares kolokoe* Girón & Short, 2021: 134 – Suriname: Sipaliwini District, CSNR: Tafelberg Summit, Arrowhead Basin.

Distribution: Neotropical: Suriname.

***Tobochares kusad* Kohlenberg & Short, 2017**

*Tobochares kusad* Kohlenberg & Short, 2017: 126 – Guyana: Region IX, Kusad Mts., Mokoro Creek, 2 48.531'N, 59 51.900'W; Girón and Short 2021a: 114 [new record].

Distribution: Neotropical: Brazil (Roraima), Guyana.

***Tobochares luteomargo* Girón & Short, 2021**

*Tobochares luteomargo* Girón & Short, 2021: 115 – Venezuela: Bolívar State, 7°41'23.6"N, 64°1'56.0"W, 134 m, ca. 14 km E of Río Aro.

*Tobochares* sp. 10, Short et al., (2021).

Distribution: Neotropical: Venezuela.

***Tobochares microps* Girón & Short, 2021**

*Tobochares microps* Girón & Short, 2021: 135 – Suriname: Sipaliwini District, N3 53.359’ W56 10.052’, CSNR: Tafelberg Summit, near South Rim.

*Tobochares* sp. 2A, Short et al. 2021.

Distribution: Neotropical: Suriname.

***Tobochares pallidus* Kohlenberg & Short, 2017**

*Tobochares pallidus* Kohlenberg & Short, 2017: 130 – Venezuela: Amazonas State, Tobogan de la Selva, old “tobogancito”, 5°23.207'N, 67°36.922'W.

Distribution: Neotropical: Venezuela.

***Tobochares pemon* Girón & Short, 2021**

*Tobochares pemon* Girón & Short, 2021: 136 – Venezuela: Bolívar, 5°51'N, 62°33'W, Auyan-tepui.

Distribution: Neotropical: Venezuela.

***Tobochares romanoae* Girón & Short, 2021**

*Tobochares romanoae* Girón & Short, 2021: 137 – Brazil: Roraima, Amajari, 3°36.381'N, 61°42.878'W, Serra do Tepequém, Igarape Preto Negro, Cachoeira Leje Preta.

Distribution: Neotropical: Brazil (Roraima).

***Tobochares sipaliwini* Short & Kadosoe, 2011**

*Tobochares sipaliwini* Short & Kadosoe, 2011: 85 – Suriname, Sipaliwini District, Camp 2, on Sipaliwini River, Inselberg, 2 10.973'N, 56 47.235'W; Kohlenberg and Short 2017: 132 [redescription]; Girón and Short 2021a: 115 [new record].

Distribution: Neotropical: Brazil (Roraima), Guyana, Suriname.

***Tobochares striatus* Short, 2013**

*Tobochares striatus* Short, 2013: 83 – Suriname, Sipaliwini District, 2.24554°N, 55.77000°W, Camp 2 Grensgebergte Rock; Kohlenberg and Short 2017: 136 [redescription]; Girón and Short 2021a: 115 [new record].

Distribution: Neotropical: Suriname.

***Tobochares sulcatus* Short & García, 2007**

*Tobochares sulcatus* Short & García, 2007: 4 – Venezuela: Amazonas State, Tobogan de la Selva, ca. 40 km S of Puerto Ayacucho, margin of Rio Coromoto; Short and Fikáček 2011: 91 [catalog]; Kohlenberg and Short 2017: 140 [redescription].

Distribution: Neotropical: Venezuela.

### *Troglochares* Spangler, 1981

***Troglochares ashmolei* Spangler, 1981**

*Troglochares ashmolei* Spangler, 1981a: 318 – Ecuador, Morona-Santiago prov., Los Tayos Cave; Hansen 1999b: 156 [catalog].

Distribution: Neotropical: Ecuador.

## Supplementary Material

XML Treatment for Acidocerinae

XML Treatment for Acidocerus

XML Treatment for Agraphydrus

XML Treatment for Aulonochares

XML Treatment for Batochares

XML Treatment for Chasmogenus

XML Treatment for Colossochares

XML Treatment for Crephelochares

XML Treatment for Crucisternum

XML Treatment for Ephydrolithus

XML Treatment for Globulosis

XML Treatment for Helobata

XML Treatment for Helochares

XML Treatment for Helopeltarium

XML Treatment for Katasophistes

XML Treatment for Nanosaphes

XML Treatment for Novochares

XML Treatment for Peltochares

XML Treatment for Primocerus

XML Treatment for Quadriops

XML Treatment for Radicitus

XML Treatment for Sindolus

XML Treatment for Tobochares

XML Treatment for Troglochares
